# Space Quasi-Periodic Steady Euler Flows Close to the Inviscid Couette Flow

**DOI:** 10.1007/s00205-024-02028-1

**Published:** 2024-09-11

**Authors:** Luca Franzoi, Nader Masmoudi, Riccardo Montalto

**Affiliations:** 1 Dipartimento di Matematica “Federigo Enriques”, Università degli Studi di Milano, Via Cesare Saldini 50, 20133 Milan, Italy; 2 NYUAD Research Institute, New York University Abu Dhabi, NYUAD Saadiyat Campus, 129188 Abu Dhabi, UAE; 3 Courant Institute of Mathematical Sciences, New York University, 251 Mercer Street, New York, NY 10012 USA

**Keywords:** 35Q31, 37K55, 76B03

## Abstract

We prove the existence of steady *space quasi-periodic* stream functions, solutions for the Euler equation in a vorticity-stream function formulation in the two dimensional channel ${{\mathbb {R}}}\times [-1,1]$. These solutions bifurcate from a prescribed shear equilibrium near the Couette flow, whose profile induces finitely many modes of oscillations in the horizontal direction for the linearized problem. Using a Nash–Moser implicit function iterative scheme, near such equilibrium we construct small amplitude, space reversible stream functions, slightly deforming the linear solutions and retaining the horizontal quasi-periodic structure. These solutions exist for most values of the parameters characterizing the shear equilibrium. As a by-product, the streamlines of the nonlinear flow exhibit Kelvin’s cat eye-like trajectories arising from the finitely many stagnation lines of the shear equilibrium.

## Introduction

In the two-dimensional finite channel ${{\mathbb {R}}}\times [-1,1]$, we consider a stationary, incompressible inviscid fluid whose stream function $\psi (x,y):{{\mathbb {R}}}\times [-1,1 ]\rightarrow {{\mathbb {R}}}$ solves the stationary Euler equation in vorticity-stream function formulation

1.1 $$\begin{aligned} \{ \psi ,\Delta \psi \} := \psi _x (\Delta \psi )_y - \psi _y (\Delta \psi )_x =0, \end{aligned}$$

coupled with the impermeability condition at the boundary

1.2 $$\begin{aligned} \psi _x =0 \quad \text {on } \ \{y=\pm 1\}. \end{aligned}$$

In [38], Lin and Zeng showed on the finite periodic channel, in a $H_{x,y}^s$-neighbourhood of the Couette flow (in the vorticity space), the existence of space periodic steady solutions of (1.1) when $s<\frac{3}{2}$ and the non-existence of non-parallel traveling solutions when $s>\frac{3}{2}$. Namely, the regularity threshold $s=\frac{3}{2}$ discriminates between the presence or not of damping phenomena for the nonlinear evolution of non viscous fluids. The goal of the present paper is to give a new insight to their result when the setting is extended to the quasi-periodic case. We give now the informal statement of our result.

### InformalTheorem

Let $\kappa _{0}\in {{\mathbb {N}}}$. There exist $\varepsilon _0>0$ small enough and a family of stationary solutions $(\psi _{\varepsilon }(x,y)=\breve{\psi }_{\varepsilon }(\textbf{x},y)|_{\textbf{x}={\widetilde{\omega }}x})_{\varepsilon \in [0,\varepsilon _0]}$ of the Euler equation (1.1) in the finite channel $(x,y)\in {{\mathbb {R}}}\times [-1,1]$ that are quasi-periodic in the horizontal direction $x \in {{\mathbb {R}}}$ for some frequency vector ${\widetilde{\omega }}\in {{\mathbb {R}}}^{\kappa _{0}}$, with $\textbf{x}={\widetilde{\omega }}x\in {{\mathbb {T}}}^{\kappa _{0}}$. Such family bifurcates from a shear equilibrium $\psi _{{\mathfrak {m}}}(y)$ and can be chosen to be arbitrarily close to the stream function of the Couette flow $\psi _{\textrm{cou}}(y):=\tfrac{1}{2} y^2$ in $H_{\textbf{x}}^{s}H_y^{7/2-}({{\mathbb {T}}}^{\kappa _{0}}\times [-1,1])$, with $s>0$ sufficiently large.

The rigorous statement of the result is given in Theorem 1.1.

Understanding as much as possible of the fluid behaviour around shear flows is one of the main interests for the hydrodynamic stability research. Around the Couette flow, the simplest among the nontrivial shear flows, Kelvin [35] and Orr [40] proved with experiments and with computations the damping of inviscid flows for the linearized Euler equations at the shear equilibrium, which at first was a surprising result in (apparent) contrast with the (essential) Hamiltonian nature of the equations. Restricting our exposition to the two-dimensional setting, the first rigorous justification in the nonlinear case was provided by Bedrossian and Masmoudi [5] and Deng and Masmoudi [21], who proved the asymptotic stability to the planar Couette flow in ${{\mathbb {T}}}\times {{\mathbb {R}}}$ under perturbations in Gevrey regularity. These results follow the work of Mouhot and Villani [39] on the nonlinear Landau damping for the Vlasov equation. Other extensions to the inviscid damping near the Couette flow include Ionescu and Jia [31] in the finite periodic channel, Yang and Lin [50], Bianchini, Coti Zelati and Dolce [13] for stratified fluids and Antonelli, Dolce and Marcati [1] in the compressible case. For other shear flows, Zillinger [51] and Wei, Zhang and Zhao [49] proved linear inviscid damping for monotonic shears. On the contrary, the existence of non-trivial, meaning non-shear, Euler flows that cannot exhibit damping was proved by Lin and Zeng [38] and Castro and Lear [17] around the Couette flow, and by Coti Zelati, Elgindi and Widmayer [18] around non-monotone shears.

In order to look for quasi-periodic invariant structures, we base our approach on the KAM (Kolmogorov–Arnold–Moser) theory for Partial Differential Equations. This field started in the 1990s, with the pioneering papers of Bourgain [14], Craig and Wayne [19], Kuksin [36], Wayne [48]. We refer to the recent review article [6] for a complete list of references on this topic. In the last years, together with the Nash–Moser implicit function theorem, these techniques have been developed in order to study time quasi-periodic solutions for PDEs arising from fluid dynamics. For the two dimensional water waves equations, we mention Berti and Montalto [12], Baldi, Berti, Haus and Montalto [2] for time quasi-periodic standing waves and Berti, Franzoi and Maspero [8, 9], Feola and Giuliani [24] for time quasi-periodic traveling wave solutions. Recently, the existence of time quasi-periodic solutions was proved for the contour dynamics of vortex patches in active scalar equations. We mention Berti, Hassainia and Masmoudi [10] for vortex patches of the Euler equations close to Kirchhoff ellipses, Hmidi and Roulley [30] for the quasi-geostrophic shallow water equations, Hassainia, Hmidi and Masmoudi [27] for generalized surface quasi-geostrophic equations, Roulley [43] for Euler-$\alpha$ flows, Hassainia and Roulley [29] for Euler equations in the unit disk close to Rankine vortices and Hassainia, Hmidi and Roulley [28] for 2D Euler annular vortex patches. Time quasi-periodic solutions were also constructed for the 3D Euler equations with time quasi-periodic external force [3] and for the forced 2D Navier–Stokes equations [25] approaching in the zero viscosity limit time quasi-periodic solutions of the 2D Euler equations for all times. We finally mention that time quasi-periodic solutions for the Euler equations were constructed also by Crouseilles and Faou [20] in 2D, with a very recent extension by Enciso, Peralta-Salas and Torres de Lizaur [22] in 3D and even dimensions: we remark that these latter solutions are engineered so that there are no small divisors issues to deal with, with consequently much easier proofs and a drawback of not having information on the eventual stability of the solutions.

The paragraph above shows how KAM normal form techniques started very recently to be developed in Fluid Dynamics in order to construct quasi-periodic solutions in time. On the contrary, there are only few works where the question of the quasi-periodicity *in space* is considered. To the best of our knowledge, the first result of space bi-periodic solutions to PDEs is due to Scheurle [45] for a semilinear equation on a two-dimensional strip in analytic regularity, whose solutions locally bifurcate from bi-periodic solutions of the linearized system at the equilibrium. Then, Iooss and Los [32] proved the bifurcation of stationary solutions in the hydrodynamic stability problem for forced Navier–Stokes equations on cylindrical domains, extended to time-periodic solutions in Iooss and Mielke [33]. Spatially bi-periodic solutions were studied in Bridges and Rowlands [16] for the linear stability analysis of the Ginzburg–Landau equation, and in Bridges and Dias [15] for stationary 2D gravity-capillary water waves. The general case with more than two spatial frequencies was considered by Valls [47] and Poláčik and Valdebenito [42] for elliptic equations on ${{\mathbb {R}}}^N \times {{\mathbb {R}}}$. All these results share the same idea of using one space direction as a temporal one, assuming to have hyperbolic modes for the linearized elliptic operators. The persistence of spatially quasi-periodic oscillations is then proved in [45] via a Nash–Moser implicit function theorem, in [32] with normalization techniques on the infinite dimensional “spatial phase space”, while in [42, 47] with a center manifold reduction on a finite dimensional system together with a Birkhoff normal form to ensure the application of the standard KAM theorems. As we shall see later, the last two strategies do not look suitable for our problem, since we can only establish the existence of the nonlinear elliptic equation to solve and few properties on the regularity of the nonlinearity, which do not seem enough to check the assumptions for the KAM theorem. Therefore, for our purposes, we preferred to use the Nash–Moser approach as developed by Berti and Bolle [7], which provides also a better description of the final solutions. We conclude by mentioning that spatial dynamics techniques in Fluid Dynamics were applied by Groves and Wahlén [26] to study the existence of small amplitude, solitary gravity-capillary water waves with arbitrary distribution of vorticity.

### Main Result

Our construction starts with prescribing a potential function $Q_{{\mathfrak {m}}}(y)$, even in *y*, depending on a parameter ${\mathfrak {m}}\gg 1$ such that, in the limit ${\mathfrak {m}}\rightarrow \infty$, it approaches the classical potential well

1.3 $$\begin{aligned} Q_{{\mathfrak {m}}}(y)=Q_{{\mathfrak {m}}}(\texttt{E},\texttt{r};y) {\mathop {\rightarrow }\limits ^{{\mathfrak {m}}\rightarrow \infty }} Q_{\infty }(\texttt{E},\texttt{r};y) := {\left\{ \begin{array}{ll} 0 &  \quad |y|>\texttt{r},\\ -\texttt{E}^2 &  \quad |y|<\texttt{r}, \end{array}\right. } \end{aligned}$$

where $\texttt{r}\in (0,1)$ is the width of the well and $\texttt{E}>1$ is related to its depth. The potential $Q_{{\mathfrak {m}}}$ is analytic in all its entries and its derivatives approach the derivatives of $Q_{\infty }$ uniformly on compact sets avoiding the points $y=\pm \texttt{r}$. The explicit expression of $Q_{{\mathfrak {m}}}(y)$ is provided in (1.27). Moreover, the parameters $\texttt{E}$ and $\texttt{r}$ are related by the analytic constraint

1.4 $$\begin{aligned} \texttt{E}\texttt{r}= \left( \kappa _0+\tfrac{1}{4}\right) \pi . \end{aligned}$$

The value $\kappa _0\in {{\mathbb {N}}}$ is fixed from the very beginning and it prescribes via (1.4) the exact number of negative eigenvalues $-\lambda _{1,{\mathfrak {m}}}^2(\texttt{E}),...,-\lambda _{\kappa _0,{\mathfrak {m}}}^2(\texttt{E})<0$ for the operator

1.5 $$\begin{aligned} \mathcal {L}_{{\mathfrak {m}}} : = - \partial _{y}^2 + Q_{{\mathfrak {m}}}(y), \quad \text {with eigenfunctions } \quad \mathcal {L}_{{\mathfrak {m}}} \phi _{j,{\mathfrak {m}}} = -\lambda _{j,{\mathfrak {m}}}^2 \phi _{j,{\mathfrak {m}}}, \end{aligned}$$

where we imposed Dirichlet boundary conditions on $[- 1, 1]$. The rest of the spectrum $(\lambda _{j,{\mathfrak {m}}}^2(\texttt{E}))_{j\geqslant \kappa _{0}+1}$ is strictly positive. We remark that the eigenfunctions $(\phi _{j,{\mathfrak {m}}}(y)=\phi _{j,{\mathfrak {m}}}(\texttt{E};y))_{j\in {{\mathbb {N}}}}$, which form a $L^2$-orthonormal basis with respect to the standard $L^2$-scalar product, depend explicitly on the parameter $\texttt{E}$.

Shear flows, namely velocity fields of the form $(u,v)=(U(y),0)$ for some function *U*(*y*) depending only on $y\in [-1,1]$, are exact stationary solutions of (1.1) under the boundary conditions in (1.2). The next step is to introduce the shear flow $(\psi _{{\mathfrak {m}}}'(y),0)$ that plays the role of equilibrium point. In particular, we define the stream function $\psi _{{\mathfrak {m}}}(y)$ as the solution of the linear ODE

1.6 $$\begin{aligned} \psi _{{\mathfrak {m}}}'''(y) = Q_{{\mathfrak {m}}}(y) \psi _{{\mathfrak {m}}}'(y), \quad y\in [-1,1]. \end{aligned}$$

In Section 3 we will construct such stream function $\psi _{{\mathfrak {m}}}(y)$, even in *y* because of the parity of $Q_{{\mathfrak {m}}}(y)$, so that, for ${\mathfrak {m}}\gg 1$ large enough, the corresponding velocity field $(\psi _{{\mathfrak {m}}}'(y),0)$ is close to the well-known Couette flow (*y*, 0) with respect to the width $\texttt{r}$ in the $H^3$-topology. Roughly speaking, the function $\psi _{{\mathfrak {m}}}(y)$ solving (1.6) behaves almost linearly when $|y| > \texttt{r}$ and exhibits oscillations of frequency $\texttt{E}$ and amplitude $\texttt{E}^{-2}$ in the inner region $|y| < \texttt{r}$. Therefore, the shear flow $(\psi _{{\mathfrak {m}}}'(y),0)$ is *non-monotone*. In Lemma (3.5), we will show that $\psi _{{\mathfrak {m}}}(y)$ has exactly $2\kappa _{0}+1$ critical points, denoted by $\texttt{y}_{0,{\mathfrak {m}}}:=0$ and $(\pm \texttt{y}_{p,{\mathfrak {m}}})_{p=1,...,\kappa _{0}}$, with $0<\texttt{y}_{1,{\mathfrak {m}}}<...<\texttt{y}_{\kappa _0,{\mathfrak {m}}}<\texttt{r}$. These points lead to divide the interval $[-1,1]$ into the union of stripes $(\texttt{I}_{p})_{p=0,1,...,\kappa _{0}}$, where

1.7 $$\begin{aligned} \begin{aligned}&\texttt{I}_{p}:=\{ y \in {{\mathbb {R}}}: \texttt{y}_{p,{\mathfrak {m}}} \leqslant |y| \leqslant \texttt{y}_{p+1,{\mathfrak {m}}}\}, \quad p=1,...,\kappa _{0}, \\ \end{aligned} \end{aligned}$$

with $\texttt{y}_{\kappa _0+1,{\mathfrak {m}}}:=1$. We will also show in Theorem 3.7 that $\psi _{{\mathfrak {m}}}(y)$ solves on each set $\texttt{I}_{p}$ a second-order nonlinear ODE. Namely, we prove that there exist $\kappa _0+1$ functions $F_{0,{\mathfrak {m}}}(\psi ),F_{1,{\mathfrak {m}}}(\psi ),...,F_{\kappa _0,{\mathfrak {m}}}(\psi )$ such that, for any $y\in \texttt{I}_{p}$, $p=0,1,...,\kappa _{0}$,

1.8 $$\begin{aligned} Q_{{\mathfrak {m}}}(y) = F_{p,{\mathfrak {m}}}'(\psi _{{\mathfrak {m}}}(y)) \ \Rightarrow \ \psi _{{\mathfrak {m}}}''(y) = F_{p,{\mathfrak {m}}}(\psi _{{\mathfrak {m}}}(y)) , \end{aligned}$$

with continuity of finitely many derivatives at the boundaries of each set $\texttt{I}_{p}$ with the adjacent problems, meaning that, for a given $S\in {{\mathbb {N}}}$ large enough, for any $0\leqslant n \leqslant S+1$ and any stripe index $p=1,...,\kappa _0$,

1.9 $$\begin{aligned} \lim _{|y|\rightarrow \texttt{y}_{p,{\mathfrak {m}}}^-} \partial _{y}^{n} (F_{p-1,{\mathfrak {m}}}(\psi _{{\mathfrak {m}}}(y)) )= \lim _{|y|\rightarrow \texttt{y}_{p,{\mathfrak {m}}}^+}\partial _{y}^{n}( F_{p,{\mathfrak {m}}}(\psi _{{\mathfrak {m}}}(y)) ) = \psi _{{\mathfrak {m}}}^{(n+2)}(\texttt{y}_{p,{\mathfrak {m}}}). \nonumber \\ \end{aligned}$$

The regularity condition (1.9) is ensured by suitable properties of the $Q_{{\mathfrak {m}}}(y)$: we postponed this explanation to Section 1.2 “The shear equilibrium $\psi _{{\mathfrak {m}}}(y)$ close to Couette and its nonlinear ODE” and Section 3.1.

On the two-dimensional channel ${{\mathbb {R}}}\times [-1,1]$, we impose quasi-periodic condition in the *x*-direction, that is, the fluid evolves in the embedded domain

1.10 $$\begin{aligned} \begin{aligned}&\mathcal {D}:= {{\mathbb {T}}}^{\kappa _{0}}\times [-1,1] \hookrightarrow {{\mathbb {R}}}\times [-1,1] , \quad {{\mathbb {T}}}^{\kappa _0}:=({{\mathbb {R}}}/2\pi {{\mathbb {Z}}})^{\kappa _0}. \end{aligned} \end{aligned}$$

On the domain $\mathcal {D}$ we define the Laplacian $\Delta _{\omega }:=(\omega \cdot \partial _\textbf{x})^2+\partial _y^2$ for some frequency vector $\omega \in {{\mathbb {R}}}^{\kappa _{0}}$, where $\textbf{x}\in {{\mathbb {T}}}^{\kappa _0}$. It is well known that a subclass of solutions of the steady Euler equation (1.1) is given by those stream functions $\psi (x,y)$ that additionally solve semilinear elliptic equations of the form $\Delta \psi := (\partial _{x}^2+ \partial _{y}^2) \psi = F(\psi )$, for some function $F:{{\mathbb {R}}}\rightarrow {{\mathbb {R}}}$.

The goal of this paper is to construct solutions to the steady Euler equation (1.1)–(1.2) in the domain $\mathcal {D}$ close to the shear equilibrium $(\psi _{{\mathfrak {m}}}'(y),0)$. In particular, by (1.1) and (1.8), we look for stream functions quasi-periodic in *x* of the form

1.11 $$\begin{aligned} \psi (x,y) =\breve{\psi }(\textbf{x},y)_{\textbf{x}=\omega x}= \psi _{{\mathfrak {m}}}(y) + {\varphi }(\textbf{x},y)|_{\textbf{x}=\omega x}, \quad {\varphi }(\textbf{x}, -1) \!=\! {\varphi }(\textbf{x}, 1) = 0 , \nonumber \\ \end{aligned}$$

where $\psi _{{\mathfrak {m}}}(y)$ solves (1.8) and ${\varphi }(\textbf{x},y)$ is a solution of

1.12 $$\begin{aligned} \{\psi _{{\mathfrak {m}}} ,\Delta _{\omega } {\varphi }\} + \{{\varphi },\psi _{\mathfrak {m}}''\} + \{{\varphi },\Delta _{\omega }{\varphi }\} =0. \end{aligned}$$

By a direct computation, we have that a particular class of solutions of (1.12) is given by those functions ${\varphi }(\textbf{x}, y)$ solving, for any $p=0,1,...., \kappa _0$,

1.13 $$\begin{aligned} \Delta _{\omega } {\varphi }(\textbf{x},y) = F_{p,\eta }(\psi _{{\mathfrak {m}}}(y)+{\varphi }(\textbf{x},y)) - F_{p,{\mathfrak {m}}}(\psi _{{\mathfrak {m}}}(y)), \quad (\textbf{x},y)\in {{\mathbb {T}}}^{\kappa _0}\times \texttt{I}_{p}. \nonumber \\ \end{aligned}$$

The functions $(F_{p,\eta }(\psi ))_{p=0,1,...,\kappa _{0}}$ in (1.13) are regularized versions of the functions $(F_{p,{\mathfrak {m}}}(\psi ))_{p=0,1,...,\kappa _{0}}$, suitably defined for a small parameter $\eta >0$ as in (4.1) of the form

1.14 $$\begin{aligned} \begin{aligned} F_{p,\eta }(\psi ) = {\left\{ \begin{array}{ll} \frac{1}{2}\big (F_{p-1,{\mathfrak {m}}}(\psi ) + F_{p,{\mathfrak {m}}}(\psi ) \big ) = F_{p-1,\eta }(\psi )&  \quad | \psi - \psi _{{\mathfrak {m}}}(\texttt{y}_{p,{\mathfrak {m}}}) | \leqslant \eta , \\ F_{p,{\mathfrak {m}}}(\psi ) &  \quad \begin{aligned} &  | \psi - \psi _{{\mathfrak {m}}}(\texttt{y}_{p,{\mathfrak {m}}}) | \geqslant 2\eta \ \ \text {and}\\ &  | \psi - \psi _{{\mathfrak {m}}}(\texttt{y}_{p+1,{\mathfrak {m}}}) | \geqslant 2\eta , \end{aligned} \\ \frac{1}{2}\big (F_{p+1,{\mathfrak {m}}}(\psi ) + F_{p,{\mathfrak {m}}}(\psi ) \big ) = F_{p+1,\eta }(\psi ) &  \quad | \psi - \psi _{{\mathfrak {m}}}(\texttt{y}_{p+1,{\mathfrak {m}}}) | \leqslant \eta , \end{array}\right. } \end{aligned}\nonumber \\ \end{aligned}$$

with smooth connections in the remaining regions, so that they uniformly converge in the limit $\eta \rightarrow 0$ to the functions $F_{p,{\mathfrak {m}}}(\psi )$ in (1.8)–(1.9), see Proposition 4.1. Ultimately, in Section 4 we will choose $\eta =\varepsilon ^{\frac{1}{S}}$ as in (4.22), where $\varepsilon$ denotes the size of the perturbation ${\varphi }(\textbf{x},y)$ in (1.11) and where $S\in {{\mathbb {N}}}$ is the number of derivatives that we have to control in (1.9).

The linearization of the equation in (1.12) around the equilibrium ${\varphi }=0$ is given by

1.15 $$\begin{aligned} \{\psi _{{\mathfrak {m}}} ,\Delta _{\omega } {\varphi }\} + \{{\varphi },\psi _{\mathfrak {m}}''\} =0. \end{aligned}$$

By (1.5)–(1.6), a particular class of solutions of (1.15) on the domain $\mathcal {D}$ is given by

1.16 $$\begin{aligned} (\omega \cdot \partial _{\textbf{x}})^2{\varphi }(\textbf{x},y) = \mathcal {L}_{{\mathfrak {m}}}{\varphi }(\textbf{x},y), \quad {\varphi }(\textbf{x},-1)={\varphi }(\textbf{x},1) =0, \end{aligned}$$

where the self-adjoint Schrödinger operator $\mathcal {L}_{{\mathfrak {m}}}$, defined in (1.5) with Dirichlet boundary conditions on $[-1,1]$, is studied in Proposition 3.10,

The linearized equation in (1.15)–(1.16) around the trivial equilibrium ${\varphi }\equiv 0$ admits the family of space quasi-periodic solutions

1.17 $$\begin{aligned} \varphi (x, y) = \sum _{j=1}^{\kappa _0} A_j \cos (\lambda _{j,{\mathfrak {m}}}(\texttt{E}) x) \phi _{j,{\mathfrak {m}}}(y), \end{aligned}$$

for some nonzero coefficients $A_j\in {{\mathbb {R}}}\setminus \{0\}$ with frequency vector

1.18 $$\begin{aligned} \omega \equiv \vec {\omega }_{{\mathfrak {m}}}(\texttt{E}):=(\lambda _{1,{\mathfrak {m}}}(\texttt{E}),...,\lambda _{\kappa _0,{\mathfrak {m}}}(\texttt{E}))\in {{\mathbb {R}}}^{\kappa _0}\setminus \{0\}. \end{aligned}$$

The analysis of the whole linearized systems at the equilibrium and the geometry of the “spatial” phase space is postponed to Section 4. We will also prove in Proposition 5.7 that, for most values of $\texttt{E}\in [\texttt{E}_1,\texttt{E}_2]$, with $\texttt{E}_1>(\kappa _{0}+\tfrac{1}{4})\pi$, the vector $\vec {\omega }_{{\mathfrak {m}}}(\texttt{E})$ in (1.18) is Diophantine: namely, given ${\overline{\upsilon }}\in (0,1)$ and ${\overline{\tau }} \gg 1$ sufficiently large, there exists a Borel set

1.19 $$\begin{aligned} {\overline{\mathcal {K}}} = {\overline{\mathcal {K}}}({\overline{\upsilon }},{\overline{\tau }}) := \big \{ \texttt{E}\in [\texttt{E}_1,\texttt{E}_2] : |\vec {\omega }_{{\texttt{m}}}({\texttt{E}}) \cdot \ell | \geqslant {\overline{\upsilon }} \mathinner {\langle {\ell }\rangle }^{-{\overline{\tau }}}, \ \forall \ell \in {{\mathbb {Z}}}^{\kappa _0} \setminus \{ 0 \} \big \}, \nonumber \\ \end{aligned}$$

such that $\texttt{E}_2-\texttt{E}_2 - |{\overline{\mathcal {K}}}| = o({\overline{\upsilon }})$. This ensures that the linear solutions in (1.17) are quasi-periodic with non-resonant frequency vectors.

Equation (1.12) enjoys some symmetries. Since the shear equilibrium $\psi _{{\mathfrak {m}}}(y)$ is even in $y\in [-1,1]$ and so are the eigenfunctions of the linear operator $\mathcal {L}_{{\mathfrak {m}}}$ in Proposition 3.10, we have that (1.12) is invariant with respect to the involution ${\varphi }(\cdot ,y)\mapsto {\varphi }(\cdot ,-y)$. Moreover, Equation (1.12) is also invariant with respect to the involution ${\varphi }(\textbf{x},y)\mapsto {\varphi }(-\textbf{x},-y)$: we refer to such solutions as *space reversible*, or simply *reversible*. We conclude that we look for solutions

1.20 $$\begin{aligned} {\varphi }(\textbf{x},y)\in \textrm{even}(\textbf{x})\textrm{even}(y). \end{aligned}$$

The function ${\varphi }(\textbf{x},y)$ is searched in the Sobolev space $H^{s,3}$, as defined in (2.1).

The main result of this paper is the existence of a stream function of the form (1.11), where the functions ${\varphi }(\textbf{x},y)$ are small amplitude, reversible *space quasi-periodic* solutions of the system (1.12) with frequency vector $\omega \in {{\mathbb {R}}}^{\kappa _{0}}$, bifurcating from a solution (1.17) of the linearization around the trivial equilibria. Such solutions are constructed for a * fixed valued of the depth* $\texttt{E}\in {\overline{\mathcal {K}}}$ in (1.19) and *for most values of an auxiliary parameter*

1.21 $$\begin{aligned} {\texttt{A}} \in \mathcal {J}_\varepsilon ({\texttt{E}}) := [\texttt{E}-\sqrt{\varepsilon },\texttt{E}+\sqrt{\varepsilon }]. \end{aligned}$$

This new parameter is introduced to ensure that frequency vectors $\omega \in {{\mathbb {R}}}^{\kappa _0}$, close to the unperturbed frequency vector $\vec {\omega }_{{\mathfrak {m}}}(\texttt{E})$ in (1.18), is non-resonant as well.

#### Theorem 1.1

(Spatial KAM for 2D Euler equations in a channel) Fix $\kappa _0\in {{\mathbb {N}}}$ and ${\mathfrak {m}}\gg 1$. Fix also $\texttt{E}\in {\overline{\mathcal {K}}}$ as in (1.19) and $\xi = (\xi _1, \ldots , \xi _{\kappa _0}) \in {{\mathbb {R}}}_{>0}^{\kappa _0}$. Then there exist ${\overline{s}}>0$, $\varepsilon _0>0$ such that the following hold:

(1) For any $\varepsilon \in (0, \varepsilon _0)$ there exists a Borel set $\mathcal {G}_{\varepsilon }=\mathcal {G}_{\varepsilon }(\texttt{E})\subset \mathcal {J}_{\varepsilon }(\texttt{E})$, with$\mathcal {J}_{\varepsilon }(\texttt{E})$ as in (1.21) and with density 1 at $\texttt{E}$ when $\varepsilon \rightarrow 0$, namely $\lim _{\varepsilon \rightarrow 0} (2\sqrt{\varepsilon })^{-1}|\mathcal {G}_{\varepsilon }(\texttt{E})| =1$;

(2) There exists $h_\varepsilon =h_{\varepsilon }(\texttt{E}) \in H^3_0([- 1, 1])$, $\Vert h_\varepsilon \Vert _{H^3} \lesssim \varepsilon$, $h_\varepsilon = \textrm{even}(y)$, such that, for any $\texttt{A}\in \mathcal {G}_{\varepsilon }$, Equation (1.12) has a space quasi-periodic solution of the form

1.22 $$\begin{aligned} {\varphi }_{\varepsilon }(\textbf{x},y)|_{\textbf{x}={\widetilde{\omega }}(\texttt{A}) x}= &   h_{\varepsilon }(\texttt{E};y) + \varepsilon \sum _{j=1}^{\kappa _0}\sqrt{\xi _{j}} \cos ({\widetilde{\omega }}_{j}(\texttt{A})x) \phi _{j,{\mathfrak {m}}}(\texttt{E};y) \nonumber \\    &   + r_\varepsilon (\textbf{x},y)|_{\textbf{x}={\widetilde{\omega }}(\texttt{A}) x}, \end{aligned}$$

where ${\varphi }_{\varepsilon }={\varphi }_{\varepsilon }(\texttt{E},\texttt{A};\textbf{x},y) = \textrm{even}(\textbf{x}) \textrm{even}(y)$, $r_{\varepsilon }=r_{\varepsilon }(\texttt{E},\texttt{A};\textbf{x},y)\in H^{{\overline{s}},3}$ (see Definition (2.1)), with $\lim _{\varepsilon \rightarrow 0} \frac{\Vert r_\varepsilon \Vert _{{\overline{s}},3}}{{\varepsilon }}=0$, and ${\widetilde{\omega }}=({\widetilde{\omega }}_{j})_{j=1,...,\kappa _0}\in {{\mathbb {R}}}^{\kappa _0}$, depending on $\texttt{A}$ and $\varepsilon$, with $| {\widetilde{\omega }}(\texttt{A})-\vec {\omega }_{{\mathfrak {m}}}(\texttt{E}) | \leqslant C \sqrt{\varepsilon }$, with $C>0$ independent of $\texttt{E}$ and $\texttt{A}$. Moreover for any $\varepsilon \in [0, \varepsilon _0]$, the stream function

1.23 $$\begin{aligned} \psi _{\varepsilon }(x,y) =\breve{\psi }_{\varepsilon }(\textbf{x},y)|_{\textbf{x}={\widetilde{\omega }}(\texttt{A})x} = \psi _{{\mathfrak {m}}}(y) + {\varphi }_{\varepsilon }(\textbf{x},y)|_{\textbf{x}={\widetilde{\omega }}(\texttt{A})x}, \end{aligned}$$

with ${\varphi }_{\varepsilon }(\textbf{x},y)$ as in (1.22), defines a space quasi-periodic solution of the steady 2D Euler equation (1.1) that is close to the Couette flow with estimates

1.24 $$\begin{aligned} \Vert \breve{\psi }_{\varepsilon } - \psi _{\textrm{cou}} \Vert _{{\overline{s}} , 3} \lesssim _{{\overline{s}}} \tfrac{1}{\sqrt{\texttt{E}}} + \varepsilon , \quad \psi _{\textrm{cou}}(y):= \tfrac{1}{2} y^2. \end{aligned}$$

Let us make some remarks on the result.

(1) *Structure of the stationary solutions.* The stream functions (1.22)–(1.23) in Theorem 1.1 are slight perturbations of the shear equilibrium $\psi _{{\mathfrak {m}}}(y)$. The first term of ${\varphi }_{\varepsilon }(\textbf{x},y)$ in (1.23) is the shear $h_{\varepsilon }(y)$ and it comes from the forced modification in (1.12) of the local nonlinearities $F_{p,{\mathfrak {m}}}(\psi )$ into $F_{p,\eta }(\psi )$. By Lemma 4.3 and (4.22), it is small with $\eta ^S=\varepsilon$ and therefore vanishes in the limit $\varepsilon \rightarrow 0$. The second term of ${\varphi }_{\varepsilon }(\textbf{x},y)$ in (1.23) retains the space quasi-periodicity of the linearized solutions (1.17) with frequency vectors ${\widetilde{\omega }}$ that are close to the unperturbed frequency vector $\vec {\omega }_{{\mathfrak {m}}}(\texttt{E})$ in (1.18). This term is constructed with a suitable Nash–Moser iterative scheme in order to deal with the eigenfunctions $(\phi _{j,{\mathfrak {m}}}(\texttt{E};y))_{j\in {{\mathbb {N}}}}$ depending on the parameter $\texttt{E}$, which is an issue not present in previous papers. Such solutions exist for fixed values of the depth $\texttt{E}\in {\overline{\mathcal {K}}}$ so that $\vec {\omega }_{{\mathfrak {m}}}(\texttt{E})$ is Diophantine and for most values of the auxiliary parameter $\texttt{A}\in \mathcal {J}_{\varepsilon }(\texttt{E})$ so that ${\widetilde{\omega }}={\widetilde{\omega }}(\texttt{A},\varepsilon )$ is non-resonant as well. We refer to Section 1.2 “A Nash–Moser scheme of hypothetical conjugation with the auxiliary parameter” for an extensive discussion.

(2) *From quasi-periodic stationary to quasi-periodic traveling.* By changing the frame reference $x \mapsto x -ct$ with an arbitrary speed $c\in {{\mathbb {R}}}$, we deduce the existence of *quasi-periodic traveling* solutions, according to [8], of the form

$$\begin{aligned} \begin{aligned} \psi _{\textrm{tr}}(t,x,y)&:= c y + \psi _{\varepsilon }(x-ct,y) \\&\ = c y + \psi _{{\mathfrak {m}}}(y) + {\varphi }_{\varepsilon }(\phi ,y)|_{\phi =\textbf{x}-\vartheta ={\widetilde{\omega }}(x-ct)}, \quad \textbf{x},\vartheta \in {{\mathbb {T}}}^{\kappa _0}, \end{aligned} \end{aligned}$$

solving the Euler equations in vorticity formulation

$$\begin{aligned} (\Omega _{\textrm{tr}})_t+(\psi _{\textrm{tr}})_y (\Omega _{\textrm{tr}})_x - ( \psi _{\textrm{tr}})_x (\Omega _{\textrm{tr}})_y =0, \quad \Omega _{\textrm{tr}}:= \Delta \psi _{\textrm{tr}}. \end{aligned}$$

We read these solutions also as quasi-periodic in time with time frequency vector $c{\widetilde{\omega }}$ parallel to the space frequency vector ${\widetilde{\omega }}$. It is of great interest to see whether there exist quasi-periodic solutions to the Euler equations both in time and in space, but with non-collinear frequency vectors.

(3) *Generalized Kelvin cat’s eyes, lack of damping and regularity thresholds.* The flow generated by the stream function (1.22)–(1.23) is a deformation of the near-Couette shear flow $(\psi _{{\mathfrak {m}}}'(y),0)$ of the form

1.25 $$\begin{aligned} \begin{pmatrix} u(x,y)\\ v(x,y) \end{pmatrix}= \begin{pmatrix} \psi _{{\mathfrak {m}}}'(y) + h_{\varepsilon }'(y)\\ 0 \end{pmatrix} + \varepsilon \sum _{j=1}^{\kappa _0} \sqrt{\xi _j} \begin{pmatrix} \cos ({\widetilde{\omega }}_j x) \phi _{j,{\mathfrak {m}}}'(y) \\ {\widetilde{\omega }}_j \sin ({\widetilde{\omega }}_j x) \phi _{j,{\mathfrak {m}}}(y) \end{pmatrix} + o(\varepsilon ). \nonumber \\ \end{aligned}$$

We first observe that, since $\psi _{{\mathfrak {m}}}(y)$, $h_{\varepsilon }(y)$ and the eigenfunctions $\phi _{j,{\mathfrak {m}}}(y)$ are even in *y*, the streamlines of the perturbed flows have a generalized cat’s eyes structure near the stagnation line $\{y=0\}$ of the shear flow $(\psi _{{\mathfrak {m}}}'(y)+h_{\varepsilon }'(y),0)$, with saddle and center points near the roots of the trigonometric equation $\sum _{j=1}^{\kappa _0}\sqrt{\xi _j}{\widetilde{\omega }}_j \phi _{j,{\mathfrak {m}}}(0)\sin ({\widetilde{\omega }}_j x) =0$. Possible other cat’s eyes-like streamlines may appear near the lines $\{ y = \pm \texttt{y}_{p,{\mathfrak {m}}}\}$, $p=1,...,\kappa _0$, corresponding to the critical points for $\psi _{{\mathfrak {m}}}(y)$, depending on further properties of the eigenfunctions $(\phi _{j,{\mathfrak {m}}}(y))_{j=1,...,\kappa _0}$ that we do not investigate in this paper.

We also observe that, no matter the geometry of the streamlines, the velocity field (1.25) has non-trivial vertical component that is quasi-periodic in $x\in {{\mathbb {R}}}$. The presence of such quasi-periodic stationary solutions prevents damping phenomena in the evolution of the dynamics for the Euler equations with quasi-periodic conditions in *x*. Our result agrees with the analysis made for the periodic case in [38] and their (vorticity) regularity threshold $s<\frac{3}{2}$. Indeed in Theorem 3.4 we show that $\psi _{{\mathfrak {m}}}(y)$ is close to $\psi _{\textrm{cou}}(y)$ with $\sqrt{\texttt{r}}$ in the $H_y^3$-topology. At the same time, arguing as in the proof of Theorem 3.4 with an easy computation that we omit here, it is possible to show that the bound for the $L^2$-norm of $\psi _{{\mathfrak {m}}}^{(4)}(y)$ diverges with $\texttt{r}^{-1/2}$. Therefore, a standard interpolation argument shows that we can construct $\psi _{{\mathfrak {m}}}(y)$ arbitrarily close to $\psi _{\textrm{cou}}(y)$ in the $H_{y}^\rho$-topology, with the (stream) regularity $\rho < \frac{7}{2}$. We remark that here only the regularity for the estimate (1.24) in the vertical direction *y* is below such threshold, whereas the Sobolev regularity in the horizontal direction $\textbf{x}= {\widetilde{\omega }}x$ has to be sufficiently large to compensate, during the Nash–Moser iteration, the loss of derivatives coming from the small divisors and the Diophantine conditions, see (2.10).

### Strategy of the Proof

We look for stationary solutions of the Euler equation in vorticity-stream function formulation (1.1) as solutions of semilinear elliptic PDEs (1.13). The quasi-periodic solutions in *x* of Theorem 1.1 are then searched via a Nash–Moser implicit function theorem on such elliptic equations, with initial guess given by the solutions (1.17) of the linearized Euler equations at the shear equilibrium (1.15). The main difficulties and novelties of our results can be summarized as follows:

- Each space quasi-periodic function ${\varphi }_{\varepsilon }(\textbf{x},y)$ solve the nonlinear PDE (1.13) with nonlinearities explicitly depending of the size $\varepsilon$ of the solution;
- The nonlinearity of the semilinear elliptic problem that we solve is actually an "unknown" of the problem and it has to be constructed in such a way that one has a near Couette, space quasi-periodic solution to the Euler equation (1.12);
- The nonlinearities have finite smoothness and their derivatives lose in size;
- The unperturbed frequencies of oscillations are only implicitly defined and their non-degeneracy property relies on an asymptotic expansion for large values of the parameter. It implies that the required non-resonance conditions are not trivial to verify;
- The basis of eigenfunctions $(\phi _{j,{\mathfrak {m}}}(y))_{j\in {{\mathbb {N}}}}$ of the operator $\mathcal {L}_{{\mathfrak {m}}}$ in (1.5) is not the standard exponential basis and depends explicitly on the parameter $\texttt{E}$.

We now illustrate the main steps to prove Theorem 1.1 and how we will overcome the main difficulties.

**The shear equilibrium** $\psi _{{\mathfrak {m}}}(y)$ **close to Couette and its nonlinear ODE.** The first issue that we need to solve is to determine which nonlinear differential equation is satisfied by $\psi _{{\mathfrak {m}}}(y)$ and the regularity properties of the nonlinearity. Our starting point is the linear ODE

1.26 $$\begin{aligned} \psi _{{\mathfrak {m}}}'''(y)= Q_{{\mathfrak {m}}}(y)\psi _{{\mathfrak {m}}}'(y), \end{aligned}$$

where $Q_{{\mathfrak {m}}}(y)$ is a prescribed analytic potential of the form (see also (3.1))

1.27 $$\begin{aligned} \begin{aligned} Q_{{\mathfrak {m}}}(y)= Q_{{\mathfrak {m}}}(\texttt{E},\texttt{r};y)&:= -\texttt{E}^2\Big ( \Big ( \Big (\frac{\cosh (\frac{y}{\texttt{r}})}{\cosh (1)}\Big )^{\mathfrak {m}}+1 \Big )^{-1}+g_{{\mathfrak {m}},S}\big (\tfrac{y}{\texttt{r}}\big ) \Big ), \end{aligned} \end{aligned}$$

that approaches the singular finite well potential $Q_{\infty }(y):= -\texttt{E}^2\chi _{(-1,1)}(\frac{y}{\texttt{r}})$ in (1.3) with estimates as in Lemma 3.2. There are some degrees of freedom in the choice of the potential $Q_{{\mathfrak {m}}}(y)$ that we will take advantage of in the construction of our solutions.

First, the choice of the parameters $\texttt{E}>(\kappa _{0}+\frac{1}{4})\pi$ and $\texttt{r}\in (0,1)$ controls both the numbers of negative eigenvalues of the Schrödinger operator $\mathcal {L}_{{\mathfrak {m}}}:= -\partial _y^2 + Q_{{\mathfrak {m}}}(y)$, via the constrain $\texttt{E}\texttt{r}= (\kappa _0+\tfrac{1}{4})\pi$ in (1.4), and their non-resonance properties. These $\kappa _0$ negative eigenvalues determine the frequencies of oscillations in the horizontal direction for the solutions of the linearized system at the equilibrium, see (1.17)–(1.18). The non-degeneracy of the curve $\texttt{E}\mapsto \vec {\omega }_{{\mathfrak {m}}}(\texttt{E})$, which is needed to ensure Diophantine non-resonance conditions on the frequency vector $\vec {\omega }_{{\mathfrak {m}}}(\texttt{E})$ and on its perturbations, is proved in Section 5. We remark that an extra difficulty is due to the fact that these $\kappa _0$ linear frequencies are proved initially to be close to the $\kappa _0$ real roots of a transcendental equation, see (3.44) in Theorem 3.10. This issue is overcome by proving asymptotic expansions of the latter roots, see Lemma (5.3), and then by a perturbative argument.

Back to the second order ODE (1.26) for $\psi _{{\mathfrak {m}}}'(y)$, its odd solutions, roughly speaking, behave as the affine Couette shear flow in the outer region $|y|> \texttt{r}$ and as oscillations of frequency $\texttt{E}$ and amplitude $\texttt{E}^{-2}$ in the inner region $|y|<\texttt{r}$. In particular, the shear flow $(\psi _{{\mathfrak {m}}}'(y),0)$ has $2\kappa _0+1$ stagnation lines, including the axis $\{y=0\}$ and with the remaining ones symmetric with respect to it. These stagnation lines will be the boundaries of the stripes ${{\mathbb {R}}}\times \texttt{I}_{p}$ in (1.7), (1.10), where $(\pm \texttt{y}_{p,{\mathfrak {m}}})_{p=0,1,...,\kappa _0}$ denote the critical points of $\psi _{{\mathfrak {m}}}(y)$.

The second-order nonlinear ODE satisfied by $\psi _{{\mathfrak {m}}}(y)$ is determined via the Cauchy problem solved with a nonlinear vector field $F(\psi )$ induced by $Q_{{\mathfrak {m}}}(y)$, see (1.8), and with initial datum $F(\psi _{{\mathfrak {m}}}(0)) = \psi _{{\mathfrak {m}}}''(0)$. Because $\psi _{{\mathfrak {m}}}'(y)$ is not monotone, the nonlinearity is constructed locally on each domain where $\psi _{{\mathfrak {m}}}$ is invertible. Therefore, morally speaking, the nonlinearity $F(\psi )$ globally behaves as a multi-valued function defined on the range domain $\psi _{{\mathfrak {m}}}([-1,1])$. We now sketchily describe the general idea of the construction of the nonlinearity starting around $\psi _{{\mathfrak {m}}}(0)$. Since $\psi _{{\mathfrak {m}}}'(y)$ odd, we can locally solve the initial value problem

1.28 $$\begin{aligned} {\left\{ \begin{array}{ll} F_{0,{\mathfrak {m}}}' (\psi ) = Q_{{\mathfrak {m}}} (\psi _{{\mathfrak {m}}}^{-1}(\psi )), \quad \psi \in \psi _{{\mathfrak {m}}}([0,T]), \\ F_{0,{\mathfrak {m}}}(\psi _{{\mathfrak {m}}}(0)) = \psi _{{\mathfrak {m}}}''(0). \end{array}\right. } \end{aligned}$$

The local solution of (1.28) extends until it meets the next critical point of $\psi _{{\mathfrak {m}}}(y)$, namely on $\psi _{{\mathfrak {m}}}([0,\texttt{y}_{1,{\mathfrak {m}}}))$, recalling that $\psi _{{\mathfrak {m}}}'(\texttt{y}_{1,{\mathfrak {m}}})=0$. To pass over this critical point, we define a new Cauchy problem

$$\begin{aligned} {\left\{ \begin{array}{ll} F_{1,{\mathfrak {m}}}' (\psi ) = Q_{{\mathfrak {m}}} (\psi _{{\mathfrak {m}}}^{-1}(\psi )), \quad \psi \in \psi _{{\mathfrak {m}}}([\texttt{y}_{1,{\mathfrak {m}}},T]), \\ F_{1,{\mathfrak {m}}}(\psi _{{\mathfrak {m}}}(\texttt{y}_{1,{\mathfrak {m}}})) = F_{0,{\mathfrak {m}}}(\psi _{{\mathfrak {m}}}(\texttt{y}_{1,{\mathfrak {m}}})). \end{array}\right. } \end{aligned}$$

By (1.26), we deduce $\psi _{{\mathfrak {m}}}'''(\texttt{y}_{1,{\mathfrak {m}}})=0$. By this latter identity, it is possible to show that $F_{0,{\mathfrak {m}}}(\psi _{{\mathfrak {m}}}(y))$ and $F_{1,{\mathfrak {m}}}(\psi _{{\mathfrak {m}}}(y))$ agree at $y=\texttt{y}_{1,{\mathfrak {m}}}$ with $\mathcal {C}^1$-continuity. For the purposes of the Nash–Moser nonlinear iteration, $\mathcal {C}^1$-regularity for the nonlinearity is definitely not enough to deal with the loss of derivatives coming from the small divisors. It is at this point that we use the extra degrees of freedom coming from the potential $Q_{{\mathfrak {m}}}(y)$: indeed, in (1.27) we can choose the corrector $g_{{\mathfrak {m}},S}$ to impose arbitrarily finitely many vanishing conditions on the odd derivatives of $Q_{{\mathfrak {m}}}(y)$ at $y=\texttt{y}_{1,{\mathfrak {m}}}$, and consequently on $\psi _{{\mathfrak {m}}}(y)$ by (1.26), which we use to ensure $\mathcal {C}^S$-regularity of the nonlinearities $F_{1,{\mathfrak {m}}}$ and $F_{0,{\mathfrak {m}}}$ at $\psi =\psi _{{\mathfrak {m}}}(y)$ for an arbitrarily fixed and large $S\in {{\mathbb {N}}}$. The main idea here is that the regularity is determined by the "local evenness" of $Q_{{\mathfrak {m}}}(y)$ and $\psi _{{\mathfrak {m}}}(y)$ around the critical point $\texttt{y}_{1,{\mathfrak {m}}}$, meaning that we can write

$$\begin{aligned} Q_{{\mathfrak {m}}}(\texttt{y}_{1,{\mathfrak {m}}}+\delta ) = \sum _{n=0}^{S} \frac{Q_{{\mathfrak {m}}}^{(2n)}(\texttt{y}_{1,{\mathfrak {m}}})}{(2n)!} \delta ^{2n} +\frac{Q_{{\mathfrak {m}}}^{(2S+1)}(\texttt{y}_{1,{\mathfrak {m}}})}{(2S+1)!} \delta ^{2S+1} + o(|\delta |^{2(S+1)}), \end{aligned}$$

and similarly for $\psi _{{\mathfrak {m}}}(y)$, see Lemma 3.6, so that we can invert $\psi _{{\mathfrak {m}}}$ as a function of $\delta ^2$ with inverse of finite regularity. The corrector $g_{{\mathfrak {m}},S}(z)$ will be a polynomial functions, therefore analytic, that we use to control the local behaviour of $Q_{{\mathfrak {m}}}(y)$ also at the other critical points, without affecting the global shape of the potential.

This construction is then iterated when we reach the remaining finitely many critical points $\texttt{y}_{2,{\mathfrak {m}}}<...<\texttt{y}_{\kappa _0,{\mathfrak {m}}}<\texttt{y}_{\kappa _0+1,{\mathfrak {m}}}:=1$. All the technical details are provided in Section 3: in particular, in Theorem 3.7 we show that there exist $\mathcal {C}^{S+1}({{\mathbb {R}}})$ functions $F_{0,{\mathfrak {m}}}(\psi ),...,F_{\kappa _0,{\mathfrak {m}}}(\psi )$ such that

1.29 $$\begin{aligned} \psi _{{\mathfrak {m}}}''(y) = F_{p,{\mathfrak {m}}}(\psi _{{\mathfrak {m}}}(y)) \quad \forall y \in \texttt{I}_{p}, \quad p=0,1,...,\kappa _{0}, \end{aligned}$$

and with $\mathcal {C}^{S+1}$-continuity at $\psi =\psi _{{\mathfrak {m}}}(y)$ as in (1.9).

**A forced elliptic PDE for the perturbation of the shear equilibrium.** Now that we have a good description of the shear equilibrium $\psi _{{\mathfrak {m}}}(y)$, we look for solutions depending on $x\in {{\mathbb {R}}}$ and ask what problems are solved by stream functions of the form $\psi (x,y)=\breve{\psi }(\textbf{x},y)|_{\textbf{x}=\omega x}=\psi _{{\mathfrak {m}}}(y)+{\varphi }(\textbf{x},y)_{\textbf{x}=\omega x}$. The first naïve attempt would be to look for solutions of the nonlinear PDE

$$\begin{aligned} \Delta _{\omega } \breve{\psi } ( \textbf{x},y) = F_{p,{\mathfrak {m}}} (\breve{\psi } (\textbf{x}, y)) , \quad (\textbf{x},y) \in {{\mathbb {T}}}^{\kappa _{0}}\times \texttt{I}_{p}, \quad p=0,1,...,\kappa _{0} \end{aligned}$$

with the same nonlinearities $F_{p,{\mathfrak {m}}}(\psi )$ as in (1.29) and insert the ansatz for $\breve{\psi }(\textbf{x},y)$. The equations for the perturbation ${\varphi }(\textbf{x},y)$ would be, for $(\textbf{x},y)\in {{\mathbb {T}}}^{\kappa _{0}} \times \texttt{I}_{p}$, $p=0,1,...,\kappa _0$,

$$\begin{aligned} (\omega \cdot \partial _{\textbf{x}})^2{\varphi }- \mathcal {L}_{{\mathfrak {m}}}{\varphi }- \big ( F_{p,{\mathfrak {m}}}(\psi _{{\mathfrak {m}}}(y)+{\varphi }) - F_{p,{\mathfrak {m}}}(\psi _{{\mathfrak {m}}}(y)) \big ) = 0, \end{aligned}$$

with $\mathcal {L}_{{\mathfrak {m}}}$ as in (1.5). This approach fails immediately because the continuity at $\pm \texttt{y}_{p,{\mathfrak {m}}}$ for $F_{p-1,{\mathfrak {m}}}(\psi _{{\mathfrak {m}}}(y)+{\varphi }(\textbf{x},y))$ and $F_{p,{\mathfrak {m}}}(\psi _{{\mathfrak {m}}}(y)+{\varphi }(\textbf{x},y))$ already does not hold any more in general (unless we require ${\varphi }(\textbf{x},\pm \texttt{y}_{p,{\mathfrak {m}}})=0$ for any $\textbf{x}\in {{\mathbb {T}}}^{\kappa _{0}}$, which is a too strong conditions, not even satisfied by the eigenfunctions of $\mathcal {L}_{{\mathfrak {m}}}$). Recalling that our ultimate goal is to solve the Euler equation (1.1), the idea is to slightly change the nonlinear functions $F_{p,{\mathfrak {m}}}(\psi )$ and “make enough room” in neighbourhoods of the critical values $\psi _{{\mathfrak {m}}}(\texttt{y}_{p,{\mathfrak {m}}})$ to ensure enough smoothness of the new nonlinearities when $\psi (x,y)$ is evaluated close to the stagnation lines $\{y=\pm \texttt{y}_{p,{\mathfrak {m}}}\}$. In particular, for a small parameter $\eta \ll 1$, we will use the regularized nonlinearity $F_{p,\eta }(\psi )$ as in (1.14) instead of $F_{p,{\mathfrak {m}}}(\psi )$. With this (non-unique) choice of the modified nonlinearities, we have that $F_{p,\eta }(\psi ) = F_{p-1,\eta }(\psi )$ when $\psi$ belongs to the open neighbourhood $B_{\eta }(\psi _{{\mathfrak {m}}}(\texttt{y}_{p,{\mathfrak {m}}}))$, $p=1,...,\kappa _0$. The finite smooth continuity at the stagnation line $\{y=\pm \texttt{y}_{p,{\mathfrak {m}}}\}$ between $F_{p-1,\eta }(\psi _{{\mathfrak {m}}}(y)+{\varphi }(\textbf{x},y))$ and $F_{p,\eta }(\psi _{{\mathfrak {m}}}(y)+{\varphi }(\textbf{x},y))$ is then easily satisfied, as soon as the perturbation ${\varphi }(\textbf{x},y)$ is small enough.

The new question that arises now is to estimate how close the two nonlinearities $F_{p,{\mathfrak {m}}}(\psi )$ and $F_{p,\eta }(\eta )$ (together with their derivatives) are with respect to the small parameter $\eta \ll 1$. Generally speaking, one only gets uniformly convergence in the limit $\eta \rightarrow 0$ and the derivatives of $F_{p,\eta }(\psi )$ exploding when $\eta \rightarrow 0$, due to presence of shrinking cut-off functions. The good news here is that, thanks to the "local evenness" that we were able to impose earlier on $Q_{{\mathfrak {m}}}(y)$ and $\psi _{{\mathfrak {m}}}(y)$ at the critical points $\pm \texttt{y}_{p,{\mathfrak {m}}}$, we can prove estimates (see Proposition 4.1), for $n=0,1,...,S+1$,

1.30 $$\begin{aligned} \sup _{y\in [-1,1]}\sup _{p=0,1,...,\kappa _{0}}| F_{p,\eta }^{(n)}(\psi _{{\mathfrak {m}}}(y)) - F_{p,{\mathfrak {m}}}^{(n)}(\psi _{{\mathfrak {m}}}(y)) | \lesssim _{n} \eta ^{S+\frac{3}{2} -n}. \end{aligned}$$

We finally conclude that the equation for the perturbation ${\varphi }(\textbf{x},y)$ that we are going to solve is (1.12), which implies, by (1.8), that $\Delta (\psi _{{\mathfrak {m}}}(y)+{\varphi }(\textbf{x},y))=F_{p,{\mathfrak {m}}}(\psi _{{\mathfrak {m}}}(y)+{\varphi }(\textbf{x},y))$ and that $\psi _{{\mathfrak {m}}}(y)+{\varphi }(\textbf{x},y)$ is a solutions of Euler equation (1.1). We point out that, by expanding

$$\begin{aligned} \begin{aligned}&F_{p,\eta }(\psi _{{\mathfrak {m}}}(y)+{\varphi }({\textbf {x}},y)) - F_{p,{\mathfrak {m}}}(\psi _{{\mathfrak {m}}}(y)) - F_{p,{\mathfrak {m}}}'(\psi _{{\mathfrak {m}}}(y)){\varphi }({\textbf {x}},y)\\&\quad = F_{p,\eta }(\psi _{{\mathfrak {m}}}(y)) - F_{p,{\mathfrak {m}}}(\psi _{{\mathfrak {m}}}(y))\\&\qquad +\big ( F_{p,\eta }'(\psi _{{\mathfrak {m}}}(y)) - F_{p,{\mathfrak {m}}}'(\psi _{{\mathfrak {m}}}(y))\big ){\varphi }({\textbf {x}},y) + \tfrac{1}{2} F_{p,\eta }''(\psi _{{\mathfrak {m}}}(y)) {\varphi }^2({\textbf {x}},y) + ... \end{aligned} \end{aligned}$$

the equation for ${\varphi }$ in (1.12) contains the forcing term $F_{p,\eta }(\psi _{{\mathfrak {m}}}(y)) - F_{p,{\mathfrak {m}}}(\psi _{{\mathfrak {m}}}(y))$ and a correction at the linear level $\big ( F_{p,\eta }'(\psi _{{\mathfrak {m}}}(y)) - F_{p,{\mathfrak {m}}}'(\psi _{{\mathfrak {m}}}(y))\big ){\varphi }({\textbf {x}},y)$. By (1.30), both of them are actually arbitrarily small with respect to (powers of) $\eta$, therefore they will be treated as perturbative terms in the Nash–Moser nonlinear iteration. In particular, by a small shifting of the unknown ${\varphi }(\textbf{x},y)$ with the $\textbf{x}$-independent function $h_{\eta }(y)$ in Lemma 4.3, it is possible to remove the forcing term and include its contribution directly into the nonlinearity.

**A Nash–Moser scheme of hypothetical conjugation with the auxiliary parameter.** Finally, the construction of the quasi-periodic solutions in spatial variable *x*, treated here as a temporal one, follows in the same approach of other KAM papers in fluid dynamics, see for instance [2, 8, 9, 12]. The main points are the splitting of the phase space (here "spatial phase space") into tangential and normal invariant subspaces, the introduction of action-angle coordinates on the tangential subspace and the definition of the nonlinear functional to implement the Nash–Moser iteration. The solutions are searched as embeddings $i:{{\mathbb {T}}}^{\kappa _{0}}\rightarrow {{\mathbb {T}}}^{\kappa _{0}}\times {{\mathbb {R}}}^{\kappa _{0}}\times \mathcal {X}_{\perp }^s$ in the phase space of the form $\textbf{x}\mapsto i(\textbf{x}) = (\theta (\textbf{x}),I(\textbf{x}),z(\textbf{x}))$, where $\mathcal {X}_{\perp }^{\perp }$ is the restriction of the functional space $H^{s,3}\times H^{s,1}$ to the normal subspace. The embedding is searched as the zero of the nonlinear functional

1.31 $$\begin{aligned} \textbf{F}(i) := \omega \cdot \partial _{\textbf{x}}i(\textbf{x}) - X_{\mathcal {H}_{\varepsilon }}(i(\textbf{x})), \end{aligned}$$

where $\mathcal {H}_{\varepsilon }$ is the Hamiltonian in action-angle coordinate (see also (4.38))

1.32 $$\begin{aligned} \mathcal {H}_{\varepsilon }(\theta ,I,z) = \vec {\omega }_{{\mathfrak {m}}}(\texttt{E}) \cdot I + \tfrac{1}{2} \big ( z, \Big ({\begin{matrix} -\mathcal {L}_{{\mathfrak {m}}} &  0 \\ 0 &  \textrm{Id} \end{matrix}}\Big ) \big )_{L^2} + \sqrt{\varepsilon }P_{\varepsilon }( A(\theta ,I,z)) , \end{aligned}$$

with $\vec {\omega }_{{\mathfrak {m}}}(\texttt{E})$ as in (1.18), $P_{\varepsilon }$ a perturbative contribution from the nonlinear terms and $A(\theta ,I,z)$ the action-angle map as in (4.33). The frequency vector $\omega \in {{\mathbb {R}}}^{\kappa _{0}}$ becomes a parameter to determine in order to get a solutions of $\textbf{F}(i)=0$. Here a significant difficulty that was not present in previous works appears.

We first recall the strategy used in the previous works. In the spirit of analysis of Hamiltonian dynamics of Herman–Féjoz [23], one usually relaxes the problem by introducing a counterterm $\alpha \in {{\mathbb {R}}}^{\kappa _{0}}$ and modifying the Hamiltonian $\mathcal {H}_{\varepsilon }$ in (1.32) as

$$\begin{aligned} {\widetilde{\mathcal {H}}}_{\alpha } := \alpha \cdot I + \tfrac{1}{2} \big ( z, \Big ({\begin{matrix} -\mathcal {L}_{{\mathfrak {m}}} &  0 \\ 0 &  \textrm{Id} \end{matrix}}\Big ) \big )_{L^2} + \sqrt{\varepsilon }P_{\varepsilon }(A(\theta ,I,z)) . \end{aligned}$$

The counterterm $\alpha$ becomes an unknown of the problem together with the embedding $i(\textbf{x})$ and one searches for solutions of

1.33 $$\begin{aligned} {\widetilde{\textbf{F}}}(i,\alpha ) = {\widetilde{\textbf{F}}}(\omega ,\texttt{E},\varepsilon ;i,\alpha ) := \omega \cdot \partial _{\textbf{x}}i(\textbf{x}) - X_{{\widetilde{\mathcal {H}}}_{\alpha }}(i(\textbf{x}))=0. \end{aligned}$$

One then obtains a solution $(i_\infty ,\alpha _{\infty })(\omega ,\texttt{E},\varepsilon )$, defined for all parameters $(\omega ,\texttt{E})\in {{\mathbb {R}}}^{\kappa _{0}}\times [\texttt{E}_1,\texttt{E}_2]$, such that (1.33) is solved whenever the parameters satisfy the Diophantine non-resonance condition

1.34 $$\begin{aligned} | \omega \cdot \ell | \geqslant \upsilon \mathinner {\langle {\ell }\rangle }^{-\tau } \quad \forall {{\mathbb {Z}}}^{\kappa _{0}}\setminus \{0\}. \end{aligned}$$

The original equation $\textbf{F}(i_{\infty })=0$ is then solved if $\alpha _{\infty }=\alpha _{\infty }(\omega ,\texttt{E},\varepsilon ) = \vec {\omega }_{{\mathfrak {m}}}(\texttt{E})$ and, since $\alpha _{\infty }(\cdot ,\texttt{E},\varepsilon )$ is expected to be invertible for any fixed $\texttt{E}$, this should fix ${\widetilde{\omega }}={\widetilde{\omega }}(\texttt{E},\varepsilon ) = \alpha _{\infty }^{-1}(\vec {\omega }_{{\mathfrak {m}}}(\texttt{E}),\texttt{E},\varepsilon )$. To ensure that ${\widetilde{\omega }}={\widetilde{\omega }}(\texttt{E})$ satisfies the non-resonance condition (1.34), we need to control a finite number of derivatives in the parameter $\texttt{E}\in [\texttt{E}_1,\texttt{E}_2]$. However, the basis of eigenfunctions $\phi _{j,{\mathfrak {m}}}(y)$ of the operator $\mathcal {L}_{{\mathfrak {m}}}$ in (1.5) is not the standard exponential basis and depends explicitly on the parameter $\texttt{E}$, with the consequence that also the Sobolev phase spaces $\mathcal {X}_{\perp }^s$ vary with respect to the parameter. We do not have a clear and explicit control on the variations of the eigenfunctions with respect to the parameter $\texttt{E}$, as well as of the local nonlinearities. This may be a potential source of divergences in the estimates that may prevent the imposition of the non-resonance conditions.

We apply here a new strategy. We solve (1.31)–(1.32) for a fixed value of the depth $\texttt{E}\in (\texttt{E}_1,\texttt{E}_2)$ such that $\vec {\omega }_{{\mathfrak {m}}}(\texttt{E})$ is a Diophantine non-resonant frequency vector. We will prove in Proposition 5.7 that this property holds for most values of $\texttt{E}$ in any compact interval $[\texttt{E}_1,\texttt{E}_2]$. For any $\varepsilon >0$, we introduce an auxiliary parameter

$$\begin{aligned} \texttt{A}\in \mathcal {J}_{\varepsilon }(\texttt{E}):= [\texttt{E}-\sqrt{\varepsilon },\texttt{E}+\sqrt{\varepsilon }] \end{aligned}$$

so that $\vec {\omega }_{{\mathfrak {m}}}(\texttt{A})$ is close to the unperturbed frequency vector $\vec {\omega }_{{\mathfrak {m}}}(\texttt{E})$ with estimates

1.35 $$\begin{aligned} \big | \partial _{\texttt{A}}^n \big ( \vec {\omega }_{{\mathfrak {m}}}(\texttt{E})- \vec {\omega }_{{\mathfrak {m}}}(\texttt{A}) \big ) \big | \lesssim \sqrt{\varepsilon }, \quad \forall n\in {{\mathbb {N}}}_0, \quad \forall \,\texttt{A}\in \mathcal {J}_{\varepsilon }(\texttt{E}). \end{aligned}$$

We remark that the properties of non-degeneracy and transversality for the vector $\vec {\omega }_{{\mathfrak {m}}}(\texttt{E})$ (see Theorem 5.5 and Proposition 5.6) hold also for $\vec {\omega }_{{\mathfrak {m}}}(\texttt{A})$ and its perturbations on the whole interval $[\texttt{E}_1,\texttt{E}_2]$ with constants that are independent of $\varepsilon >0$. By adding and subtracting the term $\vec {\omega }_{{\mathfrak {m}}}(\texttt{A})\cdot I$ in (1.32), we now introduce the counterterm $\alpha \in {{\mathbb {R}}}^{\kappa _{0}}$ and we consider the modified Hamiltonian

1.36 $$\begin{aligned} \begin{aligned} \mathcal {H}_{\varepsilon ,\alpha }&:= \alpha \cdot I + \tfrac{1}{2} \big ( z, \Big ({\begin{matrix} -\mathcal {L}_{{\mathfrak {m}}} &  0 \\ 0 &  \textrm{Id} \end{matrix}}\Big ) \big )_{L^2} \\&\ \ \ \ \ + \sqrt{\varepsilon }\big ( P_{\varepsilon }(A(\theta ,I,z) + \tfrac{1}{\sqrt{\varepsilon }} \big (\vec {\omega }_{{\mathfrak {m}}}(\texttt{E})-\vec {\omega }_{{\mathfrak {m}}}(\texttt{A}) \big )\cdot I \big ). \end{aligned} \end{aligned}$$

The great advantage of this procedure is that the modified Hamiltonian $\mathcal {H}_{\varepsilon ,\alpha }$ directly depends on the new parameter $\texttt{A}$ only through the correction term $\big (\vec {\omega }_{{\mathfrak {m}}}(\texttt{E})-\vec {\omega }_{{\mathfrak {m}}}(\texttt{A}) \big )\cdot I$, which is perturbative because of the estimates (1.35). As before, the counterterm $\alpha$ becomes an unknown of the problem together with the embedding $i(\textbf{x})$ and we search for solutions of

1.37 $$\begin{aligned} \mathcal {F}(i,\alpha ) = \mathcal {F}(\omega ,\texttt{A},\texttt{E},\varepsilon ;i,\alpha ) := \omega \cdot \partial _{\textbf{x}}i(\textbf{x}) - X_{\mathcal {H}_{\varepsilon ,\alpha }}(i(\textbf{x}))=0. \end{aligned}$$

We stress once more that the nonlinear functional in (1.37) still depends on $\texttt{E}$, but its value is fixed during the Nash–Moser estimate and we are not interest in how the solutions vary with respect to it. The Nash–Moser scheme is not affected by this modification and we will obtain a solution $(i_\infty ,\alpha _{\infty })(\omega ,\texttt{A},\varepsilon )$, defined for all parameters $(\omega ,\texttt{A})\in {{\mathbb {R}}}^{\kappa _{0}}\times \mathcal {J}_{\varepsilon }(\texttt{E})$, such that (1.37) is solved whenever the parameters satisfy the Diophantine non-resonance condition (1.34). The modified Hamiltonian $\mathcal {H}_{\varepsilon ,\alpha _{\infty }}$ in (1.36) will therefore coincide again with $\mathcal {H}_{\varepsilon }$ in (1.32), and consequently the original equation $\textbf{F}(i_{\infty })=0$ in (1.31) will be solved, if $\alpha _{\infty }=\alpha _{\infty }(\omega ,\texttt{A},\varepsilon ) = \vec {\omega }_{{\mathfrak {m}}}(\texttt{A})$. Since $\alpha _{\infty }(\,\cdot \,,\texttt{A},\varepsilon )$ will be invertible for any fixed $\texttt{A}$, this will fix ${\widetilde{\omega }}={\widetilde{\omega }}(\texttt{A},\varepsilon ) = \alpha _{\infty }^{-1}(\vec {\omega }_{{\mathfrak {m}}}(\texttt{A}),\texttt{A},\varepsilon )$. It will finally be possible to prove that the perturbed frequency vector ${\widetilde{\omega }}(\texttt{A},\varepsilon )$ is Diophantine for most values of $\texttt{A}\in \mathcal {J}_{\varepsilon }(\texttt{E})$: we will prove this in Theorem 6.1. We remark that the Diophantine conditions for ${\widetilde{\omega }}(\texttt{A};\varepsilon )$ will be weaker than the ones for $\vec {\omega }_{{\mathfrak {m}}}(\texttt{E})$.

Among the reasons why our modified scheme actually works, we identified the following factors that certainly help the convergence to our result: Equation (1.12) for ${\varphi }$ at the end of the day is semilinear, so there is no need to perform any regularization of the linearized vector field; the linearized operator in the normal direction is directly invertible without any reducibility to a diagonal operator and, therefore, no Melnikov non-resonance conditions are needed; the only non-resonance conditions that appear are the Diophantine conditions on the frequency vectors when we invert the operator $\omega \cdot \partial _{\textbf{x}}$ on functions with zero average in $\textbf{x}\in {{\mathbb {T}}}^{\kappa _{0}}$. We surely find of great interest to see if our strategy still works or can be further improved when these cases are not met and still we have a parament-dependent basis of eigenfunctions.

**A final comment on the parameters and their interdependences.** The construction of the space quasi-periodic stream functions in Theorem 1.1 requires several parameters that we have to tune and match appropriately throughout the entire paper. For sake of clarity, we list all of them:

$\bullet$ $\kappa _0\in {{\mathbb {N}}}$ is the number of frequencies of oscillations and ultimately the dimension of the quasi-periodicity. It is fixed once for all at the very beginning;

$\bullet$ $\texttt{E}\in [\texttt{E}_1,\texttt{E}_2]$, $\texttt{E}_1\gg (\kappa _0+\tfrac{1}{4})\pi$, and $\texttt{r}\in (0,1)$ parametrize the potential $Q_{{\mathfrak {m}}}(y)$ in (1.3), (1.27), affecting its depth and its width, respectively. They are related by the constraint (1.4). The threshold $\texttt{E}_1$ will be chosen sufficiently large in Section 5, depending only on $\kappa _0$. The parameter $\texttt{r}$ will measure the proximity to the Couette flow, see Proposition 3.4;

$\texttt{A}\in \mathcal {J}_{\varepsilon }(\texttt{E}):=[\texttt{E}-\sqrt{\varepsilon },\texttt{E}+\sqrt{\varepsilon }]$ is the auxiliary parameter close to a fixed value of $\texttt{E}\in (\texttt{E}_1,\texttt{E}_2)$ with $\sqrt{\varepsilon }$, where $\varepsilon \in (0,\varepsilon _0)$ is the size of the perturbation ${\varphi }_{\varepsilon }$ in (1.23). This parameter will be used to prove non-resonance condition for the final frequency of oscillations;

$\bullet$ ${\mathfrak {m}}\geqslant {\overline{{\mathfrak {m}}}}\gg 1$ is a large parameter measuring how close the analytic potential $Q_{{\mathfrak {m}}}(y)$ in (1.27) is to the singular well potential in (1.3). The threshold ${\overline{{\mathfrak {m}}}}$ will be chosen sufficiently large, depending on $\texttt{E}$, $\texttt{r}$ and $\kappa _0$. Once this large threshold is determined, the value ${\mathfrak {m}}$ can be arbitrarily fixed once for all.

$\bullet$ $S \in {{\mathbb {N}}}$ is large, but finite, regularity that we impose on the local nonlinearities in (1.12). Its value is ultimately fixed when we estimate the Nash–Moser iterations in Section 8 and it will depend only on $\kappa _0$ and the loss of derivatives coming from the Diophantine conditions in (2.10);

$\bullet$ $\eta \in [0,{\overline{\eta }}]$, with ${\overline{\eta }}\ll 1$, is a small parameter parametrizing the modification of the local nonlinearities around the critical values $\psi _{{\mathfrak {m}}}(\texttt{y}_{p,{\mathfrak {m}}})$, $p=1,..,\kappa _0$. The threshold ${\overline{\eta }}$ will depend on ${\mathfrak {m}}$ and *S*, once the value of ${\mathfrak {m}}\geqslant {\overline{{\mathfrak {m}}}}$ is fixed. Ultimately, the value of $\eta$ is linked to the size $\varepsilon$ of the quasi-periodic perturbation ${\varphi }_{\varepsilon }$ in (1.23) by (4.22).

**Outline of the paper.** The rest of the paper is organized as follows. In Section 2, we recall the functional setting and the basic lemmata that we will use in the following. Section 3 is devoted to the analysis of the shear equilibrium $\psi _{{\mathfrak {m}}}(y)$. In particular, we estimate the proximity to the Couette flow in Proposition 3.4, we determine the local nonlinearities for the second order ODE satisfied by the stream function $\psi _{{\mathfrak {m}}}(y)$ in Theorem 3.7 and we analyse the spectral properties of the linear operator $\mathcal {L}_{{\mathfrak {m}}}= -\partial _{y}^2 + Q_{{\mathfrak {m}}}(y)$ in Proposition 3.10. In Section 4 we set the partial differential equation that we will solve, its Hamiltonian formulation with the action-angle variables and the nonlinear functional map for the Nash–Moser Theorem 4.5. In Section 5 we prove the non-degeneracy and the transversality properties for the negative eigenvalues of the operator $\mathcal {L}_{{\mathfrak {m}}}$ that are needed to impose Diophantine non-resonance conditions on them, together with the measure estimates for the final frequencies in Theorem 6.1 of Section 6. In Section 7 we study the approximate inverse of the linearized vector field at any approximate solution of the Nash–Moser nonlinear iteration. We conclude with Section 8 with the Proof of Theorem 4.5, which directly implies the validity of Theorem 1.1.

## Functional Setting

In this paper we consider functions in the following Sobolev space

2.1 $$\begin{aligned}&H^{s,\rho } := H^s({{\mathbb {T}}}^{\kappa _0},H_0^\rho ([-1,1])) \nonumber \\&\quad := \Big \{ u(\textbf{x},y)=\!\sum _{\ell \in {{\mathbb {Z}}}^{\kappa _0}} u_{\ell }(y)e^{\textrm{i}\ell \cdot \textbf{x}} \,:\, \Vert u \Vert _{s,\rho }^2:=\! \sum _{\ell \in {{\mathbb {Z}}}^{\kappa _0}} \mathinner {\langle {\ell }\rangle }^{2s}\Vert u_\ell \Vert _{H_0^\rho ([-1,1])}^2<\infty \Big \}, \end{aligned}$$

where $\mathinner {\langle {\ell }\rangle }:=\max \{1,|\ell |\}$ and, recalling (1.20),

$$\begin{aligned} H_0^\rho ([-1,1]) := \big \{ \,u(y) \in H^\rho ([-1,1]) \,:\, u(-1)=u(1)=0, \ u(-y)=u(y)\, \big \}. \end{aligned}$$

For $s \geqslant \frac{\kappa _0}{2}+1$, $\rho \geqslant 1$, we have that $H^{s,\rho }\subset \mathcal {C}({{\mathbb {T}}}^{\kappa _0}\times [-1,1])$ and that $H^{s, \rho }$ is an algebra.

**Whitney–Sobolev functions.** We consider families of Sobolev functions

$$\begin{aligned} \lambda =(\omega ,\texttt{A})\mapsto u(\lambda )=u(\lambda ;\textbf{x},y) \in H^{s,\rho }, \end{aligned}$$

which are $k_0$-times differentiable in the sense of Whitney with respect to the parameter $\lambda = (\omega ,\texttt{A}) \in F \subset {{\mathbb {R}}}^{\kappa _0+1}$ where $F\subset {{\mathbb {R}}}^{\kappa _0+1}$ is a closed set. We refer to Definition 2.1 in [2], for the definition of Whitney–Sobolev functions. Given $\upsilon \in (0,1)$, by the Whitney extension theorem (for example Theorem B.2, [2]), we have the equivalence

2.2 $$\begin{aligned} \Vert u \Vert _{s,\rho ,F}^{k_0,\upsilon } \sim _{\kappa _0,k_0} {\mathop \sum }_{\left| \alpha \right| \leqslant k_0} \upsilon ^{\left| \alpha \right| } \Vert \partial _{\lambda }^\alpha \mathcal {E}_{k}u \Vert _{L^\infty ({{\mathbb {R}}}^{\kappa _0+1},H^{s-|\alpha |,\rho })}, \end{aligned}$$

where $\mathcal {E}_{k}u$ denotes an extension of *u* to all the parameter space ${{\mathbb {R}}}^{\kappa _{0}+1}$. For simplicity, we denote $\Vert \ \Vert _{s,\rho ,F}^{k_0,\upsilon } = \Vert \ \Vert _{s,\rho }^{k_0,\upsilon }$, we use the right hand side of (2.2) as definition of the norm itself, we denote $\mathcal {E}_{k}u$ by *u* and we still denote the function spaces by $H^{s,\rho }$. In particular, we shall deal with functions with Sobolev regularity $s\geqslant s_0$, where the threshold regularity $s_0$ is chosen as

2.3 $$\begin{aligned} s_0:=s_0(\kappa _0,k_0):=\lfloor \tfrac{\kappa _0}{2}\rfloor +1+k_0 \in {{\mathbb {N}}}. \end{aligned}$$

### Lemma 2.1

(*i*) For all $s\geqslant s_0$, $\rho \geqslant 1$ and any $u,v\in H^{s,\rho }$,

2.4 $$\begin{aligned} \Vert u v \Vert _{s, \rho }^{k_0,\upsilon } \leqslant C(s,k_0) \Vert u \Vert _{s, \rho }^{k_0,\upsilon }\Vert v \Vert _{s_0, \rho }^{k_0,\upsilon } + C(s_0,k_0)\Vert u \Vert _{s_0, \rho }^{k_0,\upsilon }\Vert v \Vert _{s, \rho }^{k_0,\upsilon }. \end{aligned}$$

(*ii*) Let $s \geqslant s_0$, $a \in H^{s, 1}$, $u \in H^{s, 0}$. Then $a u \in H^{s, 0}$ and

2.5 $$\begin{aligned} \Vert a u \Vert _{s, 0}^{k_0, \upsilon } \lesssim _s \Vert a \Vert _{s_0, 1}^{k_0, \upsilon } \Vert u \Vert _{s, 0}^{k_0, \upsilon } + \Vert a \Vert _{s, 1}^{k_0, \upsilon } \Vert u \Vert _{s_0, 0}^{k_0, \upsilon }. \end{aligned}$$

Similarly if $a \in H^s({{\mathbb {T}}}^{\kappa _0})$ and $u \in H^{s, 0}$, then $a u \in H^{s, 0}$ and

2.6 $$\begin{aligned} \Vert au \Vert _{s, 0}^{k_0, \upsilon } \lesssim _s \Vert a \Vert _{s_0}^{k_0, \upsilon } \Vert u \Vert _{s, 0}^{k_0, \upsilon } + \Vert a \Vert _{s}^{k_0, \upsilon } \Vert u \Vert _{s_0, 0}^{k_0, \upsilon }. \end{aligned}$$

### Proof

Proof of (*i*). For parameter independent Sobolev functions, that is $k_0=0$, the tame estimates (2.4) follows from standard tame estimates arguments, using the algebra property for functions in $H_0^{\rho }([-1,1])$ for $\rho \geqslant 1$, see Lemma 2.9 in [11]. In general, the tame estimates (2.4) follows as in [3, 12] with respect to the definition of the weighted norm in (2.2) and the choice of $s_0$ in (2.3) (here, as in [3, 9], the norm of $\partial _{\omega }^\alpha u$, $|\alpha |\leqslant k_0$, is estimated in $H^{s-|\alpha |, \rho }$, whereas in [2, 8, 12] is estimated just in $H^s$).

Proof of (*ii*). To simplify notations, we write $\Vert \cdot \Vert _{s, \rho }$ instead of $\Vert \cdot \Vert _{s, \rho }^{k_0, \upsilon }$. By expanding *a* and *u* in Fourier series with respect to $\textbf{x} \in {{\mathbb {T}}}^{\kappa _0}$, we have $a(\textbf{x}, y) = \sum _{\ell \in {{\mathbb {Z}}}^{\kappa _0}} a_\ell (y) e^{\textrm{i}\ell \cdot \textbf{x}}$ and $u(\textbf{x}, y) = \sum _{\ell \in {{\mathbb {Z}}}^{\kappa _0}} u_\ell (y) e^{\textrm{i}\ell \cdot \textbf{x}}$, implying that

$$\begin{aligned} a(\textbf{x}, y) u(\textbf{x}, y) = \sum _{\ell , \ell ' \in {{\mathbb {Z}}}^{\kappa _0}} a_{\ell - \ell '}(y) u_{\ell '}(y) e^{\textrm{i}\ell \cdot \textbf{x}}. \end{aligned}$$

Therefore

2.7 $$\begin{aligned} \Vert a u \Vert _{s, 0}^2 \leqslant \sum _{\ell \in {{\mathbb {Z}}}^{\kappa _0}} \langle \ell \rangle ^{2 s} \Big ( \sum _{\ell '\in {{\mathbb {Z}}}^{\kappa _0}} \Vert a_{\ell - \ell '} u_{\ell '} \Vert _{L^2_y} \Big )^2. \end{aligned}$$

Using the embedding $H_0^1([- 1, 1]) \subset L^\infty ([- 1, 1])$, $\Vert \cdot \Vert _{L^\infty _y} \lesssim \Vert \cdot \Vert _{H^1_y}$, we have that, for any $\ell , \ell ' \in {{\mathbb {Z}}}^{\kappa _0}$,

$$\begin{aligned} \Vert a_{\ell - \ell '} u_{\ell '}\Vert _{L^2_y} \leqslant \Vert a_{\ell - \ell '} \Vert _{L^\infty _y} \Vert u_{\ell '} \Vert _{L^2_y} \lesssim \Vert a_{\ell - \ell '} \Vert _{H^1_y} \Vert u_{\ell '} \Vert _{L^2_y}. \end{aligned}$$

Therefore, the inequality (2.7) leads to

$$\begin{aligned} \begin{aligned} \Vert a u \Vert _{s, 0}^2&\lesssim \sum _{\ell \in {{\mathbb {Z}}}^{\kappa _0}} \langle \ell \rangle ^{2 s} \Big ( \sum _{\ell '\in {{\mathbb {Z}}}^{\kappa _0}} \Vert a_{\ell - \ell '} \Vert _{H^1_y} \Vert u_{\ell '} \Vert _{L^2_y} \Big )^2 \lesssim _s \mathcal {I}_1 + \mathcal {I}_2, \\ \mathcal {I}_1&:= \sum _{\ell \in {{\mathbb {Z}}}^{\kappa _0}} \Big ( \sum _{\ell '\in {{\mathbb {Z}}}^{\kappa _0}} \langle \ell - \ell ' \rangle ^{s} \Vert a_{\ell - \ell '} \Vert _{H^1_y} \Vert u_{\ell '} \Vert _{L^2_y} \Big )^2 , \\ \mathcal {I}_2&:= \sum _{\ell \in {{\mathbb {Z}}}^{\kappa _0}} \Big ( \sum _{\ell '\in {{\mathbb {Z}}}^{\kappa _0}} \langle \ell ' \rangle ^s\Vert a_{\ell - \ell '} \Vert _{H^1_y} \Vert u_{\ell '} \Vert _{L^2_y} \Big )^2 \end{aligned} \end{aligned}$$

where we have used the trivial fact $\langle \ell \rangle ^s \lesssim _s \langle \ell ' \rangle ^s + \langle \ell - \ell ' \rangle ^s$, for any $\ell , \ell ' \in {{\mathbb {Z}}}^{\kappa _0}$. By multiplying and dividing by $\langle \ell ' \rangle ^{s_0}$, using that $\sum _{\ell '} \langle \ell ' \rangle ^{- 2 s_0} < + \infty$ and by the Cauchy–Schwartz inequality, we estimate the term $\mathcal {I}_1$ with

$$\begin{aligned} \begin{aligned} \mathcal {I}_1&\lesssim \sum _{\ell \in {{\mathbb {Z}}}^{\kappa _0}} \sum _{\ell ' \in {{\mathbb {Z}}}^{\kappa _0}} \langle \ell - \ell ' \rangle ^{2s} \Vert a_{\ell - \ell '} \Vert _{H^1_y}^2 \langle \ell ' \rangle ^{2 s_0} \Vert u_{\ell '} \Vert _{L^2_y}^2 \\&\lesssim \sum _{\ell ' \in {{\mathbb {Z}}}^{\kappa _0}} \langle \ell ' \rangle ^{2 s_0} \Vert u_{\ell '} \Vert _{L^2_y}^2 \sum _{\ell \in {{\mathbb {Z}}}^{\kappa _0}} \langle \ell - \ell ' \rangle ^{2 s} \Vert a_{\ell - \ell '} \Vert _{H^1_y}^2 \lesssim \Vert a \Vert _{s, 1}^2 \Vert u \Vert _{s_0, 0}^2. \end{aligned} \end{aligned}$$

By similar arguments, one can show that $\mathcal {I}_2 \lesssim \Vert a \Vert _{s_0, 1}^2 \Vert u \Vert _{s, 0}^2$ which implies the claimed interpolation estimate for $\Vert a u \Vert _{s, 0}$. In order to estimate $\Vert au \Vert _{s, 0}^{k_0, \upsilon }$ one has to estimate for any $\alpha \in {{\mathbb {N}}}^{\kappa _0}$ with $|\alpha | \leqslant k_0$,

$$\begin{aligned} \Vert \partial _\omega ^\alpha (a u) \Vert _{s - |\alpha |, 0} \lesssim _\alpha \sum _{\alpha _1 + \alpha _2 = \alpha } \Vert (\partial _\omega ^{\alpha _1}a)(\partial _\omega ^{\alpha _2} u) \Vert _{s - |\alpha |, 0} \end{aligned}$$

and every term of the latter sum is estimated as above.

The estimate (2.6) is proved similarly to (2.5) (it is actually easier since *a* does not depend on *y*). $\square$

For any $K>0$, we define the smoothing projections

2.8 $$\begin{aligned} u(\textbf{x},y) =\! \! \sum _{\ell \in {{\mathbb {Z}}}^{\kappa _0}} u_\ell (y) e^{\textrm{i}\,\ell \cdot \textbf{x}} \mapsto \Pi _{K}u(\textbf{x},y):=\!\! \sum _{|\ell |\leqslant K} u_\ell (y) e^{\textrm{i}\,\ell \cdot \textbf{x}}, \quad \Pi _{K}^\perp := \textrm{Id} - \Pi _{K}.\nonumber \\ \end{aligned}$$

The following estimates hold for the smoothing operators defined in (2.8):

2.9 $$\begin{aligned} \Vert \Pi _K u \Vert _{s, \rho }^{k_0,\upsilon } \leqslant K^\alpha \Vert u \Vert _{s-\alpha , \rho }^{k_0,\upsilon }, \ 0\leqslant \alpha \leqslant s , \quad \Vert \Pi _K^\perp u \Vert _{s, \rho }^{k_0,\upsilon } \leqslant K^{-\alpha } \Vert u \Vert _{s+\alpha , \rho }^{k_0,\upsilon }, \ \alpha \geqslant 0. \nonumber \\ \end{aligned}$$

We also recall the standard Moser tame estimate for the nonlinear composition operator

$$\begin{aligned} u(\textbf{x},y)\mapsto \texttt{f}(u)(\textbf{x},y) :=f(\textbf{x},y,u(\textbf{x},y)). \end{aligned}$$

For the purposes of this paper, we state this result in the case of finite regularity of the nonlinear function.

### Lemma 2.2

(Composition operator) Let $s \geqslant s_0$, $\rho \in {{\mathbb {N}}}$. Then there exists $\sigma = \sigma ( \rho ) > 0$ such that for any $f\in \mathcal {C}^{s + \sigma }({{\mathbb {T}}}^{\kappa _0}\times [-1,1]\times {{\mathbb {R}}},{{\mathbb {R}}})$, if $u(\lambda )\in H^{s,\rho }$ is a family of Sobolev functions satisfying $\Vert u \Vert _{s_0, \rho }^{k_0,\upsilon }\leqslant 1$, then

$$\begin{aligned} \Vert \texttt{f}(u) \Vert _{s, \rho }^{k_0,\upsilon }\leqslant C(s,k_0) \Vert f \Vert _{\mathcal {C}^{s + \sigma }}\big ( 1+\Vert u \Vert _{s, \rho }^{k_0,\upsilon } \big ) . \end{aligned}$$

If $f(\textbf{x},y, 0) = 0$, then $\Vert \texttt{f}(u) \Vert _{s, \rho }^{k_0,\upsilon }\leqslant C(s,k_0) \Vert f \Vert _{\mathcal {C}^{s + \sigma }} \Vert u \Vert _{s, \rho }^{k_0,\upsilon }$. Moreover, if $f\in \mathcal {C}^\infty$, then the same result holds for any $s\geqslant s_0$.

### Proof

See for example Lemmata 2.14, 2.15 in [11] and Lemma 2.6 in [2]. The proof relies on the multilinear Leibniz rule, on the Faà di Bruno formula, on the tame estimates in (2.4) and on interpolation inequalities. $\square$

**Diophantine equation.** If $\omega$ is a Diophantine vector in $\texttt{D}\texttt{C}(\upsilon ,\tau )$, defined by

2.10 $$\begin{aligned} \texttt{D}\texttt{C}(\upsilon ,\tau ):=\Big \{ \omega \in {{\mathbb {R}}}^{\kappa _0}\, : \, |\omega \cdot \ell |\geqslant \upsilon \mathinner {\langle {\ell }\rangle }^{-\tau } \ \forall \,\ell \in {{\mathbb {Z}}}^{\kappa _0}\setminus \{0\} \Big \}, \end{aligned}$$

then the equation $\omega \cdot \partial _\textbf{x}v = u$, where $u(\textbf{x})$ has zero average with respect to $\textbf{x}\in {{\mathbb {T}}}^{\kappa _0}$, has the periodic solution

$$\begin{aligned} (\omega \cdot \partial _\textbf{x})^{-1} u(\textbf{x}) := \sum _{\ell \in {{\mathbb {Z}}}^{\kappa _0}\setminus \{0\}} \frac{u_{\ell }}{\textrm{i}\,\omega \cdot \ell } e^{\textrm{i}\ell \cdot \textbf{x}} . \end{aligned}$$

For $F\subseteq \texttt{D}\texttt{C}(\upsilon ,\tau )$, one has

$$\begin{aligned} \Vert (\omega \cdot \partial _\textbf{x})^{-1}u \Vert _{s,\rho ,F}^{k_0,\upsilon } \leqslant C(k_0)\upsilon ^{-1}\Vert u \Vert _{s+\mu ,\rho ,F}^{k_0,\upsilon }, \quad \mu :=k_0+\tau (k_0+1) . \end{aligned}$$

**Reversible and reversibility preserving conditions.** In the next sections we will consider (spatial) reversible and reversibility maps in order to preserve the symmetry of the solutions (1.20). To do so, let $\mathcal {S}$ be the involution acting on the real variables $\zeta =(\zeta _1,\zeta _2)\in {{\mathbb {R}}}^2$ defined by

2.11 $$\begin{aligned} \mathcal {S}: \begin{pmatrix} \zeta _1(y) \\ \zeta _2(y) \end{pmatrix}\mapsto \begin{pmatrix} \zeta _1(-y) \\ -\zeta _2(-y) \end{pmatrix}. \end{aligned}$$

In action-angle variables $\zeta = (\theta ,I,w) \in {{\mathbb {T}}}^{\kappa _0}\times {{\mathbb {R}}}^{\kappa _0}\times {{\mathbb {R}}}^2$, which will be introduced in (4.33), we consider the following involution

2.12 $$\begin{aligned} \vec {\mathcal {S}} : (\theta ,I,z) \mapsto (-\theta ,I,\mathcal {S}z). \end{aligned}$$

Let $\textbf{x}\in {{\mathbb {T}}}^{\kappa _0}$. A function $\zeta (\textbf{x},\,\cdot \,)$ is called *(spatial) reversible* if $\mathcal {S}\zeta (\textbf{x},\,\cdot \,)= \zeta (-\textbf{x},\,\cdot \,)$ and *anti-reversible* if $-\mathcal {S}\zeta (\textbf{x},\,\cdot \,)= \zeta (-\textbf{x},\,\cdot \,)$. The same definition holds in action-angle variables $(\theta ,I,z)$ with the involution $\mathcal {S}$ in (2.11) replaced by $\vec {\mathcal {S}}$ in (2.12).

A $\textbf{x}$-dependent family of operators $\mathcal {R}(\textbf{x})$, $\textbf{x}\in {{\mathbb {T}}}^{\kappa _0}$, is *reversible* if $\mathcal {R}(-\textbf{x}) \circ \mathcal {S}= - \mathcal {S}\circ \mathcal {R}(\textbf{x})$ for all $\textbf{x}\in {{\mathbb {T}}}^{\kappa _0}$ and it is *reversibility preserving* if $\mathcal {R}(-\textbf{x}) \circ \mathcal {S}= \mathcal {S}\circ \mathcal {R}(\textbf{x})$ for all $\textbf{x}\in {{\mathbb {T}}}^{\kappa _0}$. A reversibility preserving operator maps reversible, respectively anti-reversible, functions into reversible, respectively anti-reversible, functions, see Lemma 3.22 in [8]. We remark also that, if *X* is a reversible vector field, namely $X\circ \mathcal {S}= - \mathcal {S}\circ X$, and $\zeta (\textbf{x},\,\cdot \,)$ is a reversible function, then the linearized operator $\textrm{d}_\zeta X(\zeta (\textbf{x},\,\cdot \,))$ is reversible, see for example Lemma 3.22 in [8].

## A Shear Equilibrium Close to Couette with Oscillations

The goal of this quite lengthy section is to construct and analyse the shear flow $(\psi _{{\mathfrak {m}}}'(y),0)$ with stream function $\psi _{{\mathfrak {m}}}(y)$ that in an equilibrium configuration from which we will bifurcate the space quasi-periodic solutions of the stationary Euler equations. The properties of the stream function $\psi _{{\mathfrak {m}}}(y)$ are essentially dictated by the analytic potential function $Q_{{\mathfrak {m}}}(y)$ in (3.1), which is suitably engineered to meet regularity properties, smallness estimates and parameter dependences. First, in Section 3.1 we show that the stream function $\psi _{{\mathfrak {m}}}(y)$ is close to the Couette stream function $\psi _{\textrm{cou}}(y):=\tfrac{1}{2} y^2$ with respect to the $H^3$-norm, see Proposition 3.4 . In Section 3.2 we prove Theorem 3.7, which states the existence of local nonlinearities such that $\psi _{{\mathfrak {m}}}(y)$ solves the second-order nonlinear ODE (3.37). We conclude in Sect. 3.3 with the spectral analysis of the linear operator $\mathcal {L}_{{\mathfrak {m}}}:=-\partial _y^2 +Q_{{\mathfrak {m}}}(y)$ in Proposition 3.10.

### The Potential $Q_{{\mathfrak {m}}}(y)$ and the Proximity to the Couette Flow

We consider a shear flow $(u,v)=(\psi _{{\mathfrak {m}}}'(y),0)$ on ${{\mathbb {R}}}\times [-1,1]$, where $\psi _{{\mathfrak {m}}}'(y)$ is the odd solution of the second-order ODE

3.1 $$\begin{aligned} \psi _{{\mathfrak {m}}}'''(y) = Q_{\mathfrak {m}}(y) \psi _{{\mathfrak {m}}}'(y), \quad Q_{{\mathfrak {m}}} (y):= -\texttt{E}^2\Big (h_{{\mathfrak {m}}}\big ( \tfrac{y}{\texttt{r}} \big ) +g_{{\mathfrak {m}},S}\big (\tfrac{y}{\texttt{r}}\big ) \Big ), \end{aligned}$$

with $\texttt{r}\in (0,1)$, $\texttt{E}>0$, ${\mathfrak {m}},S \in {{\mathbb {N}}}$ and the function $h_{{\mathfrak {m}}}(z)$ given by

3.2 $$\begin{aligned} h_{{\mathfrak {m}}}(z):= \Big ( \Big (\frac{\cosh (z)}{\cosh (1)}\Big )^{\mathfrak {m}}+1 \Big )^{-1}. \end{aligned}$$

The function $g_{\mathfrak {m}}(\tfrac{y}{\texttt{r}})$ is a polynomial corrector which is chosen to satisfy the following property

#### Lemma 3.1

Let $(\texttt{y}_{p,{\mathfrak {m}}})_{{\mathfrak {m}}\in {{\mathbb {N}}}}$, $p=1,...,\kappa _0$ be arbitrary sequences in $(0,\texttt{r})$ converging to $\texttt{y}_{p,\infty }= \frac{p\pi }{\texttt{E}}$. Then, for any $S\in {{\mathbb {N}}}$ and for ${\mathfrak {m}}\gg 1$ sufficiently large, there exists a polynomial function $g_{{\mathfrak {m}},S}(z)$ even in $z:=\frac{y}{\texttt{r}}$ and of degree $d(S,\kappa _0)\in {{\mathbb {N}}}$ such that the analytic potential $Q_{{\mathfrak {m}}}(y)$ satisfies the following finitely many conditions:

3.3 $$\begin{aligned} \partial _y^{2n-1} Q_{\mathfrak {m}}(\pm \texttt{y}_{p,{\mathfrak {m}}}) = 0 \quad \forall \, n=1,...,S, \quad p=1,..., \kappa _0. \end{aligned}$$

Moreover, for any $n\in {{\mathbb {N}}}_0$, the following estimate holds

3.4 $$\begin{aligned} \sup _{y\in [-1,1]}|\partial _{z}^n g_{{\mathfrak {m}},S}(\tfrac{y}{\texttt{r}}) | \lesssim _{n} \sum _{p=1}^{\kappa _0}\sum _{k=1}^{S} |h_{{\mathfrak {m}}}^{(2k-1)}(\texttt{y}_{j,{\mathfrak {m}}})|. \end{aligned}$$

#### Proof

We use a classical Hermite interpolation argument, also referred as Lagrangian interpolation with derivatives. Let ${\widetilde{\texttt{y}}}_{p,{\mathfrak {m}}}:=\frac{\texttt{y}_{p,{\mathfrak {m}}}}{\texttt{r}}$, $p=1,...,\kappa _0$. We search for a polynomial $g_{{\mathfrak {m}},S}(z)$ of the form

3.5 $$\begin{aligned} g_{{\mathfrak {m}},S}(z) = \sum _{j=\pm 1,...,\pm \kappa _0} F_{j}(z), \quad \text {with} \quad F_{j}^{(2n-1)}({\widetilde{\texttt{y}}}_{j',{\mathfrak {m}}}) = 0 \quad \forall \, j'\ne j. \end{aligned}$$

Let $j\in \{\pm 1,...,\pm \kappa _0\}$ be fixed. We construct $F_{j}(z)$ as the linear combination

3.6 $$\begin{aligned} F_{j}(z)= \sum _{k=1}^{S} a_{j,2k-1} f_{j,2k-1}(z), \quad f_{j,2k-1}(z) = \frac{(z-{\widetilde{\texttt{y}}}_{j,{\mathfrak {m}}})^{2k-1}}{(2k-1)!} {{\widehat{f}}}_{j}(z), \end{aligned}$$

where, for $T>1$ arbitrarily large, we define

3.7 $$\begin{aligned} {{\widehat{f}}}_{j}(z):= (z^2-T^2)^{2S} \prod _{j'=\pm 1,...,\pm \kappa _0\atop j'\ne j} (z-{\widetilde{\texttt{y}}}_{j',{\mathfrak {m}}})^{2S} \end{aligned}$$

The structure of (3.6)–(3.7) allows both to impose the condition in (3.5) and to control later the estimates (3.4) by choosing $T\gg 1$. We search now for the coefficients $a_{j,1}, a_{j,3},...,a_{j,2S-1}$ so that the conditions in (3.3) are satisfied, which, by (3.1) and (3.5), amounts to ask

3.8 $$\begin{aligned} F_{j}^{(2n-1)}({\widetilde{\texttt{y}}}_{j,{\mathfrak {m}}}) = c_{j,2n-1} := - h_{{\mathfrak {m}}}^{(2n-1)}({\widetilde{\texttt{y}}}_{j,{\mathfrak {m}}}), \quad \forall \,n=1,...,S. \end{aligned}$$

By differentiating (3.6) at each order $2n-1$, $n=1,...,S$, solving (3.8) amounts to solving the linear system

$$\begin{aligned} \begin{pmatrix} f_{j,1}^{(1)}({\widetilde{\texttt{y}}}_{j,{\mathfrak {m}}}) &  f_{j,3}^{(1)}({\widetilde{\texttt{y}}}_{j,{\mathfrak {m}}}) &  \cdots &  f_{j,2S-1}^{(1)}({\widetilde{\texttt{y}}}_{j,{\mathfrak {m}}}) \\ f_{j,1}^{(3)}({\widetilde{\texttt{y}}}_{j,{\mathfrak {m}}}) &  f_{j,3}^{(3)}({\widetilde{\texttt{y}}}_{j,{\mathfrak {m}}}) &  \cdot &  f_{j,2S-1}^{(3)}({\widetilde{\texttt{y}}}_{j,{\mathfrak {m}}}) \\ \vdots &  \vdots &  \ddots &  \vdots \\ f_{j,1}^{(2S-1)}({\widetilde{\texttt{y}}}_{j,{\mathfrak {m}}}) &  f_{j,3}^{(2S-1)}({\widetilde{\texttt{y}}}_{j,{\mathfrak {m}}}) &  \cdots &  f_{j,2S-1}^{(2S-1)}({\widetilde{\texttt{y}}}_{j,{\mathfrak {m}}}) \end{pmatrix}\begin{pmatrix} a_{j,1} \\ a_{j,3} \\ \vdots \\ a_{j,2S-1} \end{pmatrix} = \begin{pmatrix} c_{j,1} \\ c_{j,3} \\ \vdots \\ c_{j,2S-1} \end{pmatrix}. \end{aligned}$$

The system above is lower triangular. Indeed, by (3.6) and (3.7), we have

$$\begin{aligned} f_{j,2n-1}^{(2n-1)}({\widetilde{\texttt{y}}}_{j,{\mathfrak {m}}}) = {{\widehat{f}}}_{j}({\widetilde{\texttt{y}}}_{j,{\mathfrak {m}}}) \quad \forall \, n=1,...,S, \quad f_{j,2k-1}^{(2n-1)}({\widetilde{\texttt{y}}}_{j,{\mathfrak {m}}})=0 \quad \forall \,k>n. \end{aligned}$$

We solve directly the system (3.8) and we get

3.9 $$\begin{aligned} \begin{aligned} a_{j,1}&= \frac{c_{j,1}}{{{\widehat{f}}}_{j}({\widetilde{\texttt{y}}}_{j,{\mathfrak {m}}})}, \\ a_{j,2k-1}&= \frac{1}{{{\widehat{f}}}_{j}({\widetilde{\texttt{y}}}_{j,{\mathfrak {m}}})} \Big ( c_{j,2k-1} - \sum _{n=1}^{k-1} a_{j,2n-1} f_{j,2n-1}^{(2k-1)}({\widetilde{\texttt{y}}}_{j,{\mathfrak {m}}}) \Big ), \quad k=2,...,S. \end{aligned} \end{aligned}$$

We finally show the estimates in (3.4). By (3.5)–(3.8), taking $T\gg \frac{1}{\texttt{r}} >1$ sufficiently large, the estimates for the coefficients in (3.9) can be made arbitrarily small and we have, for any $n\in {{\mathbb {N}}}_0$,

$$\begin{aligned} \sup _{z\in [-\frac{1}{\texttt{r}},\frac{1}{\texttt{r}}]}|\partial _{z}^n g_{{\mathfrak {m}},S}(z) | \lesssim \sum _{p=1}^{\kappa _0}\sum _{k=1}^{S} |c_{\pm p, 2k-1}| = \sum _{p=1}^{\kappa _0}\sum _{k=1}^{S} |h_{{\mathfrak {m}}}^{(2n-1)}({\widetilde{\texttt{y}}}_{j,{\mathfrak {m}}})|. \end{aligned}$$

This concludes the proof of the claim. $\square$

The potential function $Q_{\mathfrak {m}}(y) = Q_{{\mathfrak {m}}}(\texttt{E},\texttt{r};y)$ is analytic in all its entries and, in the limit ${\mathfrak {m}}\rightarrow +\infty$, it approaches the singular potential

3.10 $$\begin{aligned} Q_\infty (y) = Q_\infty (\texttt{E},\texttt{r};y) := {\left\{ \begin{array}{ll} 0 &  \quad |y| >\texttt{r}, \\ -\texttt{E}^2 &  \quad |y|< \texttt{r}. \end{array}\right. } \end{aligned}$$

#### Lemma 3.2

(Estimates for $Q_{{\mathfrak {m}}}(y)$) We have

3.11 $$\begin{aligned} \sup _{{\mathfrak {m}}\gg 1}\Vert Q_{\mathfrak {m}}\Vert _{L^\infty ([-1,1])} \lesssim \Vert Q_\infty \Vert _{L^\infty ([-1,1])} \lesssim \texttt{E}^2. \end{aligned}$$

Moreover, for any fixed $\gamma >0$ sufficiently small, we have, for some constant $C>1$ and for any $n\in {{\mathbb {N}}}_0$,

3.12 $$\begin{aligned} \sup _{y\in [-1,1] \atop y\notin B_\gamma (\texttt{r})\cup B_\gamma (-\texttt{r})} \! | \partial _{y}^{n}(Q_{{\mathfrak {m}}}(y)-Q_\infty (y))| \leqslant \texttt{E}^2 C^n \left( \frac{{\mathfrak {m}}^n}{\texttt{r}^n} + \sum _{j=1}^{S} \frac{{\mathfrak {m}}^{2k-1}}{\texttt{r}^{2k-1}} \right) e^{-\frac{{\mathfrak {m}}}{2}\gamma }. \nonumber \\ \end{aligned}$$

As a consequence, as ${\mathfrak {m}}\rightarrow \infty$, we have that $| \partial _y^n(Q_{\mathfrak {m}}(y) -Q_\infty (y))| \rightarrow 0$ for any $n\in {{\mathbb {N}}}_0$, uniformly in $y\in {{\mathbb {R}}}\setminus (B_\gamma (\texttt{r})\cup B_\gamma (-\texttt{r}))$, and $\Vert Q_{\mathfrak {m}}- Q_\infty \Vert _{L^p([-1,1])} \rightarrow 0$ for any $p\in [1,\infty )$.

#### Proof

Recalling (3.1), (3.2) and (3.10), we write

3.13 $$\begin{aligned} \begin{aligned}&Q_{\mathfrak {m}}(y)=-\texttt{E}^2{\widetilde{Q}}_{{\mathfrak {m}}}\big (\tfrac{y}{\texttt{r}}\big ), \quad Q_\infty (y)=-\texttt{E}^2{\widetilde{Q}}_{\infty }\big (\tfrac{y}{\texttt{r}}\big ), \quad \text {where}\\&{\widetilde{Q}}_{{\mathfrak {m}}}(z):= h_{{\mathfrak {m}}}(z)+g_{{\mathfrak {m}},S}(z) , \quad {\widetilde{Q}}_{\infty }(z):={\left\{ \begin{array}{ll} 0 &  \quad |z| > 1 , \\ 1 &  \quad |z|< 1 . \end{array}\right. } \end{aligned} \end{aligned}$$

We start by proving (3.12). First, by the estimate (3.4) in Lemma 3.1 and the fact that $| \texttt{y}_{p,{\mathfrak {m}}}| < \texttt{r}$ for any $p=1,...,\kappa _{0}$, we note that, for any $\gamma >0$ sufficiently small,

3.14 $$\begin{aligned} \sup _{y\in [-1,1]}|g_{{\mathfrak {m}},S}^{(n)}(\tfrac{y}{\texttt{r}}) | \lesssim _{n} \sum _{k=1}^{S} \sup _{|y| \leqslant \texttt{r}-\gamma } |h_{{\mathfrak {m}}}^{(2k-1)}\big (\tfrac{y}{\texttt{r}}\big )|, \quad \forall \, n\in {{\mathbb {N}}}_0 . \end{aligned}$$

It implies that $g_{{\mathfrak {m}}}\big ( \tfrac{y}{\texttt{r}} \big )$ and its derivatives are bounded on $[-1,1]$ by finitely many derivatives of $h_{{\mathfrak {m}}}\big ( \tfrac{y}{\texttt{r}}\big )$ on $[-\texttt{r}+\gamma ,\texttt{r}-\gamma ]$. Also, because $g_{{\mathfrak {m}},S}$ is a polynomial, its derivatives of order *n* will identically vanish for any $n\in {{\mathbb {N}}}$ large enough. By (3.13), we estimate

3.15 $$\begin{aligned} \begin{aligned} \sup _{y\in [-1,1] \atop y\notin B_\gamma (\texttt{r})\cup B_\gamma (-\texttt{r})} \big | {\widetilde{Q}}_{{\mathfrak {m}}}^{(n)}\big (\tfrac{y}{\texttt{r}}\big ) - {\widetilde{Q}}_{\infty }^{(n)}\big (\tfrac{y}{\texttt{r}}\big )\big | \leqslant \texttt{J}_{1} + \texttt{J}_{2}, \end{aligned} \end{aligned}$$

where

3.16 $$\begin{aligned} \texttt{J}_{1} := \sup _{y\in [-1,1] \atop y\notin B_\gamma (\texttt{r})\cup B_\gamma (-\texttt{r})} \big | h_{{\mathfrak {m}}}^{(n)}\big (\tfrac{y}{\texttt{r}}\big ) - {\widetilde{Q}}_{\infty }^{(n)}\big (\tfrac{y}{\texttt{r}}\big )\big | , \quad \texttt{J}_{2} :=\sup _{y\in [-1,1] \atop y\notin B_\gamma (\texttt{r})\cup B_\gamma (-\texttt{r})} \big | g_{{\mathfrak {m}},S}^{(n)}\big (\tfrac{y}{\texttt{r}}\big ) \big |. \nonumber \\ \end{aligned}$$

By direct computations on the derivatives of $h_{{\mathfrak {m}}}(z)$ in (3.4) , we obtain the estimate, for some constant ${\widetilde{C}}>1$,

3.17 $$\begin{aligned} \begin{aligned} | h_{{\mathfrak {m}}}^{(n)}(z)-{\widetilde{Q}}_{\infty }^{(n)}(z)|&\leqslant {\widetilde{C}}^n {\mathfrak {m}}^n \max \Big \{ \Big (\tfrac{\cosh (1-\gamma )}{\cosh (1)}\Big )^{{\mathfrak {m}}}, \Big (\tfrac{\cosh (1)}{\cosh (1+\gamma )}\Big )^{{\mathfrak {m}}} \Big \} \\&\leqslant {\widetilde{C}}^n {\mathfrak {m}}^n e^{-\frac{{\mathfrak {m}}}{2}\gamma }, \quad \forall \,|z| \notin B_\gamma (1); \end{aligned} \end{aligned}$$

here we used the following estimates

$$\begin{aligned} \begin{aligned}&\frac{f^n}{(f+1)^{n+1}} \leqslant {\left\{ \begin{array}{ll} f , &  \quad 0\leqslant f < 1 ,\\ f^{-1}, &  \quad f >1 , \end{array}\right. }, \ n\in {{\mathbb {N}}}_0, \\&\frac{\cosh (1-\gamma )}{\cosh (1)}, \frac{\cosh (1)}{\cosh (1+\gamma )} \leqslant e^{-\gamma /2}, \quad \forall \, 0 \leqslant \gamma \ll 1. \end{aligned} \end{aligned}$$

We deduce that

3.18 $$\begin{aligned} \texttt{J}_{1}\leqslant {\widetilde{C}}^n \frac{{\mathfrak {m}}^{n}}{\texttt{r}^{n}}e^{\frac{{\mathfrak {m}}}{2}\gamma }. \end{aligned}$$

On the other hand, by (3.14) and (3.17), we estimate $\texttt{J}_{2}$ by

3.19 $$\begin{aligned} \texttt {J}_{2} \leqslant \sum _{k=1}^{S} \sup _{|y|\leqslant \texttt {r}-\gamma } \big | h_{{\mathfrak {m}}}^{(2k-1)}\big (\tfrac{y}{\texttt {r}}\big ) - {\widetilde{Q}}_{\infty }^{(2k-1)}\big (\tfrac{y}{\texttt {r}}\big )\big | \leqslant \sum _{k=1}^{S} {\widetilde{C}}^{2k-1} \frac{{\mathfrak {m}}^{2k-1}}{\texttt {r}^{2k-1}} e^{-\frac{{\mathfrak {m}}}{2}\gamma }.\nonumber \\ \end{aligned}$$

Therefore, by (3.13), (3.15), (3.16), (3.18), (3.19), we conclude that the estimate (3.12) holds for any $n\in {{\mathbb {N}}}_0$, with $C:={\widetilde{C}}^{2S-1}$.

We now prove (3.11). We have that

$$\begin{aligned} \begin{aligned}&h_{{\mathfrak {m}}}'(z) = - {\mathfrak {m}}\Big ( \Big (\frac{\cosh (z)}{\cosh (1)}\Big )^{\mathfrak {m}}+1 \Big )^{-2} \Big (\frac{\cosh (z)}{\cosh (1)}\Big )^{\mathfrak {m}}\tanh (z)< 0 \quad \forall \, z\geqslant 0, \\&h_{{\mathfrak {m}}}(0) = \Big ( \Big (\frac{\cosh (0)}{\cosh (1)}\Big )^{\mathfrak {m}}+1 \Big )^{-1} < 1. \end{aligned} \end{aligned}$$

It implies that $\Vert h_{{\mathfrak {m}}} \Vert _{L^{\infty }({{\mathbb {R}}})} \leqslant \Vert {\widetilde{Q}}_{\infty }\Vert _{L^\infty ({{\mathbb {R}}})}= 1$. The estimate $\Vert g_{{\mathfrak {m}},S}(\frac{\cdot }{\texttt{r}})\Vert _{L^{\infty }[-1,1]} \lesssim \Vert {\widetilde{Q}}_{\infty }(\frac{\cdot }{\texttt{r}})\Vert _{L^{\infty }[-1,1]}$ follows by (3.14) with $n=0$ and (3.17) for $|z|\leqslant 1-\gamma$. Therefore, together with (3.13), we deduce (3.11).

The claim for the $L^{p}$-convergence follows by the pointwise convergence of $Q_{{\mathfrak {m}}}(y)$ to $Q_{\infty }(y)$ for any $y\in [-1,1]\setminus \{ \pm \texttt{r}\}$, the estimate $\Vert Q_{\mathfrak {m}}\Vert _{L^\infty ([-1,1])} \lesssim \Vert Q_\infty \Vert _{L^\infty ([-1,1])}\lesssim \texttt{E}^2$ uniformly in ${\mathfrak {m}}\gg 1$ and the integrability of $Q_{\infty }$ in the compact interval $[-1,1]$. $\square$

Among all the possible solutions of (3.1), we look for those that are odd on ${{\mathbb {R}}}$ and satisfy the “Couette condition” $\psi _{{\mathfrak {m}}}'(y)\sim y$ as $|y|\rightarrow \infty$. In particular, we prove that there exists a solution that, on compact sets excluding the singular points $y=\pm \texttt{r}$ of the potential $Q_{\infty }(y)$ in (3.10), approaches uniformly, as ${\mathfrak {m}}\rightarrow \infty$,

3.20 $$\begin{aligned} \psi _{\infty }'(y) := {\left\{ \begin{array}{ll} y -A \,\textrm{sgn}(y) &  \quad y \in K_{\textrm{out}} \subset \subset {{\mathbb {R}}}\setminus [-\texttt{r},\texttt{r}],\\ B \sin (\texttt{E}y) &  \quad y \in K_{\textrm{in}} \subset \subset (-\texttt{r},\texttt{r}), \end{array}\right. } . \end{aligned}$$

Because we are not asking for the continuity of $\psi _{\infty }'(y)$ around $y=\pm \texttt{r}$, the constants $A,B\in {{\mathbb {R}}}$ are actually free and independent one from the other: we fix them in (3.24), in order to prove Proposition 3.4. First, we prove the uniform limit for the stream function $\psi _{{\mathfrak {m}}}(y)$ approaching $\psi _{\infty }(y)$ in (3.20) together with its derivatives on compact sets excluding $y=\pm \texttt{r}$.

#### Lemma 3.3

For any $T\geqslant 1$, $\gamma \in (0,\frac{1}{2})$ and $n\in {{\mathbb {N}}}_0$, we have

3.21 $$\begin{aligned} \begin{aligned}&\sup _{ |y| \leqslant \texttt{r}-\gamma } |\partial _y^n(\psi _{{\mathfrak {m}}}(y) + \tfrac{B}{\texttt{E}} \cos (\texttt{E}y) ) | \rightarrow 0 \quad \text { as } \ \ {\mathfrak {m}}\rightarrow \infty , \\&\sup _{ |y| \in [\texttt{r}+\gamma ,T] } |\partial _y^n(\psi _{{\mathfrak {m}}}(y)-\tfrac{1}{2}(y-A\,\textrm{sgn}(y))^2 ) | \rightarrow 0 \quad \text { as } \ \ {\mathfrak {m}}\rightarrow \infty . \end{aligned} \end{aligned}$$

#### Proof

We start with the first limit in (3.21), with $|y|\leqslant \texttt{r}-\gamma$. Once the claim is proved for $n\geqslant 1$, then the claim for $n=0$ follows by integration. Thus, we start to prove the claim for $n=1,2$. We write the second order equations $u''(y) = Q_{\mathfrak {m}}(y) u(y)$ and $u''(y) = Q_\infty (y) u(y)$ as first order systems, namely

3.22 $$\begin{aligned} \begin{aligned}&\Phi '(y) = A_{\mathfrak {m}}(y) \Phi (y), \quad \Phi '(y) = A_\infty (y) \Phi (y), \\&\Phi := \begin{pmatrix} u \\ u' \end{pmatrix}, \quad A_{\mathfrak {m}}(y) := \begin{pmatrix} 0 &  1\\ Q_{\mathfrak {m}}(y) &  0 \end{pmatrix}, \quad A_\infty (y) := \begin{pmatrix} 0 &  1\\ Q_\infty (y) &  0 \end{pmatrix}. \end{aligned} \end{aligned}$$

Let $\Phi _{{\mathfrak {m}}} (y):= (\psi _{{\mathfrak {m}}}'(y), \psi _{{\mathfrak {m}}}''(y))$, $\Phi _{\infty ,\textrm{in}}(y) :=( B\sin (\texttt{E}y),B\texttt{E}\cos (\texttt{E}y))$ (recall (3.1)–(3.20)) and $\mathcal {F}_{{\mathfrak {m}}} := \Phi _{{\mathfrak {m}}}(y) - \Phi _{\infty ,\textrm{in}}(y)$. We have

$$\begin{aligned} \begin{aligned} \mathcal {F}_{{\mathfrak {m}}}'(y)&= \Phi _{{\mathfrak {m}}}'(y) - \Phi _{\infty ,\textrm{in}}'(y) = A_{\mathfrak {m}}(y) \Phi _{{\mathfrak {m}}}(y) - A_\infty (y) \Phi _{\infty ,\textrm{in}}(y) \\&= A_{{\mathfrak {m}}}(y) \mathcal {F}_{{\mathfrak {m}}}(y) + \big ( A_{\mathfrak {m}}(y) - A_\infty (y) \big ) \Phi _{\infty ,\textrm{in}}(y), \end{aligned} \end{aligned}$$

implying that $\mathcal {F}_{{\mathfrak {m}}}$ solves the Cauchy problem

$$\begin{aligned} \begin{aligned}&\mathcal {F}_{{\mathfrak {m}}}'(y) = A_{\mathfrak {m}}(y)\mathcal {F}_{{\mathfrak {m}}}(y) + \mathcal {R}_{{\mathfrak {m}}}(y), \quad \mathcal {F}_{{\mathfrak {m}}}(0) = \Phi _{{\mathfrak {m}}}(0)-\Phi _{\infty ,\textrm{in}}(0), \\ \text {where} \quad&\mathcal {R}_{{\mathfrak {m}}}(y) := \big ( A_{\mathfrak {m}}(y) - A_\infty (y) \big ) \Phi _{\infty ,\textrm{in}}(y). \end{aligned} \end{aligned}$$

Note that, by (3.1), (3.10), (3.20) and the oddness of $\psi _{{\mathfrak {m}}}'(y)$, we get $\psi _{{\mathfrak {m}}}'''(y) + B\texttt{E}^2 \sin (\texttt{E}y) \rightarrow 0$ as $\texttt{m}\rightarrow +\infty$. By integration, we deduce that $\mathcal {F}_{{\mathfrak {m}}}(0)\rightarrow 0$ as ${\mathfrak {m}}\rightarrow +\infty$. For $|y|\leqslant \texttt{r}-\gamma$, one has that

$$\begin{aligned} \mathcal {F}_{{\mathfrak {m}}}(y) = \mathcal {F}_{{\mathfrak {m}}}(0) + \int _0^y A_{\mathfrak {m}}(z) \mathcal {F}_{{\mathfrak {m}}}(z) \,\textrm{d}{z} + \int _0^y \mathcal {R}_{{\mathfrak {m}}}(z) \,\textrm{d}{z} . \end{aligned}$$

By the definitions of $A_{\mathfrak {m}}$, $A_\infty$ in (3.22) and by Lemma 3.2, one has $\Vert A_{\mathfrak {m}}\Vert _{L^\infty } \leqslant \texttt{E}^2$ and

$$\begin{aligned} \begin{aligned} \Big | \int _0^y \mathcal {R}_{{\mathfrak {m}}}(z) \,\textrm{d}{z} \Big |&\leqslant \int _{-\texttt{r}+\gamma }^{\texttt{r}-\gamma } |A_{\mathfrak {m}}(z) - A_\infty (z) | |\Phi _{\infty ,\textrm{in}}(z)| \,\textrm{d}{z} \\&\leqslant \Vert \Phi _{\infty ,\textrm{in}} \Vert _{L^\infty (B_{\texttt{r}-\gamma }(0))} \Vert Q_{\mathfrak {m}}- Q_\infty \Vert _{L^1(B_{\texttt{r}-\gamma }(0))}. \end{aligned} \end{aligned}$$

Therefore, for any $|y|\leqslant \texttt{r}-\gamma$, one obtains the estimate

$$\begin{aligned} \begin{aligned} |\mathcal {F}_{{\mathfrak {m}}}(y)|&\leqslant |\mathcal {F}_{{\mathfrak {m}}}(0)| + \Vert \Phi _{\infty ,\textrm{in}} \Vert _{L^\infty (B_{\texttt{r}-\gamma }(0))} \Vert Q_{\mathfrak {m}}- Q_\infty \Vert _{L^1(B_{\texttt{r}-\gamma }(0))}\\  &\quad + \texttt{E}^2 \int _0^y |\mathcal {F}_{{\mathfrak {m}}}(z) | \,\textrm{d}{z} . \end{aligned} \end{aligned}$$

By Gronwall inequality, Lemma 3.2 and $\mathcal {F}_{{\mathfrak {m}}}(0)\rightarrow 0$ as ${\mathfrak {m}}\rightarrow 0$, we get

3.23 $$\begin{aligned} \sup _{|y|\leqslant \texttt{r}-\gamma } | \mathcal {F}_{{\mathfrak {m}}}(y)| \leqslant e^{\texttt{E}^2 (\texttt{r}-\gamma ) } \big ( |\mathcal {F}_{{\mathfrak {m}}}(0)| + \texttt{E}^2 \Vert Q_{\mathfrak {m}}- Q_\infty \Vert _{L^1} \big ) \rightarrow 0 \quad \text {as } \ {\mathfrak {m}}\rightarrow 0, \nonumber \\ \end{aligned}$$

which proves the first claim in (3.21) for $n=1,2$. For $n\geqslant 3$, using the same previous notations, we have that, for any $\ell \in {{\mathbb {N}}}$, $\mathcal {F}_{{\mathfrak {m}}}^{(\ell )}(y)$ solves iteratively the Cauchy problem

$$\begin{aligned} \begin{aligned}&(\mathcal {F}_{{\mathfrak {m}}}^{(\ell )})'(y) = A_{\mathfrak {m}}(y)\mathcal {F}_{{\mathfrak {m}}}^{(\ell )}(y) + \mathcal {R}_{{\mathfrak {m}},\ell }(y), \quad \mathcal {F}_{{\mathfrak {m}}}^{(\ell )}(0) = \Phi _{{\mathfrak {m}}}^{(\ell )}(0)-\Phi _{\infty ,\textrm{in}}^{(\ell )}(0), \\&\text {where} \quad \mathcal {R}_{{\mathfrak {m}},\ell }(y) := A_{\mathfrak {m}}'(y) \mathcal {F}_{{\mathfrak {m}}}^{(\ell -1)}(y) + \mathcal {R}_{{\mathfrak {m}},\ell -1}'(y), \quad \mathcal {F}_{{\mathfrak {m}}}^{(0)}(y):= \mathcal {F}_{{\mathfrak {m}}}(y) . \end{aligned} \end{aligned}$$

Then, the similar conclusion in (3.23) holds with the same arguments of before.

Finally, when $\texttt{r}+\gamma \leqslant |y| \leqslant T$, the second claim in (3.21) is proved with the same scheme as before, replacing $\Phi _{\infty ,\textrm{in}}(y)$ with $\Phi _{\infty ,\textrm{out}}(y):=(y-A\,\textrm{sgn}(y),1)$, setting as initial data $\mathcal {F}_{{\mathfrak {m}}}^{(\ell )}(\texttt{r}+\gamma ) = \mathcal {F}_{{\mathfrak {m}}}^{(\ell )}(-(\texttt{r}+\gamma )) = \Phi _{{\mathfrak {m}}}^{(\ell )}(y+\texttt{r})-\Phi _{\infty ,\textrm{out}}^{(\ell )}(\texttt{r}+\gamma )$ for any $\ell \in {{\mathbb {N}}}_0$ and having $\Vert A_{{\mathfrak {m}}} \Vert _{L^\infty ({{\mathbb {R}}}\setminus B_{\texttt{r}+\gamma }(0))}\leqslant \texttt{E}^2 e^{-\texttt{m}\gamma }$ by Lemma 3.2. We therefore omit further details. $\square$

We can now prove the proximity result for the shear flow $(\psi _{{\mathfrak {m}}}'(y),0)$ to the Couette flow (*y*, 0) in the Sobolev regularity $H^1$ (in vorticity space). The main tool is the approximation Lemma 3.3. To this end, we fix, *independently of* ${\mathfrak {m}}\gg 1$, the constants

3.24 $$\begin{aligned} A:= \texttt{r}-\frac{1}{\texttt{E}^2}, \quad B:= \frac{1}{\texttt{E}^2 \cos (\texttt{E}\texttt{r})}{\mathop {=}\limits ^{(1.4)}} \frac{1}{\texttt{E}^2 \sin (\texttt{E}\texttt{r})}, \end{aligned}$$

and the small radius $\gamma \ll \texttt{r}$

3.25 $$\begin{aligned} \gamma = \gamma (\texttt{r})= \texttt{r}^{5}. \end{aligned}$$

#### Proposition 3.4

(Proximity to the Couette flow) There exists ${\overline{{\mathfrak {m}}}} ={\overline{{\mathfrak {m}}}}(\texttt{r})$ being $\gg 1$ large enough such that, for ${\mathfrak {m}}\geqslant {\overline{{\mathfrak {m}}}}$, there exists a stream function $\psi _{{\mathfrak {m}}}(y)$, with being $\psi _{{\mathfrak {m}}}'(y)$ the odd solution of (3.1), that is close to the stream function of the Couette flow $\psi _{\textrm{cou}}(y):=\tfrac{1}{2} y^2$ in the $H^3$-norm, with the estimate

$$\begin{aligned} \Vert \psi _{{\mathfrak {m}}} -\psi _{\textrm{cou}} \Vert _{H^3[-1,1]} \lesssim \sqrt{\texttt{r}}. \end{aligned}$$

#### Proof

By interpolation for Sobolev norms, it is enough to prove estimates for $\Vert \psi _{{\mathfrak {m}}}- \psi _{\textrm{cou}} \Vert _{L^2[-1,1]}$ and for $\Vert \psi _{{\mathfrak {m}}}''' \Vert _{L^2[-1,1]}$. We start with the estimate for $\psi _{{\mathfrak {m}}}-\psi _{\textrm{cou}}$. By parity of the integrand, we split

3.26 $$\begin{aligned} \Vert \psi _{{\mathfrak {m}}} - \psi _{\textrm{cou}} \Vert _{L^2[-1,1]}^2&= 2\int _{\texttt{r}+\gamma }^{1}\big | \psi _{{\mathfrak {m}}}(y) - \tfrac{1}{2} y^2 \big |^2 \,\textrm{d}{y} + 2\int _{B_{\gamma }(\texttt{r})}\big | \psi _{{\mathfrak {m}}}(y) - \tfrac{1}{2} y^2 \big |^2 \,\textrm{d}{y} \nonumber \\&\quad + 2\int _{0}^{\texttt{r}-\gamma }\big | \psi _{{\mathfrak {m}}}(y) - \tfrac{1}{2} y^2 \big |^2 \,\textrm{d}{y} =: I_{0,\textrm{out}} + I_{0,\gamma } + I_{0,\textrm{in}}. \end{aligned}$$

On the compact intervals $[\texttt{r}+\gamma ,1]$ and $[0,\texttt{r}-\gamma ]$, we use the approximations in Lemma 3.3. In particular, by (3.24), (1.4), for any ${\mathfrak {m}}\geqslant {\overline{{\mathfrak {m}}}}$, with ${\overline{{\mathfrak {m}}}} \gg 1$ sufficiently large, we estimate that

3.27 $$\begin{aligned} \tfrac{1}{2} I_{0,\textrm{out}}&\leqslant \int _{\texttt{r}+\gamma }^{1}\big | \psi _{{\mathfrak {m}}}(y) - \tfrac{1}{2}(y -A\,\textrm{sgn}(y) ) \big |^2 \,\textrm{d}{y} + \int _{\texttt{r}+\gamma }^{1}\big | \tfrac{1}{2} (y-A\,\textrm{sgn}(y))^2 - \tfrac{1}{2} y^2 \big |^2 \,\textrm{d}{y} \nonumber \\&= \int _{\texttt{r}+\gamma }^{1}\big | \psi _{{\mathfrak {m}}}(y) - \tfrac{1}{2}(y -A\,\textrm{sgn}(y) ) \big |^2 \,\textrm{d}{y} + A^2\int _{\texttt{r}+\gamma }^{1} \big | |y| - \tfrac{1}{2} A \big |^2 \,\textrm{d}{y} \nonumber \\&\leqslant \int _{\texttt{r}+\gamma }^{1}\big | \psi _{{\mathfrak {m}}}(y) - \tfrac{1}{2}(y -A\,\textrm{sgn}(y) ) \big |^2 \,\textrm{d}{y} + A^2(1-\tfrac{1}{2} A)^2 (1-(\texttt{r}+\gamma )) \nonumber \\&\lesssim \int _{\texttt{r}+\gamma }^{1}\big | \psi _{{\mathfrak {m}}}(y) - \tfrac{1}{2}(y -A\,\textrm{sgn}(y) ) \big |^2 \,\textrm{d}{y} + A^2 \lesssim 2A^2 \lesssim \texttt{r}^2; \end{aligned}$$

3.28 $$\begin{aligned} \tfrac{1}{2} I_{0,\textrm{in}}&\leqslant \int _{0}^{\texttt{r}-\gamma }\big | \psi _{{\mathfrak {m}}}(y) + \tfrac{B}{\texttt{E}} \cos (\texttt{E}y) \big |^2 \,\textrm{d}{y} + \int _{0}^{\texttt{r}-\gamma } \big | \tfrac{B}{\texttt{E}}\cos (\texttt{E}y) + \tfrac{1}{2} y^2 \big |^2 \,\textrm{d}{y} \nonumber \\&\lesssim \int _{0}^{\texttt{r}-\gamma }\big | \psi _{{\mathfrak {m}}}(y) + \tfrac{B}{\texttt{E}} \cos (\texttt{E}y) \big |^2 \,\textrm{d}{y} + \max \{ \tfrac{B}{\texttt{E}},(\texttt{r}-\gamma )^2 \}^2 (\texttt{r}-\gamma ) \nonumber \\&\lesssim \int _{0}^{\texttt{r}-\gamma }\big | \psi _{{\mathfrak {m}}}(y) + \tfrac{B}{\texttt{E}} \cos (\texttt{E}y) \big |^2 \,\textrm{d}{y} + (\texttt{r}-\gamma )^5 \lesssim 2\texttt{r}^5. \end{aligned}$$

On the compact neighbourhood $\{ ||y|-\texttt{r}| \leqslant \gamma \}$, the approximation with the singular case ${\mathfrak {m}}\rightarrow \infty$ fails. However, being $Q_{{\mathfrak {m}}}(y)$ uniformly bounded by $\texttt{E}^2$ for any ${\mathfrak {m}}\in {{\mathbb {N}}}$ (see Lemma 3.2), all the solutions of (3.1) are uniformly bounded with respect to ${\mathfrak {m}}\in {{\mathbb {N}}}$ on $[-1,1]$, as well as $\psi _{{\mathfrak {m}}}(y)$ by integration. Therefore, by (3.26) and (3.25), we have the estimate

3.29 $$\begin{aligned} \tfrac{1}{2} I_{0,\gamma } \lesssim \max \big \{ \Vert \psi _{{\mathfrak {m}}}\Vert _{L^\infty (B_{\gamma }(\texttt{r}))}, (\texttt{r}+\gamma )^2 \big \}^2 \gamma \lesssim \texttt{r}^{5} . \end{aligned}$$

By (3.26), (3.27), (3.28), (3.29), (3.24), we conclude that $\Vert \psi _{{\mathfrak {m}}} - \psi _{\textrm{cou}} \Vert _{L^2[-1,1]} \lesssim \texttt{r}$.

We move now to estimate the $L^2$-norm of $\psi _{{\mathfrak {m}}}'''(y)$. Similarly as in (3.26), we split

3.30 $$\begin{aligned} \Vert \psi _{{\mathfrak {m}}}''' \Vert _{L^2[-1,1]}^2&\leqslant 2 \int _{\texttt{r}+\gamma }^{1}|\psi _{{\mathfrak {m}}}'''(y) |^2 \,\textrm{d}{y} + 2 \int _{B_{\gamma }(\texttt{r})}|\psi _{{\mathfrak {m}}}'''(y) |^2 \,\textrm{d}{y} + 2 \int _{0}^{\texttt{r}-\gamma }|\psi _{{\mathfrak {m}}}'''(y) |^2 \,\textrm{d}{y} \nonumber \\&=: I_{3,\textrm{out}} + I_{3,\gamma } + I_{3,\textrm{in}} . \end{aligned}$$

As before, we use the approximation Lemma 3.3 on the compact intervals $[\texttt{r}+\gamma ,1]$ and $[0,\texttt{r}-\gamma ]$, with the same choice of the constants *A*, *B* as in (3.24) and for ${\mathfrak {m}}\geqslant {\overline{{\mathfrak {m}}}}$, with a possibly larger ${\overline{{\mathfrak {m}}}}= {\overline{{\mathfrak {m}}}}(\texttt{r})\gg 1$:

3.31 $$\begin{aligned} \tfrac{1}{2} I_{3,\textrm{in}}&\leqslant \int _{0}^{\texttt{r}-\gamma } | \psi _{{\mathfrak {m}}}'''(y) + B\,\texttt{E}^2 \sin (\texttt{E}y) |^2 \,\textrm{d}{y} + B^2 \,\texttt{E}^4 \int _{0}^{\texttt{r}-\gamma } | \sin (\texttt{E}y)|^2 \,\textrm{d}{y}\nonumber \\&\leqslant \int _{0}^{\texttt{r}-\gamma } | \psi _{{\mathfrak {m}}}'''(y) + B\,\texttt{E}^2 \sin (\texttt{E}y) |^2 \,\textrm{d}{y} + B^2 \,\texttt{E}^4 (\texttt{r}-\gamma ) \nonumber \\&\lesssim \int _{0}^{\texttt{r}-\gamma } | \psi _{{\mathfrak {m}}}'''(y) + B\,\texttt{E}^2 \sin (\texttt{E}y) |^2 \,\textrm{d}{y} + \texttt{r}\lesssim 2\texttt{r}; \end{aligned}$$

3.32 $$\begin{aligned} \tfrac{1}{2} I_{3,\textrm{out}}&= \int _{\texttt{r}+\gamma }^{1}| \psi _{{\mathfrak {m}}}'''(y) |^2\,\textrm{d}{y} \lesssim 2\texttt{r}. \end{aligned}$$

To estimate finally $I_{3,\gamma }$, we recall that $\psi _{{\mathfrak {m}}}'(y)$ solves (3.1) and, as for (3.29), that it is uniformly bounded with respect to ${\mathfrak {m}}\in {{\mathbb {N}}}$ on the whole interval $[-1,1]$. Therefore, by (3.30), (3.25), (1.4) and Lemma 3.2, we get

3.33 $$\begin{aligned} \tfrac{1}{2} I_{3,\gamma }&= \int _{B_{\gamma }(\texttt{r})} | Q_{{\mathfrak {m}}}(y) |^2 |\psi _{{\mathfrak {m}}}'(y)|^2 \,\textrm{d}{y} \leqslant \texttt{E}^4 \int _{B_{\gamma }(\texttt{r})} |\psi _{{\mathfrak {m}}}'(y)|^2 \,\textrm{d}{y} \lesssim \texttt{E}^4 \gamma \lesssim \texttt{r}. \nonumber \\ \end{aligned}$$

By (3.30), (3.31), (3.32), (3.33), we conclude that $\Vert \psi _{{\mathfrak {m}}}''' \Vert _{L^2[-1,1]}\lesssim \sqrt{\texttt{r}}$. This concludes the proof of the proposition. $\square$

### The Existence of the Local Nonlinearities

The goal of this section is to determine which nonlinear ODE is locally solved by the stream function $\psi _{{\mathfrak {m}}}(y)$, starting from the linear ODE (3.1). By Lemma 3.3, in the limit ${\mathfrak {m}}\rightarrow \infty$, the stream function $\psi _{{\mathfrak {m}}}(y)$ converges locally on compact sets excluding the singular values $y=\pm \texttt{r}$ to a limit function $\psi _{\infty }(y)$, given in (3.20), solving locally the second-order semilinear ODE

3.34 $$\begin{aligned} \psi _{\infty }''(y) = F_\infty (\psi _{\infty }(y)) := {\left\{ \begin{array}{ll} 1 &  \quad y\in K_{\textrm{out}} \subset \subset {{\mathbb {R}}}\setminus [-\texttt{r},\texttt{r}] , \\ -\texttt{E}^2 \psi _{\infty }(y) &  \quad y \in K_{\textrm{in}} \subset \subset (-\texttt{r},\texttt{r}) . \end{array}\right. }\nonumber \\ \end{aligned}$$

We want to show that the even function $\psi _{{\mathfrak {m}}}(y)$ solves an ODE similar to (3.34) but on the whole interval $[-1,1]$, including the neighbourhoods of the singularities $y=\pm \texttt{r}$, which are smoothed out thanks to the analyticity of the potential $Q_{{\mathfrak {m}}}(y)$ in (3.1). Outside the neighbourhoods of these singular values, the nonlinearity is expected to be a slight modification of the one in (3.34) and, morally speaking, to behave locally as a single function and globally as multivalued function. The construction of the local nonlinearities is carried out in Theorem 3.7. To this end, we need a couple of preliminary results. First, as a corollary of Lemma 3.3, we deduce monotonicity properties for the stream function $\psi _{{\mathfrak {m}}}(y)$.

#### Lemma 3.5

There exists ${\overline{{\mathfrak {m}}}} = {\overline{{\mathfrak {m}}}}(\texttt{r})\gg 1$, possibly larger than the one fixed in Proposition 3.4, such that, for any ${\mathfrak {m}}\geqslant {\overline{{\mathfrak {m}}}}$, the following hold:

(i) $\psi _{{\mathfrak {m}}}(|y|)$ is strictly monotone for $|y| \geqslant \texttt{r}+\gamma$;
(ii) $\psi _{{\mathfrak {m}}}(|y|)$ is strictly monotone for $|y| \in B_\gamma (\texttt{r})$;
(iii) $\psi _{{\mathfrak {m}}}(y)$ has $2\kappa _0+1$ critical points $0=\texttt{y}_{0,{\mathfrak {m}}}< |\pm \texttt{y}_{1,{\mathfrak {m}}}|< ...< |\pm \texttt{y}_{\kappa _0,{\mathfrak {m}}}| < \texttt{r}-\gamma$ and no saddle points when $|y|\leqslant \texttt{r}-\gamma$. In particular, $\psi _{{\mathfrak {m}}}''$ does not vanish at these critical points.

#### Proof

Proof of (*i*). By Lemma 3.3 and (3.24), (3.25), we have, for any $|y|\geqslant \texttt{r}+\gamma$,

$$\begin{aligned} \begin{aligned} | \psi _{{\mathfrak {m}}}'(y) |&\geqslant |y -A\,\textrm{sgn}(y)| - | \psi _{{\mathfrak {m}}}'(y) - (y-A\,\textrm{sgn}(y)) |\\&\geqslant \gamma + \tfrac{1}{\texttt{E}^2} - | \psi _{{\mathfrak {m}}}'(y) - (y-A\,\textrm{sgn}(y)) | \geqslant \tfrac{1}{2}\big ( \gamma + \tfrac{1}{\texttt{E}^2} \big ) >0, \end{aligned} \end{aligned}$$

having ${\mathfrak {m}}\geqslant {\overline{{\mathfrak {m}}}}$. This proves item (*i*) and ensures that $\psi _{{\mathfrak {m}}}(y)$ is invertible in this region.

Proof of (*ii*). By parity, we prove just that $\psi _{{\mathfrak {m}}}(y)$ is strictly monotone for $|y-\texttt{r}|\leqslant \gamma$. First, by Lemma 3.3 and (3.24), (3.25), (1.4), we have the pointwise estimate, for ${\mathfrak {m}}\geqslant {\overline{{\mathfrak {m}}}}$, with ${\overline{{\mathfrak {m}}}}\gg 1$ large enough, and up to subsequences,

$$\begin{aligned} \begin{aligned}&\psi _{{\mathfrak {m}}}'(\texttt{r}-\gamma ) = B \sin (\texttt{E}(\texttt{r}-\gamma )) + \big ( \psi _{{\mathfrak {m}}}'(\texttt{r}-\gamma ) - B\sin (\texttt{E}(\texttt{r}-\gamma )) \big )\\&\quad = \frac{1}{\texttt{E}^2} \Big ( 1 + \frac{-\texttt{E}\gamma }{\sin (\texttt{E}\texttt{r})} \frac{\sin (\texttt{E}(\texttt{r}-\gamma ))-\sin (\texttt{E}\texttt{r})}{-\texttt{E}\gamma } \Big ) + \big ( \psi _{{\mathfrak {m}}}'(\texttt{r}-\gamma ) - B\sin (\texttt{E}(\texttt{r}-\gamma )) \big ) \\&\quad \geqslant \frac{1}{\texttt{E}^2 }\Big ( 1 - \frac{\texttt{E}\gamma }{|\sin (\texttt{E}\texttt{r}) |} \Big ) - \big | \psi _{{\mathfrak {m}}}'(\texttt{r}-\gamma ) - B\sin (\texttt{E}(\texttt{r}-\gamma )) \big | \\&\quad \geqslant \frac{1}{2 \texttt{E}^2 }\big ( 1 - \sqrt{2}\texttt{E}\gamma \big ) \gtrsim \texttt{r}^2>0 \end{aligned} \end{aligned}$$

and, similarly,

3.35 $$\begin{aligned} \begin{aligned} \psi _{{\mathfrak {m}}}''(\texttt{r}-\gamma ) \geqslant \frac{1}{2 \texttt{E}}\big ( 1 - \sqrt{2}\texttt{E}\gamma \big ) \gtrsim \texttt{r} > 0 \end{aligned} \end{aligned}$$

for $\texttt{r} \ll 0$ small enough, since $\texttt{r} \simeq \frac{1}{{\texttt{E}}}$ and $\gamma = \texttt{r}^5$. Therefore, the claim that $\psi _{{\mathfrak {m}}}'(y) >0$ for $|y-\texttt{r}|\leqslant \gamma$ follows if we show that $\psi _{{\mathfrak {m}}}''(y)>0$ in the same interval. By the mean value Theorem, (3.1), (3.25), (1.4) and Lemma 3.2, we have, for any $y\in [\texttt{r}-\gamma ,\texttt{r}+\gamma ]$,

3.36 $$\begin{aligned} \begin{aligned} | \psi _{{\mathfrak {m}}}'' (y)&- \psi _{{\mathfrak {m}}}''(\texttt{r}-\gamma )| \leqslant 2\gamma \Vert \psi _{{\mathfrak {m}}}''' \Vert _{L^\infty [-1,1]} \\&\leqslant 2\gamma \Vert Q_{{\mathfrak {m}}} \Vert _{L^\infty [-1,1]} \Vert \psi _{{\mathfrak {m}}}' \Vert _{L^\infty [-1,1]} \lesssim \texttt{E}^2 \gamma \lesssim \texttt{r}^{3}. \end{aligned} \end{aligned}$$

Therefore, for $\texttt{r}\ll 1$, by (3.36), (3.35) we deduce

$$\begin{aligned} \psi _{{\mathfrak {m}}}''(y) \geqslant \psi _{{\mathfrak {m}}}''(\texttt{r}-\gamma ) - | \psi _{{\mathfrak {m}}}'' (y) - \psi _{{\mathfrak {m}}}''(\texttt{r}-\gamma )| \gtrsim \texttt{r} - O(\texttt{r}^3) \gtrsim \texttt{r} > 0. \end{aligned}$$

This concludes the proof of item (*ii*).

Proof of (*iii*).Under the constrain (1.4), let $\texttt{y}_{j,\infty }=\frac{j\pi }{\texttt{E}}$, $j=0,\pm 1, ...,\pm \kappa _0$ be the $2\kappa _0 + 1$ zeroes of $\psi _{\infty }'(y):=B \sin (\texttt{E}y)$ in $[-\texttt{r},\texttt{r}]$, and therefore critical points for $\psi _{\infty }(y)=\frac{B}{\texttt{E}} \cos (\texttt{E}y)$. It is also trivial, by (3.24), (1.4), that $|\psi _{\infty }''(\texttt{y}_{j,\infty })|= \frac{\sqrt{2}}{\texttt{E}}>0$ and that $|\texttt{y}_{\pm \kappa _0,\infty }|= \frac{\kappa _0 \pi }{\texttt{E}}< \texttt{r}$. By Lemma 3.3 and (3.25), for ${\mathfrak {m}}\geqslant {\overline{{\mathfrak {m}}}}$, with ${\overline{{\mathfrak {m}}}}\gg 1$ large enough, we have that $\psi _{{\mathfrak {m}}}'(y)$ has $2\kappa _0+1$ zeroes on $[-(\texttt{r}-\gamma ),\texttt{r}-\gamma ]$, denoted by $\texttt{y}_{j,{\mathfrak {m}}}$ for $j=0,\pm 1, ..., \pm {\mathfrak {m}}$, each one sufficiently close to $\texttt{y}_{j,\infty }$ and satisfying $|\psi _{{\mathfrak {m}}}''(\texttt{y}_{j,{\mathfrak {m}}})|\geqslant \frac{\sqrt{2}}{2 \texttt{E}}$. Moreover, by the parity of $\psi _{{\mathfrak {m}}}(y)$, we deduce that $\texttt{y}_{0,{\mathfrak {m}}}=0$ and that $\texttt{y}_{-p,{\mathfrak {m}}}=-\texttt{y}_{p,{\mathfrak {m}}}$ for any $p=1,...,\kappa _0$. This proves the claim in item (*iii*) and concludes the proof of the lemma. $\square$

The following result follows from Lemma 3.1 and it is a key tool to for proving the claimed regularity properties in Theorem 3.7:

#### Lemma 3.6

Let ${\overline{{\mathfrak {m}}}} \gg 1$ be fixed as in Lemma 3.5. Let $\texttt{y}_{p,{\mathfrak {m}}}$ be a critical point for $\psi _{{\mathfrak {m}}}(y)$, for a stripe index $p=1,...,\kappa _0$. For ${\mathfrak {m}}\geqslant {\overline{{\mathfrak {m}}}}$, the following holds

$$\begin{aligned} \begin{aligned}&\psi _{{\mathfrak {m}}}^{(2n-1)}(\texttt{y}_{p,{\mathfrak {m}}}) = 0 , \quad \forall \, n=1,...,S+1. \end{aligned} \end{aligned}$$

As a consequence, we have the expansion, for $|\delta |$ small enough,

$$\begin{aligned} \psi _{{\mathfrak {m}}}(\texttt{y}_{p,{\mathfrak {m}}}+\delta ) = \sum _{n=0}^{S+1} \frac{\psi _{{\mathfrak {m}}}^{(2n)}(\texttt{y}_{p,{\mathfrak {m}}})}{(2n)!} \delta ^{2n} + \frac{\psi _{{\mathfrak {m}}}^{(2S+3)}(\texttt{y}_{p,{\mathfrak {m}}})}{(2S+3)!} \delta ^{2S+3} + o(|\delta |^{2(S+2)}). \end{aligned}$$

#### Proof

The expansion is a direct consequence of Lemma 3.1 together with iterative derivatives of (3.1). Indeed, by Lemma 3.1-(*iii*), we have $\psi _{{\mathfrak {m}}}'(\texttt{y}_{p,{\mathfrak {m}}})=0$, whereas, for $n\geqslant 2$, we have, by the Leibniz rule,

$$\begin{aligned} \psi _{{\mathfrak {m}}}^{(2n-1)}(y)=\partial _y^{2(n-2)}\psi _{{\mathfrak {m}}}'''(y)= \sum _{j=0}^{2(n-2)}\left( {\begin{array}{c}2(n-2)\\ j\end{array}}\right) Q_{{\mathfrak {m}}}^{(j)}(y) \psi _{{\mathfrak {m}}}^{(2n-3-k)}(y). \end{aligned}$$

The claim $\psi _{{\mathfrak {m}}}^{(2n-1)}(\texttt{y}_{p,{\mathfrak {m}}}) =0$, for $n=1,...,S+1$, follows consequently by an induction argument. $\square$

We are now ready to prove the main result of this section. We introduce the following notation for the left and the right neighbourhood of a given point, respectively: for any $r > 0$, we define

$$\begin{aligned} B_{r}^{-}(\texttt{y}) := \{ y\in {{\mathbb {R}}}\, : \, y\in (\texttt{y}-r, \texttt{y}) \}, \quad B_{r}^{+}(\texttt{y}) := \{ y\in {{\mathbb {R}}}\, : \, y \in (\texttt{y},\texttt{y}+r) \}. \end{aligned}$$

For any $S \in {{\mathbb {N}}}$, we denote by $\mathcal {C}^S_0({{\mathbb {R}}})$ the space of $\mathcal {C}^S$ functions $f : {{\mathbb {R}}}\rightarrow {{\mathbb {R}}}$ with compact support. In order to state the next theorem, we also recall the definition of the interval ${\texttt{I}}_p = \{ y \in {{\mathbb {R}}}: \texttt{y}_{p,{\mathfrak {m}}} \leqslant |y| \leqslant \texttt{y}_{p+1,{\mathfrak {m}}}\}$ given in (1.7).

#### Theorem 3.7

(Local nonlinearities) Let $S\in {{\mathbb {N}}}$ and let ${\mathfrak {m}}\geqslant {\overline{{\mathfrak {m}}}}$, with ${\overline{{\mathfrak {m}}}} \gg 1$ fixed as in Proposition 3.4. For any $p=0,1,...,\kappa _0$, there exists a nonlinear function $F_{p,{\mathfrak {m}}}\in \mathcal {C}_{0}^{S+1}({{\mathbb {R}}})$, $\psi \rightarrow F_{p,{\mathfrak {m}}}(\psi )$, such that $\psi _{{\mathfrak {m}}}(y)$ solves the nonlinear ODE

3.37 $$\begin{aligned} \psi _{\mathfrak {m}}''(y) = F_{p,{\mathfrak {m}}}(\psi _{\mathfrak {m}}(y)), \quad y\in \texttt{I}_{p}. \end{aligned}$$

In particular, the derivative of $F_{p,{\mathfrak {m}}}$ evaluated at $\psi =\psi _{{\mathfrak {m}}}(y)$ satisfies, for any $y\in {{\mathbb {R}}}$,

3.38 $$\begin{aligned} (\partial _\psi F_{p,{\mathfrak {m}}})(\psi _{\mathfrak {m}}(y))=Q_{\mathfrak {m}}(y), \quad \forall \,p=0,1,...,\kappa _0. \end{aligned}$$

We have $\mathcal {C}^{S+1}$-continuity at $\psi =\psi _{{\mathfrak {m}}}(y)$ at the critical points $y=\pm \texttt{y}_{p,{\mathfrak {m}}}$, $p=1,...,\kappa _0$, meaning that, for any $n=0,1,...,S+1$,

3.39 $$\begin{aligned} \lim _{|y|\rightarrow \texttt{y}_{p,{\mathfrak {m}}}^-} \partial _{y}^{n} (F_{p-1,{\mathfrak {m}}}(\psi _{{\mathfrak {m}}}(y)) )= \lim _{|y|\rightarrow \texttt{y}_{p,{\mathfrak {m}}}^+}\partial _{y}^{n}( F_{p,{\mathfrak {m}}}(\psi _{{\mathfrak {m}}}(y)) ) = \psi _{{\mathfrak {m}}}^{(n+2)}(\texttt{y}_{p,{\mathfrak {m}}}) .\nonumber \\ \end{aligned}$$

#### Proof

Each function $F_{p,\texttt{m}}(\psi )$ is constructed on the interval of monotonicity for the stream function $\psi _{{\mathfrak {m}}}(y)$ by solving Cauchy problems that lead to (3.38). By Lemma 3.6, the behaviour around the critical points $\pm \texttt{y}_{1,{\mathfrak {m}}},...,\pm \texttt{y}_{\kappa _0,{\mathfrak {m}}}$ both for the stream function $\psi _{{\mathfrak {m}}}(y)$ and the potential $Q_{{\mathfrak {m}}}(y)$ will determine the regularity of the functions. The construction is carried out in several steps.

**Step 1) Behaviour of** $\psi _{{\mathfrak {m}}}(y)$ **around the critical points.** It is convenient to rewrite both the stream function $\psi _{{\mathfrak {m}}}(y)$ and the potential $Q_{{\mathfrak {m}}}(y)$ as quadratic functions with finite regularity locally around each critical point of $\psi _{{\mathfrak {m}}}(y)$.

We start with $\texttt{y}_{0,{\mathfrak {m}}}=0$. Since both $Q_{{\mathfrak {m}}}(y)$ and $\psi _{{\mathfrak {m}}}(y)$ are even in *y*, we write

$$\begin{aligned} Q_{{\mathfrak {m}}}(y) = K_{0,{\mathfrak {m}}}(y^2) , \quad \psi _{{\mathfrak {m}}}(y) = G_{0,{\mathfrak {m}}}(y^2), \quad |y| < \texttt{y}_{1,{\mathfrak {m}}}:=\texttt{r}_{0,+}. \end{aligned}$$

By Lemma 3.5-(*iii*) we have $\psi _{{\mathfrak {m}}}''(0)\ne 0$ and $|G_{0,{\mathfrak {m}}}'(y^2)| = \big |\frac{\psi _{{\mathfrak {m}}}'(y)}{2y}\big |>0$ for any $y\in \texttt{I}_{0}$. Therefore, $G_{0,{\mathfrak {m}}}(z)$ is invertible for $|z|<\sqrt{\texttt{y}_{1,{\mathfrak {m}}}}$. In the same region, because both $Q_{{\mathfrak {m}}}(y)$ and $\psi _{{\mathfrak {m}}}(y)$ are analytic and even, we have that $K_{0,{\mathfrak {m}}}(z)$ and $G_{0,{\mathfrak {m}}}(z)$ are in $\mathcal {C}^{\infty }$, as well as $G_{0,{\mathfrak {m}}}^{-1}$.

We move now around $|y|=\texttt{y}_{p,{\mathfrak {m}}}$, $p=1,..,\kappa _0-1$. By Lemma 3.1, we deduce that we can write, for $||y|-\texttt{y}_{p,{\mathfrak {m}}}| < \texttt{r}_{p,\pm }$, with $\texttt{r}_{p,\pm }:=|\texttt{y}_{p,{\mathfrak {m}}} - \texttt{y}_{p\pm 1,{\mathfrak {m}}}|$,

$$\begin{aligned} Q_{{\mathfrak {m}}}(y) = K_{p,{\mathfrak {m}},\pm }\big ((|y|-\texttt{y}_{p,{\mathfrak {m}}})^2\big ) , \quad K_{p,{\mathfrak {m}},\pm } \in \mathcal {C}^{S}(B_{\sqrt{\texttt{r}_{p,\pm }}}(0)) . \end{aligned}$$

Similarly, by Lemma 3.6, we write, for $||y|-\texttt{y}_{p,{\mathfrak {m}}}| <\texttt{r}_{p,\pm }$,

$$\begin{aligned} \psi _{{\mathfrak {m}}}(y) = G_{p,{\mathfrak {m}},\pm }\big ((|y|-\texttt{y}_{p,{\mathfrak {m}}})^2\big ) , \quad G_{p,{\mathfrak {m}},\pm } \in \mathcal {C}^{S+1}(B_{\sqrt{\texttt{r}_{p,\pm }}}(0)). \end{aligned}$$

By Lemma 3.5-(*iii*), we have

$$\begin{aligned} \begin{aligned}&\big |G_{p,{\mathfrak {m}},-}'\big ( (|y|-\texttt{y}_{p,{\mathfrak {m}}})^2 \big )\big | = \Big | \frac{\psi _{{\mathfrak {m}}}'\big ( |y|-\texttt{y}_{p,{\mathfrak {m}}} \big )}{2(|y|-\texttt{y}_{p,{\mathfrak {m}}})} \Big |>0 \quad \forall \, y \in \texttt{I}_{p-1}, \\&\big |G_{p,{\mathfrak {m}},+}'\big ( (|y|-\texttt{y}_{p,{\mathfrak {m}}})^2 \big )\big | = \Big | \frac{\psi _{{\mathfrak {m}}}'\big ( |y|-\texttt{y}_{p,{\mathfrak {m}}} \big )}{2(|y|-\texttt{y}_{p,{\mathfrak {m}}})} \Big | >0 \quad \forall \, y \in \texttt{I}_{p}, \\&\lim _{|y|\rightarrow \texttt{y}_{p,{\mathfrak {m}}}^{\pm }} G_{p,{\mathfrak {m}},\pm }'\big ( (|y|-\texttt{y}_{p,{\mathfrak {m}}})^2 \big ) = \tfrac{1}{2} \psi _{{\mathfrak {m}}}''(\texttt{y}_{p,{\mathfrak {m}}}) \ne 0. \end{aligned} \end{aligned}$$

Therefore, $G_{p,{\mathfrak {m}},-}(z)$ is invertible for $|z|<\sqrt{\texttt{r}_{p,-}}$ and $G_{p,{\mathfrak {m}},+}(z)$ is invertible for $|z|<\sqrt{\texttt{r}_{p,+}}$, with inverses $G_{p,{\mathfrak {m}},\pm }^{-1}$ being in $\mathcal {C}^{S+1}$ in the respective regions.

Finally, we consider the critical points $|y|=\texttt{y}_{\kappa _0,{\mathfrak {m}}}$. With the same previous arguments, we have, for $\texttt{y}_{\kappa _0-1,{\mathfrak {m}}}< |y| \leqslant \texttt{y}_{\kappa _0,{\mathfrak {m}}}$, with $\texttt{r}_{\kappa _0,-}:=\texttt{y}_{\kappa _0,{\mathfrak {m}}}-\texttt{y}_{\kappa _0-1,{\mathfrak {m}}}$

$$\begin{aligned} \begin{aligned}&Q_{{\mathfrak {m}}}(y) = K_{\kappa _0,{\mathfrak {m}},-} \big ( (|y|-\texttt{y}_{\kappa _0,{\mathfrak {m}}})^2 \big ), \quad K_{\kappa _0,\pm ,-} \in \mathcal {C}^{S}(B_{\sqrt{\texttt{r}_{\kappa _0,-}}}(0)), \\&\psi _{{\mathfrak {m}}}(y) = G_{\kappa _0,{\mathfrak {m}},-} \big ( (|y|-\texttt{y}_{\kappa _0,{\mathfrak {m}}})^2 \big ), \quad Q_{\kappa _0,\pm ,-} \in \mathcal {C}^{S+1}(B_{\sqrt{\texttt{r}_{\kappa _0,-}}}(0)), \end{aligned} \end{aligned}$$

and, for $\texttt{y}_{\kappa _0,{\mathfrak {m}}}\leqslant |y|\leqslant 1$, with $\texttt{r}_{\kappa _0,+}:=1-\texttt{y}_{\kappa _0,{\mathfrak {m}}}$,

$$\begin{aligned} \begin{aligned}&Q_{{\mathfrak {m}}}(y) = K_{\kappa _0,{\mathfrak {m}},+} \big ( (|y|-\texttt{y}_{\kappa _0,{\mathfrak {m}}})^2 \big ), \quad K_{\kappa _0,\pm ,+} \in \mathcal {C}^{S}(B_{\sqrt{\texttt{r}_{\kappa _0,+}}}(0)), \\&\psi _{{\mathfrak {m}}}(y) = G_{\kappa _0,{\mathfrak {m}},+} \big ( (|y|-\texttt{y}_{\kappa _0,{\mathfrak {m}}})^2 \big ), \quad Q_{\kappa _0,\pm ,+} \in \mathcal {C}^{S+1}(B_{\sqrt{\texttt{r}_{\kappa _0,+}}}(0)). \end{aligned} \end{aligned}$$

Also in this case, by Lemma 3.5, $G_{\kappa _0,{\mathfrak {m}},-}(z)$ is invertible for $|z|<\sqrt{\texttt{r}_{\kappa _0,-}}$ and $G_{\kappa _0,{\mathfrak {m}},+}(z)$ is invertible for $|z|<\sqrt{\texttt{r}_{\kappa _0,+}}$, with inverses $G_{\kappa _0,{\mathfrak {m}},\pm }^{-1}$ being in $\mathcal {C}^{S+1}$ in the respective regions.

**Step 2) Existence and smoothness of** $F_{p,{\mathfrak {m}}}(\psi )$. We start with the stripe indexes $p=0,1,..., \kappa _0-1$. We look for $F_{p,{\mathfrak {m}}}(\psi )$ of the form $F_{p,{\mathfrak {m}}}(\psi )=-\texttt{E}^2 \psi + Z_{p,{\mathfrak {m}}}(\psi )$. Let $0<\gamma _{0}\ll \texttt{r}_{0,+}$ and $0<\gamma _{p}\ll \min \{\texttt{r}_{p,-},\texttt{r}_{p,+}\}$, $p\geqslant 1$, small enough. First, we define $Z_{p,{\mathfrak {m}},+}(\psi )$ as the solution of the Cauchy problem

$$\begin{aligned} {\left\{ \begin{array}{ll} \partial _\psi Z_{p,{\mathfrak {m}},+}(\psi ) = K_{p,{\mathfrak {m}},+}(G_{p,{\mathfrak {m}},+}^{-1}(\psi ))+\texttt{E}^2, \quad \psi \in \psi _{{\mathfrak {m}}}(B_{\texttt{r}_{p,+}-\gamma _{p+1}}^{+}(\texttt{y}_{p,{\mathfrak {m}}})), \\ Z_{p,{\mathfrak {m}},+} (\psi _{{\mathfrak {m}}}(\texttt{y}_{p,{\mathfrak {m}}})) = \psi _{{\mathfrak {m}}}''(\texttt{y}_{p,{\mathfrak {m}}}) + \texttt{E}^2 \psi _{{\mathfrak {m}}}(\texttt{y}_{p,{\mathfrak {m}}}). \end{array}\right. } \end{aligned}$$

Then, we define $Z_{p,{\mathfrak {m}},-}(\psi )$ as the solution of the Cauchy problem

$$\begin{aligned} {\left\{ \begin{array}{ll} \partial _\psi Z_{p,{\mathfrak {m}},-}(\psi ) = K_{p+1,{\mathfrak {m}},-}(G_{p+1,{\mathfrak {m}},-}^{-1}(\psi ))+\texttt{E}^2, \quad \psi \in \psi _{{\mathfrak {m}}}(B_{\texttt{r}_{p,-}-\gamma _{p}}^{-}(\texttt{y}_{p+1,{\mathfrak {m}}})), \\ Z_{p,{\mathfrak {m}},-} (\psi _{{\mathfrak {m}}}(\texttt{y}_{p+1,{\mathfrak {m}}})) = \psi _{{\mathfrak {m}}}''(\texttt{y}_{p+1,{\mathfrak {m}}}) + \texttt{E}^2 \psi _{{\mathfrak {m}}}(\texttt{y}_{p+1,{\mathfrak {m}}}). \end{array}\right. } \end{aligned}$$

By Step 1 and the boundedness of the vector fields following from Lemma 3.2, both problems are well defined, with $Z_{p,{\mathfrak {m}},+}\in \mathcal {C}^{S+1}\big ( \psi _{{\mathfrak {m}}}(B_{\texttt{r}_{p,+}-\gamma _{p+1}}^{+}(\texttt{y}_{p,{\mathfrak {m}}})) \big )$ and $Z_{p,{\mathfrak {m}},-}\in \mathcal {C}^{S+1}\big ( \psi _{{\mathfrak {m}}}(B_{\texttt{r}_{p+1,-}-\gamma _{p}}^{-}(\texttt{y}_{p+1,{\mathfrak {m}}})) \big )$. We claim that, when $\psi \in \psi _{{\mathfrak {m}}}(B_{\texttt{r}_{p,+}-\gamma _{p+1}}^{+}(\texttt{y}_{p,{\mathfrak {m}}})) \cap \psi _{{\mathfrak {m}}}(B_{\texttt{r}_{p+1,-}-\gamma _{p}}^{-}(\texttt{y}_{p+1,{\mathfrak {m}}}))$, then $Z_{p,{\mathfrak {m}},+}(\psi )= Z_{p,{\mathfrak {m}},-}(\psi )$. Indeed, using the respective initial values, the two functions satisfy, for $|y| \in B_{\texttt{r}_{p,+}-\gamma _{p+1}}^{+}(\texttt{y}_{p,{\mathfrak {m}}}) \cap B_{\texttt{r}_{p+1,-}-\gamma _{p}}^{-}(\texttt{y}_{p+1,{\mathfrak {m}}})$,

$$\begin{aligned} (\partial _\psi Z_{p,{\mathfrak {m}},+})(\psi _{\mathfrak {m}}(y)) = Q_{{\mathfrak {m}}} (y) +\texttt{E}^2= (\partial _\psi Z_{p,{\mathfrak {m}},-} (\psi _{{\mathfrak {m}}}(y)) ) . \end{aligned}$$

By uniqueness of the solution of the Cauchy problems, the claim follows. We denote both solutions by $Z_{p,{\mathfrak {m}}}(\psi )$ when $\psi \in \psi _{{\mathfrak {m}}}( B_{\texttt{r}_{p,+}}^{+}(\texttt{y}_{p,{\mathfrak {m}}})) = \psi _{{\mathfrak {m}}}(B_{\texttt{r}_{p+1,-}}^{-}(\texttt{y}_{p+1,{\mathfrak {m}}}))$ and we conclude that $Z_{p,{\mathfrak {m}}} \in \mathcal {C}^{S+1}\big ( \psi _{{\mathfrak {m}}}( B_{\texttt{r}_{p,+}}^{+}(\texttt{y}_{p,{\mathfrak {m}}})) \big )$, as well as for $F_{p,{\mathfrak {m}}}(\psi )=-\texttt{E}^2\psi + Z_{p,{\mathfrak {m}}}(\psi )$.

Finally, let $p=\kappa _0$. We define $F_{\kappa _0,{\mathfrak {m}}}(\psi )$ as the solution of the Cauchy problem

$$\begin{aligned} {\left\{ \begin{array}{ll} \partial _\psi F_{\kappa _0,{\mathfrak {m}}}(\psi ) = K_{\kappa _0,{\mathfrak {m}},+} ( G_{\kappa _0,{\mathfrak {m}},+}^{-1}(\psi ) ) , \quad \psi \in \psi _{{\mathfrak {m}}}(B_{\texttt{r}_{\kappa _0,+}}(\texttt{y}_{\kappa _0,{\mathfrak {m}}})) , \\ F_{\kappa _0,{\mathfrak {m}}} (\psi _{{\mathfrak {m}}}(\texttt{y}_{\kappa _0,{\mathfrak {m}}})) = \psi _{{\mathfrak {m}}}''(\texttt{y}_{\kappa _0,{\mathfrak {m}}}). \end{array}\right. } \end{aligned}$$

By Step 1 and the boundedness of the vector field following from Lemma 3.2, the problem is well defined, with $F_{\kappa _0,{\mathfrak {m}}} \in \mathcal {C}^{S+1}\big ( \psi _{{\mathfrak {m}}}(B_{\texttt{r}_{\kappa _0,+}}^{+}(\texttt{y}_{\kappa _0,{\mathfrak {m}}})) \big )$.

**Step 3) Global** $\mathcal {C}^{S+1}$-**continuity.** By Whitney extension Theorem, we extend all the functions $F_{p,{\mathfrak {m}}}(\psi )$, $p=0,1,...,\kappa _0$, from their domains of definition to global functions in $\mathcal {C}_{0}^{S+1}({{\mathbb {R}}})$. For sake of simplicity in the notation, we keep denoting the extensions by $F_{p,{\mathfrak {m}}}(\psi )$. Note that, by Step 1 and the construction of the Cauchy problems in Step 2, for any $p=0,1,....,\kappa _0$ and any $y\in \texttt{I}_{p}$, we have

$$\begin{aligned} (\partial _\psi F_{p,{\mathfrak {m}}})(\psi _{{\mathfrak {m}}}(y)) = Q_{{\mathfrak {m}}}(y). \end{aligned}$$

By the smoothness of $Q_{{\mathfrak {m}}}(y)$ and the choice of the initial values in the Cauchy problems, we obtain that both (3.38) and (3.39) hold. This concludes the proof of the theorem. $\square$

#### Remark 3.8

During the proof of Theorem 3.7, for $p=0,1,...,\kappa _0-1$ we constructed the nonlinearities $F_{p,{\mathfrak {m}}}(\psi ) = -\texttt{E}^2 \psi + Z_{p,{\mathfrak {m}}}(\psi )$ by defining Cauchy problems for $Z_{p,{\mathfrak {m}}}(\psi )$, whereas for $p=\kappa _0$ we directly consider the Cauchy problem for $F_{\kappa _0,{\mathfrak {m}}}(\psi )$. There is no conceptual difference between the two kinds of constructions. The reason behind this choice is purely expository: we just wanted to highlight that the nonlinearity $F_{p,{\mathfrak {m}}}(\psi )$ when $p < \kappa _0$ is a perturbation of the linear function $-\texttt{E}^2\psi$. It is also possible to show that, when $|y| \geqslant \texttt{r}+ \gamma$, we have $F_{\kappa _0,{\mathfrak {m}}}(\psi _{{\mathfrak {m}}}(y)) = 1 + Z_{\kappa _0,{\mathfrak {m}}}(\psi _{{\mathfrak {m}}}(y))$ for some small function $Z_{\kappa _0,{\mathfrak {m}}}(\psi )$. Morally speaking, the nonlinearities constructed in Theorem 3.7 are slight local modifications of the nonlinearity $F_{\infty }(\psi )$ in (3.34).

The following corollary of Theorem 3.7 and Lemmata 3.1, 3.6 will be used at the beginning of Sect. 4:

#### Corollary 3.9

For any $n=1,...,S$ and for any $p=1,...,\kappa _0$, we have

3.40 $$\begin{aligned} \begin{aligned}&(\partial _{\psi }^n F_{p,{\mathfrak {m}}})(\psi _{{\mathfrak {m}}}(y)) = (\partial _{\psi }^n F_{p-1,{\mathfrak {m}}})(\psi _{{\mathfrak {m}}}(y)) = {\mathfrak {P}}_{n}(y), \end{aligned} \end{aligned}$$

where the function ${\mathfrak {P}}_{n}(y)$ is independent of the strip index *p*. Moreover, there exists ${\overline{\delta }}={\overline{\delta }}(p,{\mathfrak {m}})>0$ small enough such that, for any $\delta \in (0,{\overline{\delta }})$, the function ${\mathfrak {P}}_{n}(y)$ satisfies the expansions

3.41 $$\begin{aligned} {\mathfrak {P}}_{n}(\texttt{y}_{p,\texttt{m}}+\delta ) = \sum _{k=0}^{S-n} \frac{{\mathfrak {p}}_{2k}^{(n)}}{(2k)!}\delta ^{2k} + \frac{{\mathfrak {p}}_{2(S-n)+1}^{(n)}}{(2(S-n)+1)!}\delta ^{2(S-n)+1} + o(|\delta |^{2(S-n+1)}). \nonumber \\ \end{aligned}$$

#### Proof

We argue by induction. For $n=1$, we have that (3.40) and (3.41) hold true by (3.38) and Lemma 3.1, setting ${\mathfrak {P}}_{0}(y):=Q_{{\mathfrak {m}}}(y)$. We now assume by induction that the claim holds for a fixed $n\in \{1,..,S-1\}$ and we show it for $n+1$. By differentiating (3.38) iteratively in *y*, we have

3.42 $$\begin{aligned} (\partial _{\psi }^{n+1} F_{p,{\mathfrak {m}}})(\psi _{{\mathfrak {m}}}(y)) = \frac{1}{\psi _{{\mathfrak {m}}}'(y)} \partial _y \big ( (\partial _{\psi }^n F_{p,{\mathfrak {m}}})(\psi _{{\mathfrak {m}}}(y)) \big ), \end{aligned}$$

with a similar formula for $F_{p-1,\texttt{m}}$. By the induction assumption, $(\partial _{\psi }^n F_{p,{\mathfrak {m}}})(\psi _{{\mathfrak {m}}}(y))$ and $(\partial _{\psi }^n F_{p-1,{\mathfrak {m}}})(\psi _{{\mathfrak {m}}}(y))$ satisfy (3.40) at the step *n*. This proves (3.40) at the step $n+1$, with ${\mathfrak {P}}_{n+1}(y)$ given by the right hand side of (3.42). Moreover, by the induction assumption, the expansion in (3.41) holds at the step *n* and we compute

3.43 $$\begin{aligned}&\partial _y \big ( (\partial _{\psi }^n F_{p,{\mathfrak {m}}})(\psi _{{\mathfrak {m}}}(y)) \big ) = \partial _y {\mathfrak {P}}_{n}(y) \nonumber \\&= \tfrac{{\mathfrak {p}}_{2}^{(n)}}{2}(y-\texttt{y}_{p,\texttt{m}}) + \sum _{k=2}^{S-n}\tfrac{{\mathfrak {p}}_{2k}^{(n)}}{(2k-1)!}(y-\texttt{y}_{p,\texttt{m}})^{2k-1} \nonumber \\&\quad + \tfrac{{\mathfrak {p}}_{2(S-n)+1}^{(n)}}{(2(S-n))!}(y-\texttt{y}_{p,\texttt{m}})^{2(S-n)} + o(|y-\texttt{y}_{p,\texttt{m}}|^{2(S-n)+1})\nonumber \\&= \tfrac{{\mathfrak {p}}_{2}^{(n)}}{2}(y-\texttt{y}_{p,\texttt{m}})\Big ( 1 + \sum _{k=1}^{S-n-1}\tfrac{{\widetilde{{\mathfrak {p}}}}_{2k}^{(n)}}{(2k)!}\left( y-\texttt{y}_{p,\texttt{m}}\right) ^{2k}\nonumber \\&\quad + \tfrac{{\widetilde{{\mathfrak {p}}}}_{2(S-n)-1}^{(n)}}{(2(S-n)-1)!}(y-\texttt{y}_{p,\texttt{m}})^{2(S-n)-1} + o(|y-\texttt{y}_{p,\texttt{m}}|^{2(S-n)}) \Big ), \quad {\widetilde{{\mathfrak {p}}}}_{j}^{(n)}:= \tfrac{2\,{\mathfrak {p}}_{j+2}^{(n)}}{(j+1){\mathfrak {p}}_{2}^{(n)}}. \end{aligned}$$

By Lemma 3.6, a similar computation leads to

$$\begin{aligned} \begin{aligned} \psi _{\texttt{m}}'(y)&= \tfrac{\psi _{{\mathfrak {m}}}''(\texttt{y}_{p,\texttt{m}})}{2} (y-\texttt{y}_{p,\texttt{m}})\Big ( 1 + \sum _{k=1}^{S} \tfrac{{\widetilde{\psi }}_{\texttt{m},2k}}{(2k)!}(y-\texttt{y}_{p,\texttt{m}})^{2k} \\&\quad + \tfrac{{\widetilde{\psi }}_{\texttt{m},2S+1}}{(2S+1)!}(y-\texttt{y}_{p,\texttt{m}})^{2S+1}+o(|y-\texttt{y}_{p,\texttt{m}}|^{2(S+1)}) \Big ), \quad {\widetilde{\psi }}_{\texttt{m},j}:=\tfrac{2\psi _{\texttt{m}}^{(j+2)}(\texttt{y}_{p,\texttt{m}})}{(j+1)\psi _{\texttt{m}}''(\texttt{y}_{p,\texttt{m}})}. \end{aligned} \end{aligned}$$

By Lemma 3.5-(*iii*), we get that $\psi _{\texttt{m}}'(y)$ is invertible for *y* sufficiently close to $\texttt{y}_{p,\texttt{m}}$, with $(\psi _{\texttt{m}}')^{-1}$ having a similar expansion as above, with different coefficients provided by the Neumann series. Combining such expansion in (3.42) together with (3.43), the claim in (3.41) holds at the step $n+1$. This concludes the proof. $\square$

### Spectral Analysis of the Linear Operator

We now want to study the spectrum of the operator $\mathcal {L}_{{\mathfrak {m}}} := - \partial _y^2 + Q_{{\mathfrak {m}}}(y)$. We shall emphasize that this linear operator depends on the parameter $\texttt{E}\in [\texttt{E}_1,\texttt{E}_2]$, with $1< \texttt{E}_1 < \texttt{E}_2$, hence we often write $\mathcal {L}_{{\mathfrak {m}}} \equiv \mathcal {L}_{{\mathfrak {m}}}(\texttt{E})$ and $Q_{{\mathfrak {m}}}(y) \equiv Q_{{\mathfrak {m}}}(\texttt{E};y)$. We shall prove that $\mathcal {L}_{{\mathfrak {m}}}(\texttt{E})$ has a finite number of negative eigenvalues, which we will use them in Sect. 5 in order to impose some Diophantine conditions by cutting away some resonance zones in the parameter space $[\texttt{E}_1,\texttt{E}_2]$. Such a property will be inferred from the limit operator $\mathcal {L}_{\infty }=\mathcal {L}_{\infty }(\texttt{E})$.

#### Proposition 3.10

The Schrödinger operator $\mathcal {L}_{{\mathfrak {m}}}:=-\partial _y^2 +Q_{{\mathfrak {m}}}(y)$, with $Q_{{\mathfrak {m}}}(y)$ as in (3.1), is self-adjoint in $L^2_0([-1,1])$ on the domain

$$\begin{aligned} D(\mathcal {L}_{{\mathfrak {m}}}):= \big \{\phi \in H_0^1([-1,1]) \, : \, \phi (y)=\phi (-y) \big \}, \end{aligned}$$

with a countable $L^2$-basis of eigenfunctions $(\phi _{j,{\mathfrak {m}}}(y))_{j\in {{\mathbb {N}}}}\subset \mathcal {C}^{\infty }[-1,1]$ corresponding to the eigenvalues $(\mu _{j,{\mathfrak {m}}})_{j\in {{\mathbb {N}}}}$. Moreover, under the constraint (1.4), with respect to the order $\mu _{1,{\mathfrak {m}}}< \mu _{2,{\mathfrak {m}}}< ...< \mu _{j,{\mathfrak {m}}} < ...$, there exists ${\overline{{\mathfrak {m}}}}= {\overline{{\mathfrak {m}}}}(\texttt{E}_1,\texttt{E}_2,\kappa _0)\gg 1$, possibly larger than the threshold in Proposition 3.4, such that, for any ${\mathfrak {m}}\geqslant {\overline{{\mathfrak {m}}}}$, the first $\kappa _0$ eigenvalues are strictly negative and larger than $-\texttt{E}^2$, whereas all the others are strictly positive; we write

$$\begin{aligned} \mu _{j,{\mathfrak {m}}}={\left\{ \begin{array}{ll} -\lambda _{j,{\mathfrak {m}}}^2\in (-\texttt{E}^2,0) &  \quad j=1,... ,\kappa _0,\\ \lambda _{j,{\mathfrak {m}}}^2>0 &  \quad j\geqslant \kappa _0+1. \end{array}\right. } \end{aligned}$$

In particular, for any $j=1,...,\kappa _0$, we have that $\lambda _{j,{\mathfrak {m}}}$ is close to $\lambda _{j,\infty }$, with the latter being the *j*-th root out of $\kappa _0$ of the transcendental equation in the region $\lambda \in (0,\texttt{E})$

3.44 $$\begin{aligned} {\mathfrak {F}}(\lambda ):= \lambda \cos \big (\texttt{r}\sqrt{\texttt{E}^2-\lambda ^2}\big )\coth ((1-\texttt{r})\lambda ) - \sqrt{\texttt{E}^2-\lambda ^2}\sin \big (\texttt{r}\sqrt{\texttt{E}^2-\lambda ^2}\big ) = 0 . \nonumber \\ \end{aligned}$$

#### Remark 3.11

The existence of the $\kappa _0$ roots of the Equation  (3.44) is established in Lemma 5.3, where an asymptotic for such zeroes when $\texttt{E}\rightarrow \infty$ is provided as well.

#### Proof

We split the proof in to several steps.

**Step 1) 0 is not an eigenvalue.** The self-adjointness of $\mathcal {L}_{\mathfrak {m}}$ and its spectral resolution follow by standard arguments of functional analysis. The smoothness of the eigenfunctions $(\phi _{j,{\mathfrak {m}}}(y))_{j\in {{\mathbb {N}}}}$ follows from standard regularity properties for the Sturm–Liouville problem, since $Q_{{\mathfrak {m}}}\in \mathcal {C}^{\infty }[-1,1]$ (it is actually analytic in $[-1,1]$).

We claim now that 0 is not an eigenvalue. Indeed, if we know that $\mu _{\kappa _0,{\mathfrak {m}}}<0$, we claim that $\mu _{\kappa _0+1,{\mathfrak {m}}}>0$. We argue by contradiction and we assume that $\mu _{\kappa _0+1,{\mathfrak {m}}}\leqslant 0$. We have that the odd function $\psi _{\mathfrak {m}}'(y)$ is a generalized eigenfunction for $\mathcal {L}_{\mathfrak {m}}=-\partial _y^2 +Q_{\mathfrak {m}}(y)$, since $\mathcal {L}_{\mathfrak {m}}\psi _{{\mathfrak {m}}}'=0$ by (3.1). Combining this with $\mathcal {L}_{{\mathfrak {m}}}\phi _{\kappa _0+1,{\mathfrak {m}}}= \mu _{\kappa _0+1,{\mathfrak {m}}}\phi _{\kappa _0+1,{\mathfrak {m}}}$, we get

3.45 $$\begin{aligned} \mu _{\kappa _0+1,{\mathfrak {m}}} \psi _{{\mathfrak {m}}}' (y)\phi _{\kappa _0+1,{\mathfrak {m}}} (y)= \big ( \psi _{{\mathfrak {m}}}''(y) \phi _{\kappa _0+1,{\mathfrak {m}}}(y) - \psi _{{\mathfrak {m}}}' (y)\phi _{\kappa _0+1,{\mathfrak {m}}}'(y) \big )'. \nonumber \\ \end{aligned}$$

Without loss of generality, we assume $\phi _{\kappa _0+1,{\mathfrak {m}}}(0)>0$ and, by symmetry, we work on $y\in [0,1]$. In this region, by Lemma 3.5-(iii), $\psi _{{\mathfrak {m}}}'(y)$ vanishes at the $\kappa _0+1$ points $\texttt{y}_{0,{\mathfrak {m}}}=0$, $(\texttt{y}_{p,{\mathfrak {m}}})_{p=1,...,\kappa _0}$. At the same time, the even eigenfunction $\phi _{\kappa _0+1,{\mathfrak {m}}}(y)$ vanishes at $\kappa _0$ nodes in (0, 1) (see for instance Theorem 9.4 in [46]). We deduce that one of the following cases have to happen:

- All the points $\texttt{y}_{p,{\mathfrak {m}}}$, $p=1,...,\kappa _0$, are also nodes for $\phi _{\kappa _0+1,{\mathfrak {m}}}$: $\psi _{{\mathfrak {m}}}'(\texttt{y}_{p,{\mathfrak {m}}})=\phi _{\kappa _0+1,{\mathfrak {m}}}(\texttt{y}_{p,{\mathfrak {m}}})=0$;
- There exists at least one $p=1,...,\kappa _0$ such that $\texttt{y}_{p,{\mathfrak {m}}}$ is not a zero of $\phi _{\kappa _0+1,{\mathfrak {m}}}$ and lies between two if its zeroes, say ${\overline{\texttt{y}}}_1$ and ${\overline{\texttt{y}}}_2$, with $0<{\overline{\texttt{y}}}_1< \texttt{y}_{p,{\mathfrak {m}}}<{\overline{\texttt{y}}}_2\leqslant 1$.

We assume that the second case holds. Furthermore, without any loss of generality, we also assume that $\phi _{\kappa _0+1,{\mathfrak {m}}}(y)>0$ and $\psi _{{\mathfrak {m}}}'(y)<0$ for any $y\in ({\overline{\texttt{y}}}_1,\texttt{y}_{p,{\mathfrak {m}}})$. We integrate (3.45) on $[{\overline{\texttt{y}}}_1,\texttt{y}_{p,{\mathfrak {m}}}]$ and we get

3.46 $$\begin{aligned}  &   \mu _{\kappa _0+1,{\mathfrak {m}}} \int _{{\overline{\texttt{y}}}_1}^{\texttt{y}_{p,{\mathfrak {m}}}} \psi _{{\mathfrak {m}}}'(y) \phi _{\kappa _0+1,{\mathfrak {m}}}(y)\,\textrm{d}{y} = \big [ \psi _{{\mathfrak {m}}}''(y) \phi _{\kappa _0+1,{\mathfrak {m}}}(y) - \psi _{{\mathfrak {m}}}'(y) \phi _{\kappa _0+1,{\mathfrak {m}}}'(y) \big ]_{{\overline{\texttt{y}}}_1}^{\texttt{y}_{p,{\mathfrak {m}}}} \nonumber \\  &   \quad = \psi _{{\mathfrak {m}}}''(\texttt{y}_{p,{\mathfrak {m}}})\phi _{\kappa _0+1,{\mathfrak {m}}}(\texttt{y}_{p,{\mathfrak {m}}}) + \psi _{{\mathfrak {m}}}'({\overline{\texttt{y}}}_1)\phi _{\kappa _0+1,{\mathfrak {m}}}({\overline{\texttt{y}}}_1). \end{aligned}$$

By construction, $\phi _{\kappa _0+1,{\mathfrak {m}}}(\texttt{y}_{p,{\mathfrak {m}}})>0$ and $\phi _{\kappa _0+1,{\mathfrak {m}}}'({\overline{\texttt{y}}}_1)>0$, whereas $\psi _{{\mathfrak {m}}}'({\overline{\texttt{y}}}_1)\leqslant 0$ and $\psi _{{\mathfrak {m}}}''(\texttt{y}_{p,{\mathfrak {m}}})>0$. We conclude that the right hand side of (3.46) is strictly negative and the integral on the left hand side is strictly negative by construction as wel. But we assumed at the beginning that $\mu _{\kappa _0+1,{\mathfrak {m}}}\leqslant 0$, therefore we have reached a contradiction and the claim is proved.

**Step 2) Negative eigenvalues in the limit case** ${\mathfrak {m}}\rightarrow \infty$. We consider first the limit case ${\mathfrak {m}}\rightarrow \infty$, with $Q_\infty (y)$ given in (3.1). Let $\lambda >0$. We look for solutions $\phi \in \mathcal {C}^1([-1,1])$ of the eigenvalue problem

3.47 $$\begin{aligned} {\left\{ \begin{array}{ll} \mathcal {L}_{\infty } \phi (y):-\phi ''(y) + Q_\infty (y)\phi (y) = -\lambda ^2\phi (y), &  \quad y\in [-1,1]\setminus \{\pm \texttt{r}\}, \\ \phi (y)=\phi (-y), \quad \phi (-1)=\phi (1)=0, \\ \lim _{y\rightarrow \pm \texttt{r}^{-}}\phi (y)=\lim _{y\rightarrow \pm \texttt{r}^{+}}\phi (y), \\ \lim _{y\rightarrow \pm \texttt{r}^{-}}\phi '(y)=\lim _{y\rightarrow \pm \texttt{r}^{+}}\phi '(y). \end{array}\right. } \end{aligned}$$

The conditions $\phi (y)=\phi (-y)$ and $\phi \in \mathcal {C}^1$ fail the problem to be solved when $\lambda \geqslant \texttt{E}$. On the other hand, for $0<\lambda <\texttt{E}$, the solutions however are given by

3.48 $$\begin{aligned} \phi (y)={\left\{ \begin{array}{ll} -c_1 \sinh (\lambda (1+y)) &  \quad -1\leqslant y<\texttt{r}, \\ c_2\cos (\varsigma (\lambda )y) &  \quad |y|\leqslant \texttt{r},\\ c_1 \sinh (\lambda (1-y)) &  \quad \texttt{r}<y\leqslant 1, \end{array}\right. } \end{aligned}$$

where $\varsigma (\lambda ):=\sqrt{\texttt{E}^2-\lambda ^2}$. The last two conditions in (3.47) at $y=\texttt{r}$ translate into

$$\begin{aligned} {\left\{ \begin{array}{ll} c_1 \sinh (\lambda (1-\texttt{r}))-c_2\cos (\varsigma (\lambda )\texttt{r})=0,\\ c_1 \lambda \cosh (\lambda (1-\texttt{r}))-c_2\varsigma (\lambda )\sin (\varsigma (\lambda )\texttt{r})=0. \end{array}\right. } \end{aligned}$$

A nontrivial solution $(c_1,c_2)\in {{\mathbb {R}}}^2\setminus \{0\}$ exists only when $\lambda$ solves

$$\begin{aligned} \varsigma (\lambda )\sin (\texttt{r}\varsigma (\lambda ))\sinh ((1-\texttt{r})\lambda )=\lambda \cos (\texttt{r}\varsigma (\lambda ))\cosh ((1-\texttt{r})\lambda ), \end{aligned}$$

which is equivalent to (3.44). Under the constrain (1.4), Equation (3.44) has exactly $\kappa _0$ distinct zeroes in the interval $\lambda \in (0,\texttt{E})$, see Remark 3.11 and Lemma 5.3. We denote these zeroes by $\lambda _{j,\infty }=\lambda _{j,\infty }(\texttt{E})$ for any $j=1,...,\kappa _0$. The negative eigenvalues of $\mathcal {L}_{\infty }$ are then given by $\mu _{j,\infty }=\mu _{j,\infty }(\texttt{E}):=-\lambda _{j,\infty }^2(\texttt{E})$, with corresponding eigenfunctions $\phi _{j,\infty }(y)=\phi _{j,\infty }(\texttt{E};y)$ given in (3.48) with $\lambda =\lambda _{j,\infty }(\texttt{E})$ and $c_1,c_2$ chosen as normalizing constants in $L^2$. We also have

$$\begin{aligned} \mu _{j,\infty } = {\mathfrak {B}}_{\infty } (\phi _{j,\infty }), \quad {\mathfrak {B}}_{\infty }(\psi ) = (\mathcal {L}_{\infty } \psi , \psi ) _{L^2}. \end{aligned}$$

**Step 3) Negative eigenvalues when** ${\mathfrak {m}}\gg 1$. We now analyse the case ${\mathfrak {m}}\gg 1$. In Step 1 we showed that, assuming only $\kappa _0$ negative eigenvalues of $\mathcal {L}_{{\mathfrak {m}}}$, the rest of the spectrum is strictly positive. We now prove that, for any $j=1,...,\kappa _0$,

3.49 $$\begin{aligned} \sup _{\texttt{E}\in [\texttt{E}_1, \texttt{E}_2]} |\mu _{j, {\mathfrak {m}}}(\texttt{E}) - \mu _{j, \infty }(\texttt{E})| \rightarrow 0 \quad \text {as} \quad \texttt{m}\rightarrow \infty , \end{aligned}$$

where $(\mu _{j, \infty })_{j=1,...,\kappa _0}$ are all the negative eigenvalues of the limit operator $\mathcal {L}_{\infty }:=-\partial _y^2 + Q_{\infty }(y)$ that we characterized in Step 2. For any $j = 1,..., \kappa _0$, let us denote by $\mathcal {E}_{j- 1}$ the set of all finite dimensional subspaces of dimension $j - 1$ of $H_0^1(- 1, 1)$. By the Min–Max Theorem (for instance, see [46]), we have that the eigenvalues $\mu _{1,{\mathfrak {m}}}< \mu _{2,{\mathfrak {m}}}< ... < \mu _{\kappa _0,{\mathfrak {m}}}$ of $\mathcal {L}_{\mathfrak {m}}$ satisfy the variational formulation, for any $j =1,...,\kappa _0$,

3.50 $$\begin{aligned} \begin{aligned}&\mu _{j,{\mathfrak {m}}} := \sup _{E \in \mathcal {E}_{j - 1}} \inf \big \{ {\mathfrak {B}}_{{\mathfrak {m}}}(\psi ) \, : \, \psi \in E^\bot \cap H_0^1(-1, 1), \ \Vert \psi \Vert _{L^2} = 1 \big \} , \\&{\mathfrak {B}}_{{\mathfrak {m}}}(\psi ):= (\mathcal {L}_{\mathfrak {m}}\psi ,\psi )_{L^2}, \end{aligned} \end{aligned}$$

Each eigenfunction $\phi _{j,{\mathfrak {m}}}(y)$ is the solution to the max-min variational problems in (3.50). Let $E_j = \textrm{span}\{ \phi _{1, \infty }, \ldots , \phi _{j - 1, \infty } \} \in \mathcal {E}_{j - 1}$ and let $\phi _{j, \infty } \in E_j^\bot \cap H_0^1(- 1, 1)$ where, for $i =1,...,\kappa _0$, the function $\phi _{i, \infty }$ is the i-th eigenfunction of the operator $\mathcal {L}_{\infty }$. By standard theory for Sturm Liouville operators and by the Sobolev embedding, for any $\texttt{E}\in [\texttt{E}_1, \texttt{E}_2]$, one has that $\phi _{j, \infty } \equiv \phi _{j, \infty }(\texttt{E})$ satisfies

3.51 $$\begin{aligned} \Vert \phi _{j, \infty } \Vert _{L^\infty } \lesssim \Vert \phi _{j, \infty } \Vert _{H^1} \leqslant C(\texttt{E}_1, \texttt{E}_2, j), \end{aligned}$$

for some constant $C(\texttt{E}_1, \texttt{E}_2, j) > 0$. We compute

$$\begin{aligned} \begin{aligned} {\mathfrak {B}}_{{\mathfrak {m}}}(\phi _{j, \infty })&=(\mathcal {L}_{\mathfrak {m}}\phi _{j, \infty }, \phi _{j, \infty })_{L^2} = (\mathcal {L}_{\infty }\phi _{j, \infty }, \phi _{j, \infty })_{L^2} + \big ( (\mathcal {L}_{\mathfrak {m}}- \mathcal {L}_{\infty }) \phi _{j, \infty },\, \phi _{j, \infty } \big )_{L^2} \\&\leqslant \lambda _{j, \infty } + |c_j({\mathfrak {m}}, \texttt{E})|, \quad c_j({\mathfrak {m}}, \texttt{E}) := \big ( (Q_{\mathfrak {m}}(y) - Q_\infty (y)) \phi _{j, \infty },\, \phi _{j, \infty } \big )_{L^2}. \end{aligned} \end{aligned}$$

By a similar computation, we also have

$$\begin{aligned} \lambda _{j, \infty } = {\mathfrak {B}}_{\infty }(\phi _{j, \infty }) \leqslant {\mathfrak {B}}_{{\mathfrak {m}}}(\phi _{j, \infty }) + |c_j({\mathfrak {m}}, \texttt{E})|. \end{aligned}$$

By taking the infimum over $\psi \in E_j^\bot$ with $\Vert \psi \Vert _{L^2} = 1$ one gets the two inequalities

$$\begin{aligned} \begin{aligned} \inf \{ {\mathfrak {B}}_{{\mathfrak {m}}}(\psi ) : \psi \in E_j^\bot , \ \Vert \psi \Vert _{L^2} = 1 \} \leqslant \lambda _{j, \infty } + |c_j({\mathfrak {m}}, \texttt{E})|, \\ \lambda _{j, \infty } \leqslant \inf \{ {\mathfrak {B}}_{{\mathfrak {m}}}(\psi ) : \psi \in E_j^\bot , \ \Vert \psi \Vert _{L^2} = 1 \} + |c_j({\mathfrak {m}}, \texttt{E})| \end{aligned} \end{aligned}$$

and then, by taking the supremum over $E \in \mathcal {E}_{j - 1}$, one obtains that

$$\begin{aligned} \mu _{j, {\mathfrak {m}}}(\texttt{E}) \leqslant \mu _{j, \infty }(\texttt{E}) + |c_j({\mathfrak {m}}, \texttt{E})|, \quad \mu _{j, \infty }(\texttt{E}) \leqslant \mu _{j, {\mathfrak {m}}}(\texttt{E}) + |c_j({\mathfrak {m}}, \texttt{E})| \end{aligned}$$

namely

$$\begin{aligned} |\mu _{j, {\mathfrak {m}}}(\texttt{E}) - \mu _{j, \infty }(\texttt{E})| \leqslant |c_j({\mathfrak {m}}, \texttt{E})| \end{aligned}$$

It remains to estimate the term $c_j({\mathfrak {m}}, \texttt{E}) = \big ( (Q_\infty (y) - Q_{\mathfrak {m}}(y)) \phi _{j, \infty },\, \phi _{j, \infty } \big )_{L^2}$. By the Cauchy–Schwartz inequality, using that $\Vert \phi _{j, \infty } \Vert _{L^2} = 1$, one has

$$\begin{aligned} \begin{aligned} \big | \big (&(Q_\infty (y) - Q_{\mathfrak {m}}(y)) \phi _{j, \infty }\, \phi _{j, \infty } \big )_{L^2} \big | \leqslant \Vert (Q_\infty (y) - Q_{\mathfrak {m}}(y)) \phi _{j, \infty } \Vert _{L^2} \Vert \phi _{j, \infty } \Vert _{L^2} \\  &\leqslant \Vert Q_{\mathfrak {m}}- Q_\infty \Vert _{L^2} \Vert \phi _{j, \infty } \Vert _{L^\infty } {\mathop {\leqslant }\limits ^{(3.3)}} C(\texttt{E}_1, \texttt{E}_2, \kappa _0) \Vert Q_{\mathfrak {m}}- Q_0\Vert _{L^2} \rightarrow 0 \quad \text {as} \quad {\mathfrak {m}}\rightarrow 0 \end{aligned} \end{aligned}$$

by Lemma 3.2, uniformly with respect to $\texttt{E}\in [\texttt{E}_1, \texttt{E}_2]$. Hence, we deduce (3.49) and, by fixing ${\overline{{\mathfrak {m}}}} = {\overline{{\mathfrak {m}}}}(\texttt{E}_1,\texttt{E}_2,\kappa _0)\gg 1$ sufficiently large, for any ${\mathfrak {m}}\geqslant {\overline{{\mathfrak {m}}}}$ we get $-\texttt{E}^2<\mu _{1, {\mathfrak {m}}}(\texttt{E})< \ldots< \mu _{\kappa _0, {\mathfrak {m}}}(\texttt{E}) < 0$ for any $\texttt{E}\in [\texttt{E}_1, \texttt{E}_2]$. since $-\texttt{E}^2<\mu _{1, \infty }(\texttt{E})< \ldots< \mu _{\kappa _0, \infty }(\texttt{E}) < 0$. This concludes the proof. $\square$

#### Remark 3.12

The estimate (3.49) actually holds when $j\geqslant \kappa _0+1$. The proof is essentially identical and it is here omitted, since we are interested only in the full characterization of the negative spectrum. We also remark that, by refining the result of the $L^p$ convergence in Lemma 3.2, it is possible to show an explicit rate of convergence of (3.49) with respect to ${\mathfrak {m}}\gg 1$.

## The Nonlinear Elliptic Systems with Oscillating Modes

In the previous section, we constructed the stream function $\psi _{{\mathfrak {m}}}(y)$, which is a steady solution of the Euler equation (1.1), that locally solves the second-order nonlinear ODE in Theorem 3.7. We now go back to the search of *x*-dependent solutions that are perturbations of the shear equilibrium $\psi _{{\mathfrak {m}}}(y)$. First, in Proposition 4.1 we suitably modify the local nonlinearities of Theorem 3.7, leading to to the elliptic systems in (4.11). Then, we analyse the linearized systems at the equilibrium ${\varphi }\equiv 0$ and the parametrization of the “spatial phase space” based on the solutions of such linearized systems. This choice of coordinates is then used to search the solutions for the nonlinear elliptic system (4.11) as zeroes of the nonlinear functional (4.43) via a Nash–Moser implicit function Theorem, whose statement is provided at the end of the section.

### Regularization of the Nonlinearity

In Theorem 3.7 we constructed functions $F_{p,{\mathfrak {m}}}(\psi )$ for any stripe index $p=0,1,...,\kappa _0$ so that the unperturbed stream function $\psi _{{\mathfrak {m}}}(y)$ solves the equation in (3.37) on each stripe $\texttt{I}_{p}$. The functions are $\mathcal {C}^{S+1}$-continuous and they satisfy the regularity conditions (3.39) at the critical points $(\texttt{y}_{p,{\mathfrak {m}}})_{p=1,...,\kappa _0}$. Even without taking derivatives, these conditions are clearly violated by perturbation of $\psi _{{\mathfrak {m}}}(y)$, in the sense that, for generic functions ${\varphi }(x,y)$, we have

$$\begin{aligned} \lim _{y\rightarrow \pm \texttt{r}^{-}} F_{p-1,{\mathfrak {m}}}(\psi _{{\mathfrak {m}}}(y)+{\varphi }(x,y)) \ne \lim _{y\rightarrow \pm \texttt{r}^{+}} F_{p,{\mathfrak {m}}}(\psi _{{\mathfrak {m}}}(y)+{\varphi }(x,y)) \quad \forall \,x\in {{\mathbb {R}}}. \end{aligned}$$

The continuity would be recovered for these nonlinearities only we ask ${\varphi }(x,\texttt{y}_{p,{\mathfrak {m}}})\equiv 0$ in $x\in {{\mathbb {R}}}$, which is a too strong condition. To this end, we need to modify the nonlinear functions to avoid this issue around the critical points and accommodate small perturbations of $\psi _{{\mathfrak {m}}}(y)$. We introduce a small parameter $\eta >0$. For any stripe index $p=0,...,\kappa _0$, we define the functions,

4.1 $$\begin{aligned} \begin{aligned} F_{p,\eta }(\psi ):= F_{p,{\mathfrak {m}}}(\psi )&+\tfrac{1}{2} \chi _{\eta }(\psi - \psi _{{\mathfrak {m}}}(\texttt{y}_{p,{\mathfrak {m}}})) \big ( F_{p-1,{\mathfrak {m}}}(\psi )- F_{p,{\mathfrak {m}}}(\psi ) \big ) \\&+\tfrac{1}{2} \chi _{\eta }(\psi - \psi _{{\mathfrak {m}}}(\texttt{y}_{p+1,{\mathfrak {m}}})) \big ( F_{p+1,{\mathfrak {m}}}(\psi )- F_{p,{\mathfrak {m}}}(\psi ) \big ), \end{aligned} \end{aligned}$$

with $F_{-1,{\mathfrak {m}}}:=F_{0,{\mathfrak {m}}}$, $\texttt{y}_{\kappa _0+1,{\mathfrak {m}}}:=1$, $F_{\kappa _0+1,{\mathfrak {m}}}:=F_{\kappa _0,{\mathfrak {m}}}$, and where the cut-off function $\chi _\eta$ has the form

4.2 $$\begin{aligned} \begin{aligned}&\chi _\eta (\psi ) := \chi (\psi /\eta ), \quad \chi \in C^\infty ({{\mathbb {R}}}), \quad \chi (\psi ) = \chi (- \psi ), \\&\quad 0 \leqslant \chi \leqslant 1, \quad \chi \equiv 1 \ \text { on } \ B_{1}(0), \quad \chi \equiv 0 \ \text { on } \ {{\mathbb {R}}}\setminus B_{2}(0), \\&\quad \chi '(|\psi |) \leqslant 0, \quad \forall \psi \in {{\mathbb {R}}}. \end{aligned} \end{aligned}$$

#### Proposition 4.1

(Modified local nonlinearities) The following hold:

(i) The functions $F_{p,\eta }$ in (4.1) are in $\mathcal {C}_0^{S+1}({{\mathbb {R}}})$. Moreover, for any stripe index *p*, we have $\Vert F_{p,\eta }-F_{p,{\mathfrak {m}}}\Vert _{L^\infty ({{\mathbb {R}}})} \rightarrow 0$ as $\eta \rightarrow 0$;
(ii) We have $F_{p-1,\eta }=F_{p,\eta }$ on $B_{\eta }(\psi _{{\mathfrak {m}}}(\texttt{y}_{p,{\mathfrak {m}}}))$ for any $p=1,...,\kappa _0$. As a consequence, for any sufficiently smooth function ${\varphi }(\textbf{x},y)$ sufficiently small in $L^\infty$ the regularity conditions
  $$\begin{aligned}  &   \lim _{|y|\rightarrow \texttt{y}_{p,{\mathfrak {m}}}^-} \partial _{(\textbf{x},y)}^{n} (F_{p-1,\eta }(\psi _{{\mathfrak {m}}}(y) +{\varphi }(\textbf{x},y)) )\\  &   \quad = \lim _{|y|\rightarrow \texttt{y}_{p,{\mathfrak {m}}}^+}\partial _{(\textbf{x},y)}^{n}( F_{p,\eta }(\psi _{{\mathfrak {m}}}(y)+{\varphi }(\textbf{x},y)) ) . \end{aligned}$$
  are satisfied for $n\in {{\mathbb {N}}}_0^{\kappa _0+1}$, $0\leqslant |n|\leqslant S$, and for any $\textbf{x}\in {{\mathbb {T}}}^{\kappa _{0}}$, $p=1,...,\kappa _0$;
(iii) For any $n \in {{\mathbb {N}}}_0$, with $n \leqslant S + 1$, one has
  $$\begin{aligned} \sup _{\psi \in {{\mathbb {R}}}} |F_{p, \eta }^{(n)}(\psi )| \lesssim \eta ^{- n}; \end{aligned}$$
(iv) There exists ${\overline{\eta }}={\overline{\eta }}({\mathfrak {m}},S)>0$ small enough such that, for any $\eta \in [0,{\overline{\eta }}]$ and for any $n=0,1,...,S+1$
  4.3 $$\begin{aligned} \sup _{y\in [-1,1]}\sup _{p=0,1,...,\kappa _0}|F_{p,\eta }^{(n)}(\psi _{{\mathfrak {m}}}(y)) - F_{p,{\mathfrak {m}}}^{(n)}(\psi _{{\mathfrak {m}}}(y)) | \lesssim _{n} \eta ^{S+\tfrac{3}{2} -n} . \end{aligned}$$

#### Proof

The proof of items (*i*) and (*ii*) are a direct consequence of (4.1), (4.2) and of Theorem 3.7. The item (*iii*) follows by an explicit calculation, by differentiating the formula (4.1) and using that the cut off function $\chi _\eta$ in (4.2) satisfies $|\partial _\psi ^n \chi _\eta (\psi )| \lesssim _n \eta ^{- n}$.

We now prove item (*iv*). By (4.1), (4.2), the claim trivially holds when $\psi _{{\mathfrak {m}}}(y) \notin B_{2\eta } (\psi _{{\mathfrak {m}}}(\texttt{y}_{p,{\mathfrak {m}}}))\cup B_{2\eta } (\psi _{{\mathfrak {m}}}(\texttt{y}_{p+1,{\mathfrak {m}}}))$. Therefore, let $\psi _{{\mathfrak {m}}}(y) \in B_{2\eta } (\psi _{{\mathfrak {m}}}(\texttt{y}_{p,{\mathfrak {m}}}))$. The case $\psi _{{\mathfrak {m}}}(y) \in B_{2\eta } (\psi _{{\mathfrak {m}}}(\texttt{y}_{p+1,{\mathfrak {m}}}))$ works similarly and we omit it. By Lemma 3.5, for $\eta >0$ sufficiently small, there exist $\delta ^{\pm } = \delta ^{\pm }(p,{\mathfrak {m}},\texttt{E})>0$ such that $\psi _{{\mathfrak {m}}}(y) \in B_{2\eta } (\psi _{{\mathfrak {m}}}(\texttt{y}_{p,{\mathfrak {m}}}))$ is parametrized by

4.4 $$\begin{aligned} \begin{aligned}&\psi _{{\mathfrak {m}}}(y) = \psi _{{\mathfrak {m}}}(\texttt{y}_{p,{\mathfrak {m}}}-\delta ^{-}) = \psi _{{\mathfrak {m}}}(\texttt{y}_{p,{\mathfrak {m}}}+\delta ^{+}), \\&\texttt{y}_{p,{\mathfrak {m}}}-\delta ^{-}\in \texttt{I}_{p-1},\quad \texttt{y}_{p,{\mathfrak {m}}}+\delta ^{+} \in \texttt{I}_{p}. \end{aligned} \end{aligned}$$

In particular, by (4.4), Lemma 3.5-(*iii*) and the mean value Theorem, we get

4.5 $$\begin{aligned} \begin{aligned} {\varphi }= {\varphi }(\delta ^{\pm })&:= \psi _{{\mathfrak {m}}}(\texttt{y}_{p,{\mathfrak {m}}}\pm \delta ^{\pm }) - \psi _{{\mathfrak {m}}}(\texttt{y}_{p,{\mathfrak {m}}}) = \int _{0}^{\pm \delta ^{\pm }} \psi _{{\mathfrak {m}}}'(\texttt{y}_{p,{\mathfrak {m}}}+\delta _1)\,\textrm{d}{\delta }_1 \\&= \int _{0}^{\pm \delta ^{\pm }} \int _{0}^{\delta _1} \psi _{{\mathfrak {m}}}''(\texttt{y}_{p,{\mathfrak {m}}}+\delta _2)\,\textrm{d}{\delta }_2 \,\textrm{d}{\delta }_1 \simeq \tfrac{\psi _{{\mathfrak {m}}}''(\texttt{y}_{p,{\mathfrak {m}}})}{2} (\delta ^{\pm })^2, \end{aligned} \end{aligned}$$

for $\delta ^{\pm }$ sufficiently small, from which we deduce that

4.6 $$\begin{aligned} \delta ^{\pm }({\varphi }) = O({\varphi }^\frac{1}{2}) = O(\eta ^{\frac{1}{2}}) , \quad \eta \rightarrow 0. \end{aligned}$$

We claim now that

4.7 $$\begin{aligned} (\delta ^{+})^2 - (\delta ^{-})^2 = O(\eta ^{S+\frac{3}{2}}). \end{aligned}$$

To see this, we recall the expansion in Lemma 3.6. By (4.4), we compute

$$\begin{aligned} \begin{aligned} 0&= \psi _{{\mathfrak {m}}}(\texttt{y}_{p,{\mathfrak {m}}}+\delta ^{+}) - \psi _{{\mathfrak {m}}}(\texttt{y}_{p,{\mathfrak {m}}}-\delta ^{-}) = \sum _{n=0}^{S+1} \frac{\psi _{{\mathfrak {m}}}^{(2n)}(\texttt{y}_{p,{\mathfrak {m}}})}{(2n)!}\big ( (\delta ^{+})^{2n} -(\delta ^{-})^{2n} \big ) \\&\quad + \frac{\psi _{{\mathfrak {m}}}^{(2S+3)}(\texttt{y}_{p,{\mathfrak {m}}})}{(2S+3)!}\big ( (\delta ^{+})^{2S+3}+(\delta ^{-})^{2S+3} \big ) + o\big ( |\delta ^{+}|^{2(S+2)} + |\delta ^{-}|^{2(S+2)} \big ) \end{aligned} \end{aligned}$$

It follows that

$$\begin{aligned} \begin{aligned}&\frac{\psi _{{\mathfrak {m}}}^{''}(\texttt{y}_{p,{\mathfrak {m}}})}{2}\big ( (\delta ^{+})^{2} -(\delta ^{-})^{2} \big ) \Big ( 1 + o\big ( (\delta ^{+})^{2} + (\delta ^{-})^{2} \big ) \Big ) \\&\quad = - \frac{\psi _{{\mathfrak {m}}}^{(2S+3)}(\texttt{y}_{p,{\mathfrak {m}}})}{(2S+3)!}\big ( (\delta ^{+})^{2S+3}+(\delta ^{-})^{2S+3} \big ) + o\big ( |\delta ^{+}|^{2(S+2)} + |\delta ^{-}|^{2(S+2)} \big ). \end{aligned} \end{aligned}$$

Therefore, by Lemma 3.5-(*iii*) and (4.6), for $\eta >0$ sufficiently small, we can invert the factor $\frac{\psi _{{\mathfrak {m}}}^{''}(\texttt{y}_{p,{\mathfrak {m}}})}{2}\big ( 1 + o\big ( (\delta ^{+})^{2} + (\delta ^{-})^{2} \big ) \big )$ and deduce the claim in (4.7) as desired.

We are now ready to prove (4.3). We start with $n=0$. By (4.1), (4.4), (4.5), (3.1), Theorem 3.7, Lemma (3.6) and the mean value Theorem, we compute

4.8 $$\begin{aligned} \begin{aligned}&F_{p,\eta }(\psi _{{\mathfrak {m}}}(y)) - F_{p,{\mathfrak {m}}}(\psi _{{\mathfrak {m}}}(y)) = \tfrac{1}{2}\chi _{\eta }({\varphi }) \big ( F_{p-1,{\mathfrak {m}}} (\psi _{{\mathfrak {m}}}(y) ) - F_{p,{\mathfrak {m}}}(\psi _{{\mathfrak {m}}}(y) \big )\\&\quad =\tfrac{1}{2}\chi _{\eta }({\varphi })\big ( F_{p-1,{\mathfrak {m}}} (\psi _{{\mathfrak {m}}}(\texttt{y}_{p,{\mathfrak {m}}}-\delta ^{-})) - F_{p,{\mathfrak {m}}}(\psi _{{\mathfrak {m}}}(\texttt{y}_{p,{\mathfrak {m}}}+\delta ^{+}))\big ) \\&\quad =\tfrac{1}{2}\chi _{\eta }({\varphi })\big ( \psi _{{\mathfrak {m}}}''(\texttt{y}_{p,{\mathfrak {m}}}-\delta ^{-}) - \psi _{{\mathfrak {m}}}''(\texttt{y}_{p,{\mathfrak {m}}}+\delta ^{+})\big ) \\&\quad =\tfrac{1}{2} \chi _{\eta }({\varphi })\int _{\delta ^{+}}^{-\delta ^{-}} \psi _{{\mathfrak {m}}}'''(\texttt{y}_{p,{\mathfrak {m}}}+\delta _1) \,\textrm{d}{\delta }_1 \\&\quad =\tfrac{1}{2} \chi _{\eta }({\varphi })\int _{\delta ^{+}}^{-\delta ^{-}}\big ( Q_{{\mathfrak {m}}} \psi _{{\mathfrak {m}}}' \big )(\texttt{y}_{p,{\mathfrak {m}}}+\delta _1) \,\textrm{d}{\delta }_1\\&\quad =\tfrac{1}{2} \chi _{\eta }({\varphi })\int _{\delta ^{+}}^{-\delta ^{-}}\int _{0}^{\delta _1} \big ( Q_{{\mathfrak {m}}}\psi _{{\mathfrak {m}}}' \big )'(\texttt{y}_{p,{\mathfrak {m}}}+\delta _2) \,\textrm{d}{\delta }_2 \,\textrm{d}{\delta }_1 \\&\quad =\tfrac{1}{2} \chi _{\eta }({\varphi })\int _{\delta ^{+}}^{-\delta ^{-}}\int _{0}^{\delta _1} \big ( Q'_{{\mathfrak {m}}}\psi _{{\mathfrak {m}}}' + Q_{{\mathfrak {m}}} \psi _{{\mathfrak {m}}}'' \big )(\texttt{y}_{p,{\mathfrak {m}}}+\delta _2) \,\textrm{d}{\delta }_2 \,\textrm{d}{\delta }_1 \end{aligned} \end{aligned}$$

By (4.8), (4.5), (4.2) Lemma 3.2 and (4.7), we conclude that, for $\eta$ sufficiently small,

$$\begin{aligned} | F_{p,\eta }(\psi _{{\mathfrak {m}}}(y)) - F_{p,{\mathfrak {m}}}(\psi _{{\mathfrak {m}}}(y))| \lesssim \big | (\delta ^{+})^2 - (\delta ^{-})^2 \big | \lesssim \eta ^{S+\frac{3}{2}}. \end{aligned}$$

Therefore the claim holds for $n=0$. Let now $n\in \{1,...,S+1\}$. First, we look for the derivatives of $F_{p-1,{\mathfrak {m}}}(\psi _{{\mathfrak {m}}}(y))-F_{p,{\mathfrak {m}}}(\psi _{{\mathfrak {m}}}(y))$, still with $\psi _{{\mathfrak {m}}}(y)$ as in (4.4). By Corollary 3.9 and the mean value Theorem, we have

4.9 $$\begin{aligned}&F_{p-1,{\mathfrak {m}}}^{(n)}(\psi _{{\mathfrak {m}}}(y)) - F_{p,{\mathfrak {m}}}^{(n)}(\psi _{{\mathfrak {m}}}(y)) = F_{p-1,{\mathfrak {m}}}^{(n)}(\psi _{{\mathfrak {m}}}(\texttt{y}_{p,{\mathfrak {m}}}-\delta ^{-})) \nonumber \\&\quad - F_{p,{\mathfrak {m}}}^{(n)}(\psi _{{\mathfrak {m}}}(\texttt{y}_{p,{\mathfrak {m}}}+\delta ^{+})) \nonumber \\&\quad = {\mathfrak {P}}_{n-1}(\texttt{y}_{p,{\mathfrak {m}}}-\delta ^{-}) - {\mathfrak {P}}_{n-1}(\texttt{y}_{p,{\mathfrak {m}}}+\delta ^{+}) = \int _{\delta ^{+}}^{-\delta ^{-}} {\mathfrak {P}}_{n-1} '(\texttt{y}_{p,{\mathfrak {m}}}+\delta _1) \,\textrm{d}{\delta }_1. \end{aligned}$$

By (3.41) in Corollary 3.9, it is clear, for $n=1,...,S$, that ${\mathfrak {P}}_{n-1}'(\texttt{y}_{p,\texttt{m}})=0$. Therefore, again by the mean value Theorem, we obtain

4.10 $$\begin{aligned} \begin{aligned} {\mathfrak {P}}_{n-1} '(\texttt{y}_{p,{\mathfrak {m}}}+\delta _1) = \int _{0}^{\delta _1} {\mathfrak {P}}_{n-1} ''(\texttt{y}_{p,{\mathfrak {m}}}+\delta _2) \,\textrm{d}{\delta }_2. \end{aligned} \end{aligned}$$

By Lemmata 3.2, 3.5, the integrand in (4.10) is uniformly bounded in the domain of integration for $\eta >0$ sufficiently small. Therefore, collecting (4.9) with (4.10), we have the estimate

$$\begin{aligned} | F_{p-1,{\mathfrak {m}}}^{(n)}(\psi _{{\mathfrak {m}}}(y)) - F_{p,{\mathfrak {m}}}^{(n)}(\psi _{{\mathfrak {m}}}(y)) | \lesssim _{n} \big | (\delta ^{+})^2 - (\delta ^{-})^2 \big | \lesssim \eta ^{S+\frac{3}{2}}. \end{aligned}$$

We finally prove the claimed estimate (4.3) for $n\in \{1,...,S+1\}$. By Leibniz rule and by (4.4), we have

$$\begin{aligned} \begin{aligned}&\partial _{\psi }^{n}\big (F_{p,\eta }(\psi )-F_{p,{\mathfrak {m}}}(\psi )\big ) = \sum _{j=0}^{n}\left( {\begin{array}{c}n\\ j\end{array}}\right) \chi _{\eta }^{(n-j)}({\varphi }) \big ( F_{p-1}^{(j)}(\psi ) - F_{p,{\mathfrak {m}}}^{(j)}(\psi ) \big ) \end{aligned} \end{aligned}$$

The claim (4.3) then follows by (4.10) and (4.2), recalling that $|\chi _{\eta }^{n-j}({\varphi })| \leqslant \eta ^{-(n-j)}$. This concludes the proof of the proposition. $\square$

### The Hamiltonian Formulation

The manifold of the zeroes (1.12) admits a formulation as an Hamiltonian vector field. First, for fixed $\eta >0$, we rewrites the elliptic equations in (1.12) as the second order forced PDE

4.11 $$\begin{aligned} {\left\{ \begin{array}{ll} (\omega \cdot \partial _\textbf{x})^2{\varphi }-\mathcal {L}_{{\mathfrak {m}}}{\varphi }-g_{\eta }(y,{\varphi })= f_{\eta }(y), \quad (\textbf{x},y)\in {{\mathbb {T}}}^{\kappa _0} \times [-1,1], \\ {\varphi }(\textbf{x}, -1) = {\varphi }(\textbf{x}, 1) = 0 , \quad \omega \in {{\mathbb {R}}}^{\kappa _0} , \end{array}\right. } \end{aligned}$$

where the operator $\mathcal {L}_{{\mathfrak {m}}}$ is as in Theorem 3.10, the forcing term $f_{\eta }(y)$ is defined by

4.12 $$\begin{aligned} f_{\eta }(y):= \sum _{p=0}^{\kappa _0} \chi _{\texttt{I}_{p}}(y) f_{p,\eta }(y), \quad f_{p,\eta }(y):= F_{p,\eta }(\psi _{{\mathfrak {m}}}(y)) - F_{p,{\mathfrak {m}}}(\psi _{{\mathfrak {m}}}(y)), \nonumber \\ \end{aligned}$$

and the nonlinear function $g_{\eta }(y,{\varphi })$ is defined by

4.13 $$\begin{aligned} \begin{aligned}&g_{\eta }(y,{\varphi }):= \sum _{p=0}^{\kappa _0} \chi _{\texttt{I}_{p}}(y) g_{p,\eta }(y,{\varphi }), \\&g_{p,\eta }(y,{\varphi }) :=F_{p,\eta }(\psi _{{\mathfrak {m}}}(y)+{\varphi }) - F_{p,\eta }(\psi _{{\mathfrak {m}}}(y))- F_{p,{\mathfrak {m}}}'(\psi _{{\mathfrak {m}}}(y)) {\varphi }\\&\ \ = \big ( F_{p,\eta }'(\psi _{{\mathfrak {m}}}(y)) - F_{p,{\mathfrak {m}}}'(\psi _{{\mathfrak {m}}}(y)) \big ) {\varphi }+ \int _{0}^{1} (1-\varrho ) F_{p,\eta }''(\psi _{{\mathfrak {m}}}(y)+\varrho {\varphi }) \,\textrm{d}{\varrho }\, {\varphi }^2\,; \end{aligned} \end{aligned}$$

here $\chi _{\texttt{I}_{p}}$ denotes the characteristic function for the interval $\texttt{I}_{p}$, $F_{p,\eta }(\psi )$ is as in (4.1) and $F_{p,{\mathfrak {m}}}(\psi )$ is as in Theorem 3.7. Note that, by (3.39) and Proposition 4.1 the compatibility conditions

$$\begin{aligned} \begin{aligned}&\lim _{|y|\rightarrow \texttt{y}_{p,{\mathfrak {m}}}^-} \partial _{(\textbf{x},y)}^{n} \big ( g_{p-1,\eta }(y,{\varphi }(\textbf{x},y))\big ) = \lim _{|y|\rightarrow \texttt{y}_{p,{\mathfrak {m}}}^+} \partial _{(\textbf{x},y)}^{n} \big ( g_{p,\eta }(y,{\varphi }(\textbf{x},y))\big ), \end{aligned} \end{aligned}$$

hold for any $n\in {{\mathbb {N}}}_0^{\kappa _0+1}$, $0\leqslant |n|\leqslant S$, and $p=1,...,\kappa _0$, assuming the smallness condition, for $s_0 \leqslant s \leqslant q(S,k_0)$,

$$\begin{aligned} \Vert {\varphi }\Vert _{s_0,1}^{k_0,\upsilon } \leqslant C \varepsilon \ll 1, \end{aligned}$$

which implies the small bound in $L^\infty$. We now provide a Lemma in which we estimate the forcing term $f_\eta$ and the nonlinearity $g_\eta$ in (4.12), (4.13).

#### Lemma 4.2

The following estimates hold:

(i) **(Estimates in** $H^1_y$**).** We have $\Vert f_\eta \Vert _{H^1} \lesssim \eta ^{S + \frac{1}{2}}$. Moreover, the composition operator $\varphi \in B_1(0) \mapsto g_\eta (y, \varphi (y)) \in H_0^1([- 1, 1])$, with $B_1(0) := \{ \varphi \in H_0^1([- 1, 1]) : \Vert \varphi \Vert _{H^1} \leqslant 1\}$, satisfies the estimates
  $$\begin{aligned} \begin{aligned}&\Vert g_\eta (\cdot , \varphi ) \Vert _{H^1} \lesssim \eta ^{S - \frac{1}{2}}\Vert \varphi \Vert _{H^1} + \eta ^{- 3} \Vert \varphi \Vert _{H^1}^2, \quad \\&\Vert \textrm{d}g_\eta (\cdot , \varphi )[h] \Vert _{H^1} \lesssim \eta ^{S - \frac{1}{2}} \Vert h \Vert _{H^1} + \eta ^{- 4} \Vert \varphi \Vert _{H^1} \Vert h \Vert _{H^1}. \end{aligned} \end{aligned}$$
(ii) **(Estimates in** $H^s_\textbf{x} H^1_y$**).** Assume $\Vert \varphi \Vert _{s_0, 1}^{k_0,\upsilon } \leqslant 1$. Then there exists $\sigma > 0$ such that, for $S> s_0 + \sigma$ large enough and for any $s_0 \leqslant s \leqslant S- \sigma$, one has
  4.14 $$\begin{aligned} \Vert g_\eta (\cdot , \varphi ) \Vert _{s, 1}^{k_0,\upsilon }&\lesssim _s \eta ^{S - \frac{1}{2}}\Vert \varphi \Vert _{s, 1}^{k_0,\upsilon } + \eta ^{- (s + \sigma )} \Vert \varphi \Vert _{s_0, 1}^{k_0,\upsilon } \Vert \varphi \Vert _{s, 1}^{k_0,\upsilon }; \nonumber \\ \Vert \textrm{d}g_\eta (\cdot , \varphi )[h] \Vert _{s, 1}^{k_0,\upsilon }&\lesssim _s \eta ^{S - \frac{1}{2}}\Vert h\Vert _{s, 1}^{k_0,\upsilon } + \eta ^{- (s + \sigma )} \Vert \varphi \Vert _{s, 1}^{k_0,\upsilon } \Vert h \Vert _{s_0, 1}^{k_0,\upsilon } ; \nonumber \\ \Vert \textrm{d}^2 g_\eta (\cdot , \varphi )[h_1, h_2] \Vert _{s, 1}^{k_0,\upsilon }&\lesssim _s \eta ^{- (s + \sigma )}\big ( \Vert h_1 \Vert _{s_0, 1}^{k_0,\upsilon } \Vert h_2 \Vert _{s, 1}^{k_0,\upsilon } + \Vert h_1 \Vert _{s, 1}^{k_0,\upsilon } \Vert h_2 \Vert _{s_0, 1}^{k_0,\upsilon } \nonumber \\&\quad + \Vert \varphi \Vert _{s, 1}^{k_0,\upsilon } \Vert h_1 \Vert _{s_0, 1}^{k_0,\upsilon } \Vert h_2 \Vert _{s_0, 1}^{k_0,\upsilon } \big ). \end{aligned}$$

#### Proof

Proof of (*i*). By the definition of $f_\eta$, $g_\eta$ in (4.12), (4.13), it suffices to estimate $f_{p, \eta }$, $g_{p, \eta }$ for any $p = 1, \ldots , \kappa _0$. By (4.3) applied with $n = 0, 1$, one obtains that

4.15 $$\begin{aligned} \begin{aligned} \Vert f_{p, \eta } \Vert _{H^1} \leqslant \Vert F_{p,\eta }(\psi _{{\mathfrak {m}}} ) - F_{p,{\mathfrak {m}}}(\psi _{{\mathfrak {m}}} ) \Vert _{\mathcal {C}^1} \lesssim \eta ^{S + \frac{1}{2}} \end{aligned} \end{aligned}$$

which implies the claimed bound on $f_{p, \eta }$. In order to estimate $g_{p, \eta }$, we write

4.16 $$\begin{aligned} \begin{aligned} g_{p, \eta }(y, \varphi )&= \mathcal {I}_1 + \mathcal {I}_2 , \\ \mathcal {I}_1 (\varphi )&:= \big ( F_{p,\eta }'(\psi _{{\mathfrak {m}}}(y)) - F_{p,{\mathfrak {m}}}'(\psi _{{\mathfrak {m}}}(y)) \big ) {\varphi }, \quad \\ \mathcal {I}_2(\varphi )&:= \int _{0}^{1} (1-\varrho ) F_{p,\eta }''(\psi _{{\mathfrak {m}}}(y)+\varrho {\varphi }) \,\textrm{d}{\varrho }\, {\varphi }^2. \end{aligned} \end{aligned}$$

and we estimate $\mathcal {I}_1$ and $\mathcal {I}_2$ separately.

**Estimate of** $\mathcal {I}_1(\varphi )$. By the algebra property of $H_0^1$ and the estimate (4.3) applied with $n = 1, 2$, we have

$$\begin{aligned} \begin{aligned} \Vert \mathcal {I}_1(\varphi ) \Vert _{H^1}&\lesssim \Vert \big ( F_{p,\eta }'(\psi _{{\mathfrak {m}}}(y)) - F_{p,{\mathfrak {m}}}'(\psi _{{\mathfrak {m}}}(y)) \big ) \Vert _{H^1} \Vert \varphi \Vert _{H^1} \\&\lesssim \Vert \big ( F_{p,\eta }'(\psi _{{\mathfrak {m}}}(y)) - F_{p,{\mathfrak {m}}}'(\psi _{{\mathfrak {m}}}(y)) \big ) \Vert _{\mathcal {C}^1} \Vert \varphi \Vert _{H^1} {\mathop {\lesssim }\limits ^{(4.2)}} \eta ^{S - \frac{1}{2}} \Vert \varphi \Vert _{H^1}, \end{aligned} \end{aligned}$$

and similarly $\Vert \textrm{d}\mathcal {I}_1(\varphi ) [h] \Vert _{H^1} \lesssim \eta ^{S - \frac{1}{2}} \Vert h \Vert _{H^1}$, since the map $\varphi \mapsto \mathcal {I}_1(\varphi )$ is linear.

**Estimate of** $\mathcal {I}_2(\varphi )$. The differential of $\mathcal {I}_2$ is given by

4.17 $$\begin{aligned} \begin{aligned} \textrm{d}\mathcal {I}_2(\varphi )[h]&= 2 \int _{0}^{1} (1-\varrho ) F_{p,\eta }''(\psi _{{\mathfrak {m}}}(y)+\varrho {\varphi }) \,\textrm{d}{\varrho }\, {\varphi }h \\&\ \ \, + \int _{0}^{1} (1-\varrho ) F_{p,\eta }'''(\psi _{{\mathfrak {m}}}(y)+\varrho {\varphi }) \varrho \,\textrm{d}{\varrho }\, {\varphi }^2 h. \end{aligned} \end{aligned}$$

By Proposition 4.1-(*iii*), for $\Vert \varphi \Vert _{H^1} \leqslant 1$, a direct calculation shows that

$$\begin{aligned} \sup _{\varrho \in [0, 1]} \Vert F_{p,\eta }^{(k)}(\psi _{{\mathfrak {m}}}(y)+\varrho {\varphi }) \Vert _{H^1} \lesssim \Vert F \Vert _{\mathcal {C}^4} \lesssim \eta ^{- 4} \quad k = 2,3. \end{aligned}$$

The latter estimate, together with the algebra property of $H_0^1$ implies that

$$\begin{aligned} \begin{aligned} \Vert \mathcal {I}_2(\varphi ) \Vert _{H^1} \lesssim \eta ^{- 3} \Vert \varphi \Vert _{H^1}^2 , \quad \Vert \textrm{d}\mathcal {I}_2(\varphi )[h] \Vert _{H^1} \lesssim \eta ^{- 4} \Vert \varphi \Vert _{H^1} \Vert h \Vert _{H^1} \end{aligned} \end{aligned}$$

Proof of (*ii*). By (4.13), it is enough to estimate $g_{p, \eta }(y, \varphi )$ for any $p = 1, \ldots , \kappa _0$ and, according to (4.16), we estimate $\mathcal {I}_1$ and $\mathcal {I}_2$ separately.

**Estimate of** $\mathcal {I}_1(\varphi )$. By Lemma 2.1 and by applying again the estimate (4.3) with $n = 0, 1$, one obtains that

$$\begin{aligned} \begin{aligned} \Vert \mathcal {I}_1(\varphi ) \Vert _{s, 1}^{k_0,\upsilon }&= \Vert \big ( F_{p,\eta }'(\psi _{{\mathfrak {m}}}(y)) - F_{p,{\mathfrak {m}}}'(\psi _{{\mathfrak {m}}}(y)) \big ) {\varphi }\Vert _{s, 1}^{k_0,\upsilon } \\&\lesssim \Vert F_{p,\eta }'(\psi _{{\mathfrak {m}}}(y)) - F_{p,{\mathfrak {m}}}'(\psi _{{\mathfrak {m}}}(y)) \Vert _{H^1} \Vert \varphi \Vert _{s, 1}^{k_0, \upsilon } \\&\lesssim \Vert F_{p,\eta }'(\psi _{{\mathfrak {m}}}(y)) - F_{p,{\mathfrak {m}}}'(\psi _{{\mathfrak {m}}}(y)) \Vert _{\mathcal {C}^1} \Vert \varphi \Vert _{s, 1}^{k_0, \upsilon } \lesssim \eta ^{S - \frac{1}{2}} \Vert \varphi \Vert _{s, 1}^{k_0, \upsilon }. \end{aligned} \end{aligned}$$

The estimates for $\textrm{d}\mathcal {I}_1(\varphi )$ and $\textrm{d}^2 \mathcal {I}_1(\varphi )$ follows similarly since $\mathcal {I}_1$ is linear with respect to $\varphi$.

**Estimate of** $\mathcal {I}_2(\varphi )$. The first differential of $\mathcal {I}_2(\varphi )$ in (4.16) is given in (4.17), whereas the second differential has the form

$$\begin{aligned} \textrm{d}^2 \mathcal {I}_2(\varphi )[h_1, h_2]&= 2 \int _{0}^{1} (1-\varrho ) F_{p,\eta }''(\psi _{{\mathfrak {m}}}(y)+\varrho {\varphi }) \,\textrm{d}{\varrho }\, h_2 h_1\\  &\quad + 3 \int _{0}^{1} (1-\varrho ) F_{p,\eta }''' (\psi _{{\mathfrak {m}}}(y)+\varrho {\varphi }) \varrho \,\textrm{d}{\varrho }\, \varphi \,h_1 h_2 \\&\quad + \int _{0}^{1} (1-\varrho ) F_{p,\eta }^{(4)}(\psi _{{\mathfrak {m}}}(y)+\varrho {\varphi }) \varrho ^2 \,\textrm{d}{\varrho }\, {\varphi }^2 h_1 h_2 . \end{aligned}$$

By applying Proposition 4.1-(*iii*) and the composition lemma 2.2, for $\Vert \varphi \Vert _{s_0, 1}^{k_0, \upsilon } \leqslant 1$, some $\sigma > 0$, one has that for any $s_0 \leqslant s \leqslant S - \sigma$,

4.18 $$\begin{aligned} \sup _{\varrho \in [0, 1]} \Vert F_{p,\eta }^{(k)}(\psi _{{\mathfrak {m}}}(y)+\varrho {\varphi }) \Vert _{s, 1}^{k_0, \upsilon } \lesssim _s \eta ^{- (s + \sigma )} (1 + \Vert \varphi \Vert _{s, 1}^{k_0, \upsilon }), \quad k = 2,3,4. \nonumber \\ \end{aligned}$$

Then by the explicit expressions of $\mathcal {I}_2, \textrm{d}\mathcal {I}_2, \textrm{d}^2\mathcal {I}_2$, using the estimate (4.18), Lemma 2.1 and $\Vert \varphi \Vert _{s_0, 1}^{k_0, \upsilon } \leqslant 1$, one gets, for any $s_0 \leqslant s \leqslant S - \sigma$, the estimates

$$\begin{aligned} \begin{aligned} \Vert \mathcal {I}_2(\varphi ) \Vert _{s, 1}^{k_0,\upsilon }&\lesssim _s \eta ^{- (s + \sigma )} \Vert \varphi \Vert _{s_0, 1}^{k_0,\upsilon } \Vert \varphi \Vert _{s, 1}^{k_0,\upsilon }, \\ \Vert \textrm{d}\mathcal {I}_2(\varphi )[h] \Vert _{s, 1}^{k_0,\upsilon }&\lesssim _s \eta ^{- (s + \sigma )} \big ( \Vert h \Vert _{s_0, 1}^{k_0,\upsilon } \Vert \varphi \Vert _{s, 1}^{k_0,\upsilon } + \Vert h \Vert _{s_0, 1}^{k_0,\upsilon } \Vert \varphi \Vert _{s, 1}^{k_0,\upsilon } \big ) , \\ \Vert \textrm{d}^2 \mathcal {I}_2(\varphi )[h_1, h_2] \Vert _{s, 1}^{k_0,\upsilon }&\lesssim _s \eta ^{- (s + \sigma )}\big ( \Vert h_1 \Vert _{s_0, 1}^{k_0,\upsilon } \Vert h_2 \Vert _{s, 1}^{k_0,\upsilon } + \Vert h_1 \Vert _{s, 1}^{k_0,\upsilon } \Vert h_2 \Vert _{s_0, 1}^{k_0,\upsilon } \\&\quad \quad \quad \quad + \Vert \varphi \Vert _{s, 1}^{k_0,\upsilon } \Vert h_1 \Vert _{s_0, 1}^{k_0,\upsilon } \Vert h_2 \Vert _{s_0, 1}^{k_0,\upsilon } \big ). \end{aligned} \end{aligned}$$

By the previous arguments, one easily deduces the claimed bound (4.14). $\square$

It is actually possible to perform an affine transformation of the unknown in order to remove the forcing term $f_{\eta }(y)$ in (4.11).

#### Lemma 4.3

Let ${\overline{\eta }}\ll 1$ as in Proposition 4.1 and $S > 4$. Then, for any $\eta \in [0,{\overline{\eta }}]$, there exists a function $h_{\eta }(y) \in H_0^3[-1,1]$, even in *y* and with $h_{\eta }(0)=0$, such that $\Vert h_{\eta }\Vert _{H_y^3}\leqslant \eta ^S$ and

4.19 $$\begin{aligned} -\mathcal {L}_{{\mathfrak {m}}} h_{\eta }(y) - g_{\eta }(y,h_{\eta }(y)) = f_{\eta }(y). \end{aligned}$$

#### Proof

First, by (4.12), we note that $f_{\eta }(y)$ is even and that $f_{\eta }(0)=f_{\eta }(\pm 1)=0$. The same holds for $g_{\eta }(y,h(y))$ in (4.13), assuming $h(y)\equiv h_{\eta }(y)$ even in *y* and with $h(0)=h(\pm 1)=0$. Moreover, by Proposition 3.10 and the classical Sturm–Liouville theory for Schrödinger operators with smooth potentials, 0 is not an eigenvalue for $\mathcal {L}_{{\mathfrak {m}}}$ and the inverse operator $\mathcal {L}_{{\mathfrak {m}}}^{-1}:H_{0}^{1}[-1,1] \rightarrow H_{0}^{3}[-1,1]$ is a well defined smoothing operator. Therefore, we reformulate Equation  (4.19) as the fixed point equation

4.20 $$\begin{aligned} h(x) = T_{\eta }(h(x)), \quad T_{\eta }(h):= (-\mathcal {L}_{{\mathfrak {m}}})^{-1}\big ( f_{\eta }(y) + g_{\eta }(y,h) \big ). \end{aligned}$$

We define the domain,

$$\begin{aligned} B_{\eta } := \big \{ h\in H_{0}^{3}[-1,1] \, : \, h(0)=0, \ h(-y)=h(y),\ \Vert h \Vert _{H_y^3} \leqslant \eta ^S \big \}. \end{aligned}$$

By Proposition 4.1-(*ii*) and Lemma 4.2-(*i*), for any $h \in B_\eta$ and ${\widehat{h}} \in H_0^1([- 1, 1])$, with $\eta \ll 1$ small enough, we have

4.21 $$\begin{aligned} \begin{aligned} \Vert f_\eta \Vert _{H^1}&\lesssim \eta ^{S + \frac{1}{2}}, \quad \\ \Vert g_\eta (\cdot , h) \Vert _{H^1}&\lesssim \eta ^{S - \frac{1}{2}}\Vert h \Vert _{H^1} + \eta ^{- 3} \Vert h \Vert _{H^1}^2 \\&\lesssim \eta ^{2 S - \frac{1}{2}} + \eta ^{2 S - 3} \lesssim \eta ^{2 S - 3}, \\ \Vert \textrm{d}g_\eta (\cdot , h)[{\widehat{h}}] \Vert _{H^1}&\lesssim \eta ^{S - \frac{1}{2}} \Vert {\widehat{h}} \Vert _{H^1} + \eta ^{- 4} \Vert h \Vert _{H^1} \Vert {\widehat{h}} \Vert _{H^1} \\&\ \lesssim \big ( \eta ^{S - \frac{1}{2}} + \eta ^{S - 4} \big ) \Vert {\widehat{h}} \Vert _{H^1} \lesssim \eta ^{S - 4} \Vert {\widehat{h}} \Vert _{H^1} \end{aligned} \end{aligned}$$

By using that $\mathcal {L}_{{\mathfrak {m}}}^{-1} : H_{0}^{1}[-1,1] \rightarrow H_{0}^{3}[-1,1]$ is linear and continuous and by (4.21), one deduces that the map $T_\eta$ in (4.20) satisfies, for any $h \in B_\eta$ and ${\widehat{h}} \in H_0^3([- 1, 1])$,

$$\begin{aligned} \Vert T_\eta (h) \Vert _{H^3} \leqslant C( \eta ^{S + \frac{1}{2}} + \eta ^{2 S - 3}), \quad \Vert d T_\eta (h)[{\widehat{h}}] \Vert _{H^3} \leqslant C \eta ^{S - 4} \Vert {\widehat{h}}\Vert _{H^3} \end{aligned}$$

for some $C \geqslant 1$ independent of $\eta$. Hence, by the assumption $S>4$, with $\eta \ll 1$ small enough, the map $T_\eta : B_\eta \rightarrow B_\eta$ is a contraction, implying that there exists a unique solution $h\in B_{\eta }$ of the Equation (4.20) by a fixed point argument. $\square$

We introduce the rescaled variable $\zeta := \varepsilon ^{-1}\big ( \varphi (\textbf{x}, y) - h_\eta (y)\big )$. At this stage, we also link the parameters $\eta$ and $\varepsilon$ as

4.22 $$\begin{aligned} \eta := \varepsilon ^{\frac{1}{S}} \end{aligned}$$

where $S \gg 0$ is the smoothness of the nonlinearity $g_\eta$. Hence (4.11) in the new rescaled variable becomes

4.23 $$\begin{aligned} {\left\{ \begin{array}{ll} (\omega \cdot \partial _\textbf{x})^2\zeta -\mathcal {L}_{{\mathfrak {m}}}\zeta - \sqrt{\varepsilon } q_\varepsilon (y, \zeta )= 0, \quad (\textbf{x},y)\in {{\mathbb {T}}}^{\kappa _0} \times [-1,1], \\ \zeta (\textbf{x}, -1) = \zeta (\textbf{x}, 1) = 0 , \quad \omega \in {{\mathbb {R}}}^{\kappa _0}. \end{array}\right. } \end{aligned}$$

where

4.24 $$\begin{aligned} q_\varepsilon (y, \zeta ) := \varepsilon ^{- \frac{3}{2}}\big ( g_{\eta }(y,h_\eta (y) + \varepsilon \zeta ) - g_\eta (y, h_\eta (y)) \big ), \quad \eta = \varepsilon ^{\frac{1}{S}}. \end{aligned}$$

In the next lemma, we provide some estimates on the rescaled nonlinearity $q_{\varepsilon }$.

#### Lemma 4.4

Let $C_0 > 0$ and assume $\Vert \zeta \Vert _{s_0, 1}^{k_0, \upsilon } \leqslant C_0$. Let $S\geqslant 2(s_0+\sigma )$ with $\sigma >0$ as in Lemma 4.2-(*ii*). Then the rescaled nonlinearity $q_\varepsilon$ satisfies the following estimates. For any $s_0 \leqslant s \leqslant S/2 - \sigma$, one has

4.25 $$\begin{aligned} \begin{aligned} \Vert q_\varepsilon (\cdot , \zeta ) \Vert _{s, 1}^{k_0, \upsilon }&\lesssim _s \Vert \zeta \Vert _{s, 1}^{k_0, \upsilon } , \\ \Vert \textrm{d}q_\varepsilon (y, \zeta )[{\widehat{\zeta }}] \Vert _{s, 1}^{k_0, \upsilon }&\lesssim _s \Vert {\widehat{\zeta }} \Vert _{s, 1}^{k_0, \upsilon } + \Vert \zeta \Vert _{s, 1}^{k_0, \upsilon } \Vert {\widehat{\zeta }} \Vert _{s_0, 1}^{k_0, \upsilon } , \\ \Vert \textrm{d}^2 q_\varepsilon (\cdot , \zeta )[{\widehat{\zeta }}_1, {\widehat{\zeta }}_2] \Vert _{s, 1}^{k_0, \upsilon }&\lesssim _s \Vert {\widehat{\zeta }}_1 \Vert _{s_0, 1}^{k_0,\upsilon } \Vert {\widehat{\zeta }}_2 \Vert _{s, 1}^{k_0,\upsilon } + \Vert {\widehat{\zeta }}_1 \Vert _{s, 1}^{k_0,\upsilon } \Vert {\widehat{\zeta }}_2 \Vert _{s_0, 1}^{k_0,\upsilon } \\&\ \ + \Vert \zeta \Vert _{s, 1}^{k_0,\upsilon } \Vert {\widehat{\zeta }}_1 \Vert _{s_0, 1}^{k_0,\upsilon } \Vert {\widehat{\zeta }}_2 \Vert _{s_0, 1}^{k_0,\upsilon }. \end{aligned} \end{aligned}$$

#### Proof

We shall apply the estimates (4.14) in Lemma 4.2. We start by proving the second estimate in (4.25). One has

$$\begin{aligned} \textrm{d}q_\varepsilon (y, \zeta )[{\widehat{\zeta }}] = \varepsilon ^{- \frac{1}{2}} \textrm{d}g_{\eta }(y,h_\eta (y) + \varepsilon \zeta )[{\widehat{\zeta }}]. \end{aligned}$$

Hence, by the second estimate in (4.14), Lemma 4.3 and (4.22), one gets that for any $s_0 \leqslant s \leqslant S/2 - \sigma$,

$$\begin{aligned} \begin{aligned} \Vert \textrm{d}q_\varepsilon (y, \zeta )[{\widehat{\zeta }}]\Vert _{s, 1}^{k_0, \upsilon }&\lesssim _s \varepsilon ^{- \frac{1}{2}}\eta ^{S - \frac{1}{2}}\Vert {\widehat{\zeta }}\Vert _{s, 1}^{k_0,\upsilon } + \varepsilon ^{- \frac{1}{2}} \eta ^{- S/2} \Vert h_\eta + \varepsilon \zeta \Vert _{s, 1}^{k_0,\upsilon } \Vert {\widehat{\zeta }} \Vert _{s_0, 1}^{k_0,\upsilon } \\&\lesssim _s \varepsilon ^{- \frac{1}{2}}\eta ^{S - \frac{1}{2}}\Vert {\widehat{\zeta }}\Vert _{s, 1}^{k_0,\upsilon } + \varepsilon ^{- \frac{1}{2}} \eta ^{- S/2}\big ( \Vert h_\eta \Vert _{H^1} + \varepsilon \Vert \zeta \Vert _{s, 1}^{k_0,\upsilon } \big ) \Vert {\widehat{\zeta }} \Vert _{s_0, 1}^{k_0,\upsilon } \\&\lesssim _s \varepsilon ^{- \frac{1}{2}}\big ( \eta ^{S - \frac{1}{2}} + \eta ^{S/2} \big ) \Vert {\widehat{\zeta }}\Vert _{s, 1}^{k_0,\upsilon } + \varepsilon ^{\frac{1}{2}} \eta ^{- S/2} \Vert \zeta \Vert _{s, 1}^{k_0,\upsilon } \Vert {\widehat{\zeta }} \Vert _{s_0, 1}^{k_0,\upsilon } \\&\lesssim _s \varepsilon ^{- \frac{1}{2}} \eta ^{S/2} \Vert {\widehat{\zeta }}\Vert _{s, 1}^{k_0,\upsilon } + \varepsilon ^{\frac{1}{2}} \eta ^{- S/2} \Vert \zeta \Vert _{s, 1}^{k_0,\upsilon } \Vert {\widehat{\zeta }} \Vert _{s_0, 1}^{k_0,\upsilon } \\&\lesssim _s \varepsilon ^{- \frac{1}{2}}(\varepsilon ^{\frac{1}{S}})^{\frac{S}{2}} \Vert {\widehat{\zeta }}\Vert _{s, 1}^{k_0,\upsilon } + \varepsilon ^{\frac{1}{2}} (\varepsilon ^{\frac{1}{S}})^{- S/2} \Vert \zeta \Vert _{s, 1}^{k_0,\upsilon } \Vert {\widehat{\zeta }} \Vert _{s_0, 1}^{k_0,\upsilon } \\&\lesssim _s \Vert {\widehat{\zeta }}\Vert _{s, 1}^{k_0,\upsilon } + \Vert \zeta \Vert _{s, 1}^{k_0,\upsilon } \Vert {\widehat{\zeta }} \Vert _{s_0, 1}^{k_0,\upsilon } \end{aligned} \end{aligned}$$

which is the second estimate in (4.4). Therefore, the first estimate in (4.25) follows by (4.24), the mean value theorem and the second estimate in (4.4). We finally prove the third estimate in (4.25). The second derivative of $q_\varepsilon$ is given by

$$\begin{aligned} \textrm{d}^2 q_\varepsilon (\cdot , \zeta )[{\widehat{\zeta }}_1, {\widehat{\zeta }}_2] = \varepsilon ^{\frac{1}{2}} \textrm{d}^2 g_{\eta }(y,h_\eta (y) + \varepsilon \zeta )[{\widehat{\zeta }}_1, {\widehat{\zeta }}_2]. \end{aligned}$$

Hence, by the third estimate in (4.14), Lemma 4.3 and (4.22), for $\varepsilon \ll 1$ one gets

$$\begin{aligned} \begin{aligned} \Vert \textrm{d}^2 q_\varepsilon (\cdot , \zeta )[{\widehat{\zeta }}_1, {\widehat{\zeta }}_2] \Vert _{s, 1}^{k_0, \upsilon }&\lesssim _s \varepsilon ^{\frac{1}{2}} \eta ^{- \frac{S}{2}} \Big ( \Vert {\widehat{\zeta }}_1 \Vert _{s_0, 1}^{k_0,\upsilon } \Vert {\widehat{\zeta }}_2 \Vert _{s, 1}^{k_0,\upsilon } + \Vert {\widehat{\zeta }}_1 \Vert _{s, 1}^{k_0,\upsilon } \Vert {\widehat{\zeta }}_2 \Vert _{s_0, 1}^{k_0,\upsilon } \\&\qquad + \Vert h_\eta + \varepsilon \zeta \Vert _{s, 1}^{k_0,\upsilon } \Vert {\widehat{\zeta }}_1 \Vert _{s_0, 1}^{k_0,\upsilon } \Vert {\widehat{\zeta }}_2 \Vert _{s_0, 1}^{k_0,\upsilon } \Big ) \\&\lesssim _s \Vert {\widehat{\zeta }}_1 \Vert _{s_0, 1}^{k_0,\upsilon } \Vert {\widehat{\zeta }}_2 \Vert _{s, 1}^{k_0,\upsilon } + \Vert {\widehat{\zeta }}_1 \Vert _{s, 1}^{k_0,\upsilon } \Vert {\widehat{\zeta }}_2 \Vert _{s_0, 1}^{k_0,\upsilon } \\&\qquad + \Vert \zeta \Vert _{s, 1}^{k_0,\upsilon } \Vert {\widehat{\zeta }}_1 \Vert _{s_0, 1}^{k_0,\upsilon } \Vert {\widehat{\zeta }}_2 \Vert _{s_0, 1}^{k_0,\upsilon } \end{aligned} \end{aligned}$$

as claimed. The proof of the lemma is then concluded. $\square$

We now write the rescaled second order Equation (4.23) as a second order system. Let

4.26 $$\begin{aligned} \zeta _{1}(\textbf{x},y) := \zeta , \quad \zeta _{2} := \omega \cdot \partial _{\textbf{x}} \zeta (\textbf{x},y), \quad u : = (\zeta _1, \zeta _2) \end{aligned}$$

Hence, solving the Equation (4.23) is equivalent to solving the first order system in the variable $u = (\zeta _1, \zeta _2)$

4.27 $$\begin{aligned} {\left\{ \begin{array}{ll} \omega \cdot \partial _{\textbf{x}} u (\textbf{x},y) - J \nabla _u H_{\varepsilon }(u(\textbf{x},y))\! =\!0 , \ \ (\textbf{x},y)\in {{\mathbb {T}}}^{\kappa _0}\!\times \![-1,1], \ \omega \in {{\mathbb {R}}}^{\kappa _0} , \\ u(\textbf{x}, -1) = u (\textbf{x}, 1) = 0, \end{array}\right. } \end{aligned}$$

where $$J=\left( {\begin{matrix} 0 &  \textrm{Id}\\ -\textrm{Id} &  0 \end{matrix}}\right) $$ is the standard Poisson tensor and the Hamiltonian $H_\varepsilon$ is given by

4.28 $$\begin{aligned} \begin{aligned} H_{\varepsilon }(\zeta _1, \zeta _2)&:= \frac{1}{2}\int _{-1}^1\Big ( \zeta _{2}^2 - \zeta _{1}\mathcal {L}_{{\mathfrak {m}}}\zeta _{1} \Big )\,\textrm{d}{z} - \sqrt{\varepsilon }\int _{-1}^{1}Q_{\varepsilon }(y,\zeta _{1})\,\textrm{d}{y}, \end{aligned} \end{aligned}$$

with $(\partial _\psi Q_\varepsilon )(y,\psi )= q_\varepsilon (y, \psi )$. The symplectic 2-form induced by the Poisson tensor is given by

4.29 $$\begin{aligned} \mathcal {W}\Big ( \begin{pmatrix} \zeta _1 \\ \zeta _2 \end{pmatrix},\bigg ( \begin{matrix} {\widetilde{\zeta }}_1 \\ {\widetilde{\zeta }}_2 \end{matrix} \bigg ) \Big ) := \Big ( J^{-1} \begin{pmatrix} \zeta _1 \\ \zeta _2 \end{pmatrix},\bigg ( \begin{matrix} {\widetilde{\zeta }}_1 \\ {\widetilde{\zeta }}_2 \end{matrix} \bigg ) \Big )_{L^2} = -(\zeta _2,{\widetilde{\zeta }}_1)_{L^2} +(\zeta _1,{\widetilde{\zeta }}_2)_{L^2}, \nonumber \\ \end{aligned}$$

with $J^{-1}$ regarded as an operator acting on $L_0^2([-1,1])\times L_0^2([-1,1])$ into itself. The Hamiltonian field $X_{H_{\varepsilon }}(y,u):= J \nabla _u H_{\varepsilon }(y,u)$ is therefore characterized by the identity

$$\begin{aligned} \textrm{d}_u H_{\varepsilon }(u)[{{\widehat{u}}}] = \mathcal {W}( X_{H_{\varepsilon }}(u), {{\widehat{u}}})\quad \forall \, {{\widehat{u}}}\in L_0^2([-1,1])\times L_0^2([-1,1]). \end{aligned}$$

The "spatial phase space" $\mathcal {H}:= H_0^{2}([-1,1])\times L_0^2([-1,1])$ splits into two invariant subspaces for the Hamiltonian

$$\begin{aligned} H_0 (\zeta _1, \zeta _2) := \frac{1}{2}\int _{-1}^1\Big ( \zeta _{2}^2 - \zeta _{1}\mathcal {L}_{{\mathfrak {m}}}\zeta _{1} \Big )\,\textrm{d}{z} \end{aligned}$$

(namely (4.28) at $\varepsilon = 0$), that is, $\mathcal {H}= \mathcal {X}\oplus \mathcal {X}_\perp$, with

$$\begin{aligned} \mathcal {X}:=\textrm{span} \Big \{ \begin{pmatrix} \phi _{j,{\mathfrak {m}}}(y) \\ 0 \end{pmatrix}, \begin{pmatrix} 0 \\ \phi _{j,{\mathfrak {m}}}(y) \end{pmatrix} \, : \, j=1,...,\kappa _0 \Big \}, \end{aligned}$$

and

4.30 $$\begin{aligned} \mathcal {X}_\perp :=\Big \{ \sum _{j\geqslant \kappa _0+1}\begin{pmatrix} \alpha _j \\ \beta _j \end{pmatrix}\phi _{j,{\mathfrak {m}}}(y) \in \mathcal {H}\, : \, \alpha _j, \beta _j\in {{\mathbb {R}}}\Big \}, \end{aligned}$$

where $(\phi _{j,{\mathfrak {m}}})_{j\in {{\mathbb {N}}}}$ is the basis of eigenfunctions for the self-adjoint operator $\mathcal {L}_{{\mathfrak {m}}}$, see Proposition 3.10. In the following, we will denote by $\Pi ^\perp$ the projection on the invariant subspace $\mathcal {X}_{\perp }$ in (4.30). We note that the symmetry condition (1.20) translates in the unknown $\zeta =(\zeta _1,\zeta _2)$ as follows:

4.31 $$\begin{aligned} \zeta _1(\textbf{x}, y)\in \textrm{even}(\textbf{x})\textrm{even}(y), \quad \zeta _2(\textbf{x},y)\in \textrm{odd}(\textbf{x})\textrm{even}(x). \end{aligned}$$

### Linear Solutions Near the Shear Equilibrium

We want to study all the solutions of the linearized system around the stream function $\psi _{{\mathfrak {m}}}(y)$ at $\varepsilon = 0$. This amounts to solving the elliptic equation in (1.15) (without any quasi-periodic conditions in *x*), which is equivalent to the following first-order systems

4.32 $$\begin{aligned} {\left\{ \begin{array}{ll} \partial _x \zeta -\textbf{L}_{{\mathfrak {m}}}(\texttt{E}) \zeta = 0 , \\ \zeta ( x, -1) = \zeta (x, 1) = 0, \end{array}\right. } \quad \textbf{L}_{{\mathfrak {m}}}(\texttt{E}) :=\begin{pmatrix} 0 &  \quad \textrm{Id} \\ \mathcal {L}_{{\mathfrak {m}}}(\texttt{E}) &  \quad 0 \end{pmatrix}. \end{aligned}$$

For any ${\texttt{E}} \in [{\texttt{E}}_1, {\texttt{E}}_2]$. The spectrum of the operator $\textbf{L}_{{\mathfrak {m}}} = \textbf{L}_{{\texttt{m}}}({\texttt{E}})$ with Dirichlet boundary conditions is given by

$$\begin{aligned} \sigma (\textbf{L}_{{\mathfrak {m}}})= \big \{ \pm \sqrt{\mu _{j,{\mathfrak {m}}}} \,:\, j\in {{\mathbb {N}}}\big \} = \big \{ \pm \textrm{i}\lambda _{j,{\mathfrak {m}}} \,:\, j=1,...,\kappa _0 \big \} \cup \big \{ \pm \lambda _{j,{\mathfrak {m}}}\,:\, j\geqslant \kappa _0+1 \big \}, \end{aligned}$$

where $\{ \mu _{j, {\mathfrak {m}}}=\mu _{j, {\mathfrak {m}}}(\texttt{E}) \}_{j\in {{\mathbb {N}}}}$ are the eigenvalues of $\mathcal {L}_{{\mathfrak {m}}}$ as in Proposition 3.10. Solutions of (4.32) which satisfy (4.31) are given by

$$\begin{aligned} \begin{pmatrix} \zeta _1(x,y) \\ \zeta _2(x,y) \end{pmatrix}&= \sum _{j=1}^{\kappa _0}A_{j}\begin{pmatrix} \cos (\lambda _{j,{\mathfrak {m}}}(\texttt{E})x) \\ -\lambda _{j,{\mathfrak {m}}}\sin (\lambda _{j,{\mathfrak {m}}}(\texttt{E})x) \end{pmatrix}\phi _{j,{\mathfrak {m}}}(y)\\&\quad + \sum _{j\geqslant \kappa _0+1}B_{j}\begin{pmatrix} \cosh (\lambda _{j,{\mathfrak {m}}}(\texttt{E})x) \\ \lambda _{j,{\mathfrak {m}}}\sinh (\lambda _{j,{\mathfrak {m}}}(\texttt{E})x) \end{pmatrix}\phi _{j,{\mathfrak {m}}}(y), \end{aligned}$$

for constants $A_j,B_j\in {{\mathbb {R}}}$. We deduce that, when $B_j=0$ for any $j\geqslant \kappa _0+1$, there exist solutions of the linearized system at $\varepsilon = 0$, at the equilibrium that are periodic or quasi-periodic in *x* with at most $\kappa _0$ frequencies, depending on the non-resonance conditions between the linear frequencies $\vec {\omega }_{{\mathfrak {m}}}({\texttt{E}})$ in (1.18). The ultimate goal is to prove that, for amplitudes $0<A_1,...,A_{\kappa _0} \ll 1$ sufficiently small, close to the equilibrium $\psi _{{\mathfrak {m}}}(y)$ there exist stationary solutions to the nonlinear system (4.27) bifurcating from the quasi-periodic linear solutions above and still quasi-periodic in the space variable *x*, with frequency vectors $\omega$ close to the unperturbed linear frequency vector $\vec {\omega }_{{\mathfrak {m}}}({\texttt{E}})$ in (1.18). To impose non-resonance conditions on the desired frequency vector $\omega$ close to $\vec {\omega }_{{\mathfrak {m}}}(\texttt{E})$, we will not move the parameter $\texttt{E}$, but we argue as follows: we fix $\texttt{E}\in {\overline{\mathcal {K}}} \cap (\texttt{E}_1,\texttt{E}_2)$, with ${\overline{\mathcal {K}}}\subset [\texttt{E}_1,\texttt{E}_2]$ as in (1.19), and we consider an auxiliary parameter $\texttt{A}\in \mathcal {J}_{\varepsilon }(\texttt{E})$ as in (1.21). The perturbed frequency vector will depend on this parameter ${\texttt{A}}$, which will be used to impose the non-resonance conditions for the former.

### Action-Angle Coordinates on the Invariant Subspace $\mathcal {X}$

Functions in the “spatial phase space” $\mathcal {H}=\mathcal {X}\oplus \mathcal {X}_\perp$ are parametrized by

$$\begin{aligned} \zeta (y)=\begin{pmatrix} \zeta _1(y)\\ \zeta _2(y) \end{pmatrix}= \sum _{j=1}^{\kappa _0}\begin{pmatrix} a_j \\ b_j \end{pmatrix}\phi _{j,{\mathfrak {m}}}(y) +z(y), \end{aligned}$$

where $z\in \mathcal {X}_\perp$ and $(a_1,...,a_{\kappa _0},b_1,...,b_{\kappa _0})\in {{\mathbb {R}}}^{2\kappa _0}$ are coordinates on the $2\kappa _0$-dimensional invariant subspace $\mathcal {X}$. We introduce another set of coordinates on $\mathcal {X}$, the so called *action-angle variables*: for some normalizing constant $Z>0$, let

$$\begin{aligned} a_j: \sqrt{\frac{1}{Z}(I_j+\xi _j)} \cos (\theta _j), \quad b_j :=-\sqrt{\frac{1}{Z}(I_j+\xi _j)} \sin (\theta _j),\quad \xi _j>0 , \ |I_j| \ll \xi _j, \end{aligned}$$

where $I=(I_1,...,I_{\kappa _0})\in {{\mathbb {R}}}^{\kappa _0}$ and $\theta =(\theta _{1},...,\theta _{\kappa _0})\in {{\mathbb {T}}}^{\kappa _0}$. Therefore, the function $A:{{\mathbb {T}}}^{\kappa _0}\times {{\mathbb {R}}}^{\kappa _0}\times \mathcal {X}_\perp \rightarrow \mathcal {H}$, defined by

4.33 $$\begin{aligned} \begin{aligned} A(\theta ,I,z)&:= v^\intercal (\theta ,I)+z := \sum _{j=1}^{\kappa _0}\sqrt{\frac{1}{Z}}\begin{pmatrix} \sqrt{I_j+\xi _j}\cos (\theta _j) \\ -\sqrt{I_j+\xi _j}\sin (\theta _j) \end{pmatrix}\phi _{j,{\mathfrak {m}}} + z, \end{aligned}\qquad \end{aligned}$$

is a parametrization of the spatial phase space $\mathcal {H}$. The symplectic 2-form (4.29) reads in action-angle coordinates as

4.34 $$\begin{aligned} \mathcal {W}= \sum _{j=1}^{\kappa _0} (\textrm{d}\theta _j \wedge \textrm{d}I_j) \oplus \mathcal {W}|_{\mathcal {X}_\perp }. \end{aligned}$$

We also note that the 2-form $\mathcal {W}$ is exact, namely

4.35 $$\begin{aligned} \mathcal {W}=\textrm{d}\Lambda , \quad \text {where } \ \ \ \Lambda _{(\theta ,I,z)}[{\widehat{\theta }},{{\widehat{I}}},{{\widehat{z}}}]:= -\sum _{j=1}^{\kappa _0}I_j {\widehat{\theta }}_j +\tfrac{1}{2} (J^{-1} z,{{\widehat{z}}}) \end{aligned}$$

is the associated Liouville 1-form. Moreover, given a Hamiltonian $K:{{\mathbb {T}}}^{\kappa _0}\times {{\mathbb {R}}}^{\kappa _0}\times \mathcal {X}_\perp$, the associated Hamiltonian vector field, with respect to the symplectic 2-form (4.34), is defined by

4.36 $$\begin{aligned} X_K:= (\partial _I K, -\partial _\theta K, J\nabla _z K), \end{aligned}$$

where $\nabla _z K$ denotes the $L^2$-gradient of *K* with respect to $z\in \mathcal {X}_\perp$. Then, the equations in (4.27) (recall also the definition of $H_\varepsilon$ in (4.28)) becomes the Hamiltonian system in the action-angle coordinates $A(\theta ,I,z)$ generated by the Hamiltonian

4.37 $$\begin{aligned} \begin{aligned} \mathcal {H}_{\varepsilon }(\theta ,I,z)&:= H_{\varepsilon }(A(\theta ,I,z)) = \mathcal {N}_{{\mathfrak {m}}}(I,z)+ \sqrt{\varepsilon } (P_\varepsilon \circ A)(\theta ,I,z), \\ \mathcal {N}_{{\mathfrak {m}}}(I,z)&:= \vec {\omega }_{{\texttt{m}}}({\texttt{E}}) \cdot I +\tfrac{1}{2} (z,\big ( {\begin{matrix} -\mathcal {L}_{{\mathfrak {m}}}({\texttt{E}}) &  0 \\ 0 &  \textrm{Id} \end{matrix}} \big ) z)_{L^2}, \\ P_{\varepsilon }(\zeta _1)&:=- \int _{-1}^{1} Q_{\varepsilon }(y,\zeta _1)\,\textrm{d}{y}, \quad \text {where} \quad \partial _\psi Q_\varepsilon (y, \psi ) = q_\varepsilon (y, \psi ). \end{aligned} \end{aligned}$$

Now, for a fixed $\texttt{E}\in {\overline{\mathcal {K}}}$ as in (5.16), we write, for any auxiliary parameter $\texttt{A}\in \mathcal {J}_{\varepsilon }(\texttt{E})$, with $\mathcal {J}_{\varepsilon }(\texttt{E})$ as in (1.21),

$$\begin{aligned} \vec {\omega }_{{\texttt{m}}}({\texttt{E}}) \cdot I = \vec {\omega }_{{\texttt{m}}}({\texttt{A}}) \cdot I + \big ( \vec {\omega }_{{\texttt{m}}}({\texttt{E}}) - \vec {\omega }_{{\texttt{m}}}({\texttt{A}}) \big ) \cdot I. \end{aligned}$$

Therefore, we rewrite (4.37) as

4.38 $$\begin{aligned} \begin{aligned} \mathcal {H}_{\varepsilon }(\texttt{E};\theta ,I,z )&= \mathcal {N}_{{\mathfrak {m}}}(\texttt{A},\texttt{E};I,z )+ \sqrt{\varepsilon } \mathcal {P}_\varepsilon (\texttt{A},\texttt{E};\theta ,I,z), \\ \mathcal {N}_{{\mathfrak {m}}}(\texttt{A},\texttt{E};I,z)&:= \vec {\omega }_{{\texttt{m}}}({\texttt{A}}) \cdot I +\tfrac{1}{2} (z,\big ( {\begin{matrix} -\mathcal {L}_{{\mathfrak {m}}}({\texttt{E}}) &  0 \\ 0 &  \textrm{Id} \end{matrix}} \big ) z)_{L^2}, \\ \mathcal {P}_\varepsilon (\texttt{A},\texttt{E};\theta ,I,z)&:= P_\varepsilon (\texttt{E};A(\theta ,I,z)) + \tfrac{1}{\sqrt{\varepsilon }}\big ( \vec {\omega }_{{\texttt{m}}}({\texttt{E}}) - \vec {\omega }_{{\texttt{m}}}({\texttt{A}}) \big ) \cdot I . \end{aligned} \end{aligned}$$

We remark that the Hamiltonian $\mathcal {H}_{\varepsilon }$ does not globally depend on the auxiliary parameter $\texttt{A}\in \mathcal {J}_{\varepsilon }(\texttt{E})$. Note that, since the frequency map is analytic and $|{\texttt{A}} - {\texttt{E}}| \leqslant \sqrt{\varepsilon }$, we also have

4.39 $$\begin{aligned} \big |\partial _{{\texttt{A}}}^k \big (\vec {\omega }_{{\texttt{m}}}({\texttt{A}}) - \vec {\omega }_{{\texttt{m}}}({\texttt{E}}) \big ) \big | \lesssim _k \sqrt{\varepsilon } \quad \forall \, k\in {{\mathbb {N}}}_0 \ \Rightarrow \ | \vec {\omega }_{{\mathfrak {m}}}(\texttt{A}) - \vec {\omega }_{{\mathfrak {m}}}(\texttt{E}) |^{k_0,\upsilon } \lesssim \sqrt{\varepsilon }. \nonumber \\ \end{aligned}$$

This is actually crucial for considering the term $\big (\vec {\omega }_{{\texttt{m}}}({\texttt{E}}) - \vec {\omega }_{{\texttt{m}}}({\texttt{A}}) \big ) \cdot I$ as perturbative of size $O(\sqrt{\varepsilon })$ and it is the reason for which we choose the neighbourhood of ${\texttt{E}}$ of size $\sqrt{\varepsilon }$.

The Hamiltonian equations associated to $\mathcal {H}_\varepsilon$ become

4.40 $$\begin{aligned} {\left\{ \begin{array}{ll} \partial _x \theta -\vec {\omega }_{{\texttt{m}}}({\texttt{A}}) - \sqrt{\varepsilon }\partial _I \mathcal {P}_\varepsilon (\texttt{A},\texttt{E};\theta ,I,z)= 0 , \\ \partial _x I + \sqrt{\varepsilon } \partial _\theta \mathcal {P}_\varepsilon (\texttt{A},\texttt{E};\theta ,I,z) = 0 , \\ \partial _x z- \textbf{L}_{{\mathfrak {m}}}(\texttt{E}) z - \sqrt{\varepsilon } J \nabla _z \mathcal {P}_\varepsilon (\texttt{A},\texttt{E};\theta ,I,z) = 0 . \end{array}\right. } \end{aligned}$$

### Nash–Moser Theorem with Modified Hypothetical Conjugation

We look for an embedded invariant torus for the Hamiltonian $\mathcal {H}_{\varepsilon }$ of the form

$$\begin{aligned} i :{{\mathbb {T}}}^{\kappa _0}\rightarrow {{\mathbb {T}}}^{\kappa _0}\times {{\mathbb {R}}}^{\kappa _0}\times \mathcal {X}_\perp , \quad \textbf{x}\mapsto i (\textbf{x}):=(\theta (\textbf{x}),I(\textbf{x}),z(\textbf{x})) \end{aligned}$$

filled with quasi-periodic solutions with Diophantine frequency $\omega \in {{\mathbb {R}}}^{\kappa _0}$. The periodic component of the embedded torus is given by

4.41 $$\begin{aligned} {\mathfrak {I}}(\textbf{x}):= i(\textbf{x})- (\textbf{x},0,0):= (\Theta (\textbf{x}),I(\textbf{x}),z(\textbf{x})), \quad \Theta (\textbf{x}):= \theta (\textbf{x})-\textbf{x}, \end{aligned}$$

The expected quasi-periodic solution of the Hamiltonian equations (4.40) will have a slightly shifted frequency vector close to the unperturbed frequency vector $\vec {\omega }_{{\mathfrak {m}}}(\texttt{E})$ in (1.18). The strategy that we implement here is a modification of the Théorème de conjugaison hypothétique of Herman presented in [23], see also [7]. Recalling that we fixed $\texttt{E}\in {\overline{\mathcal {K}}}$ in (1.19), and therefore it will not be moved as a parameter in the following, we consider the modified Hamiltonian, with $\alpha \in {{\mathbb {R}}}^{\kappa _0}$,

4.42 $$\begin{aligned} \mathcal {H}_{\varepsilon , \alpha } = \mathcal {H}_{\varepsilon ,\alpha }(\texttt{A}) :=\mathcal {N}_{\alpha } + \sqrt{\varepsilon } \,\mathcal {P}_{\varepsilon }(\texttt{A}), \quad \mathcal {N}_{\alpha }:= \alpha \cdot I +\tfrac{1}{2} \left( z,\left( {\begin{matrix} -\mathcal {L}_{{\mathfrak {m}}}(\texttt{E}) &  0 \\ 0 &  \textrm{Id} \end{matrix}} \right) z\right) _{L^2}. \nonumber \\ \end{aligned}$$

We look for zeroes of the nonlinear operator

4.43 $$\begin{aligned} \begin{aligned} \mathcal {F}({{\mathfrak {I}}}, \alpha ) \equiv \mathcal {F}(i,\alpha )&:= \mathcal {F}(\omega , {\texttt{A}}, \varepsilon ;i ,\alpha ) := \omega \cdot \partial _\textbf{x}i(\textbf{x}) - X_{\mathcal {H}_{\varepsilon , \alpha }}(i(\textbf{x})) \\&:= \begin{pmatrix} \omega \cdot \partial _\textbf{x}\theta (\textbf{x}) &  - \alpha - \sqrt{\varepsilon } \partial _I \mathcal {P}_\varepsilon (\texttt{A};i(\textbf{x})) \\ \omega \cdot \partial _\textbf{x}I(\textbf{x}) &  +\sqrt{\varepsilon } \partial _\theta \mathcal {P}_\varepsilon (\texttt{A};i(\textbf{x})) \\ \omega \cdot \partial _\textbf{x}z(\textbf{x})&  -\textbf{L}_{{\mathfrak {m}}}(\texttt{E}) z(\textbf{x}) - \sqrt{\varepsilon } J \nabla _z \mathcal {P}_\varepsilon (\texttt{A};i(\textbf{x})) \end{pmatrix}. \end{aligned} \nonumber \\ \end{aligned}$$

The parameters of the problem are $\lambda = (\omega , {\texttt{A}}) \in {{\mathbb {R}}}^{\kappa _0} \times \mathcal {J}_\varepsilon ({\texttt{E}}) \subset {{\mathbb {R}}}^{\kappa _0} \times {{\mathbb {R}}}$, whereas the unknowns of the problem are $\alpha$ and the periodic component of the torus embedding ${{\mathfrak {I}}}$. Solutions of the Hamiltonian equations (4.40) are recovered by setting $\alpha =\vec {\omega }_{{\mathfrak {m}}}(\texttt{A})$. The Hamiltonian $\mathcal {H}_{\varepsilon , \alpha }$ is invariant under the involution $\vec {\mathcal {S}}$, namely

4.44 $$\begin{aligned} \mathcal {H}_{\varepsilon , \alpha } \circ \vec {\mathcal {S}} = \mathcal {H}_{\varepsilon , \alpha }, \end{aligned}$$

where $\vec {\mathcal {S}}$ is the involution defined in (2.12), (2.11). We look for a reversible torus embedding $\textbf{x}\mapsto i (\textbf{x}) = ( \theta (\textbf{x}), I(\textbf{x}), w(\textbf{x}))$, namely satisfying

4.45 $$\begin{aligned} \vec {\mathcal {S}} i(\textbf{x})= i(-\textbf{x}) . \end{aligned}$$

Recalling (4.30), let $H_\bot := \textrm{span}\big \{ \phi _{j, {\texttt{m}}} : j \geqslant \kappa _0 + 1 \big \}$ and, for any $s, \rho \geqslant 0$, we define

4.46 $$\begin{aligned} H^{s, \rho }_\bot&:= H^s({{\mathbb {T}}}^{\kappa _0}, H_0^\rho ([- 1, 1] )\cap H_\bot ) \nonumber \\&\equiv \Big \{ u(\textbf{x}, y) = \sum _{\ell \in {{\mathbb {Z}}}^{\kappa _0}} \sum _{j \geqslant \kappa _0 + 1} u_{\ell , j} e^{\textrm{i}\ell \cdot \textbf{x}} \phi _{j, {\texttt{m}}}(y) \quad \text {with} \quad \Vert u \Vert _{s, \rho }^{k_0,\upsilon } < + \infty \Big \}.\nonumber \\ \end{aligned}$$

Then, we set

4.47 $$\begin{aligned} \mathcal {X}^s_\bot := H^{s, 3}_\bot \times H^{s, 1}_\bot , \quad \mathcal {Y}^s_\bot := H^{s, 1}_\bot \times H^{s, 1}_\bot , \end{aligned}$$

with corresponding norms, for $z=(z_1,z_2)$,

$$\begin{aligned} \Vert z \Vert _{\mathcal {X}^s_\bot }^{k_0, \upsilon } := \Vert z_1 \Vert _{s, 3}^{k_0, \upsilon } + \Vert z_2 \Vert _{s, 1}^{k_0, \upsilon }, \quad \Vert z \Vert _{\mathcal {Y}^s_\bot }^{k_0, \upsilon } := \Vert z_1 \Vert _{s, 1}^{k_0, \upsilon } + \Vert z_2 \Vert _{s, 1}^{k_0, \upsilon }, \end{aligned}$$

where the norms $\Vert \,\cdot \,\Vert _{s,\rho }^{k_0,\upsilon }$ are defined in (2.1)–(2.2). We also define the spaces

4.48 $$\begin{aligned} \mathcal {X}^s := H^s({{\mathbb {T}}}^{\kappa _{0}}) \times H^s({{\mathbb {T}}}^{\kappa _{0}}) \times \mathcal {X}^s_\bot , \quad \mathcal {Y}^s := H^s({{\mathbb {T}}}^{\kappa _{0}}) \times H^s({{\mathbb {T}}}^{\kappa _{0}}) \times \mathcal {Y}^s_\bot , \nonumber \\ \end{aligned}$$

with corresponding norms, for ${{\mathfrak {I}}} = (\Theta , I, z)$,

$$\begin{aligned} \Vert {{\mathfrak {I}}} \Vert _{\mathcal {X}^s}^{k_0, \upsilon } := \Vert \Theta \Vert _s^{k_0, \upsilon } + \Vert I \Vert _s^{k_0, \upsilon } + \Vert z \Vert _{\mathcal {X}^s_\bot }^{k_0, \upsilon }, \quad \Vert {{\mathfrak {I}}} \Vert _{\mathcal {Y}^s}^{k_0, \upsilon } := \Vert \Theta \Vert _s^{k_0, \upsilon } + \Vert I \Vert _s^{k_0, \upsilon } + \Vert z \Vert _{\mathcal {Y}^s_\bot }^{k_0, \upsilon }. \end{aligned}$$

Note that

4.49 $$\begin{aligned} \Vert \cdot \Vert _{\mathcal {Y}^s}^{k_0,\upsilon } \leqslant \Vert \cdot \Vert _{\mathcal {X}^s}^{k_0,\upsilon }, \quad \forall \,s \geqslant 0, \end{aligned}$$

and that, for $s \geqslant 0$, the nonlinear map $\mathcal {F}$ maps $\mathcal {X}^{s + 1} \times {{\mathbb {R}}}^{\kappa _{0}}$ into $\mathcal {Y}^s$. We fix

4.50 $$\begin{aligned} k_0:=m_0+2, \end{aligned}$$

where $m_0$ is the index of non-degeneracy provided in Proposition 5.6, which only depends on the linear unperturbed frequencies. Thus $k_0$ is considered as an absolute constant and we will often omit to explicitly write the dependence of the various constants with respect to $k_0$. Each frequency vector $\omega =(\omega _1,...,\omega _{\kappa _0})$ will belong to a $\varrho$-neighbourhood (independent of $\varepsilon$)

4.51 $$\begin{aligned} {\mathtt {\Omega }}:=\Big \{ \omega \in {{\mathbb {R}}}^{\kappa _0} \,:\, {{\,\textrm{dist}\,}}\Big (\omega ,\vec {\omega }_{{\mathfrak {m}}}(\mathcal {J}_\varepsilon ({\texttt{E}})) \Big )<\varrho \Big \}, \quad \varrho >0, \end{aligned}$$

where $\vec {\omega }_{{\mathfrak {m}}}(\mathcal {J}_\varepsilon ({\texttt{E}})) = \big \{ \vec {\omega }_{\mathfrak {m}}({\texttt{A}}) : {\texttt{A}} \in \mathcal {J}_\varepsilon ({\texttt{E}}) \big \}$ is the range of the unperturbed linear frequency map ${\texttt{A}} \mapsto \vec {\omega }_{{\mathfrak {m}}}({\texttt{A}})$ defined in (1.18), restricted to the interval $\mathcal {J}_\varepsilon ({\texttt{E}})$ in (1.21).

#### Theorem 4.5

(Nash–Moser) Let $\kappa _0\in {{\mathbb {N}}}$ be fixed as for Proposition 3.10. Let $\tau \geqslant 1$. There exist positive constants $\textrm{a}_0,\varepsilon _0,C$ depending on $\kappa _0,k_0,\tau$ such that, for all $\upsilon =\varepsilon ^{\textrm{a}}$, $\textrm{a}\in (0,\textrm{a}_0)$, and for all $\varepsilon \in (0,\varepsilon _0)$, there exist a $k_0$-times differentiable function

4.52 $$\begin{aligned} \begin{aligned}&\alpha _{\infty }: {{\mathbb {R}}}^{\kappa _0} \times \mathcal {J}_\varepsilon ({\texttt{E}}) \rightarrow {{\mathbb {R}}}^{\kappa _0}, \quad (\omega , {\texttt{A}}) \mapsto \alpha _{\infty }(\omega , {\texttt{A}}) : = \omega +r_{\varepsilon }(\omega , {\texttt{A}}) , \\&|r_{\varepsilon }|^{k_0,\upsilon }\leqslant C\sqrt{\varepsilon } \upsilon ^{-1}, \end{aligned} \end{aligned}$$

and a family of reversible embedded tori $i_{\infty }$ defined for all $(\omega , {\texttt{A}})\in {{\mathbb {R}}}^{\kappa _0} \times \mathcal {J}_\varepsilon ({\texttt{E}})$ satisfying (4.45) and

4.53 $$\begin{aligned} \Vert i_{\infty }(\textbf{x})-(\textbf{x},0,0) \Vert _{\mathcal {X}^{s_0}}^{k_0,\upsilon } \leqslant C\sqrt{\varepsilon } \upsilon ^{-1}, \end{aligned}$$

such that, for all $(\omega , {\texttt{A}}) \in \texttt{G}^\upsilon \times \mathcal {J}_\varepsilon ({\texttt{E}})$ where the Cantor set $\texttt{G}^\upsilon$ is defined as

4.54 $$\begin{aligned} \texttt{G}^\upsilon :=\Big \{ \omega \in {\mathtt {\Omega }}\, : \, |\omega \cdot \ell | \geqslant \upsilon \mathinner {\langle {\ell }\rangle }^{-\tau }, \ \forall \,\ell \in {{\mathbb {Z}}}^{\kappa _0}\setminus \{0\} \Big \}\subset {{\mathbb {R}}}^{\kappa _0}, \end{aligned}$$

the function $i_{\infty }(\textbf{x}):= i_{\infty }(\omega ,\varepsilon ;\textbf{x})$ is a solution of

$$\begin{aligned} \mathcal {F}(\omega , {\texttt{A}}, \varepsilon ; i_{\infty },\alpha _{\infty }(\omega , {\texttt{A}})) =0. \end{aligned}$$

As a consequence, each embedded torus $\textbf{x}\mapsto i_{\infty }(\textbf{x})$ is invariant for the Hamiltonian vector field $X_{\mathcal {H}_{\varepsilon , \alpha _{\infty }(\omega , {\texttt{A}})}}$ and it is filled by quasi-periodic solutions with frequency $\omega$.

The following theorem will be proved in Sect. 8. The Diophantine condition in (4.54) is verified for most of the parameters, see Theorem 6.1.

## Transversality of the Linear Frequencies

In this section we apply the KAM theory approach of Arnold and Rüssmann (see [44]), extended to PDEs in [12, 4], in order to deal with the linear frequencies $\lambda _{j,{\mathfrak {m}}}(\texttt{E})$ defined in Proposition 3.10. We first give the following definition.

### Definition 5.1

A function $f=(f_1,\dots ,f_{\kappa _0}):[\texttt{E}_1,\texttt{E}_2]\rightarrow {{\mathbb {R}}}^{\kappa _0}$ is *non-degenerate* if, for any $c\in {{\mathbb {R}}}^{\kappa _0}\setminus \{0\}$, the scalar function $f\cdot c$ is not identically zero on the whole parameter interval $[\texttt{E}_1,\texttt{E}_2]$.

We recall the vector of the linear frequencies in (1.18), as ${\mathfrak {m}}\rightarrow \infty$,

5.1 $$\begin{aligned} \vec {\omega }_{{\mathfrak {m}}}(\texttt{E}) := \big ( \lambda _{1,{\mathfrak {m}}}(\texttt{E}),..., \lambda _{\kappa _0,{\mathfrak {m}}}(\texttt{E})\big ) \rightarrow \vec {\omega }_{\infty }(\texttt{E}) := \big ( \lambda _{1,\infty }(\texttt{E}),..., \lambda _{\kappa _0,\infty }(\texttt{E}) \big ) \in {{\mathbb {R}}}^{\kappa _0},\nonumber \\ \end{aligned}$$

where $\lambda _{j,\infty }(\texttt{E}) \in (0,\texttt{E})$ is a zero of the secular equation, recalling (3.44),

5.2 $$\begin{aligned} {\mathfrak {F}}(\lambda ):= \lambda \cos \big (\texttt{r}\sqrt{\texttt{E}^2-\lambda ^2}\big )\coth ((1-\texttt{r})\lambda ) - \sqrt{\texttt{E}^2-\lambda ^2}\sin \big (\texttt{r}\sqrt{\texttt{E}^2-\lambda ^2}\big ) = 0 , \nonumber \\ \end{aligned}$$

for any $$j=1,...,\kappa _0$$.

### Remark 5.2

All the frequencies involved are analytic with respect to the parameter $\texttt{E}\in [\pi (\kappa _{0}+\tfrac{1}{4}),\infty )$. Indeed, the frequencies $(\lambda _{j, \infty }(\texttt{E}))_{j=1}^{\kappa _0}$ are analytic because defined as implicit zeroes of the analytic function (5.2), whereas the frequencies $(\lambda _{j,{\mathfrak {m}}}(\texttt{E}))_{j=1}^{\kappa _0}$ are analytic because $-\lambda _{j,{\mathfrak {m}}}^2(\texttt{E})$ are the negative eigenvalues of the Schrödinger operator $\mathcal {L}_{{\mathfrak {m}}}=-\partial _y^2 +Q_{{\mathfrak {m}}}(y)$ with the analytic potential $Q_{{\mathfrak {m}}}(y):=Q_{{\mathfrak {m}}}(\texttt{E},\texttt{r};y)$ under the analytic constrain in (1.4) (for the physical problem of the channel, we have $\texttt{r}\in (0,1]$, from where follows the threshold $\texttt{E}\geqslant (\kappa _{0}+\tfrac{1}{4})\pi$, otherwise arbitrary).

We prove the non-degeneracy of the vector $\vec {\omega }_{{\mathfrak {m}}}(\texttt{E})$ by first showing that the limit vector $\vec {\omega }_{\infty }(\texttt{E})$ is non-degenerate and then arguing by perturbation. To do so, the key tool is an explicit asymptotic expansion for the frequencies $(\lambda _{j,\infty }(\texttt{E}))_{j=1}^{\kappa _0}$.

### Lemma 5.3

For any $j=1,...,\kappa _{0}$, let $\lambda _{j,\infty }=\lambda _{j,\infty }(\texttt{E})\in (0,\texttt{E})$ be a zero of (5.2). Then, we have the asymptotic expansion

5.3 $$\begin{aligned} \begin{aligned}&\lambda _{j,\infty }(\texttt{E}) = \texttt{E}\cos \Big ( \pi \big ( \alpha _0(j) + \alpha _2(j) \beta _{j}(\texttt{E})^2 + o(\beta _{j}(\texttt{E})^3) \big ) \Big ), \ \texttt{E}\rightarrow +\infty , \\&\beta _{j}(\texttt{E}) := \exp (((\kappa _0+\tfrac{1}{4})\pi -\texttt{E})\cos (\pi \alpha _0(j))), \end{aligned} \end{aligned}$$

with $\alpha _2(j)=\alpha _2(\alpha _0(j))\in {{\mathbb {R}}}\setminus \{0\}$, where $\alpha _0(j)\in (0,\frac{1}{2})$ is a zero of the equation

5.4 $$\begin{aligned} \sin (\pi \alpha _0(j)) = \frac{j-\frac{1}{2} - \alpha _0(j)}{\kappa _0+\frac{1}{4}}. \end{aligned}$$

### Proof

For sake of simplicity in the notation, in the following proof we will omit to write the explicit dependence of the fixed $j=1,...,\kappa _{0}$ when not needed. Therefore, let $\lambda _{j, \infty }(\texttt{E})\equiv \lambda (\texttt{E}) \in (0,\texttt{E})$, which implies $\varsigma (\lambda (\texttt{E})):=\sqrt{(\texttt{E}- \lambda (\texttt{E})^2)}\in (0,\texttt{E})$. We make the following change of variables

$$\begin{aligned} \lambda (\texttt{E}):= \texttt{E}\cos (\pi \alpha (\texttt{E})), \quad \varsigma (\lambda (\texttt{E})) = \texttt{E}\sin (\pi \alpha (\texttt{E})) , \quad \alpha (\texttt{E}) \in (0,\tfrac{1}{2}). \end{aligned}$$

Recalling the constrain $\texttt{E}\texttt{r}= (\kappa _0+\tfrac{1}{4})\pi$ in (1.4), we have that any zero $\lambda (\texttt{E})$ of (5.2) in $(0,\texttt{E})$ correspond to a solution $\alpha (\texttt{E})$ of the following equation

5.5 $$\begin{aligned}&\cos \big (\pi \alpha (\texttt{E}) +(\kappa _0+\tfrac{1}{4})\pi \sin (\pi \alpha (\texttt{E}))\big ) \nonumber \\&\quad = \cos (\pi \alpha (\texttt{E})) \cos \big ((\kappa _0+\tfrac{1}{4})\pi \sin (\pi \alpha (\texttt{E}))\big ) \big (1- \coth \big ((\texttt{E}-(\kappa _0+\tfrac{1}{4})\pi )\cos (\pi \alpha (\texttt{E}))\big ) \big ) . \end{aligned}$$

We search for solutions of the form

$$\begin{aligned} \alpha (\texttt{E}) = \alpha _0 + \alpha _1 \beta (\texttt{E}) +\alpha _2\beta (\texttt{E})^2+ \texttt{g}(\texttt{E}), \quad \texttt{g}(\texttt{E}) = o(\beta (\texttt{E})^3) , \quad \texttt{E}\rightarrow +\infty , \end{aligned}$$

where $\beta (\texttt{E})$ is defined in (5.3). By the expansion

$$\begin{aligned} 1 - \coth (z) = 1 - \frac{1+e^{-2z}}{1-e^{-2z}} = -2 \sum _{n=1}^{\infty } e^{-2nz}, \quad \forall \,z >0, \end{aligned}$$

we note that, in the regime $\texttt{E}\rightarrow \infty$,

$$\begin{aligned}&1-\coth \big ((\texttt{E}-(\kappa _0+\tfrac{1}{4})\pi )\cos (\pi \alpha (\texttt{E}))\big )\\  &\quad = -2 \sum _{n=1}^{\infty } \exp \big ( 2n((\kappa _0+\tfrac{1}{4})\pi -\texttt{E})\cos (\pi \alpha (\texttt{E})) \big ) \\&\quad = -2\sum _{n=1}^{\infty } \exp \Big (2n((\kappa _0+\tfrac{1}{4})\pi -\texttt{E})\big (\cos (\pi \alpha _0) + o(\beta (\texttt{E})) \big )\Big ) \\&\quad = -2 \sum _{n=1}^{\infty } (\beta (\texttt{E}))^{2n} \exp \big (2n((\kappa _0+\tfrac{1}{4})\pi -\texttt{E}) o(\beta (\texttt{E}))\big ) \\&\quad = -2 (\beta (\texttt{E}))^{2} - \sum _{n=2}^{\infty } (\beta (\texttt{E}))^{2n} \\&\quad + 2 \sum _{n=1}^{\infty } (\beta (\texttt{E}))^{2n} \Big ( 1- \exp \big (2n((\kappa _0+\tfrac{1}{4})\pi -\texttt{E}) o(\beta (\texttt{E}))\big ) \Big ). \end{aligned}$$

We obtain that

5.6 $$\begin{aligned} 1-\coth \big ((\texttt{E}-(\kappa _0+\tfrac{1}{4})\pi )\cos (\pi \alpha (\texttt{E}))\big ) = - 2 (\beta (\texttt{E}))^{2} + o\big ( \texttt{E}(\beta (\texttt{E}))^3 \big ), \quad \texttt{E}\rightarrow \infty . \nonumber \\ \end{aligned}$$

Since we are interested in the first two powers of $\beta (\texttt{E})$ in the expansion of (5.5), it means that the other two factors on the right hand side of (5.5) contribute only with their zeroth orders. We now compute the first two orders of the left hand side of (5.5). We have

$$\begin{aligned} \begin{aligned} \alpha (\texttt{E})&+(\kappa _0+\tfrac{1}{4})\sin (\pi \alpha (\texttt{E})) = \alpha _0+(\kappa _0+\tfrac{1}{4})\sin (\pi \alpha _0)\\  &+\alpha _1(1+(\kappa _0+\tfrac{1}{4})\pi \cos (\pi \alpha _0)) \beta (\texttt{E})\\&+\big ( \alpha _2 (1+(\kappa _0+\tfrac{1}{4})\pi \cos (\pi \alpha _0)) -\tfrac{\pi ^2}{2}(\kappa _0+\tfrac{1}{4})\alpha _1^2 \sin (\pi \alpha _0) \big )(\beta (\texttt{E}))^2 + ... \end{aligned} \end{aligned}$$

where the dots stand for higher order terms. It follows that

5.7 $$\begin{aligned}&\cos \big ( \pi \alpha (\texttt{E}) +(\kappa _0+\tfrac{1}{4}) \pi \sin (\pi \alpha (\texttt{E})) \big ) = \cos (\pi (\alpha _0+(\kappa _0+\tfrac{1}{4})\sin (\pi \alpha _0))) \nonumber \\&-\pi \sin (\pi (\alpha _0+(\kappa _0+\tfrac{1}{4})\sin (\pi \alpha _0))) \Big ( \alpha _1(1+(\kappa _0+\tfrac{1}{4})\pi \cos (\pi \alpha _0)) \beta (\texttt{E}) \nonumber \\&+\big ( \alpha _2 (1+(\kappa _0+\tfrac{1}{4})\pi \cos (\pi \alpha _0)) -\tfrac{\pi ^2(\kappa _0+\tfrac{1}{4})}{2}\alpha _1^2 \sin (\pi \alpha _0) \big )(\beta (\texttt{E}))^2 + ... \Big )\nonumber \\&-\tfrac{\pi ^2}{2}\cos (\pi (\alpha _0+(\kappa _0+\tfrac{1}{4})\sin (\pi \alpha _0)))\Big ( \alpha _1(1+(\kappa _0+\tfrac{1}{4})\pi \cos (\pi \alpha _0))\beta (\texttt{E}) +... \Big ) +... \,. \end{aligned}$$

The zeroth order term *O*(1) in (5.5) comes only from its left hand side. Therefore we impose $\alpha _0$ to solve

$$\begin{aligned} \cos (\pi (\alpha _0+(\kappa _0+\tfrac{1}{4})\sin (\pi \alpha _0))) = 0. \end{aligned}$$

It follows that $\alpha _0=\alpha _0(j)\in (0,\frac{1}{2})$ solves, for $j=1,...,\kappa _0$, the implicit Equation (5.4). Furthermore, we note that

5.8 $$\begin{aligned} \sin \big (\pi (\alpha _0(j)+(\kappa _0+\tfrac{1}{4})\sin (\pi \alpha _0(j)))\big ) = (-1)^{j-1} , \quad \forall \,j=1,...,\kappa _0. \end{aligned}$$

Also the contribution to the first order term $O(\beta (\texttt{E}))$ in (5.5) comes only from its left hand side. Looking at (5.7), together with (5.8), we impose

5.9 $$\begin{aligned} (-1)^{j-1}\pi \alpha _1 (1+(\kappa _0+\tfrac{1}{4})\pi \cos (\pi \alpha _0(j))) = 0 \quad \Rightarrow \quad \alpha _1 =0. \end{aligned}$$

Second order terms $O((\beta (\texttt{E}))^2)$ appear on both sides of (5.5). By (5.5), (5.6), (5.7), (5.8) and (5.9), we impose, for any $j=1,...,\kappa _0$,

$$\begin{aligned}  &   (-1)^{j-1} \alpha _2\pi (1+(\kappa _0+\tfrac{1}{4})\pi \cos (\pi \alpha _0(j)))\\    &   \quad = -2\cos (\pi \alpha _0(j)) \cos ((\kappa _0+\tfrac{1}{4})\pi \sin (\pi \alpha _0(j))). \end{aligned}$$

This linear equation uniquely defines $\alpha _2(j)= \alpha _2(\alpha _0(j))\ne 0$ for any $j=1,...,\kappa _0$. This concludes the proof. $\square$

We can now prove that the following proposition.

### Proposition 5.4

(Non-degeneracy for $\vec {\omega }_{\infty }(\texttt{E})$). The vector $\vec {\omega }_{\infty }(\texttt{E})$ in (5.1) is non-degenerate in the interval $[\texttt{E}_1,\texttt{E}_2]$, assuming $\texttt{E}_1\gg 1$ sufficiently large.

### Proof

We argue by contradiction. Assume, therefore, that there exists a nontrivial vector $c=(c_1,...,c_{\kappa _0})\in {{\mathbb {R}}}^{\kappa _0}\setminus \{0\}$ such that $c\cdot \vec {\omega }_{\infty }(\texttt{E}) =0$ on $[\texttt{E}_1,\texttt{E}_2]$. By Remark 5.2, the zeroes of the analytic function (5.2) are analytic in the domain $[\texttt{E}_1,\infty )$ as well. Therefore, we have $c\cdot \vec {\omega }_{\infty }(\texttt{E}) =0$ on $[\texttt{E}_1,\infty )$. By Lemma 5.3 and dividing by $\texttt{E}$, we have, for any $\texttt{E}\in [\texttt{E}_1,\infty )$,

5.10 $$\begin{aligned} c_1 \cos ( \pi \vartheta _1(\texttt{E}) ) + ... + c_{\kappa _0} \cos (\pi \vartheta _{\kappa _0}(\texttt{E})) = 0 \quad \forall \,\texttt{E}\in [\texttt{E}_1,\infty ), \end{aligned}$$

where

5.11 $$\begin{aligned} \begin{aligned}&\vartheta _j(\texttt{E}) := \alpha _0(j) + \alpha _2(j) \beta _j(\texttt{E})^2 + o(\beta _j(\texttt{E})^3), \quad \texttt{E}\rightarrow +\infty ,\\&\beta _j(\texttt{E}) := \exp \big ( ((\kappa _0+\tfrac{1}{4})\pi - \texttt{E}) \cos (\pi \alpha _0(j)) \big ) . \end{aligned} \end{aligned}$$

In particular, we further expand the asymptotic in (5.3), obtaining, for any $j=1,...,\kappa _0$, in the limit regime $\texttt{E}\rightarrow +\infty$,

5.12 $$\begin{aligned} \cos (\pi \vartheta _j(\texttt{E})) = \cos (\pi \alpha _0(j)) - \pi \alpha _2(j) \sin (\pi \alpha _0(j)) \beta _j(\texttt{E})^2 + o(\beta _j(\texttt{E})^3). \nonumber \\ \end{aligned}$$

By (5.11), we have

$$\begin{aligned} \partial _\texttt{E}\beta _j(\texttt{E})^2 = -2\cos (\pi \alpha _0(j)) \beta _j(\texttt{E})^2 , \quad \partial _\texttt{E}\big ( o(\beta _j(\texttt{E})^3)\big ) = o(\beta _j(\texttt{E})^3) . \end{aligned}$$

By differentiating with respect to $\texttt{E}$ in (5.10), using (5.12), we get, in the asymptotic regime $\texttt{E}\rightarrow + \infty$,

5.13 $$\begin{aligned}&\, c_1 \big ( \alpha _2(1) \sin (2\pi \alpha _0(1)) \beta _1(\texttt{E}) ^2 + o(\beta _1(\texttt{E})^3) \big ) \nonumber \\&\quad + \,... \, \nonumber \\&\quad + \, c_{\kappa _0-1} \big ( \alpha _2(\kappa _0-1) \sin (2\pi \alpha _0(\kappa _0-1)) \beta _{\kappa _0-1}(\texttt{E}) ^2 + o(\beta _{\kappa _0-1}(\texttt{E})^3) \big ) \nonumber \\&\quad + \, c_{\kappa _0} \big ( \alpha _2(\kappa _0) \sin (2\pi \alpha _0(\kappa _0)) \beta _{\kappa _0}(\texttt{E}) ^2 + o(\beta _{\kappa _0}(\texttt{E})^3) \big ) = 0. \end{aligned}$$

The solution $\alpha _{0}(j)$ of (5.4) are monotone increasing with respect to *j* because we have $\alpha _{0}'(j)=\big (1+(\kappa _{0}+\frac{1}{4})\pi \cos (\pi \alpha _{0}(j))\big )^{-1}>0$, since $\cos (\pi \alpha _{0}(j))\in (0,1)$ (the derivative $\alpha _{0}'(j)$ is of course meant with *j* as a continuous variable). Therefore, we have $0<\alpha _0(1)< \alpha _0(2)< ...< \alpha _0(\kappa _0-1)< \alpha _0(\kappa _0) < \tfrac{1}{2}$.

It follows that

$$\begin{aligned} 0< \exp (-\texttt{E}\cos (\pi \alpha _0(1)))< ...< \exp (-\texttt{E}\cos (\pi \alpha _0(\kappa _0))) < 1 . \end{aligned}$$

This implies that, for any $\texttt{E}>(\kappa _{0}+\frac{1}{4})\pi$ large enough, recalling (5.11),

$$\begin{aligned} 0< \beta _1(\texttt{E})< \beta _2(\texttt{E}) ....< \beta _{\kappa _0-1}(\texttt{E})< \beta _{\kappa _0}(\texttt{E})<1 \end{aligned}$$

It means that $\beta _{\kappa _0}(\texttt{E})^2$ is the leading term in (5.13). Therefore, we multiply all the terms in (5.13) by $\beta _{\kappa _0}(\texttt{E})^2$ and, taking the limit $\texttt{E}\rightarrow +\infty$, we obtain

$$\begin{aligned} c_{\kappa _0} \alpha _2(\kappa _0)\sin (\pi \alpha _0(\kappa _0)) = 0. \end{aligned}$$

Since $\alpha _2(\kappa _0)\sin (\pi \alpha _0(\kappa _0))\ne 0$ by Lemma 5.3, we obtain $c_{\kappa _0}=0$. We insert this constrain in (5.13) and we iterate the procedure with a new leading term at each step. We conclude that we must have $c_{\kappa _0} = c_{\kappa _0-1}=...=c_1=0$, which is a contradiction. The claim is proved. $\square$

Roughly speaking, the non-degeneracy is an open condition. Therefore, the property extends from the limit vector $\vec {\omega }_{\infty }(\texttt{E})$ to $\vec {\omega }_{{\mathfrak {m}}}(\texttt{E})$ when ${\mathfrak {m}}$ is sufficiently large.

### Theorem 5.5

(Non-degeneracy for $\vec {\omega }_{{\mathfrak {m}}}(\texttt{E})$*)* There exists ${\overline{{\mathfrak {m}}}} \equiv {\overline{{\mathfrak {m}}}}(\kappa _0) \gg 1$ such that, for any ${\mathfrak {m}}\geqslant {\overline{{\mathfrak {m}}}}$, the vector $\vec {\omega }_{{\mathfrak {m}}}(\texttt{E})$ in (5.1) is non-degenerate in the interval $[\texttt{E}_1,\texttt{E}_2]$, assuming $\texttt{E}_1>1$ sufficiently large.

### Proof

By contradiction, assume that for any ${\overline{{\mathfrak {m}}}}\gg 1$ there exists ${\mathfrak {m}}> {\overline{{\mathfrak {m}}}}$ and a vector $c_{{\mathfrak {m}}} \in {{\mathbb {R}}}^{\kappa _0}\setminus \{0\}$ such that $\vec {\omega }_{{\mathfrak {m}}}(\texttt{E}) \cdot c_{{\mathfrak {m}}}= 0$ for any $\texttt{E}\in [\texttt{E}_1, \texttt{E}_2]$. Clearly by defining $d_{\mathfrak {m}}:= \frac{c_{\mathfrak {m}}}{|c_{\mathfrak {m}}|}$ one also has that

$$\begin{aligned} \vec {\omega }_{{\mathfrak {m}}}(\texttt{E}) \cdot d_{\mathfrak {m}}= 0 \quad \forall \,\texttt{E}\in [\texttt{E}_1, \texttt{E}_2]. \end{aligned}$$

Since $|d_{\mathfrak {m}}| = 1$ for any ${\mathfrak {m}}>{\overline{{\mathfrak {m}}}}$, up to subsequences $d_{\mathfrak {m}}\rightarrow {\overline{d}}$, with $|\,{\overline{d}}\,| = 1$. Moreover $\sup _{\texttt{E}\in [\texttt{E}_1, \texttt{E}_2]} |\vec {\omega }_{{\mathfrak {m}}}(\texttt{E}) - \vec {\omega }_{\infty }(\texttt{E})| \rightarrow 0$ as ${\mathfrak {m}}\rightarrow \infty$ by Proposition 3.10. Hence, up to subsequences $\vec {\omega }_{{\mathfrak {m}}}(\texttt{E}) \cdot d_{\mathfrak {m}}$ converges to $\vec {\omega }_{\infty } (\texttt{E}) \cdot {\overline{d}}$ uniformly on $[\texttt{E}_1, \texttt{E}_2]$ as ${\mathfrak {m}}\rightarrow \infty$. This clearly implies that

$$\begin{aligned} \vec {\omega }_{\infty }(\texttt{E}) \cdot {\overline{d}}= 0, \quad \forall \,\texttt{E}\,\in [\texttt{E}_1, \texttt{E}_2], \end{aligned}$$

which contradicts the non degeneracy of the vector $\vec {\omega }_{\infty }$ proved in Proposition 5.4. $\square$

The next proposition is the key of the argument. It provides a quantitative bound from the qualitative non-degeneracy condition in Theorem 5.5.

### Proposition 5.6

(Transversality) Let ${\overline{{\mathfrak {m}}}}\gg 1$ as in Theorem 5.5. Then, for any ${\mathfrak {m}}\geqslant {\overline{{\mathfrak {m}}}}$, there exist $m_0\in {{\mathbb {N}}}$ and $\rho _0>0$ such that, for any $\texttt{E}\in [\texttt{E}_1,\texttt{E}_2]$,

5.14 $$\begin{aligned} \max _{0\leqslant n \leqslant m_0} | \partial _\texttt {E}^n \vec {\omega }_{{\mathfrak {m}}}(\texttt {E})\cdot \ell | \geqslant \rho _0\mathinner {\langle {\ell }\rangle } , \quad \forall \,\ell \in {{\mathbb {Z}}}^{\kappa _0}\setminus \{0\} \,. \end{aligned}$$

We call $\rho _0$ the amount of non-degeneracy and $m_0$ the index of non-degeneracy.

### Proof

Let ${\mathfrak {m}}\geqslant {\overline{{\mathfrak {m}}}}$. By contradiction, assume that for any $m\in {{\mathbb {N}}}$ there exist $\texttt{E}_m\in [\texttt{E}_1,\texttt{E}_2]$ and $\ell _m\in {{\mathbb {Z}}}^{\kappa _0}\setminus \{0\}$ such that

5.15 $$\begin{aligned} \Big | \partial _\texttt{E}^n \vec {\omega }_{{\mathfrak {m}}}(\texttt{E}_m) \cdot \frac{\ell _m}{\mathinner {\langle {\ell _m}\rangle }} \Big | < \frac{1}{\mathinner {\langle {m}\rangle }} , \quad \forall \,0\leqslant n\leqslant m \, . \end{aligned}$$

The sequences $(\texttt{E}_m)_{m\in {{\mathbb {N}}}}\subset [\texttt{E}_1,\texttt{E}_2]$ and $(\ell _m/\mathinner {\langle {\ell _m}\rangle })_{m\in {{\mathbb {N}}}}\subset {{\mathbb {R}}}^{\kappa _0}\setminus \{0\}$ are both bounded. By compactness, up to subsequences $\texttt{E}_m\rightarrow {\overline{\texttt{E}}}\in [\texttt{E}_1,\texttt{E}_2]$ and $\ell _m/\mathinner {\langle {\ell _m}\rangle }\rightarrow {\overline{c}}\ne 0$. Therefore, in the limit for $m\rightarrow + \infty$, by (5.15) we get $\partial _\texttt{E}^n \vec {\omega }_{{\mathfrak {m}}}({\overline{\texttt{E}}})\cdot {\overline{c}} = 0$ for any $n\in {{\mathbb {N}}}_0$. By the analyticity of $\vec {\omega }_{{\mathfrak {m}}}(\texttt{E})$ (see Remark 5.2), we deduce that the function $\texttt{E}\mapsto \vec {\omega }_{{\mathfrak {m}}}(\texttt{E})\cdot {\overline{c}}$ is identically zero on $[\texttt{E}_1,\texttt{E}_2]$, which contradicts Proposition 5.4. $\square$

Thanks to Proposition 5.6, we can finally prove that the Diophantine non-resonant condition for $\vec {\omega }_{{\mathfrak {m}}}(\texttt{E})$ holds on a large set of parameters.

### Proposition 5.7

Let $(\kappa _{0}+\tfrac{1}{4})\pi<\texttt{E}_1<\texttt{E}_2<\infty$ be given. Let also ${\overline{\tau }}\geqslant m_0\kappa _{0}$ and ${\overline{\upsilon }}\in (0,1)$. Then the set

5.16 $$\begin{aligned} {\overline{\mathcal {K}}} = {\overline{\mathcal {K}}}({\overline{\upsilon }},{\overline{\tau }}) := \big \{ \texttt{E}\in [\texttt{E}_1,\texttt{E}_2] \,: \, |\vec {\omega }_{{\texttt{m}}}({\texttt{E}}) \cdot \ell | \geqslant {\overline{\upsilon }} \mathinner {\langle {\ell }\rangle }^{-{\overline{\tau }}}, \ \ \forall \,\ell \in {{\mathbb {Z}}}^{\kappa _0} \setminus \{ 0 \}\ \big \}, \nonumber \\ \end{aligned}$$

is of large measure with respect to ${\overline{\upsilon }}$, namely $|[\texttt{E}_1,\texttt{E}_2] \setminus {\overline{\mathcal {K}}}| = o({\overline{\upsilon }}^{1/m_0})$.

### Proof

We write

5.17 $$\begin{aligned} {\overline{\mathcal {K}}}^c := [\texttt{E}_1,\texttt{E}_2] \setminus {\overline{\mathcal {K}}} = \bigcup _{\ell \ne 0} {\overline{R}}_{\ell } = \bigcup _{\ell \ne 0} \big \{ \texttt{E}\in [\texttt{E}_1,\texttt{E}_2] \,:\, |\vec {\omega }_{{\mathfrak {m}}}(\texttt{E})\cdot \ell | < {\overline{\upsilon }} \mathinner {\langle {\ell }\rangle }^{-{\overline{\tau }}} \big \}. \nonumber \\ \end{aligned}$$

We claim that $|{\overline{R}}_{\ell }| \lesssim ({\overline{\upsilon }}\mathinner {\langle {\ell }\rangle }^{-({\overline{\tau }}+1)})^{\frac{1}{m_0}}$. We write

$$\begin{aligned} {\overline{R}}_{\ell }= \Big \{ \texttt{E}\in [\texttt{E}_1,\texttt{E}_2] \, : \, |{\overline{f}}_{\ell }({{\texttt{E}}})| < {\overline{\upsilon }} \mathinner {\langle {\ell }\rangle }^{-({\overline{\tau }}+1)} \Big \}, \end{aligned}$$

where ${\overline{f}}_{\ell }({{\texttt{E}}}):=\vec {\omega }_{{\mathfrak {m}}}({{\texttt{E}}})\cdot \frac{\ell }{\mathinner {\langle {\ell }\rangle }}$. By Proposition 5.6, we have $\max _{0\leqslant n \leqslant k_0}|\partial _{{\texttt{E}}}^n {\overline{f}}_{\ell }({{\texttt{E}}})|\geqslant \rho _0$ for any ${{\texttt{E}}}\in [\texttt{E}_1.\texttt{E}_2]$. In addition, by Remark 5.2, we have $\max _{0\leqslant n \leqslant m_0}|\partial _{{\texttt{E}}}^n {\overline{f}}_{\ell }({{\texttt{E}}})|\leqslant C$ for any ${{\texttt{E}}}\in [\texttt{E}_1.\texttt{E}_2]$ for some constant $C=C(\texttt{E}_1,\texttt{E}_2,m_0)>0$. In particular, ${\overline{f}}_{\ell }$ is of class $\mathcal {C}^{k_0-1}=\mathcal {C}^{m_0+1}$. Thus, Theorem 17.1 in [44] applies, whence the claim follows. Finally, we estimate (5.17) by

$$\begin{aligned} |{\overline{\mathcal {K}}}^c| \leqslant \sum _{\ell \ne 0} |{\overline{R}}_{\ell }| \lesssim {\overline{\upsilon }}^{\frac{1}{m_0}} \sum _{\ell \ne 0} \mathinner {\langle {\ell }\rangle }^{-\frac{{\overline{\tau }}+1}{m_0}} \lesssim {\overline{\upsilon }}^{\frac{1}{m_0}} \end{aligned}$$

since ${\overline{\tau }} > m_0 \kappa _{0} -1$. This concludes the proof. $\square$

## Proof of Theorem 1.1 and Measure Estimates

Assuming that Theorem 4.5 holds, we deduce now Theorem 1.1. By (4.52), for any ${\texttt{A}} \in \mathcal {J}_\varepsilon ({\texttt{E}})$, the ${\texttt{A}}$-dependent family of functions $\alpha _{\infty }(\cdot , {\texttt{A}})$ from ${\mathtt {\Omega }}$ into their images $\alpha _{\infty }({\mathtt {\Omega }}\times \{ {\texttt{A}}\})$ are invertible and

6.1 $$\begin{aligned} \begin{aligned}&\beta = \alpha _{\infty }(\omega , {\texttt{A}}) = \omega +r_{\varepsilon }(\omega , {\texttt{A}}), \\&\left| r_{\varepsilon } \right| ^{k_0,\upsilon } \lesssim \sqrt{\varepsilon } \upsilon ^{-1}, \end{aligned} \Leftrightarrow \quad \begin{aligned}&\omega = \alpha _{\infty }^{-1}(\beta , {\texttt{A}}) = \beta +\breve{r}_{\varepsilon }(\beta , {\texttt{A}}), \\&\left| \breve{r}_{\varepsilon } \right| ^{k_0,\upsilon } \lesssim \sqrt{\varepsilon } \upsilon ^{-1}. \end{aligned} \end{aligned}$$

Then, for any $\beta \in \alpha _{\infty }(\texttt{G}^\upsilon \times \mathcal {J}_\varepsilon ({\texttt{E}}))$, Theorem 4.5 proves the existence of an embedded invariant torus filled by quasi-periodic solutions with Diophantine frequency $\omega =\alpha _{\infty }^{-1}(\beta , {\texttt{A}})$ for the Hamiltonian

$$\begin{aligned} \mathcal {H}_{\varepsilon , \beta } = \beta \cdot I+ \tfrac{1}{2}\big (z, ( {\begin{matrix} -\mathcal {L}_{{\mathfrak {m}}}(\texttt{E}) &  0 \\ 0 &  \textrm{Id} \end{matrix}} ) z \big )_{L^2} + \sqrt{\varepsilon } \, \mathcal {P}_\varepsilon . \end{aligned}$$

Consider the curve of the unperturbed tangential frequency vector ${\texttt{A}} \rightarrow \vec {\omega }_{{\mathfrak {m}}}({\texttt{A}})$ in (5.1). In Theorem 6.1 below we prove that, for a density 1 set of parameters ${\texttt{A}} \in \mathcal {J}_\varepsilon ({\texttt{E}})$, the vector $\alpha _{\infty }^{-1}(\vec {\omega }_{{\mathfrak {m}}}({\texttt{A}}), {\texttt{A}})$ is in $\texttt{G}^\upsilon$, obtaining an embedded torus for the Hamiltonian $\mathcal {H}_{\varepsilon ,\beta }$ with $\beta =\alpha _{\infty }(\omega ,\texttt{A})=\vec {\omega }_{{\mathfrak {m}}}(\texttt{A})$, and therefore for the Hamiltonian $\mathcal {H}_{\varepsilon }$ in (4.38), filled by quasi-periodic solutions with Diophantine frequency vector $\omega = \alpha _{\infty }^{-1}(\vec {\omega }_{{\mathfrak {m}}}({\texttt{A}}), {\texttt{A}})$, denoted ${\widetilde{\omega }}$ in Theorem 1.1. Clearly, by the estimates (4.39), (6.1), one has that the vector ${\tilde{\omega }}$ satisfies

$$\begin{aligned} {\widetilde{\omega }} = \vec {\omega }_{{\texttt{m}}}({\texttt{A}}) + O(\sqrt{\varepsilon } \upsilon ^{- 1}) = \vec {\omega }_{{\texttt{m}}}({\texttt{E}}) + O(\sqrt{\varepsilon } + \sqrt{\varepsilon } \upsilon ^{- 1} ) = \vec {\omega }_{{\texttt{m}}}({\texttt{E}}) + O( \sqrt{\varepsilon } \upsilon ^{- 1} ) . \end{aligned}$$

Thus, the function $A(i_{\infty }({\widetilde{\omega }}x)) = (\zeta _1({\widetilde{\omega }}x, y), \zeta _2({\widetilde{\omega }}x, y))$, where *A* is defined in (4.33), is a quasi-periodic solution of the Equation (4.27) and hence, by recalling Lemma 4.3, (4.22), (4.23), (4.26), $h_\eta (y) + \varepsilon \zeta _1(\omega x, y)$ is a quasi-periodic solution of (4.11). This proves Theorem 1.1, together with the following measure estimate:

### Theorem 6.1

(Measure estimate) Let

6.2 $$\begin{aligned} \begin{aligned}&\upsilon = \varepsilon ^{\text {a}} , \quad 0<\text {a}<\min \big \{ \text {a}_0, \tfrac{\texttt {d}(\tau )-1}{2(\texttt {d}(\tau )-\frac{1}{m_0})} \big \}, \quad \texttt {d}(\tau ):=\tfrac{\tau +1 -m_0\kappa _{0}}{m_0({\overline{\tau }} +1)}, \\  &\tau > \text {max}\{m_0 \kappa _0-1,\, m_0 (\kappa _0 + {\overline{\tau }} +1)-1 \} , \end{aligned} \end{aligned}$$

where $m_0$ is the index of non-degeneracy given in Proposition 5.6, $k_0:= m_0+2$, ${\overline{\tau }} \geqslant m_0\kappa _{0}$ is fixed and $\texttt{a}_0\in (0,1)$ is defined in (8.3) in Theorem 8.2 . Then, fixed $\texttt{E}\in {\overline{\mathcal {K}}}$, with ${\overline{\mathcal {K}}}$ as in (1.19) (see also (5.16) below), for $\varepsilon \in (0, \varepsilon _0)$ small enough, the set

6.3 $$\begin{aligned} \mathcal {K}_{\varepsilon }=\mathcal {K}_{\varepsilon }(\texttt{E}):= \big \{ {\texttt{A}} \in \mathcal {J}_\varepsilon ({\texttt{E}}) = [{\texttt{E}} - \sqrt{\varepsilon }, {\texttt{E}} + \sqrt{\varepsilon }] \, : \, \alpha _{\infty }^{-1}( \vec {\omega }_{{\mathfrak {m}}}({{\texttt{A}}}), {\texttt{A}}) \in \texttt{G}^\upsilon \big \} \nonumber \\ \end{aligned}$$

has density 1 as $\varepsilon \rightarrow 0$, namely

$$\begin{aligned} \frac{|\mathcal {K}_{\varepsilon }(\texttt{E})|}{|\mathcal {J}_{\varepsilon }(\texttt{E})|}=\frac{| \mathcal {K}_{\varepsilon }(\texttt{E})|}{2 \sqrt{\varepsilon }} \rightarrow 1\quad \text {as} \quad \varepsilon \rightarrow 0, \quad \text {uniformly in } \, \texttt{E}\in {\overline{\mathcal {K}}}. \end{aligned}$$

The rest of this section is devoted to prove Theorem 6.1. The key point to compute the density of the set $\mathcal {K}_\varepsilon (\texttt{E})$ is that the unperturbed frequency $\vec {\omega }_{{\texttt{m}}}({{\texttt{E}}})$ is Diophantine with constants ${\overline{\upsilon }} \in (0, 1)$ and ${\overline{\tau }}$ stronger than the ones in (4.54), namely with $\upsilon \ll {\overline{\upsilon }}$ and $\tau \gg {\overline{\tau }}$, see Proposition 5.7.

By (6.1) we have that, for ${\texttt{A}} \in \mathcal {J}_\varepsilon ({\texttt{E}})$,

6.4 $$\begin{aligned} \vec { \omega }_{\varepsilon } ({{\texttt{A}}}):= \alpha _{\infty }^{-1}(\vec {\omega }_{{\mathfrak {m}}}({\texttt{A}}), {\texttt{A}}) = \vec {\omega }_{{\mathfrak {m}}}({\texttt{A}}) +\vec {r}_{\varepsilon }({\texttt{A}}) , \end{aligned}$$

where $$\vec {r}_\varepsilon ({\texttt{A}}) := \breve{r}_\varepsilon (\vec {\omega }_{{\mathfrak {m}}}({\texttt{A}}), {\texttt{A}}) $$ satisfies

6.5 $$\begin{aligned} |\partial _{{\texttt{A}}}^k {\vec {r}}_{\varepsilon } ({{\texttt{A}}})| \leqslant C \sqrt{\varepsilon }\upsilon ^{-(1+k)} , \quad \forall \,0 \leqslant k \leqslant k_0 , \ \ \text {uniformly on } \ \mathcal {J}_\varepsilon ({\texttt{E}}). \end{aligned}$$

By (4.54), the Cantor set $\mathcal {K}_{\varepsilon }(\texttt{E})$ in (6.3) becomes

$$\begin{aligned} \mathcal {K}_{\varepsilon }(\texttt{E}):= \Big \{ {\texttt{A}} \in \mathcal {J}_\varepsilon ({\texttt{E}}) \,:\, |\vec {\omega }_{\varepsilon }({{\texttt{A}}}) \cdot \ell | \geqslant \upsilon \mathinner {\langle {\ell }\rangle }^{-\tau } , \ \forall \,\ell \in {{\mathbb {Z}}}^{\kappa _0}\setminus \{0\} \Big \}. \end{aligned}$$

We estimate the measure of the complementary set

6.6 $$\begin{aligned} \mathcal {K}_{\varepsilon }^c(\texttt {E}):= &   \mathcal {J}_\varepsilon ({\texttt {E}})\setminus \mathcal {K}_{\varepsilon }(\texttt {E}) := \bigcup _{\ell \ne 0}R_{\ell }({\texttt {E}})\\ \nonumber:= &   \bigcup _{\ell \ne 0} \Big \{ {{\texttt {A}}} \in \!\mathcal {J}_\varepsilon ({\texttt {E}}) : |\vec {\omega }_{\varepsilon } ({{\texttt {A}}})\cdot \ell |\!<\!\upsilon \mathinner {\langle {\ell }\rangle }^{-\tau } \! \Big \}. \end{aligned}$$

To estimate the measure of the sets $R_{\ell }({\texttt{E}})$ in (6.6), the key point is to show that the perturbed linear frequencies satisfy the similar lower bound in (5.14) in Proposition 5.6. The transversality property actually holds not only in the vicinity of the fixed ${\texttt{E}}$, but also on the full parameter set $[{\texttt{E}}_1, {\texttt{E}}_2]$.

### Lemma 6.2

(Perturbed transversality) For $\varepsilon \in (0,\varepsilon _0)$ small enough and for all ${{\texttt{A}}}\in [\texttt{E}_1,\texttt{E}_2]$,

6.7 $$\begin{aligned} \max _{0\leqslant n \leqslant m_0} | \partial _{{\texttt{A}}}^n \vec {\omega }_{\varepsilon }({{\texttt{A}}})\cdot \ell | \geqslant \frac{\rho _0}{2}\mathinner {\langle {\ell }\rangle } , \quad \forall \,\ell \in {{\mathbb {Z}}}^{\kappa _0}\setminus \{0\} \,; \end{aligned}$$

here $\rho _0$ is the amount of non-degeneracy that has been defined in Proposition 5.6. In particular, the same estimate (6.7) holds for any $\texttt{A}\in \mathcal {J}_{\varepsilon }(\texttt{E})$, with the constant $\rho _0$ independent of $\varepsilon >0$.

### Proof

The estimate (6.7) follows directly from (6.4)–(6.5) provided $\sqrt{\varepsilon }\upsilon ^{-(1+m_0)}\leqslant \rho _0/(2C)$, which, by (4.50) and (6.2), is satisfied for $\varepsilon$ sufficiently small. $\square$

As an application of Rüssmann Theorem 17.1 in [44], we deduce

### Lemma 6.3

(Estimates of the resonant sets) The measure of the sets $R_{\ell }({\texttt{E}})$ in (6.6) satisfies $|R_{\ell }({\texttt{E}})| \lesssim (\upsilon \mathinner {\langle {\ell }\rangle }^{-(\tau +1)})^{\frac{1}{m_0}}$ for any $\ell \ne 0$.

### Proof

We write

$$\begin{aligned} R_{\ell }({{\texttt{E}}}) = \Big \{ {{\texttt{A}}} \in \mathcal {J}_{\varepsilon }(\texttt{E})\, : \, |f_{\ell }({{\texttt{A}}})| < \upsilon \mathinner {\langle {\ell }\rangle }^{-(\tau +1)} \Big \}, \end{aligned}$$

where $f_{\ell }({{\texttt{A}}}):=\vec {\omega }_{\varepsilon }({{\texttt{A}}})\cdot \frac{\ell }{\mathinner {\langle {\ell }\rangle }}$. By (6.7), we have $\max _{0\leqslant n \leqslant k_0}|\partial _{{\texttt{A}}}^n f_{\ell }({{\texttt{A}}})|\geqslant \rho _0/2$ for any ${{\texttt{A}}}\in [\texttt{E}_1.\texttt{E}_2]$. In addition, (6.4)–(6.5) imply that $\max _{0\leqslant n \leqslant m_0}|\partial _{{\texttt{A}}}^n f_{\ell }({{\texttt{A}}})|\leqslant C$ for any ${{\texttt{A}}}\in [\texttt{E}_1.\texttt{E}_2]$, provided $\sqrt{\varepsilon }\upsilon ^{-(1+m_0)}$ is small enough, namely, by (6.2), for $\varepsilon$ small enough. In particular, $f_{\ell }$ is of class $\mathcal {C}^{k_0-1}=\mathcal {C}^{m_0+1}$. Thus, Theorem 17.1 in [44] applies, whence the lemma follows. $\square$

### Lemma 6.4

There exists $C = C({\overline{\upsilon }}) > 0$ such that, if $0 < |\ell | \leqslant C (\upsilon \varepsilon ^{-\frac{1}{2}})^{ \frac{1}{({\overline{\tau }} + 1)}}$, then $R_\ell (\texttt{E}) = \varnothing$.

### Proof

By the estimates (4.39), (6.1), we deduce $|\omega _\varepsilon ({{\texttt{A}}}) - \vec {\omega }_{{\texttt{m}}}({{\texttt{E}}})| \lesssim \sqrt{\varepsilon } \upsilon ^{- 1}$ for any ${\texttt{A}} \in \mathcal {J}_{\varepsilon }(\texttt{E})$. Hence, by Proposition 5.7, we get, for some constant $C \geqslant 0$,

$$\begin{aligned} \begin{aligned} |\omega _\varepsilon ({{\texttt{A}}}) \cdot \ell |&\geqslant |\vec {\omega }_{{\texttt{m}}}({\texttt{E}}) \cdot \ell | - C \sqrt{\varepsilon } \upsilon ^{- 1} |\ell | \geqslant \frac{{\overline{\upsilon }}}{| \ell |^{{\overline{\tau }}}} - C \sqrt{\varepsilon } \upsilon ^{- 1} |\ell | \geqslant \frac{{\overline{\upsilon }}}{2 | \ell |^{{\overline{\tau }}}} \end{aligned} \end{aligned}$$

provided that the condition stated in the statement holds. Hence, for $\upsilon \ll {\overline{\upsilon }}$ and $\tau \gg {\overline{\tau }}$, this implies that $R_\ell (\texttt{E}) = \varnothing$. $\square$

### Proof of Theorem 6.1 completed

The series $\sum _{\ell \ne 0} |\ell |^{-\frac{\tau + 1}{m_0}}$ is convergent because $\frac{\tau +1}{m_0}>\kappa _0$ by (6.2). Hence, by Lemmata 6.3, 6.4, the measure of the set $\mathcal {K}_{\varepsilon }^c(\texttt{E})$ in (6.6) is estimated by

$$\begin{aligned} \begin{aligned} |\mathcal {K}_{\varepsilon }^c(\texttt{E})|&\leqslant \sum _{|\ell |> C (\upsilon \varepsilon ^{-\frac{1}{2}})^{\frac{1}{{\overline{\tau }} + 1}} } |R_{\ell }(\texttt{E})| \lesssim \upsilon ^{\frac{1}{m_0}}\sum _{|\ell | > C (\upsilon \varepsilon ^{-\frac{1}{2}})^{\frac{1}{{\overline{\tau }} + 1}} } \frac{1}{|\ell |^{\frac{\tau + 1}{m_0}}} \\&\lesssim \upsilon ^{\frac{1}{m_0}} \Big ( \dfrac{\sqrt{\varepsilon }}{\upsilon } \Big )^{\frac{\tau + 1 - m_0 \kappa _0 }{m_0({\overline{\tau }} + 1)}} \simeq \upsilon ^{\frac{1}{m_0}} \Big ( \dfrac{\sqrt{\varepsilon }}{\upsilon } \Big )^{\alpha (\tau - \beta )} , \end{aligned} \end{aligned}$$

where $\alpha := (m_0({\overline{\tau }}+1))^{-1}$ and $\beta := m_0 \kappa _{0}-1$. Therefore, we have

$$\begin{aligned} \frac{|\mathcal {K}_\varepsilon ^2(\texttt{E})|}{2 \sqrt{\varepsilon }} \lesssim {\varepsilon }^{\texttt{p}_1} \upsilon ^{- \texttt{p}_2}, \quad \texttt{p}_1 := \frac{\alpha ( \tau - \beta )- 1}{2} , \quad \texttt{p}_2 :=\frac{m_0\alpha (\tau -\beta ) -1}{m_0} . \end{aligned}$$

Since $\tau > m_0 (\kappa _{0}+{\overline{\tau }}+1)$ by (6.2), we have that $\texttt{p}_1>0$ and, consequently, also $\texttt{p}_2>0$. Then, for $\upsilon = \varepsilon ^{\texttt{a}}$ with

$$\begin{aligned} 0< \texttt{a}< \frac{\texttt{p}_1}{\texttt{p}_2}=\frac{\alpha (\tau -\beta )-1}{2(\alpha (\tau -\beta )-\tfrac{1}{m_0})}<\frac{1}{2} <1, \end{aligned}$$

we have $(2\sqrt{\varepsilon })^{-1}| \mathcal {K}_{\varepsilon }^c(\texttt{E})|\lesssim \varepsilon ^{\texttt{p}_1-\texttt{a}\texttt{p}_2}\rightarrow 0$ as $\varepsilon \rightarrow 0$. It implies $(2\sqrt{\varepsilon })^{-1}|\mathcal {K}_{\varepsilon }(\texttt{E})|\geqslant 1- C\varepsilon ^{\frac{\textrm{a}}{m_0}}$ and the proof of Theorem 6.1 is concluded. $\square$

## Approximate Inverse

In order to implement a convergent Nash–Moser scheme that leads to a solution of $\mathcal {F}(i,\alpha )=0$, where $\mathcal {F}(i, \alpha )$ is the nonlinear operator defined in (4.43), we construct the *approximate right inverses* of the linearized operators

$$\begin{aligned} \textrm{d}_{i,\alpha }\mathcal {F}(i_{0},\alpha _{0})[{{\widehat{i}}},{\widehat{\alpha }}] = \omega \cdot \partial _\textbf{x}{{\widehat{i}}}- \textrm{d}_i X_{\mathcal {H}_{\varepsilon , \alpha }}\left( i_{0}(\textbf{x}) \right) [{{\widehat{i}}}] - \left( {\widehat{\alpha }},0,0\right) . \end{aligned}$$

Note that $\textrm{d}_{i,\alpha }\mathcal {F}(i_{0},\alpha _{0})=\textrm{d}_{i,\alpha }\mathcal {F}(i_{0})$ is independent of $\alpha _{0}$. We assume that the torus $i_{0} (\textbf{x}) = ( \theta _{0} (\textbf{x}), I_{0} (\textbf{x}), z_{0} (\textbf{x}))$ is reversible, according to (4.45).

In the sequel we shall assume the smallness condition,

$$\begin{aligned} \sqrt{\varepsilon }\upsilon ^{-1} \ll 1 \, . \end{aligned}$$

First of all, we state tame estimates for the composition operator induced by the Hamiltonian vector field $X_{\mathcal {P}_\varepsilon }= ( \partial _I \mathcal {P}_\varepsilon , - \partial _\theta \mathcal {P}_\varepsilon , J \nabla _{z} \mathcal {P}_\varepsilon )$ in (4.43).

### Lemma 7.1

(Estimates of the perturbation $\mathcal {P}_\varepsilon$) Let $S \geqslant 2(s_0+\mu )$, with $\mu >0$ as in Lemma 4.2-(*ii*). Let ${\mathfrak {I}}(\textbf{x})$ in (4.41) satisfy $\left\| {\mathfrak {I}} \right\| _{\mathcal {X}^{s_0}}^{k_0,\upsilon }\leqslant 1$. Then, for any $s_0 \leqslant s \leqslant S/2 - \mu$ and any $\varepsilon \ll 1$, we have $\left\| X_{\mathcal {P}_\varepsilon }(i) \right\| _{\mathcal {Y}^{s}}^{k_0,\upsilon } \lesssim _s 1 + \left\| {\mathfrak {I}} \right\| _{\mathcal {X}^{s}}^{k_0,\upsilon }$, and, for all ${{\widehat{i}}}:= ({\widehat{\theta }},{{\widehat{I}}},{{\widehat{z}}})$, ${{\widehat{i}}}_1:= ({\widehat{\theta }}_1,{{\widehat{I}}}_1,{{\widehat{z}}}_1)$, ${{\widehat{i}}}_2:= ({\widehat{\theta }}_2,{{\widehat{I}}}_2,{{\widehat{z}}}_2)$

$$\begin{aligned} \left\| \textrm{d}_i X_{\mathcal {P}_\varepsilon }(i)[{{\widehat{i}}}] \right\| _{\mathcal {Y}^{s}}^{k_0,\upsilon }&\lesssim _s \left\| {{\widehat{i}}} \right\| _{\mathcal {Y}^s}^{k_0,\upsilon } + \left\| {\mathfrak {I}} \right\| _{\mathcal {X}^{s}}^{k_0,\upsilon }\left\| {{\widehat{i}}} \right\| _{\mathcal {Y}^{s_0}}^{k_0,\upsilon } , \\ \left\| \textrm{d}_i^2 X_{\mathcal {P}_\varepsilon }(i)[{{\widehat{i}}}_1,{{\widehat{i}}}_2] \right\| _{\mathcal {Y}^{s}}^{k_0,\upsilon }&\lesssim _s \left\| {{\widehat{i}}}_1 \right\| _{\mathcal {Y}^s}^{k_0,\upsilon }\left\| {{\widehat{i}}}_2 \right\| _{\mathcal {Y}^{s_0}}^{k_0,\upsilon } + \left\| {{\widehat{i}}}_1 \right\| _{\mathcal {Y}^{s_0}}^{k_0,\upsilon }\left\| {{\widehat{i}}}_2 \right\| _{\mathcal {Y}^s}^{k_0,\upsilon } + \left\| {\mathfrak {I}} \right\| _{\mathcal {X}^{s}}^{k_0,\upsilon } ( \left\| {{\widehat{i}}} \right\| _{\mathcal {Y}^{s_0}}^{k_0,\upsilon } )^2 . \end{aligned}$$

### Proof

From (4.38), (4.28) and (4.33), (4.36), the Hamiltonian vector field for $\mathcal {P}_\varepsilon =P_{\varepsilon }\circ A + \tfrac{1}{\sqrt{\varepsilon }}\big ( \vec {\omega }_{{\mathfrak {m}}}(\texttt{E})-\vec {\omega }_{{\mathfrak {m}}}(\texttt{A}) \big )\cdot I$ is given by

$$\begin{aligned} X_{\mathcal {P}_\varepsilon } = \begin{pmatrix} [\partial _I v^\intercal (\theta ,I)]^T \partial _{I} P_{\varepsilon }(A(\theta ,I,z)) + \tfrac{1}{\sqrt{\varepsilon }}\big ( \vec {\omega }_{{\mathfrak {m}}}(\texttt{E})-\vec {\omega }_{{\mathfrak {m}}}(\texttt{A}) \big )\\ -[\partial _\theta v^\intercal (\theta ,I)]^T \partial _{\theta } P_{\varepsilon }(A(\theta ,I,z)) \\ \Pi _\perp J^{-1}\nabla _{z} P_{\varepsilon }(A(\theta ,I,z)) \end{pmatrix}, \end{aligned}$$

where $\Pi ^\perp$ is the projection onto the subspace $\mathcal {X}_\perp$ in (4.30). Since $\nabla _{z} P_\varepsilon (\zeta _1, \zeta _2) = q_\varepsilon (y, \zeta _1)$, the claimed estimates follow by Lemma 4.4, by the definition of $v^\intercal$ and *A* in (4.33), by the interpolation inequality (2.4) (recall also the definitions given in (4.48)) and by the estimate in (4.39). $\square$

### Invertibility of the Linearized Operator

Along this section, we assume the following hypothesis, which is verified by the approximate solutions obtained at each step of the Nash–Moser Theorem 8.2. We recall the definitions of the spaces $\mathcal {X}^s_\bot , \mathcal {Y}^s_\bot , \mathcal {X}^s, \mathcal {Y}^s$ in (4.47)–(4.48) that we shall use in the whole section.

- ANSATZ. The map $\lambda \mapsto {\mathfrak {I}}_0(\lambda ) = i_0(\lambda ;\textbf{x})- (\textbf{x},0,0)$ is $k_0$-times differentiable with respect to the parameter $\lambda =(\omega ,\texttt{A})\in {{\mathbb {R}}}^{\kappa _0}\times \mathcal {J}_{\varepsilon }(\texttt{E})$ and, for some $\sigma :=\sigma (\kappa _0, k_0, \tau ) \gg 0$, $\upsilon \in (0,1)$,
  7.1 $$\begin{aligned} \left\| {\mathfrak {I}}_0 \right\| _{\mathcal {X}^{s_0+\sigma }}^{k_0,\upsilon } + \left| \alpha _0-\omega \right| ^{k_0,\upsilon } \leqslant C \sqrt{\varepsilon } \upsilon ^{-1} , \quad \sqrt{\varepsilon } \upsilon ^{- 1} \ll 1. \end{aligned}$$

We remark that, in the sequel, we denote by $\sigma \equiv \sigma (\kappa _0,k_0, \tau ) \gg 0$ constants, which may increase from lemma to lemma, that represent “loss of derivatives”.

As in [2, 7, 12], we first modify the approximate torus $i_0 (\textbf{x})$ to obtain a nearby isotropic torus $i_\delta (\textbf{x})$, namely such that the pull-back 1-form $i_\delta ^*\Lambda$ is closed, where $\Lambda$ is the Liouville 1-form defined in (4.35). We first consider the pull-back 1-form

$$\begin{aligned} i_0^*\Lambda = \sum _{k=1}^{\nu } a_k(\textbf{x}) \textrm{d}\textbf{x}_k \, , \ \ a_k(\textbf{x}) := -\big ( [ \partial _\textbf{x}\theta _0(\textbf{x}) ]^\top I_0(\textbf{x}) \big )_k +\tfrac{1}{2} \big ( J^{-1} z_0(\textbf{x}), \partial _{\textbf{x}_k} z_0(\textbf{x}) \big )_{L^2}, \end{aligned}$$

and its exterior differential

$$\begin{aligned} i_0^*\mathcal {W}= \textrm{d}i_0^*\Lambda = \sum _{1\leqslant k < j \leqslant \nu } A_{kj} \textrm{d}\textbf{x}_k \wedge \textrm{d}\textbf{x}_j , \quad A_{kj}(\textbf{x}) := \partial _{\textbf{x}_k} a_j(\textbf{x}) - \partial _{\textbf{x}_j}a_k(\textbf{x}) \, . \end{aligned}$$

By the formula given in Lemma 5.3 in [7], we deduce that for any $s \geqslant s_0$, if $\omega$ belongs to $\texttt{G}^\upsilon$ (see (4.54)), the estimate (assuming the ansatz (7.1)),

$$\begin{aligned} \begin{aligned}&\left\| A_{kj} \right\| _{s}^{k_0,\upsilon } \lesssim _s \upsilon ^{-1}\big ( \left\| Z \right\| _{\mathcal {Y}^{s+\sigma }}^{k_0, \upsilon } + \left\| {\mathfrak {I}}_0 \right\| _{\mathcal {X}^{s+ \sigma }}^{k_0,\upsilon } \left\| Z \right\| _{\mathcal {Y}^{s_0+ \sigma }}^{k_0,\upsilon } \big ) \, , \\&\Vert A_{k j}\Vert _{s}^{k_0, \upsilon } \lesssim _s \Vert {{\mathfrak {I}}}_0 \Vert _{\mathcal {X}^{s + 1}}^{k_0, \upsilon }. \end{aligned} \end{aligned}$$

for some $\sigma \equiv \sigma (k_0, \tau ) \gg 0$ large enough, where $Z(\textbf{x})$ is the “error function”

7.2 $$\begin{aligned} Z(\textbf{x}) := \mathcal {F}(i_0,\alpha _{0}) := \omega \cdot \partial _{\textbf{x}} i_0(\textbf{x}) - X_{H_{\alpha _0}}(i_0(\textbf{x})). \end{aligned}$$

Note that, if $Z (\textbf{x}) = 0$, the torus $i_0 (\textbf{x})$ is invariant for $X_{H_{\alpha _0}}$ and the 1-form $i_0^* \Lambda$ is closed, namely the torus $i_0 (\textbf{x})$ is isotropic. We denote below the Laplacian $\Delta _\textbf{x}:= \sum _{k=1}^{\nu }\partial _{\textbf{x}_k}^2$.

#### Lemma 7.2

(Isotropic torus) The torus $i_\delta (\textbf{x}):= ( \theta _0(\textbf{x}),I_\delta (\textbf{x}),w_0(\textbf{x}) )$, defined by

$$\begin{aligned} I_\delta (\textbf{x})\!:=\! I_0(\textbf{x}) + [ \partial _\textbf{x}\theta _0(\textbf{x}) ]^{-\top }\rho (\textbf{x}) , \quad \rho = (\rho _j)_{j=1, \ldots ,\kappa _0} \, , \quad \rho _j(\textbf{x})\! := \Delta _\textbf{x}^{-1} \sum _{k=1}^{\kappa _0}\partial _{\textbf{x}_k}A_{kj}(\textbf{x}), \end{aligned}$$

is isotropic. Moreover, there is $\sigma := \sigma (\kappa _0, k_0, \tau ) \gg 0$ such that, for $S \geqslant 2(s_0+\sigma )$ if (7.1) holds, then for all $s_0 \leqslant s \leqslant S/2 - \sigma$,

7.3 $$\begin{aligned} \Vert I_\delta -I_0 \Vert _{s}^{k_0,\upsilon }&\lesssim _s \left\| {\mathfrak {I}}_0 \right\| _{\mathcal {X}^{s+1}}^{k_0,\upsilon } \, , \end{aligned}$$

7.4 $$\begin{aligned} \Vert I_\delta -I_0 \Vert _{s}^{k_0,\upsilon }&\lesssim _s \upsilon ^{-1} \big ( \left\| Z \right\| _{\mathcal {Y}^s}^{k_0,\upsilon } +\left\| Z \right\| _{\mathcal {Y}^s}^{k_0,\upsilon } \left\| {\mathfrak {I}}_0 \right\| _{\mathcal {X}^{s + \sigma }}^{k_0,\upsilon } \big ) ,\nonumber \\ \Vert \mathcal {F}(i_\delta ,\alpha _0) \Vert _{\mathcal {Y}^s}^{k_0,\upsilon }&\lesssim _s \left\| Z \right\| _{\mathcal {Y}^{s + \sigma }}^{k_0,\upsilon } +\left\| Z \right\| _{\mathcal {Y}^{s + \sigma }}^{k_0,\upsilon } \left\| {\mathfrak {I}}_0 \right\| _{\mathcal {X}^{s + \sigma }}^{k_0,\upsilon } . \end{aligned}$$

Furthermore $i_\delta (\textbf{x})$ is a reversible torus, cfr. (4.45).

#### Proof

The estimates (7.3)–(7.4) follow for example as in Lemma 5.3 in [2]. $\square$

In order to find an approximate inverse of the linearized operator $\textrm{d}_{i,\alpha }\mathcal {F}(i_\delta )$, we introduce the symplectic diffeomorphism $G_\delta :(\phi ,{\mathfrak {y}},\texttt{z}) \rightarrow (\theta ,I,z)$ of the phase space ${{\mathbb {T}}}^{\kappa _0}\times {{\mathbb {R}}}^{\kappa _0} \times \mathcal {X}_\perp$,

7.5 $$\begin{aligned} \begin{pmatrix} \theta \\ I \\ z \end{pmatrix} := G_\delta \begin{pmatrix} \phi \\ {\mathfrak {y}}\\ \texttt{z}\end{pmatrix} := \begin{pmatrix} \theta _0(\phi ) \\ I_\delta (\phi ) + \left[ \partial _\phi \theta _0(\phi ) \right] ^{-\top }{\mathfrak {y}}+ \left[ (\partial _\theta {\widetilde{z}}_0)(\theta _0(\phi )) \right] ^\top J^{-1} \texttt{z}\\ z_0(\phi ) + \texttt{z}\end{pmatrix}, \nonumber \\ \end{aligned}$$

where ${\widetilde{z}}_0(\theta ):= z_0(\theta _0^{-1}(\theta ))$. It is proved in Lemma 2 of [7] that $G_\delta$ is symplectic, because the torus $i_\delta$ is isotropic (Lemma 7.2). In the new coordinates, $i_\delta$ is the trivial embedded torus $(\phi ,{\mathfrak {y}},\texttt{z})=(\phi ,0,0)$. The diffeomorphism $G_\delta$ in (7.5) is reversibility preserving.

Under the symplectic diffeomorphism $G_\delta$, the Hamiltonian vector field $X_{H_\alpha }$ changes into

7.6 $$\begin{aligned} X_{K_\alpha } = \left( DG_\delta \right) ^{-1} X_{H_\alpha } \circ G_\delta \qquad \textrm{where} \qquad K_\alpha := H_\alpha \circ G_\delta . \end{aligned}$$

We have that $K_\alpha$ is reversibility preserving, in the sense that

7.7 $$\begin{aligned} K_\alpha \circ \vec {\mathcal {S}} = K_\alpha . \end{aligned}$$

The Taylor expansion of $K_\alpha$ at the trivial torus $(\phi ,0,0)$ is

7.8 $$\begin{aligned} K_\alpha (\phi ,{\mathfrak {y}},\texttt{z}) =&\ K_{00}(\phi ,\alpha ) + K_{10}(\phi ,\alpha ) \cdot {\mathfrak {y}}+ ( K_{01}(\phi ,\alpha ),\texttt{z})_{L^2} + \tfrac{1}{2} K_{20}(\phi ) {\mathfrak {y}}\cdot {\mathfrak {y}}\nonumber \\&+ ( K_{11}(\phi ){\mathfrak {y}},\texttt{z})_{L^2} + \tfrac{1}{2} ( K_{02}(\phi )\texttt{z},\texttt{z})_{L^2} + K_{\geqslant 3}(\phi ,{\mathfrak {y}},\texttt{z}), \end{aligned}$$

where $$K_{\geqslant 3}$$ collects all terms at least cubic in the variables $$({\mathfrak {y}},\texttt{z})$$. By (4.42) and (7.5), the only Taylor coefficients that depend on $$\alpha $$ are $$K_{00}\in {{\mathbb {R}}}$$, $$K_{10}\in {{\mathbb {R}}}^{\kappa _0}$$ and $$K_{01}\in \mathcal {X}_\perp $$, whereas the $$ \kappa _0 \times \kappa _0 $$ symmetric matrix $$K_{20} $$, $$K_{11}\in \mathcal {L}( {{\mathbb {R}}}^{\kappa _0},\mathcal {X}_\perp )$$ and the linear self-adjoint operator $$ K_{02} $$, acting on $$\mathcal {X}_\perp $$, are independent of it.

Differentiating the identities in (7.7) at $(\phi ,0,0)$, we have

7.9 $$\begin{aligned}&K_{00}(-\phi ) = K_{00}(\phi ), \quad K_{10}(-\phi ) = K_{10}(\phi ), \quad K_{20}(-\phi ) = K_{20}(\phi ), \nonumber \\&\mathcal {S}\circ K_{01}(-\phi ) = K_{01}(\phi ), \quad \mathcal {S}\circ K_{11}(-\phi ) = K_{11}(\phi ), \quad K_{02}(-\phi )\circ \mathcal {S}= \mathcal {S}\circ K_{02}(\phi ). \end{aligned}$$

The Hamilton equations associated to (7.8) are

7.10 $$\begin{aligned} {\left\{ \begin{array}{ll} \dot{\phi } = K_{10}(\phi ,\alpha ) + K_{20}(\phi ){\mathfrak {y}}+ [K_{11}(\phi )]^\top \texttt{z}+ \partial _{{\mathfrak {y}}} K_{\geqslant 3}(\phi ,{\mathfrak {y}},\texttt{z}) \\ \dot{{\mathfrak {y}}} = - \partial _\phi K_{00}(\phi ,\alpha ) - [\partial _\phi K_{10}(\phi ,\alpha )]^\top {\mathfrak {y}}- [\partial _\phi K_{01}(\phi ,\alpha )]^\top \texttt{z}\\ \ \ \ \ \ - \partial _\phi \left( \tfrac{1}{2} K_{20}(\phi ){\mathfrak {y}}\cdot {\mathfrak {y}}+ \left( K_{11}(\phi ){\mathfrak {y}},\texttt{z}\right) _{L^2} + \tfrac{1}{2} \left( K_{02}(\phi )\texttt{z},\texttt{z}\right) _{L^2} + K_{\geqslant 3}(\phi ,{\mathfrak {y}},\texttt{z}) \right) \\ \dot{\texttt{z}} = J\, \left( K_{01}(\phi ,\alpha )+ K_{11}(\phi ){\mathfrak {y}}+ K_{02}(\phi )\texttt{z}+ \nabla _{\texttt{z}} K_{\geqslant 3}(\phi ,{\mathfrak {y}},\texttt{z}) \right) \end{array}\right. },\nonumber \\ \end{aligned}$$

where $\partial _\phi K_{10}^\top$ is the $\kappa _0\times \kappa _0$ transposed matrix and $\partial _\phi K_{01}^\top , K_{11}^\top : \mathcal {X}_\perp \rightarrow {{\mathbb {R}}}^{\kappa _0}$ are defined by the duality relation $(\partial _\phi K_{01}[{\widehat{\phi }}],\texttt{z})_{L^2}={\widehat{\phi }}\cdot [\partial _\phi K_{01} ]^\top \texttt{z}$ for any ${\widehat{\phi }}\in {{\mathbb {R}}}^{\kappa _0}$, $\texttt{z}\in \mathcal {X}_\perp$. The transpose $K_{11}^\top (\phi )$ is defined similarly. On an exact solution (that is $Z=0$), the terms $K_{00}, K_{01}$ in the Taylor expansion (7.8) vanish and $K_{10}= \omega$. More precisely, arguing as in Lemma 5.4 in [2] (with minor adaptations), we have

#### Lemma 7.3

There is $\sigma := \sigma (\kappa _0, k_0, \tau ) > 0$, such that if $S \gg 2(s_0 + \sigma )$ and (7.1) holds then for all $s_0 \leqslant s \leqslant S/2 - \sigma$, one has

$$\begin{aligned}&\Vert \partial _\phi K_{00}(\cdot , \alpha _0)\Vert _{s}^{k_0, \upsilon } + \Vert K_{10}(\cdot ,\alpha _0)-\omega \Vert _{s}^{k_0, \upsilon } + \Vert K_{01}( \cdot ,\alpha _0) \Vert _{\mathcal {Y}^s_\bot }^{k_0, \upsilon } \\&\quad \lesssim _s \left\| Z \right\| _{\mathcal {Y}^{s+\sigma }}^{k_0,\upsilon } + \left\| Z \right\| _{\mathcal {Y}^{s+\sigma }}^{k_0,\upsilon } \left\| {\mathfrak {I}}_0 \right\| _{\mathcal {Y}^{s+\sigma }}^{k_0,\upsilon } , \\&\left\| \partial _\alpha K_{00} \right\| _{s}^{k_0,\upsilon } + \left\| \partial _\alpha K_{10}-\textrm{Id} \right\| _{s}^{k_0,\upsilon } + \left\| \partial _\alpha K_{01} \right\| _{\mathcal {Y}^s_\bot }^{k_0,\upsilon } \lesssim _s \Vert {\mathfrak {I}}_0 \Vert _{\mathcal {X}^{s + \sigma }}^{k_0,\upsilon } ,\\&\left\| K_{20} \right\| _{s}^{k_0,\upsilon }\lesssim _s \sqrt{\varepsilon } ( 1 + \left\| {\mathfrak {I}}_0 \right\| _{\mathcal {X}^{s+\sigma }}^{k_0,\upsilon } ), \\&\left\| K_{11}y \right\| _{\mathcal {Y}^s_\bot }^{k_0,\upsilon } \lesssim _s \sqrt{\varepsilon } ( \left\| y \right\| _s^{k_0,\upsilon }+ \left\| y \right\| _{s_0}^{k_0,\upsilon }\left\| {\mathfrak {I}}_0 \right\| _{\mathcal {X}^{s+\sigma }}^{k_0,\upsilon } ), \\&\left\| K_{11}^\top \texttt{z} \right\| _{s}^{k_0,\upsilon } \lesssim _s \sqrt{\varepsilon } ( \left\| \texttt{z} \right\| _{\mathcal {Y}^s_\bot }^{k_0,\upsilon } + \left\| \texttt{z} \right\| _{\mathcal {Y}^{s_0}_\bot }^{k_0,\upsilon }\left\| {\mathfrak {I}}_0 \right\| _{\mathcal {X}^{s+\sigma }}^{k_0,\upsilon }) \, . \end{aligned}$$

Under the linear change of variables

7.11 $$\begin{aligned} DG_\delta (\textbf{x},0,0)\begin{pmatrix} {\widehat{\phi }}\\ {\widehat{{\mathfrak {y}}}}\\ {\widehat{\texttt{z}}}\end{pmatrix}:= \begin{pmatrix} \partial _\phi \theta _0(\textbf{x}) &  0 &  0 \\ \partial _\phi I_\delta (\textbf{x}) &  [\partial _\phi \theta _0(\textbf{x})]^{-\top } &  [(\partial _\theta {\widetilde{w}}_0)(\theta _0(\textbf{x}))]^\top J^{-1} \\ \partial _\phi w_0(\textbf{x}) &  0 &  \textrm{Id} \end{pmatrix}\begin{pmatrix} {\widehat{\phi }}\\ {\widehat{{\mathfrak {y}}}}\\ {\widehat{\texttt{z}}}\end{pmatrix} , \nonumber \\ \end{aligned}$$

the linearized operator $\textrm{d}_{i,\alpha }\mathcal {F}(i_\delta )$ is approximately transformed into the one obtained when one linearizes the Hamiltonian system (7.10) at $(\phi ,{\mathfrak {y}},\texttt{z}) = (\textbf{x},0,0)$, differentiating also in $\alpha$ at $\alpha _0$ and changing $\partial _x \rightsquigarrow \omega \cdot \partial _\textbf{x}$, namely

7.12 $$\begin{aligned} \begin{pmatrix} {\widehat{\phi }} \\ {\widehat{{\mathfrak {y}}}} \\ {\widehat{\texttt{z}}} \\ {\widehat{\alpha }} \end{pmatrix} \mapsto \begin{pmatrix} \omega \cdot \partial _\textbf{x}{\widehat{\phi }}- \partial _\phi K_{10}(\textbf{x})[{\widehat{\phi }}] - \partial _\alpha K_{10}(\textbf{x})[{\widehat{\alpha }}] - K_{20}(\textbf{x}){\widehat{{\mathfrak {y}}}}- [K_{11}(\textbf{x})]^\top {\widehat{\texttt{z}}}\\ \omega \cdot \partial _\textbf{x}{\widehat{{\mathfrak {y}}}}+ \partial _{\phi \phi }K_{00}(\textbf{x})[{\widehat{\phi }}]+ \partial _\alpha \partial _\phi K_{00}(\textbf{x})[{\widehat{\alpha }}] + [\partial _\phi K_{10}(\textbf{x})]^\top {\widehat{{\mathfrak {y}}}}+ [\partial _\phi K_{01}(\textbf{x})]^\top {\widehat{\texttt{z}}}\\ \omega \cdot \partial _\textbf{x}{\widehat{\texttt{z}}}- J \, \big ( \partial _\phi K_{01}(\textbf{x})[{\widehat{\phi }}] + \partial _\alpha K_{01}(\textbf{x})[{\widehat{\alpha }}] + K_{11}(\textbf{x}) {\widehat{{\mathfrak {y}}}}+ K_{02}(\textbf{x}) {\widehat{\texttt{z}}}\big ) \end{pmatrix}.\nonumber \\ \end{aligned}$$

In order to construct an approximate inverse of (7.12), we need the operator

7.13 $$\begin{aligned} \mathcal {G}_\omega := \Pi ^\perp \left( \omega \cdot \partial _\textbf{x}- J K_{02}(\textbf{x}) \right) |_{\mathcal {X}_\perp } \end{aligned}$$

to be invertible (on reversible tori), where we recall that $\Pi ^\perp$ denotes the projection on the invariant subspace $\mathcal {X}_{\perp }$ in (4.30).

#### Lemma 7.4

There exists $\sigma := \sigma (k_0, \kappa _0, \tau ) > 0$ such that, if (7.1) holds, then, for $S\geqslant 2(s_0+\sigma )$, for any $s_0 \leqslant s \leqslant S/2 -\sigma$ and

for any $h\in \mathcal {Y}_\bot ^{s+1}$, there exists a solution $f := \mathcal {G}_\omega ^{- 1} h \in \mathcal {X}_\perp ^{s}$ of the equation $\mathcal {G}_\omega f = h$ satisfying

$$\begin{aligned} \Vert (\mathcal {G}_\omega )^{-1} h \Vert _{\mathcal {X}^s_\bot }^{k_0,\upsilon }\lesssim _{s} \Vert h \Vert _{\mathcal {Y}^{s + 1}_\bot }^{k_0,\upsilon }+ \Vert {{\mathfrak {I}}}_0 \Vert _{\mathcal {X}^{s + \sigma }}^{k_0, \upsilon } \Vert h \Vert _{\mathcal {Y}^{s_0 + 1}_\bot }^{k_0, \upsilon }. \end{aligned}$$

Moreover, if *h* is anti-reversible, then *f* is reversible.

We postpone the proof of this lemma to Sect. 7.2. To find an approximate inverse of the linear operator in (7.12) (and so of $\textrm{d}_{i,\alpha }\mathcal {F}(i_\delta )$), it is enough to invert the operator

7.14 $$\begin{aligned} {{\mathbb {D}}}\big [ {\widehat{\phi }},{\widehat{{\mathfrak {y}}}},{\widehat{\texttt{z}}},{\widehat{\alpha }}\big ]:=\begin{pmatrix} \omega \cdot \partial _\textbf{x}{\widehat{\phi }}- \partial _\alpha K_{10}(\textbf{x})[{\widehat{\alpha }}] - K_{20}(\textbf{x}){\widehat{{\mathfrak {y}}}}- K_{11}^\top (\textbf{x}){\widehat{\texttt{z}}}\\ \omega \cdot \partial _\textbf{x}{\widehat{{\mathfrak {y}}}}+\partial _\alpha \partial _\phi K_{00}(\textbf{x})[{\widehat{\alpha }}] \\ \mathcal {G}_\omega {\widehat{\texttt{z}}}- J \left( \partial _\alpha K_{01}(\textbf{x})[{\widehat{\alpha }}] + K_{11}(\textbf{x}){\widehat{{\mathfrak {y}}}}\right) \end{pmatrix} \end{aligned}$$

obtained neglecting in (7.12) the terms $\partial _\phi K_{10}$, $\partial _{\phi \phi }K_{00}$, $\partial _\phi K_{00}$, $\partial _\phi K_{01}$, (as they vanish at an exact solution). For $(\omega ,\texttt{A})\in \texttt{G}^{\upsilon } \times \mathcal {J}_{\varepsilon }(\texttt{E})$ (recall (4.54) and (1.21)), we look for an inverse of ${{\mathbb {D}}}$ by solving the system

7.15 $$\begin{aligned} {{\mathbb {D}}}\big [ {\widehat{\phi }},{\widehat{{\mathfrak {y}}}},{\widehat{\texttt{z}}},{\widehat{\alpha }}\big ] = \begin{pmatrix} g_1 \\ g_2 \\ g_3 \end{pmatrix}, \end{aligned}$$

where $(g_1, g_2, g_3)$ is an anti-reversible torus, that is

7.16 $$\begin{aligned}&g_1(\textbf{x}) = g_1(- \textbf{x}) , \qquad g_2(\textbf{x}) = - g_2(- \textbf{x}) , \qquad \mathcal {S}g_3(\textbf{x}) = - g_3(- \textbf{x}) \, . \end{aligned}$$

We start with the second equation in (7.14)–(7.15), that is $\omega \cdot \partial _\textbf{x}{\widehat{{\mathfrak {y}}}}= g_2-\partial _\alpha \partial _\phi K_{00}(\textbf{x})[{\widehat{\alpha }}]$. By (7.16) and (7.9), the right hand side of this equation is odd in $\textbf{x}$. In particular it has zero average and so, for $\omega \in \texttt{G}^\upsilon$ (recall (4.54)), one can set

7.17 $$\begin{aligned} {\widehat{{\mathfrak {y}}}}:= (\omega \cdot \partial _\textbf{x})^{-1} ( g_2 -\partial _\alpha \partial _\phi K_{00}(\textbf{x})[{\widehat{\alpha }}] ) \, . \end{aligned}$$

Next, we consider the third equation $\mathcal {G}_\omega {\widehat{\texttt{z}}}= g_3 + J ( \partial _\alpha K_{01}(\textbf{x})[{\widehat{\alpha }}]+ K_{11}(\textbf{x}){\widehat{{\mathfrak {y}}}})$. By Lemma 7.4, there is an anti-reversible solution

7.18 $$\begin{aligned} {\widehat{\texttt{z}}}:= ( \mathcal {G}_\omega )^{-1} \big ( g_3 + J ( \partial _\alpha K_{01}(\textbf{x})[{\widehat{\alpha }}]+ K_{11}(\textbf{x}){\widehat{{\mathfrak {y}}}}) \big ) \, . \end{aligned}$$

Finally, we solve the first equation in (7.15), which, inserting (7.17) and (7.18), becomes

7.19 $$\begin{aligned} \omega \cdot \partial _\textbf{x}{\widehat{\phi }}= g_1 + M_1(\textbf{x})[{\widehat{\alpha }}]+ M_2(\textbf{x})g_2 + M_3(\textbf{x})g_3, \end{aligned}$$

where

$$\begin{aligned} M_1(\textbf{x})&:= \partial _\alpha K_{10}(\textbf{x}) - M_2(\textbf{x})\partial _\alpha \partial _\phi K_{00}(\textbf{x}) + M_3(\textbf{x}) J\, \partial _\alpha K_{01} (\textbf{x}) ,\\ M_2(\textbf{x})&:= K_{20}(\textbf{x}) (\omega \cdot \partial _\textbf{x})^{-1} + K_{11}^\top (\textbf{x})\left( \mathcal {G}_\omega \right) ^{-1} J\, K_{11}(\textbf{x})(\omega \cdot \partial _\textbf{x})^{-1}, \\ M_3(\textbf{x})&:= K_{11}^\top (\textbf{x})\left( \mathcal {G}_\omega \right) ^{-1} . \end{aligned}$$

In order to solve (7.19), we choose ${\widehat{\alpha }}$ such that the average in $\textbf{x}$ of the right hand side is zero. The $\textbf{x}$-average of the matrix $M_1$ satisfies $\mathinner {\langle {M_1}\rangle }_\textbf{x}= \textrm{Id} + O(\sqrt{\varepsilon }\upsilon ^{-1})$. Then, for $\sqrt{\varepsilon }\upsilon ^{-1}$ small enough, $\mathinner {\langle {M_1}\rangle }_\textbf{x}$ is invertible and $\mathinner {\langle {M_1}\rangle }_\textbf{x}^{-1} = \textrm{Id} + O(\sqrt{\varepsilon }\upsilon ^{-1})$. Thus we define

7.20 $$\begin{aligned} {\widehat{\alpha }}:= -\mathinner {\langle {M_1}\rangle }_\textbf{x}^{-1}\big ( \mathinner {\langle {g_1}\rangle }_\textbf{x}+ \mathinner {\langle {M_2g_2}\rangle }_\textbf{x}+ \mathinner {\langle {M_3 g_3}\rangle }_\textbf{x}\big ) \, , \end{aligned}$$

and the solution of Equation (7.19)

7.21 $$\begin{aligned} {\widehat{\phi }}:= ( \omega \cdot \partial _\textbf{x})^{-1}\big ( g_1 + M_1(\textbf{x})[{\widehat{\alpha }}] + M_2(\textbf{x})g_2 + M_3(\textbf{x})g_3 \big )\, \end{aligned}$$

for $\omega \in \texttt{G}^\upsilon$. Moreover, using (7.16), (7.9), the fact that *J* and $\mathcal {S}$ anti-commutes and Lemma 7.4, one checks that $({\widehat{\phi }}, {\widehat{{\mathfrak {y}}}}, {\widehat{\texttt{z}}})$ is reversible, that is

7.22 $$\begin{aligned} {\widehat{\phi }}(\textbf{x}) = - {\widehat{\phi }}(- \textbf{x}) , \quad {\widehat{{\mathfrak {y}}}}(\textbf{x}) = {\widehat{{\mathfrak {y}}}}(- \textbf{x}) , \quad \mathcal {S}{\widehat{\texttt{z}}}(\textbf{x}) = {\widehat{\texttt{z}}}(- \textbf{x}) \, . \end{aligned}$$

In conclusion, we have obtained a solution $( {\widehat{\phi }},{\widehat{{\mathfrak {y}}}},{\widehat{\texttt{z}}},{\widehat{\alpha }})$ of the linear system (7.15), and, denoting the norm $\Vert (\phi ,{\mathfrak {y}},\texttt{z},\alpha ) \Vert _{\mathcal {X}^s}^{k_0,\upsilon }:= \max \big \{ \Vert (\phi ,{\mathfrak {y}},\texttt{z}) \Vert _{\mathcal {X}^s}^{k_0,\upsilon },\left| \alpha \right| ^{k_0,\upsilon } \big \}$ (where $\mathcal {X}^s$ is defined in (4.48)), we have

#### Proposition 7.5

For all $$(\omega ,\texttt{A})\in \texttt{G}^{\upsilon }\times \mathcal {J}_{\varepsilon }(\texttt{E})$$ and for any anti-reversible torus variation $$ g =(g_1,g_2,g_3)$$ (that is satisfying (7.16)), the linear system (7.15) has a solution $${{\mathbb {D}}}^{-1}g:= ( {\widehat{\phi }},{\widehat{{\mathfrak {y}}}},{\widehat{\texttt{z}}},{\widehat{\alpha }})$$, with $$ ( {\widehat{\phi }},{\widehat{{\mathfrak {y}}}},{\widehat{\texttt{z}}},{\widehat{\alpha }})$$ defined in (7.21), (7.17), (7.18), (7.20), where $$( {\widehat{\phi }},{\widehat{{\mathfrak {y}}}},{\widehat{\texttt{z}}})$$ is a reversible torus variation, satisfying, for any $$s_0\leqslant s\leqslant S/2 - \sigma $$

$$\begin{aligned} \Vert {{\mathbb {D}}}^{-1}g \Vert _{\mathcal {X}^s}^{k_0,\upsilon } \lesssim _{S} \upsilon ^{-1}\big ( \Vert g \Vert _{\mathcal {Y}^{s + \sigma }}^{k_0,\upsilon }+\Vert g \Vert _{\mathcal {Y}^{s_0+ \sigma }}^{k_0,\upsilon }\Vert {\mathfrak {I}}_0 \Vert _{\mathcal {X}^{s+\sigma }}^{k_0,\upsilon } \big ) . \end{aligned}$$

Finally, we prove that the operator

7.23 $$\begin{aligned} \textbf{T}_0 := \textbf{T}_0(i_0):= ( D{\widetilde{G}}_\delta )(\textbf{x},0,0) \circ {{\mathbb {D}}}^{-1} \circ (D G_\delta ) (\textbf{x},0,0)^{-1} \end{aligned}$$

is an approximate right inverse for $\textrm{d}_{i,\alpha }\mathcal {F}(i_0)$, where ${\widetilde{G}}_\delta (\phi ,{\mathfrak {y}},\texttt{z},\alpha ) := \left( G_\delta (\phi ,{\mathfrak {y}},\texttt{z}),\right. \left. \alpha \right)$ is the identity on the $\alpha$-component.

#### Theorem 7.6

(Approximate inverse) There is ${\overline{\sigma }} :={\overline{\sigma }}(\tau ,\kappa _0,k_0)>0$ such that, if (7.1) holds with $\sigma ={\overline{\sigma }}$, then, for all $(\omega ,\texttt{A})\in \texttt{G}^{\upsilon }\times \mathcal {J}_{\varepsilon }(\texttt{E})$ (recall (4.54) and (1.21)) and for any anti-reversible torus variation $g:=(g_1,g_2,g_3)$ (that is satisfying (7.16)), the operator $\textbf{T}_0$ defined in (7.23) satisfies, for $S\geqslant 2(s_0+{\overline{\sigma }})$ and for all $s_0 \leqslant s \leqslant S/2 - {\overline{\sigma }}$,

7.24 $$\begin{aligned} \begin{aligned}&\Vert \textbf{T}_0 g \Vert _{\mathcal {X}^s}^{k_0,\upsilon } \lesssim _{s} \upsilon ^{-1} \big ( \Vert g \Vert _{\mathcal {Y}^{s + {\overline{\sigma }}}}^{k_0,\upsilon } +\Vert g \Vert _{\mathcal {Y}^{s_0 + {\overline{\sigma }}}}^{k_0,\upsilon }\Vert {\mathfrak {I}}_0 \Vert _{\mathcal {X}^{s + {\overline{\sigma }}}}^{k_0,\upsilon } \big ), \\&\Vert \textbf{T}_0 g \Vert _{\mathcal {Y}^s}^{k_0,\upsilon } \lesssim _{s} \upsilon ^{-1} \big ( \Vert g \Vert _{\mathcal {Y}^{s + {\overline{\sigma }}}}^{k_0,\upsilon } +\Vert g \Vert _{\mathcal {Y}^{s_0 + {\overline{\sigma }}}}^{k_0,\upsilon }\Vert {\mathfrak {I}}_0 \Vert _{\mathcal {X}^{s + {\overline{\sigma }}}}^{k_0,\upsilon } \big ) \end{aligned} \end{aligned}$$

Moreover, the first three components of $\textbf{T}_0 g$ form a reversible torus variation (that is satisfy (7.22)). Finally, $\textbf{T}_0$ is an approximate right inverse of $\textrm{d}_{i,\alpha }\mathcal {F}(i_0)$, namely

$$\begin{aligned} \textrm{d}_{i,\alpha }\mathcal {F}(i_0) \circ \textbf{T}_0 - \textrm{Id} = \mathcal {P}(i_0) \end{aligned}$$

where, for any variation $g \in \mathcal {Y}^{s_0 + {\overline{\sigma }}}$, one has

7.25 $$\begin{aligned} \begin{aligned} \Vert \mathcal {P}(i_0) g \Vert _{\mathcal {Y}^{s_0}}^{k_0,\upsilon }&\lesssim \upsilon ^{-1} \Vert \mathcal {F}(i_0,\alpha _0) \Vert _{\mathcal {Y}^{s_0 + {\overline{\sigma }}}}^{k_0,\upsilon }\Vert g \Vert _{\mathcal {Y}^{s_0+{\overline{\sigma }}}}^{k_0,\upsilon } \end{aligned} \end{aligned}$$

#### Proof

The claim that the first three components of $\textbf{T}_0 g$, with $\textbf{T}_0$ as in (7.23), form a reversible torus variation follows from the facts that $DG_\delta (\textbf{x},0,0)$, $DG_\delta (\textbf{x},0,0)^{-1}$ are reversibility preserving and that ${{\mathbb {D}}}^{-1}$ maps anti-reversible torus variations into reversible torus variations, see Proposition 7.5.

First, we note that $DG_\delta (\textbf{x},0,0)$, defined in (7.11), satisfies the estimates, by Lemma 7.2 and (7.1), with $\mathcal {Z}^{s} = \mathcal {X}^{s}$ or $\mathcal {Z}^{s}= \mathcal {Y}^{s}$,

7.26 $$\begin{aligned}&\Vert DG_\delta (\textbf{x},0,0)[{{\widehat{i}}}] \Vert _{\mathcal {Z}^s}^{k_0,\upsilon }\! +\! \Vert DG_\delta (\textbf{x},0,0)^{-1}[{{\widehat{i}}}] \Vert _{\mathcal {Z}^s}^{k_0,\upsilon } \lesssim _{s} \Vert {{\widehat{i}}}\Vert _{\mathcal {Z}^s}^{k_0,\upsilon } + \Vert {\mathfrak {I}}_0 \Vert _{\mathcal {X}^{s+\sigma }}^{k_0,\upsilon }\Vert {{\widehat{i}}}\Vert _{\mathcal {Z}^{s_0}}^{k_0,\upsilon }, \end{aligned}$$

7.27 $$\begin{aligned}&\Vert D^2G_\delta (\textbf{x},0,0)[{{\widehat{i}}}_1,{{\widehat{i}}}_2] \Vert _{\mathcal {Z}^s}^{k_0,\upsilon } \lesssim _{s} \Vert {{\widehat{i}}}_1 \Vert _{\mathcal {Z}^s}^{k_0,\upsilon } \Vert {{\widehat{i}}}_2 \Vert _{\mathcal {Z}^{s_0}}^{k_0,\upsilon } + \Vert {{\widehat{i}}}_1 \Vert _{\mathcal {Z}^{s_0}}^{k_0,\upsilon } \Vert {{\widehat{i}}}_2 \Vert _{\mathcal {Z}^{s}}^{k_0,\upsilon } \nonumber \\&\quad + \Vert {\mathfrak {I}}_0 \Vert _{\mathcal {X}^{s+\sigma }}^{k_0,\upsilon } \Vert {{\widehat{i}}}_1 \Vert _{\mathcal {Z}^{s_0}}^{k_0,\upsilon } \Vert {{\widehat{i}}}_2 \Vert _{\mathcal {Z}^{s_0}}^{k_0,\upsilon }, \end{aligned}$$

for any variation ${{\widehat{i}}}:=({\widehat{\phi }},{\widehat{{\mathfrak {y}}}},{\widehat{\texttt{z}}})$ and for some $\sigma =\sigma (k_0, \kappa _0, \tau )>0$. These latter estimates, together with Proposition 7.5 imply the first estimate in (7.24). The second estimate easily follows from the first one by recalling the property (4.49).

We now compute the operator $\mathcal {P}$ and prove the estimate (7.25). By (4.43) and Lemma 7.2, since $X_\mathcal {N}$ is independent of the action *I*, we have

7.28 $$\begin{aligned} \textrm{d}_{i,\alpha } \mathcal {F}(i_0) - \textrm{d}_{i,\alpha }\mathcal {F}(i_\delta )= &   \sqrt{\varepsilon } \int _{0}^{1} \partial _I \textrm{d}_i X_{\mathcal {P}_\varepsilon }(\theta _0,I_\delta + \lambda (I_0-I_\delta ),z_0)\nonumber \\  &   [I_0-I_\delta ,\Pi [\,\cdot \,]] \,\textrm{d}{\lambda }=: \mathcal {E}_0 , \end{aligned}$$

where $\Pi$ throughout this proof denotes the projection $({{\widehat{i}}},{\widehat{\alpha }})\rightarrow {{\widehat{i}}}$. We denote by $\texttt{u}:=(\phi ,{\mathfrak {y}},\texttt{z})$ the symplectic coordinates induced by $G_\delta$ in (7.5). Under the symplectic map $G_\delta$, the nonlinear operator $\mathcal {F}$ in (4.43) is transformed into

$$\begin{aligned} \mathcal {F}(G_\delta (\texttt{u}(\textbf{x})),\alpha ) = DG_\delta (\texttt{u}(\textbf{x}))\big (\omega \cdot \partial _{\textbf{x}} \texttt{u}(\textbf{x}) -X_{K_\alpha } (\texttt{u}(\textbf{x}),\alpha ) \big ) , \end{aligned}$$

with $K_\alpha = H_\alpha \circ G_\delta$ as in (7.6). By differentiating at the trivial torus $\texttt{u}_\delta (\textbf{x}):= G_{\delta }^{-1}(i_\delta )(\textbf{x}) = (\textbf{x},0,0)$ and at $\alpha =\alpha _0$, we get

7.29 $$\begin{aligned} \textrm{d}_{i,\alpha }\mathcal {F}(i_\delta ) = DG_\delta (\texttt{u}_\delta (\textbf{x}))\big (\omega \cdot \partial _{\textbf{x}} -\textrm{d}_{\texttt{u},\alpha }X_{K_\alpha } (\texttt{u}_\delta (\textbf{x}),\alpha _0) \big ) D{\widetilde{G}}_\delta (\texttt{u}_\delta )^{-1} + \mathcal {E}_1 , \nonumber \\ \end{aligned}$$

where

7.30 $$\begin{aligned} \mathcal {E}_1:= D^2 G_\delta (\texttt{u}_\delta ) [DG_\delta (\texttt{u}_\delta )^{-1} \mathcal {F}(i_\delta ,\alpha _0), DG_\delta (\texttt{u}_\delta )^{-1}\Pi [\,\cdot \,] ]. \end{aligned}$$

Furthermore, by (7.12), (7.13), (7.14), we split

7.31 $$\begin{aligned} \omega \cdot \partial _{\textbf{x}} -\textrm{d}_{\texttt{u},\alpha }X_{K_\alpha } (\texttt{u}_\delta (\textbf{x}),\alpha _0) = {{\mathbb {D}}}+ R_Z, \end{aligned}$$

where

7.32 $$\begin{aligned} R_Z[{\widehat{\phi }},{\widehat{{\mathfrak {y}}}},{\widehat{\texttt{z}}},{\widehat{\alpha }}]:=\begin{pmatrix} - \partial _\phi K_{10}(\textbf{x})[{\widehat{\phi }}] \\ \partial _{\phi \phi }K_{00}(\textbf{x})[{\widehat{\phi }}] + [\partial _\phi K_{10}(\textbf{x})]^\top {\widehat{{\mathfrak {y}}}}+ [\partial _\phi K_{01}(\textbf{x})]^\top {\widehat{\texttt{z}}}\\ - J \partial _\phi K_{01}(\textbf{x})[{\widehat{\phi }}] \end{pmatrix}.\qquad \end{aligned}$$

Summing up (7.28), (7.29) and (7.31), we get the decomposition

7.33 $$\begin{aligned} \textrm{d}_{i,\alpha }\mathcal {F}(i_0) = DG_\delta (\texttt{u}_\delta )\circ {{\mathbb {D}}}\circ D{\widetilde{G}}_\delta (\texttt{u}_\delta )^{-1} + \mathcal {E}, \end{aligned}$$

where

7.34 $$\begin{aligned} \mathcal {E}:= \mathcal {E}_0 + \mathcal {E}_1 + DG_\delta (\texttt{u}_\delta )\circ R_Z\circ D{\widetilde{G}}_\delta (\texttt{u}_\delta )^{-1}, \end{aligned}$$

with $\mathcal {E}_0$, $\mathcal {E}_1$ and $R_Z$ defined in (7.28), (7.30), (7.32), respectively. Applying $\textbf{T}_0$ defined in (7.23) to the right of (7.33), since ${{\mathbb {D}}}\circ {{\mathbb {D}}}^{-1}=\textrm{Id}$ by Proposition 7.5, we get

$$\begin{aligned} \textrm{d}_{i,\alpha }\mathcal {F}(i_0)\circ \textbf{T}_0 - \textrm{Id} = \mathcal {P}, \quad \mathcal {P}:= \mathcal {E}\circ \textbf{T}_0. \end{aligned}$$

By (7.34), Lemmata 7.1, 7.2 and (7.1), (7.26), (7.27) and using the ansatz (7.1), we obtain the estimate

7.35 $$\begin{aligned} \Vert \mathcal {E}[{{\widehat{i}}},{\widehat{\alpha }}] \Vert _{\mathcal {Y}^{s_0}}^{k_0,\upsilon } \lesssim \Vert Z \Vert _{\mathcal {Y}^{s_0+\sigma }}^{k_0,\upsilon } \Vert {{\widehat{i}}}\Vert _{\mathcal {Y}^{s_0+\sigma }}^{k_0,\upsilon }, \end{aligned}$$

where $Z=\mathcal {F}(i_0,\alpha _0)$, recall (7.2). The estimate on $\mathcal {P}$ then follows by (7.35) and the second estimate in (7.24), assuming (7.1) for some ${\overline{\sigma }} \gg \sigma \gg 0$ large enough. $\square$

### Invertibility of the Operator $\mathcal {G}_{\omega }$ and Proof of Lemma 7.4

In this section we prove the invertibility of the operator $\mathcal {G}_\omega$ as a bounded operator $\mathcal {X}_\perp ^{s + 1} \rightarrow \mathcal {Y}^s_\bot$ (recall their definitions in (4.47)). First, we write an explicit expression of the linear operator $\mathcal {G}_{\omega }$, defined in (7.13), as the projection on the normal directions of the linearized Hamilton equation (4.43) at the approximate solution, up to a remainder with finite rank, therefore bounded and small.

#### Lemma 7.7

The Hamiltonian operator $\mathcal {G}_\omega$ in (7.13), acting on the subspace $\mathcal {X}_\perp ^s$, has the form

7.36 $$\begin{aligned} \mathcal {G}_\omega = \Pi ^\perp (\mathcal {G}- \sqrt{\varepsilon } J R)|_{\mathcal {X}^s_\perp } , \end{aligned}$$

where:

(*i*) $\mathcal {G}$ is the Hamiltonian operator

7.37 $$\begin{aligned} \mathcal {G}:= \omega \cdot \partial _\textbf{x}- J \partial _\zeta \nabla _\zeta H_\varepsilon (y,T_\delta (\phi )) \, , \end{aligned}$$

where $H_\varepsilon$ is the Hamiltonian in (4.28) evaluated at

7.38 $$\begin{aligned} T_\delta (\phi ):= A ( i_\delta (\phi ) ) = A\left( \theta _0(\phi ),I_\delta (\textbf{x}),z_0(\phi ) \right) = v^\intercal \left( \theta _0(\phi ),I_\delta (\phi ) \right) + z_0(\phi ),\nonumber \\ \end{aligned}$$

the torus $i_\delta (\phi ):= ( \theta _0(\phi ),I_\delta (\phi ),z_0(\phi ) )$ is as in Lemma 7.2 and $A(\theta ,I,z)$, $v^\intercal (\theta ,I)$ in (4.33);

(*ii*) $R (\phi )$ is Hamiltonian and it has the finite rank form $R(\phi )[h] = \sum _{j=1}^{\kappa _0} \left( h,g_j \right) _{L^2} \chi _j$ for any $h(\textbf{x}, y)$, for functions $g_j,\chi _j \in \mathcal {Y}^s_\bot$ that satisfy, for some $\sigma := \sigma (\tau ,\kappa _0, k_0) > 0$, for $S\geqslant 2(s_0+\sigma )$, for all $j = 1, \ldots , \kappa _0$ and for all $s_0\leqslant s \leqslant S/2 - \sigma$,

7.39 $$\begin{aligned} \begin{aligned} \left\| g_j \right\| _{\mathcal {Y}^s_\bot }^{k_0,\upsilon } + \left\| \chi _j \right\| _{\mathcal {Y}^s_\bot }^{k_0,\upsilon }&\lesssim _s 1 + \left\| {\mathfrak {I}}_0 \right\| _{\mathcal {X}^{s + \sigma }}^{k_0,\upsilon } . \end{aligned} \end{aligned}$$

Furthermore, the operators $\mathcal {G}_\omega$, $\mathcal {G}$, *R* are reversible.

#### Proof

The claims are proved as in Lemma 7.1 in [8] and Lemma 6.1 in [12]. We refer to these articles for more details. $\square$

The operator $\mathcal {G}$ in (7.37) is obtained by linearizing the original system (4.27) at any torus

$$\begin{aligned} \begin{aligned} {\overline{\zeta }}(\textbf{x})=({\overline{\zeta }}_1(\textbf{x}),{\overline{\zeta }}_2(\textbf{x}))= T_\delta (\textbf{x}), \end{aligned} \end{aligned}$$

with $T_\delta (\textbf{x})$ as in (7.38). Explicitly, we have

7.40 $$\begin{aligned} \mathcal {G}= \omega \cdot \partial _\textbf{x}- \begin{pmatrix} 0 &  \textrm{Id} \\ \mathcal {L}_{{\mathfrak {m}}} + a(\textbf{x},y) &  0 \end{pmatrix}, \quad a(\textbf{x}, y) := \sqrt{\varepsilon }q_\varepsilon (y, {\overline{\zeta }}_1( \textbf{x}, y)) \end{aligned}$$

#### Proof of Lemma 7.4

First, we estimate the norm of the function $a(\textbf{x},y)$. By Moser composition estimates in Lemma 2.2, we have that the norm of $\overline{\zeta }$ satisfies $\Vert {\overline{\zeta }}\Vert _{s, 1}^{k_0,\upsilon } \lesssim _s 1+\Vert {\mathfrak {I}}_0\Vert _{\mathcal {X}^s}^{k_0,\upsilon }$ and hence by the ansatz (7.1), $\Vert {\overline{\zeta }} \Vert _{s_0, 1}^{k_0, \upsilon } \lesssim 1$. Thus, by estimates (4.25), we get, for any $s_0 \leqslant s \leqslant S/2 - \sigma$,

7.41 $$\begin{aligned} \Vert a \Vert _{s, 1}^{k_0,\upsilon } \lesssim _s \sqrt{\varepsilon } (1+\Vert {\mathfrak {I}}_0\Vert _{\mathcal {X}^{s + \sigma }}^{k_0,\upsilon }) . \end{aligned}$$

Now, we want to solve the equation $\mathcal {G}_{\omega } f=h$. By (7.36) (7.40) we write $\mathcal {G}_\omega = \mathcal {D}_\omega + \mathcal {R}_\varepsilon$, where

7.42 $$\begin{aligned} \begin{aligned} \mathcal {D}_\omega := \Pi ^{\perp } \Big ( \omega \cdot \partial _\textbf{x}-\begin{pmatrix} 0 &  \textrm{Id} \\ \mathcal {L}_{{\mathfrak {m}}} &  0 \end{pmatrix} \Big ) \Pi ^{\perp }, \ \ \mathcal {R}_\varepsilon := - \Pi ^\perp \Big ( \begin{pmatrix} 0 &  0 \\ a &  0 \end{pmatrix} + \sqrt{\varepsilon } J R \Big )\Pi ^{\perp }, \end{aligned} \end{aligned}$$

where $JR(\phi )$ is the finite rank operator in Lemma 7.7-(*ii*). In order to invert $\mathcal {G}_{\omega }$, we conjugate it with the symmetrizing invertible transformation

7.43 $$\begin{aligned} \begin{aligned} \mathcal {M}_{\perp } := \frac{\Pi ^{\perp }}{\sqrt{2}} \begin{pmatrix} \mathcal {L}_{{\mathfrak {m}},\perp }^{- 1/2} &  \mathcal {L}_{{\mathfrak {m}},\perp }^{- 1/2} \\ -\textrm{Id} &  \quad \textrm{Id} \end{pmatrix}\Pi ^{\perp }, \quad \mathcal {M}_{\perp }^{- 1} := \frac{\Pi ^{\perp }}{\sqrt{2}} \begin{pmatrix} \mathcal {L}_{{\mathfrak {m}},\perp }^{1/2}&  \quad -\textrm{Id} \\ \mathcal {L}_{{\mathfrak {m}},\perp }^{1/2} &  \quad \textrm{Id} \end{pmatrix}\Pi ^{\perp }. \end{aligned}\qquad \quad \end{aligned}$$

where, by the standard functional calculus on the self-adjoint operator $\mathcal {L}_{{\mathfrak {m}}}$ in Proposition 3.10, for any $\alpha \in {{\mathbb {R}}}\setminus \{0\}$ we defined

7.44 $$\begin{aligned} f(y) = \sum _{j=1}^{\infty } f_j \phi _{j,{\mathfrak {m}}}(y) \ \mapsto \ \mathcal {L}_{{\mathfrak {m}},\perp }^\alpha f(y) := \mathcal {L}_{{\mathfrak {m}}}^\alpha \Pi ^\perp f(y) = \sum _{j\geqslant \kappa _{0}+1} f_j \lambda _{j,{\mathfrak {m}}}^{2\alpha } \phi _{j,{\mathfrak {m}}}(y).\nonumber \\ \end{aligned}$$

It is immediate to verify that $\big ( \mathcal {L}_{{\texttt{m}}} f , f \big )_{L^2} = \sum _{j \geqslant \kappa _0 + 1} \lambda _{j, {\texttt{m}}}^2 |f_j|^2 \geqslant \lambda _{\kappa _0 + 1}^2 \Vert f \Vert _{L^2}^2$ and

$$\begin{aligned} \begin{aligned} \big ( \mathcal {L}_{{\texttt{m}}} f , f \big )_{L^2}&\lesssim \Vert \partial _y f \Vert _{L^2}^2 + \Vert Q_{{\texttt{m}}} \Vert _{L^\infty } \Vert f \Vert _{L^2}^2 \lesssim \Vert \partial _y f \Vert _{L^2}^2 + \Vert f \Vert _{L^2}^2, \\ \Vert \partial _y f \Vert _{L^2}^2&\lesssim \big ( \mathcal {L}_{{\texttt{m}}} f , f \big )_{L^2} + |\big ( Q_{{\texttt{m}}} f , f \big )_{L^2}| \lesssim \big ( \mathcal {L}_{{\texttt{m}}} f , f \big )_{L^2} + \Vert f \Vert _{L^2}^2 \lesssim \big ( \mathcal {L}_{{\texttt{m}}} f , f \big )_{L^2}. \end{aligned} \end{aligned}$$

This implies that, for any function $f(y) = \sum _{j \geqslant \kappa _0 + 1} f_j \phi _{j, {\texttt{m}}}(y) \in H_0^1([- 1, 1])$,

7.45 $$\begin{aligned} \begin{aligned}&\Vert f \Vert _{H^1}^2 \simeq \big ( \mathcal {L}_{{\texttt{m}}, \bot } f, f \big )_{L^2} = \sum _{j \geqslant \kappa _0 + 1} \lambda _j^2 |f_j|^2 . \end{aligned} \end{aligned}$$

By the latter inequality, we easily deduce that, for any $f \in H^{s, 0}_\bot$ and $0 \leqslant \rho , \alpha \leqslant 1$,

7.46 $$\begin{aligned} \Vert \mathcal {L}_{{\texttt{m}}, \bot }^{- 1} f \Vert _{s, \rho } \lesssim \Vert f \Vert _{s, 0}, \quad \Vert \mathcal {L}_{{\texttt{m}}, \bot }^\alpha f \Vert _{s, 0} \lesssim \Vert f \Vert _{s, 1},, \quad \Vert \mathcal {L}_{{\texttt{m}}, \bot }^{- \alpha } f \Vert _{s, \rho } \lesssim \Vert f \Vert _{s, \rho }. \nonumber \\ \end{aligned}$$

By (7.42) and (7.43), we have

7.47 $$\begin{aligned} \textbf{G}_{\omega }&:= \mathcal {M}_{\perp }^{-1} \mathcal {G}_{\omega } \mathcal {M}_{\perp } = \textbf{D}_{\omega } + \textbf{R}_{\varepsilon } , \nonumber \\ \textbf{D}_{\omega }&:= \Pi ^{\perp }\begin{pmatrix} \omega \cdot \partial _{\textbf{x}} + \mathcal {L}_{{\mathfrak {m}},\perp }^{1/2} & 0 \\ 0 &  \omega \cdot \partial _{\textbf{x}} -\mathcal {L}_{{\mathfrak {m}},\perp }^{1/2} \end{pmatrix} \Pi ^{\perp }, \quad \textbf{R}_{\varepsilon } := \mathcal {M}_{\perp }^{-1} \mathcal {R}_{\varepsilon } \mathcal {M}_{\perp }.\nonumber \\ \end{aligned}$$

We split the rest of the proof in several steps.

Step 1) Analysis of $\textbf{R}_\varepsilon$. We split the remainder $\textbf{R}_\varepsilon$ in (7.47) as

$$\begin{aligned} \begin{aligned}&\textbf{R}_\varepsilon = \textbf{R}_1 + \textbf{R}_2 , \\&\textbf{R}_1 := - \mathcal {M}_\bot ^{- 1}\Pi _\bot \begin{pmatrix} 0 &  \quad 0 \\ a &  \quad 0 \end{pmatrix} \Pi _\bot \mathcal {M}_\bot , \quad \textbf{R}_2 = - \sqrt{\varepsilon } \mathcal {M}_\bot ^{- 1} J R \mathcal {M}_\bot . \end{aligned} \end{aligned}$$

By a direct calculation and using that *JR* is a finite rank operator (see Lemma 7.7), the operators $\textbf{R}_1, \textbf{R}_2$ have the following form:

7.48 $$\begin{aligned} \begin{aligned} \textbf{R}_1&= \frac{\Pi _\bot }{2} \begin{pmatrix} a \mathcal {L}_{{\texttt{m}},\perp }^{- 1/2} &  a \mathcal {L}_{{\texttt{m}},\perp }^{- 1/2} \\ - a \mathcal {L}_{{\texttt{m}},\perp }^{- 1/2} &  - a \mathcal {L}_{{\texttt{m}},\perp }^{- 1/2} \end{pmatrix} \Pi _\bot , \\ \textbf{R}_2[u]&=- \sqrt{\varepsilon }\sum _{j \in S} \big ( p_j , u \big )_{L^2_x} q_j, \quad p_j := \mathcal {M}_\bot ^\top [g_j], \quad q_j := \mathcal {M}_\bot ^{- 1}[\chi _j] . \end{aligned} \end{aligned}$$

The latter formula, together with the estimate (7.41), the tame estimate (2.5), the properties (7.46) and the ansatz (7.1) implies that $\textbf{R}_1$ satisfies the estimate

7.49 $$\begin{aligned} \Vert \textbf{R}_1 u\Vert _{s, 0}^{k_0, \upsilon } \lesssim _s \sqrt{\varepsilon }\big ( \Vert u \Vert _{s, 0}^{k_0, \upsilon } + \Vert {{\mathfrak {I}}}_0 \Vert _{\mathcal {X}^s}^{k_0, \upsilon } \Vert u \Vert _{s_0, 0}^{k_0, \upsilon } \big ), \quad \forall \, s_0 \leqslant s \leqslant S/2 - \sigma . \qquad \end{aligned}$$

We now estimate $\textbf{R}_2.$ Since $g_j = (g_j^{(1)}, g_j^{(2)})$, $\chi _{j} = (\chi _{j}^{(1)}, \chi _{j}^{(2)}) \in \mathcal {Y}^s_\bot$, by (7.39), we have, for any $s_0 \leqslant s \leqslant S/2 - \sigma$,

$$\begin{aligned} \Vert g_j^{(1)} \Vert _{s, 1}^{k_0, \upsilon }, \, \Vert \chi _{j}^{(1)} \Vert _{s, 1}^{k_0, \upsilon }, \,\Vert g_j^{(2)} \Vert _{s, 1}^{k_0, \upsilon }, \, \Vert \chi _{j}^{(2)} \Vert _{s, 1}^{k_0, \upsilon } \lesssim _s 1 + \Vert {{\mathfrak {I}}}_0 \Vert _{s + \sigma }^{k_0, \upsilon }. \end{aligned}$$

Moreover, since we have, by (7.49), (7.43) that

$$\begin{aligned} p_j = \frac{1}{\sqrt{2}}\begin{pmatrix} \mathcal {L}_{{\texttt{m}},\perp }^{- 1/2} g_j^{(1)} - g_j^{(2)}, \\ \mathcal {L}_{{\texttt{m}},\perp }^{- 1/2} g_j^{(1)} + g_j^{(2)} \end{pmatrix}, \quad q_j = \frac{1}{\sqrt{2}}\begin{pmatrix} \mathcal {L}_{{\texttt{m}},\perp }^{1/2} \chi _j^{(1)} - \chi _j^{(2)}, \\ \mathcal {L}_{{\texttt{m}},\perp }^{1/2} \chi _j^{(1)} + \chi _j^{(2)} \end{pmatrix}, \end{aligned}$$

we obtain, together with the estimates (7.46), that

$$\begin{aligned} \Vert p_j \Vert _{\mathcal {Y}^s_\bot }^{k_0, \upsilon },\, \Vert q_j \Vert _{s, 0}^{k_0, \upsilon } \lesssim _s 1 + \Vert {{\mathfrak {I}}}_0 \Vert _{\mathcal {X}^{s+\sigma }}^{k_0, \upsilon }, \quad \forall \, s_0 \leqslant s \leqslant S/2 - \sigma . \end{aligned}$$

These latter estimates, together with the formula of $\textbf{R}_2$ in (7.48) and Lemma 2.1-(*ii*) to estimates the terms $(p_j, u)_{L^2} q_j$, imply that $\textbf{R}_2$ satisfies the same estimate as $\textbf{R}_1$ in (7.49). We conclude that

7.50 $$\begin{aligned} \Vert \textbf{R}_\varepsilon u\Vert _{s, 0}^{k_0, \upsilon } \lesssim _s \sqrt{\varepsilon }\big ( \Vert u \Vert _{s, 0}^{k_0, \upsilon } + \Vert {{\mathfrak {I}}}_0 \Vert _{\mathcal {X}^{s + \sigma }}^{k_0, \upsilon } \Vert u \Vert _{s_0, 0}^{k_0, \upsilon } \big ), \quad \forall \,s_0 \leqslant s \leqslant S/2 - \sigma . \nonumber \\ \end{aligned}$$

Step 2) Inversion of $\textbf{D}_\omega$. The operators $\Pi ^\perp \big (\omega \cdot \partial _{\textbf{x}}\pm \mathcal {L}_{{\mathfrak {m}},\perp }^{1/2}\big ) \Pi ^\perp$ are invertible on their range, with bounded and smoothing inverse from $H_{\bot }^{s,\rho }$ to $H_{\bot }^{s,\rho + 1}$ for any $\rho \geqslant 0$, where the spaces are defined in (4.46). Indeed, recalling Proposition 3.10 and (7.44), the operators $\big (\omega \cdot \partial _\textbf{x}\pm \mathcal {L}_{{\mathfrak {m}},\perp }^{1/2}\big )^{-1}$ act on elements of the basis of the form $e^{\textrm{i}\ell \cdot \textbf{x}}\phi _{j,{\mathfrak {m}}}(y)$, with $j\geqslant \kappa _0+1$ and $\ell \in {{\mathbb {Z}}}^{\kappa _0}$, as

$$\begin{aligned} \big (\omega \cdot \partial _\textbf{x}\pm \mathcal {L}_{{\mathfrak {m}},\perp }^{1/2}\big )^{-1} e^{\textrm{i}\ell \cdot \textbf{x}}\phi _{j,{\mathfrak {m}}}(y) = \frac{1}{\textrm{i}\,\omega \cdot \ell \pm \lambda _{j,{\mathfrak {m}}}} e^{\textrm{i}\ell \cdot \textbf{x}}\phi _{j,{\mathfrak {m}}}(y). \end{aligned}$$

By Proposition 3.10, It is clear that $|\textrm{i}\,\omega \cdot \ell \pm \lambda _{j,{\mathfrak {m}}}|> \lambda _{j,{\mathfrak {m}}}>0$ for any $j\geqslant \kappa _0+1$ and $\ell \in {{\mathbb {Z}}}^{\kappa _0}$. Therefore, by recalling (7.45), $\textbf{D}_{\omega }$ is invertible and it satisfies the following bounds, for any $u = (u_1, u_2) \in H^{s, \rho }_\bot \times H^{s, \rho }_\bot$ and any $s, \rho \geqslant 0$,

7.51 $$\begin{aligned} \begin{aligned}&\Vert \textbf{D}_\omega ^{- 1} u \Vert _{s, 0}^{k_0,\upsilon } \lesssim \Vert \textbf{D}_\omega ^{- 1} u \Vert _{s, 1}^{k_0,\upsilon } \lesssim _s \Vert u \Vert _{s, 0}^{k_0,\upsilon }. \end{aligned} \end{aligned}$$

Step 3) Inversion of $\textbf{G}_\omega$. We write $\textbf{G}_{\omega }$ in (7.47) as

$$\begin{aligned} \textbf{G}_{\omega } = \big ( \textrm{Id} + \textbf{R}_{\varepsilon }\textbf{D}_{\omega }^{-1} \big ) \textbf{D}_{\omega }. \end{aligned}$$

The estimates in (7.50) and (7.51) imply that

$$\begin{aligned} \big \Vert \textbf{R}_{\varepsilon } \textbf{D}_{\omega }^{-1} u \big \Vert _{s, 0}^{k_0, \upsilon } \lesssim _s \sqrt{\varepsilon } \big ( \Vert u \Vert _{s, 0}^{k_0, \upsilon } + \Vert {{\mathfrak {I}}}_0 \Vert _{\mathcal {X}^{s + \sigma }}^{k_0, \upsilon } \Vert u \Vert _{s_0, 0}^{k_0, \upsilon } \big ), \quad \forall s_0 \leqslant s \leqslant S/2 - \sigma . \end{aligned}$$

Thus, by the ansatz (7.1), for $\varepsilon \ll 1$ small enough, the operator $\textbf{G}_\omega = \big (\textrm{Id} + \textbf{R}_{\varepsilon } \textbf{D}_{\omega }^{-1} \big ) \textbf{D}_\omega$ is invertible as an operator from $\mathcal {Y}^s_\bot = H^{s, 1}_\bot \times H^{s, 1}_\bot \rightarrow H^{s, 0}_\bot \times H^{s, 0}_\bot$ and its inverse satisfies the estimate, for all $s_0 \leqslant s \leqslant S/2 - \sigma$,

7.52 $$\begin{aligned} \big \Vert \textbf{G}_\omega ^{- 1} u \big \Vert _{\mathcal {Y}^s_\bot }^{k_0, \upsilon } \lesssim _s \big ( \Vert u_1 \Vert _{s, 0}^{k_0, \upsilon } + \Vert u_2 \Vert _{s, 0}^{k_0, \upsilon } \big ) + \Vert {{\mathfrak {I}}}_0\Vert _{\mathcal {X}^{s + \sigma }}^{k_0, \upsilon } \big ( \Vert u_1 \Vert _{s_0, 0}^{k_0, \upsilon } + \Vert u_2 \Vert _{s_0, 0}^{k_0, \upsilon } \big ). \nonumber \\ \end{aligned}$$

Step 4) Inversion of $\mathcal {G}_\omega$ on  $\mathcal {Y}^s_\bot$. In order to estimate $\mathcal {G}_\omega ^{- 1}$, we observe that, by (7.47), one has $\mathcal {G}_\omega ^{- 1} = \mathcal {M}_\bot \textbf{G}_\omega ^{- 1} \mathcal {M}_\bot ^{- 1}$. By the expressions of $\mathcal {M}_\bot , \mathcal {M}_\bot ^{- 1}$ in (7.43) and the estimates in (7.46), one easily deduces that

$$\begin{aligned} \Vert \mathcal {M}_\bot u \Vert _{\mathcal {Y}^s_\bot }^{k_0,\upsilon } \lesssim \Vert u \Vert _{\mathcal {Y}^s_\bot }^{k_0,\upsilon } \quad \text {and} \quad \Vert (\mathcal {M}_\bot ^{- 1} u)_1 \Vert _{s, 0}^{k_0,\upsilon } +\Vert (\mathcal {M}_\bot ^{- 1} u )_2\Vert _{s, 0}^{k_0,\upsilon } \lesssim \Vert u \Vert _{\mathcal {Y}^s_\bot }^{k_0,\upsilon }. \end{aligned}$$

Therefore, by this latter estimate, together with (7.52) we deduce that, for any $s_0 \leqslant s \leqslant S/2 - \sigma$, given $f = (f_1, f_2) \in \mathcal {Y}^s_\bot$, there exists a solution $h = (h_1, h_2) := \mathcal {G}_\omega ^{- 1} f\in \mathcal {Y}^s_\bot$ of the equation $\mathcal {G}_\omega h = f$ satisfying the tame estimates

7.53 $$\begin{aligned} \Vert h \Vert _{\mathcal {Y}^s_\bot }^{k_0, \upsilon } = \Vert \mathcal {G}_\omega ^{- 1} f \Vert _{\mathcal {Y}^s_\bot }^{k_0, \upsilon } \lesssim _s \Vert f \Vert _{\mathcal {Y}^s_\bot }^{k_0, \upsilon } + \Vert {{\mathfrak {I}}}_0 \Vert _{\mathcal {X}^{s + \sigma }}^{k_0, \upsilon } \Vert f \Vert ^{k_0, \upsilon }_{\mathcal {Y}^{s_0}_\bot }, \quad \forall s_0 \leqslant s \leqslant S/2 - \sigma . \nonumber \\ \end{aligned}$$

Moreover, if *f* is anti-reversible, then *h* is reversible.

Step 5) Estimate of $\mathcal {G}_\omega ^{- 1}$ on  $\mathcal {X}^s_\bot$. It remains to show that $h = (h_1, h_2) \in \mathcal {X}^s_\bot$, for any $s_0 \leqslant s \leqslant S/2 - \sigma$, namely $h_1 \in H^{s, 3}_\bot$ for any $s_0 \leqslant s \leqslant S/2 - \sigma$. This follows essentially by an elliptic regularity argument. Indeed, using that $\mathcal {L}_{{\texttt{m}},\perp } = \Pi _\bot (- \partial _y^2 + Q_{{\texttt{m}}}(y)) \Pi _\bot$, by (7.42), (7.44), the vector $h = (h_1, h_2)$ solves the system

7.54 $$\begin{aligned} {\left\{ \begin{array}{ll} \omega \cdot \partial _\textbf{x} h_1 - h_2 =f_1 - \mathcal {R}^{(1)}_\varepsilon [h_1, h_2], \\ \omega \cdot \partial _\textbf{x} h_2 + \partial _{y}^2 h_1 - \Pi _\bot Q_{{\texttt{m}}}(y) h_1 = f_2 - \mathcal {R}^{(2)}_\varepsilon [h_1, h_2], \end{array}\right. } \end{aligned}$$

where we write the remainder $\mathcal {R}_\varepsilon$ as

$$\begin{aligned} \mathcal {R}_\varepsilon [h_1, h_2] = \begin{pmatrix} \mathcal {R}^{(1)}_\varepsilon [h_1, h_2] \\ \mathcal {R}^{(2)}_\varepsilon [h_1, h_2] \end{pmatrix} . \end{aligned}$$

By Lemma 7.7-(*ii*), the estimates (7.41), (2.4) (use also the ansatz (7.1)), (7.53) and the form of $\mathcal {R}_\varepsilon$ in (7.42), one deduces, for any $s_0 \leqslant s \leqslant S/2 - \sigma$,

7.55 $$\begin{aligned} \begin{aligned} \Vert \mathcal {R}^{(2)}_\varepsilon [h_1, h_2] \Vert _{s, 1}^{k_0, \upsilon }&\leqslant \Vert \mathcal {R}_\varepsilon h \Vert _{\mathcal {Y}^s_\bot }^{k_0, \upsilon } \lesssim _s \sqrt{\varepsilon } \big ( \Vert h \Vert _{\mathcal {Y}^s_\bot }^{k_0, \upsilon } + \Vert {{\mathfrak {I}}}_0 \Vert _{\mathcal {X}^{s + \sigma }}^{k_0, \upsilon } \Vert h \Vert _{\mathcal {Y}^{s_0}_\bot }^{k_0, \upsilon } \big ) \\&\lesssim _s \sqrt{\varepsilon } \big ( \Vert f \Vert _{\mathcal {Y}^s_\bot }^{k_0, \upsilon } + \Vert {{\mathfrak {I}}}_0 \Vert _{\mathcal {X}^{s + \sigma }}^{k_0, \upsilon } \Vert f \Vert _{\mathcal {Y}^{s_0}_\bot }^{k_0, \upsilon } \big ) \\ \Vert Q_{{\texttt{m}}} h_1 \Vert _{s, 1}^{k_0, \upsilon }&\lesssim \Vert h_1 \Vert _{s, 1}^{k_0, \upsilon } \lesssim _s \big ( \Vert f \Vert _{\mathcal {Y}^s_\bot }^{k_0, \upsilon } + \Vert {{\mathfrak {I}}}_0 \Vert _{\mathcal {X}^{s + \sigma }}^{k_0, \upsilon } \Vert f \Vert _{\mathcal {Y}^{s_0}_\bot }^{k_0, \upsilon } \big ) \end{aligned} \end{aligned}$$

where in the second estimate we have used that $Q_{{\texttt{m}}}(y)$ is a smooth potential, with $\Vert Q_{{\texttt{m}}} \Vert _{H^1} \leqslant C({\texttt{E}}_1 , {\texttt{E}}_2)$ for some constant $C({\texttt{E}}_1 , {\texttt{E}}_2) >0$. Then, by the second equation in (7.54), since $-\partial _{y}^2 : H^3_0([- 1, 1]) \rightarrow H_0^1([- 1, 1])$ is invertible and its inverse $(- \partial _{y}^2)^{- 1}$ gains two derivatives with respect to *y*, namely $\Vert (- \partial _{y}^2)^{- 1} u \Vert _{s, 3}^{k_0, \upsilon } \lesssim \Vert u \Vert _{s, 1}^{k_0, \upsilon }$ for any $s \geqslant 0$, $u \in H^{s, 1}_\bot$, one has

$$\begin{aligned} h_1 = ( -\partial _{y}^2)^{- 1}\big [ \omega \cdot \partial _\textbf{x} h_2 - \Pi _\bot Q_{{\texttt{m}}}(y) h_1 - f_2 + \mathcal {R}^{(2)}_\varepsilon [h_1, h_2] \big ]. \end{aligned}$$

Therefore, by (7.53), (7.55) and for $\varepsilon >0$ small enough, we have, for any $s_0 \leqslant s \leqslant S/2 - \sigma$ with an eventually larger $\sigma$,

$$\begin{aligned} \begin{aligned} \Vert h_1 \Vert _{s, 3}^{k_0, \upsilon }&\leqslant \big \Vert \omega \cdot \partial _\textbf{x} h_2 - \Pi _\bot Q_{{\texttt{m}}}(y) h_1 - f_2 + \mathcal {R}^{(2)}_\varepsilon [h_1, h_2] \big \Vert _{s, 1}^{k_0, \upsilon } \\&\leqslant \Vert h_2 \Vert _{s + 1, 1}^{k_0, \upsilon } + \Vert f_2 \Vert _{s, 1}^{k_0, \upsilon } + \Vert Q_{{\texttt{m}}}(y) h_1 \Vert _{s, 1}^{k_0, \upsilon } + \Vert \mathcal {R}^{(2)}_\varepsilon [h_1, h_2] \Vert _{s, 1}^{k_0, \upsilon } \\&\lesssim _s \Vert f \Vert _{\mathcal {Y}^{s + 1}_\bot }^{k_0, \upsilon } + \Vert {{\mathfrak {I}}}_0 \Vert _{\mathcal {X}^{s + \sigma }}^{k_0, \upsilon } \Vert f \Vert ^{k_0, \upsilon }_{\mathcal {Y}^{s_0}_\bot }. \end{aligned} \end{aligned}$$

This latter estimate, together with the estimate (7.53), implies the claimed bound of Lemma 7.4. The proof is then concluded. $\square$

## Proof of Theorem 4.5

Theorem 4.5 is a consequence of Theorem 8.2 below. Recalling (4.48), we define the spaces $\mathcal {X}^\infty$ and $\mathcal {Y}^\infty$ as $\mathcal {X}^\infty := \bigcap _{s \geqslant 0} \mathcal {X}^s$, $\mathcal {Y}^\infty := \bigcap _{s \geqslant 0} \mathcal {Y}^s$, and, for any $\texttt{n}\in {{\mathbb {N}}}_0$, we define the superexponential scale

8.1 $$\begin{aligned} K_\texttt{n}: = K_0^{\chi ^\texttt{n}} , \quad \chi = 3/2\, . \end{aligned}$$

We consider the finite dimensional subspaces

$$\begin{aligned} E_\texttt{n}:= \big \{ {\mathfrak {I}}(\textbf{x})= (\Theta ,I,z)(\textbf{x}) \in \mathcal {X}^\infty \ : \ \Theta = \Pi _\texttt{n}\Theta , \ I=\Pi _\texttt{n}I , \ z = \Pi _\texttt{n}z \big \} \end{aligned}$$

where $\Pi _\texttt{n}z := \Pi _{K_\texttt{n}} z$ is defined as in (2.8) with $K_n$ in (8.1), and we denote with the same symbol $\Pi _\texttt{n}g(\textbf{x}) := \sum _{\left| \ell \right| \leqslant K_\texttt{n}} g_\ell e^{\textrm{i}\ell \cdot \textbf{x}}$. Note that the projector $\Pi _{\texttt{n}}$ maps (anti)-reversible variations into (anti)-reversible variations. We introduce some constants needed to implement the Nash–Moser iteration. Let ${\overline{\sigma }}\equiv {\overline{\sigma }}(\tau , k_0) \gg 0$ be the largest loss of derivatives coming from the construction of the approximate inverse of the linearized operator in Theorem 7.6 and let $S \gg s_0$ be the smoothness of our nonlinearity. Then, we define the following parameters

8.2 $$\begin{aligned} \begin{aligned}&{\overline{\mu }} := 3 {\overline{\sigma }} + 1, \quad \mu _1 := 3({\overline{\mu }} + 1) + 1, \quad \texttt{a}_1:= 3({\overline{\mu }} + 1) + 1 , \\&{\texttt{b}}_1 := \texttt{a}_1 + {\overline{\mu }} + \tfrac{2}{3} \mu _1 + 3, \quad \texttt{a}_2 := \texttt{a}_1 - 3 {\overline{\sigma }}, \quad S := 2(s_0 + {\texttt{b}}_1 + {\overline{\sigma }}). \end{aligned} \end{aligned}$$

### Remark 8.1

The constant $\texttt{a}_1$ is the exponent in (8.6). The constant $\texttt{a}_2$ is the exponent in (8.5). The constant $\texttt{b}_1$ is the largest increase of derivatives we need to control with respect to the low regularity $s_0$ and the constant $\mu _1$ is the exponent in the control of the high norm in (8.7).

### Theorem 8.2

(Nash–Moser) There exist $\delta _0, C_*>0$ such that, if

8.3 $$\begin{aligned} \begin{aligned}&K_0^{{\overline{\mu }} + \texttt{a}_1} \sqrt{\varepsilon }\upsilon ^{-1}< \delta _0 , \quad K_0 := \upsilon ^{-1}, \quad \\&\upsilon := \varepsilon ^{\textrm{a}}, \quad 0< \textrm{a} < \textrm{a}_0 := (2(1 + {\overline{\mu }} + \texttt{a}_1))^{-1}, \end{aligned} \end{aligned}$$

then, for all $\texttt{n}\geqslant 0$:

$(\mathcal {P}1)_\texttt{n}$ There exists a $k_0$-times differentiable function ${\widetilde{W}}_\texttt{n}:{{\mathbb {R}}}^{\kappa _0}\times \mathcal {J}_{\varepsilon }(\texttt{E}) \rightarrow E_{\texttt{n}-1}\times {{\mathbb {R}}}^{\kappa _0}$, $\lambda =(\omega ,\texttt{A})\mapsto {\widetilde{W}}_\texttt{n}(\lambda ):= ({\widetilde{{\mathfrak {I}}}}_\texttt{n}, {\widetilde{\alpha }}_\texttt{n}-\omega )$, for $\texttt{n}\geqslant 1$, and ${\widetilde{W}}_0:=0$, satisfying

8.4 $$\begin{aligned} \Vert {\widetilde{W}}_\texttt{n} \Vert _{\mathcal {X}^{s_0+{\overline{\sigma }}}}^{k_0,\upsilon } \leqslant C_* \sqrt{\varepsilon }\upsilon ^{-1} . \end{aligned}$$

Let ${\widetilde{U}}_\texttt{n}:= U_0+{\widetilde{W}}_\texttt{n}$, where $U_0:= (\textbf{x},0,0,\omega )$. The difference ${\widetilde{H}}_\texttt{n}:= {\widetilde{U}}_\texttt{n}-{\widetilde{U}}_{\texttt{n}-1}$, for $\texttt{n}\geqslant 1$, satisfies

8.5 $$\begin{aligned} \begin{aligned}&\Vert {\widetilde{H}}_1 \Vert _{\mathcal {X}^{s_0+{\overline{\sigma }}}}^{k_0,\upsilon }\leqslant C_* \sqrt{\varepsilon } \upsilon ^{-1}, \quad \Vert {\widetilde{H}}_\texttt{n} \Vert _{\mathcal {X}^{s_0+{\overline{\sigma }}}}^{k_0,\upsilon } \leqslant C_* \sqrt{\varepsilon } \upsilon ^{-1} K_{\texttt{n}-1}^{-\texttt{a}_2}, \ \forall \, \texttt{n}\geqslant 2 . \end{aligned} \end{aligned}$$

The torus embedding ${\widetilde{i}}_\texttt{n}:= (\textbf{x},0,0) + {\widetilde{{\mathfrak {I}}}}_\texttt{n}$ is reversible, that is (4.45) holds.

$(\mathcal {P}2)_\texttt{n}$ For all $\omega \in \texttt{G}^{\upsilon }$ (see (4.54)), setting $K_{-1}:=1$, we have

8.6 $$\begin{aligned} \Vert \mathcal {F}({\widetilde{U}}_\texttt{n}) \Vert _{\mathcal {Y}^{s_0}}^{k_0,\upsilon } \leqslant C_* \sqrt{\varepsilon } K_{\texttt{n}-1}^{-\texttt{a}_1} . \end{aligned}$$

$(\mathcal {P}3)_\texttt{n}$ (High norms) For any $\lambda =(\omega ,\texttt{A}) \in {{\mathbb {R}}}^{\kappa _{0}}\times \mathcal {J}_{\varepsilon }(\texttt{E})$, we have

8.7 $$\begin{aligned} \Vert {\widetilde{W}}_\texttt{n} \Vert _{\mathcal {X}^{s_0+\texttt{b}_1}}^{k_0,\upsilon } \leqslant C_* \sqrt{\varepsilon } \upsilon ^{-1} K_{\texttt{n}-1}^{\mu _1}. \end{aligned}$$

### Proof

We argue by induction.

STEP 1: Proof of $(\mathcal {P}1,2,3)_0$. By (4.43), Lemma 7.1 and Proposition 4.1, we deduce

8.8 $$\begin{aligned} \Vert \mathcal {F}(U_0) \Vert _{\mathcal {Y}^{s}}^{k_0,\upsilon } =O( \sqrt{\varepsilon }). \end{aligned}$$

The claims then follow by (8.8), taking $C_*$ large enough and by noting that $i_0:=(\textbf{x},0,0)$ is clearly reversible.

STEP 2: Assume $(\mathcal {P}1,2,3)_\texttt{n}$ for some $\texttt{n}\in {{\mathbb {N}}}_0$ and prove $(\mathcal {P}1,2,3)_{\texttt{n}+1}$. We are going to define the successive approximation ${\widetilde{U}}_{\texttt{n}+1}$ by a modified Nash–Moser scheme and prove by induction that the approximate torus ${\widetilde{i}}_{\texttt{n}+1}$ is reversible. For that, we prove the almost-approximate invertibility of the linearized operator $G_\texttt{n}= G_\texttt{n}(\lambda ):= \textrm{d}_{i,\alpha } \mathcal {F}({\widetilde{i}}_\texttt{n}(\lambda ))$. We apply Theorem 7.6 to $G_\texttt{n}(\lambda )$. It implies, for $\lambda =(\omega ,\texttt{A})\in \texttt{G}^{\upsilon }\times \mathcal {J}_{\varepsilon }(\texttt{E})$, the existence of an almost-approximate inverse $\textbf{T}_\texttt{n}:= \textbf{T}_\texttt{n}(\lambda ,{\widetilde{i}}_\texttt{n}(\lambda ))$ of the linearized operator $G_{\texttt{n}}=\textrm{d}_{i, \alpha }\mathcal {F}({\widetilde{i}}_\texttt{n})$ which satisfies, for any anti-reversible variation *g* and for any $s_0\leqslant s \leqslant s_0+\texttt{b}_1$,

8.9 $$\begin{aligned}&\Vert \textbf{T}_\texttt{n}g \Vert _{\mathcal {X}^s}^{k_0,\upsilon } \lesssim _{s_0+\texttt{b}_1} \upsilon ^{-1} (\Vert g \Vert _{\mathcal {Y}^{s+{\overline{\sigma }}}}^{k_0,\upsilon }+\Vert {\widetilde{{\mathfrak {I}}}}_\texttt{n} \Vert _{\mathcal {X}^{s+{\overline{\sigma }}}}^{k_0,\upsilon } \Vert g \Vert _{\mathcal {Y}^{s_0+{\overline{\sigma }}}}^{k_0,\upsilon }), \end{aligned}$$

8.10 $$\begin{aligned}&\Vert \textbf{T}_\texttt{n}g \Vert _{\mathcal {X}^{s_0}}^{k_0,\upsilon } \lesssim _{s_0+\texttt{b}_1} \upsilon ^{-1} \Vert g \Vert _{\mathcal {Y}^{s_0+{\overline{\sigma }}}}^{k_0,\upsilon } . \end{aligned}$$

Moreover, the first three components of $\textbf{T}_\texttt{n}g$ form a reversible variation. For all $\lambda \in \texttt{G}^{\upsilon }\times \mathcal {J}_{\varepsilon }(\texttt{E})$ we define the successive approximation

8.11 $$\begin{aligned} \begin{aligned}&U_{\texttt{n}+1} := {\widetilde{U}}_\texttt{n}+ H_{\texttt{n}+1} , \\&H_{\texttt{n}+1} := ({\widehat{{\mathfrak {I}}}}_{\texttt{n}+1}, {\widehat{\alpha }}_{\texttt{n}+1}) := -\mathbf{{\Pi }}_\texttt{n}\textbf{T}_\texttt{n}\Pi _n \mathcal {F}({\widetilde{U}}_\texttt{n}) \in E_\texttt{n}\times {{\mathbb {R}}}^{\kappa _0} , \end{aligned} \end{aligned}$$

where $\mathbf{{\Pi }}_\texttt{n}$ is defined for any $({\mathfrak {I}},\alpha )$ by

8.12 $$\begin{aligned} \mathbf{{\Pi }}_\texttt{n}({\mathfrak {I}},\alpha ) := ( \Pi _\texttt{n}{\mathfrak {I}},\alpha ) , \quad \mathbf{{\Pi }}_\texttt{n}^\perp := (\Pi _\texttt{n}^\perp {\mathfrak {I}},0). \end{aligned}$$

Since ${\widetilde{i}}_{\texttt{n}}$ is reversible by induction assumption, we have that $\mathcal {F}({\widetilde{U}}_\texttt{n}) = \mathcal {F}({\widetilde{i}}_\texttt{n}, {\widetilde{\alpha }}_n)$ is anti-reversible, that is (7.16) holds. Thus, the first three components of $\textbf{T}_\texttt{n}\mathcal {F}({\widetilde{U}}_\texttt{n})$ form a reversible variation, as well as $\mathbf{{\Pi }}_\texttt{n}\textbf{T}_\texttt{n}\Pi _\texttt{n}\mathcal {F}({\widetilde{U}}_\texttt{n})$. We now show that the iterative scheme in (8.11) is rapidly converging. We write

$$\begin{aligned} \mathcal {F}(U_{\texttt{n}+1}) = \mathcal {F}({\widetilde{U}}_\texttt{n}) + G_\texttt{n}H_{\texttt{n}+1} + Q_{\texttt{n}} , \end{aligned}$$

where $$G_\texttt{n}:= \textrm{d}_{i,\alpha } \mathcal {F}({\widetilde{i}}_\texttt{n})$$ and

8.13 $$\begin{aligned} \begin{aligned}&Q_\texttt{n}:= Q({\widetilde{U}}_\texttt{n}, H_{\texttt{n}+1}) := \mathcal {F}({\widetilde{U}}_\texttt{n}+H_{\texttt{n}+1}) - \mathcal {F}({\widetilde{U}}_\texttt{n}) - G_\texttt{n}H_{\texttt{n}+1} . \end{aligned} \end{aligned}$$

Then, by the definition of $H_{\texttt{n}+1}$ in (8.11), we have

8.14 $$\begin{aligned} \begin{aligned} \mathcal {F}(U_{\texttt{n}+1})&= \mathcal {F}({\widetilde{U}}_\texttt{n}) - G_{\texttt{n}} \mathbf{{\Pi }}_\texttt{n}\textbf{T}_\texttt{n}\Pi _\texttt{n}\mathcal {F}({\widetilde{U}}_\texttt{n}) + Q_{\texttt{n}} \\&= ( \textrm{Id} - G_{\texttt{n}} \textbf{T}_\texttt{n})\mathcal {F}({\widetilde{U}}_\texttt{n}) + G_{\texttt{n}} \mathbf{{\Pi }}_\texttt{n}^\perp \textbf{T}_\texttt{n}\mathcal {F}({\widetilde{U}}_\texttt{n}) + Q_{\texttt{n}} \\&=P_{\texttt{n}} + R_{\texttt{n}} + Q_{\texttt{n}}, \end{aligned} \end{aligned}$$

where $Q_{\texttt{n}}$ is as in (8.13) and, according also to Theorem 7.6,

8.15 $$\begin{aligned} \begin{aligned}&P_{\texttt{n}}:= (\textrm{Id}-G_{\texttt{n}}\textbf{T}_\texttt{n}) \Pi _n\mathcal {F}({\widetilde{U}}_\texttt{n})= -\mathcal {P}({\widetilde{i}}_{\texttt{n}}) \Pi _n \mathcal {F}({\widetilde{U}}_\texttt{n}) , \\&R_{\texttt{n}}:= G_{\texttt{n}}\mathbf{{\Pi }}_\texttt{n}^\perp \textbf{T}_\texttt{n}\Pi _n\mathcal {F}({\widetilde{U}}_\texttt{n}) + \Pi _\texttt{n}^\bot \mathcal {F}({\widetilde{U}}_\texttt{n}). \end{aligned} \end{aligned}$$

First, by (4.43) , (8.8), (8.4) and Lemma 7.1, we have, for any $\lambda \in {{\mathbb {R}}}^{\kappa _0}\times \mathcal {J}_{\varepsilon }(\texttt{E})$,

8.16 $$\begin{aligned} \Vert \mathcal {F}({\widetilde{U}}_\texttt{n}) \Vert _{\mathcal {Y}^s}^{k_0,\upsilon }&\leqslant \Vert \mathcal {F}(U_0) \Vert _{\mathcal {Y}^s}^{k_0,\upsilon } + \Vert \mathcal {F}({\widetilde{U}}_\texttt{n}) - \mathcal {F}(U_0)\Vert _{\mathcal {Y}^s}^{k_0,\upsilon } \lesssim _{s} \sqrt{\varepsilon } + \Vert {\widetilde{W}}_{\texttt{n}} \Vert _{\mathcal {X}^{s+{\overline{\sigma }}}}^{k_0,\upsilon } . \nonumber \\ \end{aligned}$$

The latter estimate, together with (8.3), (8.4), implies that

8.17 $$\begin{aligned} \upsilon ^{-1} \Vert \mathcal {F}({\widetilde{U}}_\texttt{n}) \Vert _{\mathcal {Y}^{s_0}}^{k_0,\upsilon } \leqslant 1. \end{aligned}$$

We start with the estimates for $H_{\texttt{n}+1}$ since we will need them for the other estimates. By (8.11), (8.12), (2.9), (8.9), (8.10), (8.16), (8.17), we have

8.18 $$\begin{aligned} \Vert H_{\texttt{n}+1} \Vert _{\mathcal {X}^{s_0 + \texttt{b}_1}}^{k_0,\upsilon }&\lesssim _{s_0+\texttt{b}_1} K_{\texttt{n}}^{2{\overline{\sigma }}} \Vert \textbf{T}_\texttt{n}\mathcal {F}({\widetilde{U}}_{\texttt{n}}) \Vert _{\mathcal {X}^{s_0+\texttt{b}_1-2{\overline{\sigma }}}} \nonumber \\&\lesssim _{s_0+\texttt{b}_1} \upsilon ^{-1} K_{\texttt{n}}^{2{\overline{\sigma }}} \big (\Vert \mathcal {F}({\widetilde{U}}_{\texttt{n}})\Vert _{\mathcal {Y}^{s_0+\texttt{b}_1-{\overline{\sigma }}}}^{k_0,\upsilon }+\Vert {\widetilde{{\mathfrak {I}}}}_\texttt{n}\Vert _{\mathcal {X}^{s_0+\texttt{b}_1-{\overline{\sigma }}}}^{k_0,\upsilon } \Vert \mathcal {F}({\widetilde{U}}_{\texttt{n}}) \Vert _{\mathcal {Y}^{s_0+{\overline{\sigma }}}}^{k_0,\upsilon }\big ) \nonumber \\&\lesssim _{s_0+\texttt{b}_1} \upsilon ^{-1} K_{\texttt{n}}^{2{\overline{\sigma }}}\big ( \sqrt{\varepsilon } + \Vert {\widetilde{W}}_{\texttt{n}} \Vert _{\mathcal {X}^{s_0+\texttt{b}_1}}^{k_0,\upsilon } \big ), \end{aligned}$$

8.19 $$\begin{aligned} \Vert H_{\texttt{n}+1} \Vert _{\mathcal {X}^{s_0}}^{k_0,\upsilon }&\lesssim _{s_0} \upsilon ^{-1} K_{\texttt{n}}^{{\overline{\sigma }}} \Vert \mathcal {F}({\widetilde{U}}_{\texttt{n}}) \Vert _{\mathcal {Y}^{s_0}}^{k_0,\upsilon }, \end{aligned}$$

8.20 $$\begin{aligned} \Vert H_{\texttt{n}+1} \Vert _{\mathcal {X}^{s_0 + {\overline{\sigma }}}}^{k_0,\upsilon }&\lesssim \upsilon ^{-1} K_{\texttt{n}}^{2 {\overline{\sigma }}} \Vert \mathcal {F}({\widetilde{U}}_{\texttt{n}}) \Vert _{\mathcal {Y}^{s_0}}^{k_0,\upsilon } . \end{aligned}$$

We estimate $P_{\texttt{n}}$, $Q_{\texttt{n}}$ and $R_{\texttt{n}}$ with respect to the Sobolev norms in the low regularity $s_0$. By the definition of $Q_{\texttt{n}}$ in (8.13), together with (4.43), Lemma 7.1, (8.4), (8.11), (2.9), (8.10), (8.19), (8.20), we have the quadratic estimate,

8.21 $$\begin{aligned} \Vert Q_{\texttt{n}} \Vert _{\mathcal {Y}^{s_0}}^{k_0,\upsilon }&\lesssim _{s_0} \sqrt{\varepsilon }\big ( \Vert H_{\texttt{n}+1} \Vert _{\mathcal {X}^{s_0}}^{k_0,\upsilon } \big )^2 \lesssim _{s_0} \sqrt{\varepsilon } \upsilon ^{- 2} K_\texttt{n}^{2 {\overline{\sigma }}} \big (\Vert \mathcal {F}({\widetilde{U}}_\texttt{n}) \Vert _{\mathcal {Y}^{s_0}}^{k_0, \upsilon } \big )^2 . \end{aligned}$$

Before estimating $P_{\texttt{n}}$, by (8.16) (applied with $s = s_0 + {\overline{\sigma }}$), we have

8.22 $$\begin{aligned} \begin{aligned} \Vert \mathcal {F}({\widetilde{U}}_{\texttt{n}}) \Vert _{\mathcal {Y}^{s_0+{\overline{\sigma }}}}^{k_0,\upsilon }&\leqslant \Vert \Pi _\texttt{n}\mathcal {F}({\widetilde{U}}_\texttt{n}) \Vert _{\mathcal {Y}^{s_0 + {\overline{\sigma }}}}^{k_0, \upsilon } + \Vert \Pi _\texttt{n}^\bot \mathcal {F}({\widetilde{U}}_\texttt{n}) \Vert _{\mathcal {Y}^{s_0 + {\overline{\sigma }}}}^{k_0, \upsilon } \\&{\mathop {\lesssim }\limits ^{(2.9)}} K_\texttt{n}^{{\overline{\sigma }}} \Vert \mathcal {F}({\widetilde{U}}_\texttt{n}) \Vert _{\mathcal {Y}^{s_0}}^{k_0, \upsilon } + K_\texttt{n}^{- {\texttt{b}}_1 + 2 {\overline{\sigma }}} \Vert \mathcal {F}({\widetilde{U}}_\texttt{n})\Vert _{\mathcal {Y}^{s_0 + {\texttt{b}}_1 - {\overline{\sigma }}}}^{k_0, \upsilon } \\&{\mathop {\lesssim }\limits ^{(8.16)}} K_\texttt{n}^{{\overline{\sigma }}} \Vert \mathcal {F}({\widetilde{U}}_\texttt{n}) \Vert _{\mathcal {Y}^{s_0}}^{k_0, \upsilon } + K_\texttt{n}^{- {\texttt{b}}_1 + 2 {\overline{\sigma }}} \big (\sqrt{\varepsilon } + \Vert {\widetilde{W}}_\texttt{n}\Vert ^{k_0, \upsilon }_{\mathcal {X}^{s_0 + {\texttt{b}}_1}} \big ) . \end{aligned}\nonumber \\ \end{aligned}$$

By (8.15), Theorem 7.6, (8.3), (8.16), (8.17), (8.22) and, by induction assumption, (8.4) at the step $\texttt{n}$, we have

8.23 $$\begin{aligned} \Vert P_{\texttt{n}} \Vert _{\mathcal {Y}^{s_0}}^{k_0,\upsilon }&\lesssim _{s_0} \upsilon ^{-1} \Vert \mathcal {F}({\widetilde{U}}_\texttt{n}) \Vert _{\mathcal {Y}^{s_0 + {\overline{\sigma }}}}^{k_0, \upsilon } \Vert \Pi _\texttt{n}\mathcal {F}({\widetilde{U}}_\texttt{n}) \Vert _{\mathcal {Y}^{s_0 + {\overline{\sigma }}}}^{k_0, \upsilon }, \nonumber \\&\lesssim \upsilon ^{- 1}K_\texttt{n}^{2 {\overline{\sigma }}} \big ( \Vert \mathcal {F}({\widetilde{U}}_\texttt{n}) \Vert _{\mathcal {Y}^{s_0}}^{k_0, \upsilon } \big )^2 + K_\texttt{n}^{- {\texttt{b}}_1 + 3 {\overline{\sigma }}} \big (\sqrt{\varepsilon } + \Vert {\widetilde{W}}_\texttt{n}\Vert ^{k_0, \upsilon }_{\mathcal {X}^{s_0 + {\texttt{b}}_1}} \big ) \upsilon ^{- 1} \Vert \mathcal {F}({\widetilde{U}}_\texttt{n}) \Vert _{\mathcal {Y}^{s_0}}^{k_0, \upsilon } \nonumber \\&{\mathop {\lesssim }\limits ^{(8.17)}} \upsilon ^{- 1}K_\texttt{n}^{2 {\overline{\sigma }}} \big ( \Vert \mathcal {F}({\widetilde{U}}_\texttt{n}) \Vert _{\mathcal {Y}^{s_0}}^{k_0, \upsilon } \big )^2 + K_\texttt{n}^{- {\texttt{b}}_1 + 3 {\overline{\sigma }}} \big (\sqrt{\varepsilon } + \Vert {\widetilde{W}}_\texttt{n}\Vert ^{k_0, \upsilon }_{\mathcal {X}^{s_0 + {\texttt{b}}_1}} \big ) . \end{aligned}$$

We now estimate $R_{\texttt{n}}$. By (2.9), (4.43), Lemma 7.1, (8.4), (8.15), (8.16), (8.17) and Theorem 7.6, one gets

8.24 $$\begin{aligned} \begin{aligned} \Vert R_{\texttt{n}} \Vert _{\mathcal {Y}^{s_0}}^{k_0, \upsilon }&\leqslant \Vert \Pi _\texttt{n}^\bot \mathcal {F}({\widetilde{U}}_\texttt{n}) \Vert _{\mathcal {Y}^{s_0}}^{k_0, \upsilon } + \Vert G_{\texttt{n}}\mathbf{{\Pi }}_\texttt{n}^\perp \textbf{T}_\texttt{n}\Pi _n\mathcal {F}({\widetilde{U}}_\texttt{n}) \Vert _{\mathcal {Y}^{s_0}}^{k_0, \upsilon } \\&\lesssim K_n^{{\overline{\sigma }} - {\texttt{b}}_1} \big (\sqrt{\varepsilon } + \Vert {\widetilde{W}}_\texttt{n}\Vert _{\mathcal {X}^{s_0 + {\texttt{b}}_1}}^{k_0, \upsilon } \big ) + \Vert \mathbf{{\Pi }}_\texttt{n}^\perp \textbf{T}_\texttt{n}\Pi _n\mathcal {F}({\widetilde{U}}_\texttt{n}) \Vert _{\mathcal {X}^{s_0 + 1}}^{k_0, \upsilon } \\&\lesssim K_n^{{\overline{\sigma }} - {\texttt{b}}_1} \big (\sqrt{\varepsilon } + \Vert {\widetilde{W}}_\texttt{n}\Vert _{\mathcal {X}^{s_0 + {\texttt{b}}_1}}^{k_0, \upsilon } \big ) + K_\texttt{n}^{- {\texttt{b}}_1} \Vert \textbf{T}_\texttt{n}\Pi _n\mathcal {F}({\widetilde{U}}_\texttt{n}) \Vert _{\mathcal {X}^{s_0 + {\texttt{b}}_1 + 1}}^{k_0, \upsilon } \\&\lesssim K_n^{{\overline{\sigma }} - {\texttt{b}}_1} \big (\sqrt{\varepsilon } + \Vert {\widetilde{W}}_\texttt{n}\Vert _{\mathcal {X}^{s_0 + {\texttt{b}}_1}}^{k_0, \upsilon } \big ) \\&+ K_\texttt{n}^{{\overline{\sigma }}- {\texttt{b}}_1} \upsilon ^{- 1} \Big ( \Vert \Pi _n\mathcal {F}({\widetilde{U}}_\texttt{n}) \Vert _{\mathcal {Y}^{s_0 + {\texttt{b}}_1 + 1}}^{k_0, \upsilon } + \Vert {\widetilde{W}}_\texttt{n}\Vert _{\mathcal {X}^{s_0 + {\texttt{b}}_1 + 1 }}^{k_0, \upsilon } \Vert \Pi _n \mathcal {F}({\widetilde{U}}_\texttt{n}) \Vert _{\mathcal {Y}^{s_0 }}^{k_0, \upsilon }\Big ) \\&\lesssim K_n^{{\overline{\sigma }} - {\texttt{b}}_1} \big (\sqrt{\varepsilon } + \Vert {\widetilde{W}}_\texttt{n}\Vert _{\mathcal {X}^{s_0 + {\texttt{b}}_1}}^{k_0, \upsilon } \big ) + K_\texttt{n}^{{\overline{\sigma }} + 1- {\texttt{b}}_1} \upsilon ^{- 1} \Vert \Pi _n\mathcal {F}({\widetilde{U}}_\texttt{n}) \Vert _{\mathcal {Y}^{s_0 + {\texttt{b}}_1 }}^{k_0, \upsilon } \\&\lesssim K_\texttt{n}^{2 {\overline{\sigma }} + 1 - {\texttt{b}}_1} \upsilon ^{- 1} \big ( \sqrt{\varepsilon } + \Vert {\widetilde{W}}_\texttt{n}\Vert _{\mathcal {X}^{s_0 + {\texttt{b}}_1}}^{k_0, \upsilon } \big ). \end{aligned}\nonumber \\ \end{aligned}$$

By (8.14), (8.21), (8.23), (8.24), (8.16), (8.17), we finally estimate $\mathcal {F}(U_{\texttt{n}+1})$ by

8.25 $$\begin{aligned} \begin{aligned}&\Vert \mathcal {F}(U_{\texttt{n}+1})\Vert _{\mathcal {Y}^{s_0}}^{k_0,\upsilon } \lesssim \upsilon ^{-1} K_{\texttt{n}}^{{\overline{\mu }}-\texttt{b}_1} (\sqrt{\varepsilon } + \Vert {\widetilde{W}}_{\texttt{n}} \Vert _{\mathcal {X}^{s_0+\texttt{b}_1}}^{k_0,\upsilon } )\\&\quad \quad + \upsilon ^{-1} K_{\texttt{n}}^{{\overline{\mu }}} \big ( \Vert \mathcal {F}({\widetilde{U}}_{\texttt{n}}) \Vert _{\mathcal {Y}^{s_0}}^{k_0,\upsilon } \big )^2 + \sqrt{\varepsilon } \upsilon ^{-2} K_\texttt{n}^{{\overline{\mu }}} (\Vert \mathcal {F}({\widetilde{U}}_{\texttt{n}})\Vert _{\mathcal {Y}^{s_0}}^{k_0,,\upsilon })^2 \\&{\mathop {\lesssim }\limits ^{\sqrt{\varepsilon } \upsilon ^{- 1} \ll 1}} \upsilon ^{-1} K_{\texttt{n}}^{{\overline{\mu }}-\texttt{b}_1} (\sqrt{\varepsilon } + \Vert {\widetilde{W}}_{\texttt{n}} \Vert _{\mathcal {X}^{s_0+\texttt{b}_1}}^{k_0,\upsilon } ) + \upsilon ^{-1} K_{\texttt{n}}^{{\overline{\mu }}} \big ( \Vert \mathcal {F}({\widetilde{U}}_{\texttt{n}}) \Vert _{\mathcal {Y}^{s_0}}^{k_0,\upsilon } \big )^2 , \end{aligned} \end{aligned}$$

with ${\overline{\mu }}:=3{\overline{\sigma }}+1$. Moreover, by (8.11), (8.9), (8.8), we have

8.26 $$\begin{aligned} \Vert W_{1} \Vert _{\mathcal {X}^{s_0+\texttt{b}_1}}^{k_0,\upsilon } = \Vert H_1 \Vert _{\mathcal {X}^{s_0+\texttt{b}_1}}^{k_0,\upsilon } \lesssim \upsilon ^{-1} \Vert \mathcal {F}(U_0) \Vert _{\mathcal {Y}^{s_0+{\overline{\sigma }}+\texttt{b}_1}}^{k_0,\upsilon } \lesssim \sqrt{\varepsilon } \upsilon ^{-1}, \end{aligned}$$

and, noting that $W_{\texttt{n}+1}={\widetilde{W}}_\texttt{n}+ H_{\texttt{n}+1}$ for $\texttt{n}\geqslant 1$, we have, by (8.18),

8.27 $$\begin{aligned} \Vert W_{\texttt{n}+1} \Vert _{\mathcal {X}^{s_0+\texttt{b}_1}}^{k_0,\upsilon } \lesssim \upsilon ^{-1} K_{\texttt{n}}^{{\overline{\mu }}} (\sqrt{\varepsilon } + \Vert {\widetilde{W}}_{\texttt{n}} \Vert _{\mathcal {X}^{s_0+\texttt{b}_1}}^{k_0,\upsilon }). \end{aligned}$$

We extend $H_{\texttt{n}+1}$ in (8.11), defined for $\omega \in \texttt{G}^{\upsilon }$, to ${\widetilde{H}}_{\texttt{n}+1}$ defined for all parameters $\lambda \in {{\mathbb {R}}}^{\kappa _{0}}\times \mathcal {J}_{\varepsilon }(\texttt{E})$ with equivalent $\Vert \,\cdot \, \Vert _{s}^{k_0,\upsilon }$ norms and we set ${\widetilde{U}}_{\texttt{n}+1}:= {\widetilde{U}}_{\texttt{n}} + {\widetilde{H}}_{\texttt{n}+1}$. Therefore, by (8.25), (8.26), (8.27), the induction assumptions, the choice of the constants in (8.2) and the smallness condition in (8.3), we conclude that (8.4), (8.5), (8.6), (8.7) hold at the step $\texttt{n}+1$. Finally, by (8.11), (4.43), (4.42), (4.44), Theorem 7.6 and the induction assumption on ${\widetilde{U}}_{\texttt{n}}$, we have that ${\widehat{{\mathfrak {I}}}}_{\texttt{n}+1}$ satisfies (4.45) and so ${\widetilde{U}}_{\texttt{n}+1}$ is a reversible embedding. This concludes the proof. $\square$

### Proof of Theorem 4.5

Let $\upsilon =\varepsilon ^{\texttt{a}}$, with $0<\texttt{a}< \texttt{a}_0$ (see (8.3)). Then, there exists $\varepsilon _0>0$ small enough such that the smallness condition (8.3) holds and Theorem 8.2 applies. By (8.5), the sequence ${\widetilde{W}}_\texttt{n}= {\widetilde{U}}_\texttt{n}- (\textbf{x},0,0,(\omega ,\texttt{A}))= ({\mathfrak {I}}_{\texttt{n}},\alpha _{\texttt{n}}-\omega )$ converges to a function $W_\infty :{{\mathbb {R}}}^{\kappa _0}\times \mathcal {J}_{\varepsilon }(\texttt{E})\rightarrow H_\textbf{x}^{s_0} \times H_\textbf{x}^{s_0} \times H^{s_0,1}\times {{\mathbb {R}}}^{\kappa _0}$ and we define

$$\begin{aligned} U_\infty :=(i_\infty ,\alpha _{\infty }) = (\textbf{x},0,0,(\omega ,\texttt{A})) + W_\infty . \end{aligned}$$

The torus $i_\infty$ is reversible; namely, it satisfies (4.45). Moreover, by (8.4) and (8.5), we deduce that

$$\begin{aligned} \Vert U_\infty - U_0 \Vert _{s_0+{\overline{\sigma }}} \leqslant C_* \sqrt{\varepsilon }\upsilon ^{-1} , \quad \Vert U_\infty -{\widetilde{U}}_{\texttt{n}} \Vert _{s_0+{\overline{\sigma }}}^{k_0,\upsilon } \leqslant C_* \sqrt{\varepsilon }\upsilon ^{-1} K_{\texttt{n}}^{-\texttt{a}_2}, \ \forall \, \texttt{n}\geqslant 1. \end{aligned}$$

In particular, (4.52), (4.53) hold. By Theorem 8.2-$(\mathcal {P}2)_{\texttt{n}}$, we deduce $\mathcal {F}(\omega ,U_\infty (\omega ))=0$ for any $(\omega ,\texttt{A})\in \texttt{DC}(\upsilon , \tau )\times \mathcal {J}_{\varepsilon }(\texttt{E})$ and hence also for $(\omega ,\texttt{A}) \in \texttt{G}^{\upsilon }\times \mathcal {J}_{\varepsilon }(\texttt{E})$ (see (4.54), (1.21)), where the set ${\mathtt {\Omega }}$ in (4.54) is the $\varrho$-neighbourhood of the unperturbed linear frequencies in (4.51). This concludes the proof of Theorem 4.5. $\square$

## Data Availability

Data availability is not applicable to this article as no new data were created or analysed in this study.
